# Targeted Capture Sequencing in Whitebark Pine Reveals Range-Wide Demographic and Adaptive Patterns Despite Challenges of a Large, Repetitive Genome

**DOI:** 10.3389/fpls.2016.00484

**Published:** 2016-04-21

**Authors:** John V. Syring, Jacob A. Tennessen, Tara N. Jennings, Jill Wegrzyn, Camille Scelfo-Dalbey, Richard Cronn

**Affiliations:** ^1^Department of Biology, Linfield College, McMinnvilleOR, USA; ^2^Department of Integrative Biology, Oregon State University, CorvallisOR, USA; ^3^Department of Botany and Plant Pathology, Oregon State University, CorvallisOR, USA; ^4^Department of Ecology and Evolutionary Biology, University of Connecticut, StorrsCT, USA; ^5^Jack Baskin School of Engineering, University of California, Santa Cruz, Santa CruzCA, USA; ^6^Pacific Northwest Research Station, United States Department of Agriculture, Forest Service, CorvallisOR, USA

**Keywords:** *Pinus albicaulis*, whitebark pine, gene conservation, target enrichment, SNP, landscape genomics

## Abstract

Whitebark pine (*Pinus albicaulis*) inhabits an expansive range in western North America, and it is a keystone species of subalpine environments. Whitebark is susceptible to multiple threats – climate change, white pine blister rust, mountain pine beetle, and fire exclusion – and it is suffering significant mortality range-wide, prompting the tree to be listed as ‘globally endangered’ by the International Union for Conservation of Nature and ‘endangered’ by the Canadian government. Conservation collections (*in situ* and *ex situ*) are being initiated to preserve the genetic legacy of the species. Reliable, transferrable, and highly variable genetic markers are essential for quantifying the genetic profiles of seed collections relative to natural stands, and ensuring the completeness of conservation collections. We evaluated the use of hybridization-based target capture to enrich specific genomic regions from the 27 GB genome of whitebark pine, and to evaluate genetic variation across loci, trees, and geography. Probes were designed to capture 7,849 distinct genes, and screening was performed on 48 trees. Despite the inclusion of repetitive elements in the probe pool, the resulting dataset provided information on 4,452 genes and 32% of targeted positions (528,873 bp), and we were able to identify 12,390 segregating sites from 47 trees. Variations reveal strong geographic trends in heterozygosity and allelic richness, with trees from the southern Cascade and Sierra Range showing the greatest distinctiveness and differentiation. Our results show that even under non-optimal conditions (low enrichment efficiency; inclusion of repetitive elements in baits), targeted enrichment produces high quality, codominant genotypes from large genomes. The resulting data can be readily integrated into management and gene conservation activities for whitebark pine, and have the potential to be applied to other members of 5-needle pine group (*Pinus* subsect. *Quinquefolia*) due to their limited genetic divergence.

## Introduction

**FIGURE 1:**
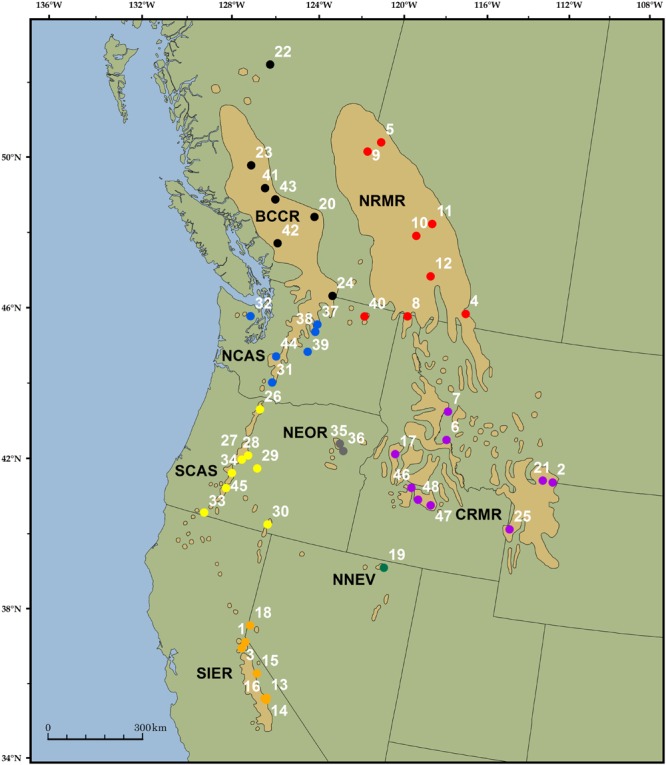
**Range of whitebark pine (tan polygons; Little, 1971) and the location of the 48 trees included in the analysis.** Unique colors and associated four-letter abbreviations represent groups of samples chosen *a priori* based on previous research (Richardson et al., 2002). Sample 42 was excluded from sample-specific analyses due to poor quality sequencing.

Whitebark pine (*Pinus albicaulis*) has an expansive range occurring across ∼340,000 square kilometers in the western U.S. and Canada (**Figure 1**; Wilson, 2007; COSEWIC, 2010). There are an estimated 200 million individuals of whitebark pine in Canada (COSEWIC, 2010), with a potential 300–400 million individuals range-wide. This species has proven susceptible to an interconnected suite of threats that include anthropogenic climate change, white pine blister rust (*Cronartium ribicola*), mountain pine beetle outbreaks (*Dendroctonus ponderosae*), and fire exclusion; as a consequence, the species is suffering tremendous mortality across its range (Kendall and Keane, 2001; Warwell et al., 2007; Aubry et al., 2008; Gibson et al., 2008; Bockino and Tinker, 2012). At the ongoing rate of decline of 1.5–3.5%/year in Alberta and British Columbia, population reductions between 78–97% are predicted within the next 100 years (COSEWIC, 2010). Populations in the U.S. are equally threatened, with reported declines over the last several decades ranging between ∼40% (western Cascade Range, Washington; Shoal and Aubry, 2004) to 70% (eastern California; Millar et al., 2012). As a result of these reported declines, whitebark pine is globally assessed as endangered by the IUCN (2015), regionally endangered by the Canadian government (COSEWIC, 2010), and has been determined to be “warranted” for listing under the U.S. Endangered Species Act (currently withheld due to funding limitations; US Fish and Wildlife Service, 2011).

An understanding of the amount and apportionment of spatial genetic diversity is essential for science-based management and restoration, but these needs have not been adequately met for whitebark pine due to historical limitations in available genetic markers for pines, a group that is species-rich (>100; Price et al., 2000) and defined by large (up to 30 Gbp; Morse et al., 2009) and complex genomes. Allozymes and organelle DNA sequences have seen the widest application in pines (Jorgensen and Hamrick, 1997; Rogers et al., 1999; Richardson et al., 2002; Bower and Aitken, 2007; Mahalovich and Hipkins, 2011), but these methods suffer from low power and/or uniparental inheritance. The advent of next-generation genomics technologies has ushered an explosion in methods that can be reliably applied to non-model species (Cronn et al., 2012; Jones and Good, 2016), and this offers new opportunities to develop genetic markers that are ideally suited for addressing gene conservation in whitebark and other imperiled conifers. Even with advancements in DNA sequencing, genomes of this size cannot be affordably sequenced for population-level studies. Instead, methods that reduce the complexity of the genome to a reliably sampled subset of loci are more cost-effective. Hybridization-based capture methods have emerged as one of the most powerful methods for developing codominant genetic markers with high transferability across laboratories and studies, populations, and possibly species (Gnirke et al., 2009; Cronn et al., 2012; Tennessen et al., 2012; Hebert et al., 2013; Neves et al., 2013; Jones and Good, 2016; Müller et al., 2015), and they have recently been successfully applied to evaluate diversity in conifers with large-genomes (Neves et al., 2013; Müller et al., 2015; Pavy et al., 2016).

Whitebark pine is a superb organism in which to test solution hybridization methods for use in conifer conservation because of its large and complex genome, its expansive and highly fragmented range, and the management implications resulting from an understanding of its population structure. The species spans nearly 21° latitude from the southern Sierras to northern British Columbia, and 28° longitude from the Cascade Mountains of the Pacific Northwest to the Rocky Mountains of Alberta and the US (**Figure 1**). It forms mixed or pure populations in high elevation landscapes, resulting in a highly fragmented distribution across the landscape, with many stands geographically isolated on high elevation ‘tree islands’ (Arno and Hoff, 1989; Jorgensen and Hamrick, 1997; Keane et al., 2011).

Whitebark pine is unusual among conifers in having no commercial value, but it is a keystone species of subalpine environments and it provides vital ecosystem services (Mills et al., 1993; Mattson et al., 2001; Tomback et al., 2001; Aubry et al., 2008). Whitebark’s large wingless seeds are an important food source for mammals and birds (Keane et al., 1990), and trees provide nesting sites, cover, and protection for species associated with alpine ecosystems. The crowns help to accumulate snow during winter, and the canopy provides shade that increases snowpack retention and delays snow melt during the growing season (Farnes, 1990; Tomback et al., 2001). The health of this species directly impacts the ecological health of subalpine environments, as well as downslope ecosystems (Ellison et al., 2005). Population structure in whitebark pine has been heavily influenced by Pleistocene glaciation, which resulted in a pattern of northward and higher elevation recolonization of Canada from multiple lower-elevation refugia following deglaciation (Richardson et al., 2002; Krakowski et al., 2003; Shafer et al., 2010). Whitebark pine is one of a small number of pines that is bird dispersed, with the primary disperser being the Clark’s nutcracker (*Nucifraga columbiana*). Seed dispersal by nutcrackers has a strong impact on genetic structure (Furnier et al., 1987; Richardson et al., 2002), with birds traveling from several 100 m to >10 km to cache seeds (Tomback, 2005).

Due to their wind pollination syndrome, conifers typically show low among-population variation and very high within-stand variation (Jorgensen and Hamrick, 1997; Rogers et al., 1999; Petit and Hampe, 2006). Whitebark pine shows a similar structure, with low allozyme-based estimates of differentiation (F_ST_ ≈ 0.06 (Bruederle et al., 2001; Krakowski et al., 2003; Bower and Aitken, 2008), indicating that most diversity is found within populations, with limited genetic structure except at larger geographic scales (Jorgensen and Hamrick, 1997; Bruederle et al., 2001; Bower and Aitken, 2008). Despite evidence for low neutral marker differentiation, significant differences in quantitative phenotypic traits (height growth, cold hardiness, timing of needle flush) have been shown to exist over smaller geographic scales (Bower and Aitken, 2007, 2008). The nature of this variation is largely clinal and indicative of adaptation to local environments (COSEWIC, 2010). Clinal patterns have also been detected in allozymes, as correlations between observed heterozygosity and geographic variables have been shown to be significant (*R*^2^ = 0.36, latitude; *R*^2^ = 0.30 longitude) (Krakowski et al., 2003). Evidence for strong population uniqueness (e.g., private alleles or haplotypes) is generally lacking in the species, although Richardson et al. (2002) found that populations from Yellowstone and the Southern Sierras were significantly divergent from the rest of the population with regard to chloroplast and mitochondrial genome haplotypes.

In this paper, we use hybridization-based capture probes to assess sequence variability across enriched targets from whitebark pine across its range. We designed capture probes that targeted 200 contiguous bases from genomic regions encoding 7,849 expressed transcripts, and used these probes to enrich targets from 48 trees spanning the geographic (and presumably genetic) range of whitebark pine. Our results show that the inclusion of repetitive genomic elements in the probe pool hampered the efficiency of even enrichment across targets, but that the resulting dataset still permitted interrogation of 4,452 transcripts and 32% of targeted positions (528,873 sites), revealing 12,390 segregating sites. Analysis of population variation based on this subset of information reveals strong geographic trends in heterozygosity and allelic richness, with trees from the southern Cascade and Sierra Range showing the greatest distinctiveness and differentiation. Our study shows that target enrichment generates high quality codominant genotypes, even when enrichment is low and repetitive loci are included in the bait pool, and that the resulting data are relevant to management and gene conservation activities.

## Materials and Methods

### Sample Collections and DNA Extraction

Forty-eight samples were collected from across the range of whitebark pine, each representing a unique location (**Figure 1**, Supplementary Table S1). Sampling was stratified by geographic region to approximately match provinces identified as ‘distinctive’ based on mitochondrial DNA surveys (Richardson et al., 2002). These *a priori* groupings were used to calculate F_ST_ (see below). We included 1 – 9 trees per region: NRMR (Northern Rocky Mountain Range, *N* = 8), CRMR (Central Rocky Mountain Range, *N* = 9), BCCR (British Columbia Coast Range, *N* = 7), NCAS (North Cascades, *N* = 7), SCAS (Southern Cascades, *N* = 7), NEOR (Northeastern Oregon, *N* = 2), NNEV (Northern Nevada, *N* = 1) and SIER (Sierra Mountain Range, *N* = 7) (Supplementary Table S1). Diploid genomic DNA was extracted from needles (35 samples) or seed embryos (13 samples) using the FastDNA Kit (Qbiogene, Irvine, CA, USA). A total of 50–100 mg of tissue was used for needle extractions, while entire embryos were dissected and used in extractions.

### Probe Design, Library Construction, and DNA Sequencing

Hybridization probes for this study were developed using two sources of information. First, we chose an initial set of target loci that previously showed a high degree of hybridization-enrichment success in loblolly pine (Neves et al., 2013). Briefly, the authors developed hybridization probes for 14,729 loblolly pine transcripts, and reported high-success for 11,552 loci. Homologs of these ‘high-success’ loblolly pine transcripts were identified from a draft needle transcriptome for whitebark pine (Baker et al., in review) using BLASTN (Altschul et al., 1990) and an *e*-value cutoff of 1 e-50. BLAT (Kent, 2002) was used to identify and remove chloroplast (NCBI sequence FJ899566.1) and mitochondrial (Neale et al., 2014 ^1^) transcripts, and this resulted in a final list of 7,855 transcripts for probe design. Sequences were masked using RepeatMasker v. 4.0 (Smit et al., 2014) for simple repeats and low complexity DNA, and individual 100-mer probes were localized to the 5′ end of each transcript, with two probes per locus covering 200 contiguous bases (no probe overlap). In a small number of cases, probes were moved 3′ to avoid masked repeats. The final bait pool was synthesized by MycroArray LLC (MyBaits; Ann Arbor, MI, USA) and it included 15,698 probes spanning 1,569,800 bp from 7,849 unique transcripts. The whitebark pine transcriptome reference used in the design of hybridization probes, the sequences of the hybridization probes, and a summary of homologous regions in sugar pine to each pair of hybridization probes are available in the TreeGenes database ^2^.

Genomic DNAs (∼500 – 1,000 ng) were sheared to ∼200 bp fragments by sonication for 15–45 min (Diagenode BioRuptor; Denville, NJ, USA) and converted into standard Illumina sequencing libraries (NEBNext; New England Biolabs, Ipswich, MA, USA) with 24 unique index sequencing adapters, as previously described (Weitemier et al., 2014). Library DNA concentration was determined by fluorometry (Qubit High Sensitivity; Thermo-Fisher, Waltham, MA, USA), and libraries were pooled into equimolar 12-plexes containing 1,000 ng of total DNA. Target enrichment for 48 DNAs was conducted using 12-plexes (four capture reactions) following standard protocols from the manufacturer (MycroArray MyBait v. 2.0). Enriched pools were amplified using eight cycles, DNA concentration was determined by fluorometry, and 12-plexes were pooled to produce two equimolar 24-plexes. These 24-plexes were diluted to 10 pM, and 11 pmol was sequenced on two lanes of the HiSeq 2500 using paired-end 100 bp reads (University of Oregon Genomics Center^3^). Base calling and sample demultiplexing followed standard Illumina workflows. A flowchart showing the steps involved in probe design, library construction, and data analysis is provided in Supplementary Figure S1.

### Sequence Analysis

We determined genotypes from Illumina data following previously described methods (Tennessen et al., 2013). In brief, we first filtered Illumina reads in FASTQ format by converting all base calls with Phred-scaled quality scores less than 20 to missing (N), and removing all reads with fewer than 75 non-missing sites. Samples with fewer than 4 million retained reads were excluded from most analyses. We used BWA aln (Li and Durbin, 2009) to align reads to the original whitebark pine transcript sequences from which baits had been designed, using the default BWA maximum edit distance of 0.04, and converted genotypes to vcf format using SAMtools (Li et al., 2009). We retained genotypes as valid only if depth was between 4 and 50, the Phred-scaled likelihood was 0, and Phred-scaled likelihood for all other potential genotypes was greater than or equal to a threshold that scaled with depth, following -10(log_10_(1/(2ˆD))), where D = depth, up to a maximum of threshold of 50. Genotypes that failed any of these criteria were considered missing, and sites with more than 10 missing genotypes were discarded. We also discarded all sites from any transcript in which any site violated Hardy–Weinberg equilibrium with *p* < 0.001 due to an excess of heterozygotes, as these could contain sequence from multiple paralogs; transcripts with homozygote excess were retained as these could be due to population structure. All sites were treated as bi-allelic, with the most common observed allele as the major allele and all other observed alleles binned as the minor allele, with frequency denoted as minor allele frequency (MAF).

In order to examine variation in coverage among targets, we counted the number of reads aligning to each transcript among the 48 samples. In order to find homologous genomic repeats, we ran BLAT for each probe against the draft sugar pine genome (*P. lambertiana*^4^), divided into 171 smaller files for computational ease, with minScore = 90 and stepSize = 1. We counted any *P. lambertiana* sequence matching either of a transcript’s probes as a homologous region for that transcript.

### Population Genetic Analysis

We calculated genetic diversity (π) and Tajima’s D, both across the entire dataset and for each transcript separately. We annotated transcripts by searching for open reading frames of at least 300 bp, and classifying variants as synonymous, non-synonymous, nonsense (changing a stop codon), or non-coding. For each transcript, we also calculated the number of variants, the number of sites retained, and the mean depth at retained sites.

We performed principal component analysis (PCA) on genotypes at segregating sites. We first replaced all missing genotypes with the most common genotypes for that site. That is, if MAF < 1/3, then major allele homozygotes (expected frequency > 44%) are more common than heterozygotes (expected frequency < 44%) and missing genotypes were assumed to be major allele homozygotes; otherwise missing genotypes were assumed to be heterozygotes. We calculated F_ST_ for all sites among the six geographic groupings that contained at least six samples. We only included variants with MAF at least 10% in our F_ST_ calculations, since rare variants have a low maximum F_ST_. We also calculated pairwise composite linkage disequilibrium (LD) as Δ_AB_ among all pairs of variants on different genes with MAF at least 10%, using the Perlymorphism package (Stajich and Hahn, 2005). We considered Δ_AB_ values of at least 0.15 to be meaningful. We excluded exceptionally divergent subpopulations from LD analysis, to minimize the effect of population structure on LD.

To identify variants that may show signatures of adaptation to local environments, we first used the ClimateNA v5.10 software package^5^ (Hamann et al., 2013) to assign climatic variables to all collection sites based on their latitude and longitude. We chose eight climate variables to examine based on their likely importance to *P. albicaulis* fitness: mean warmest month temperature (MWMT), mean summer precipitation (MSP), summer heat:moisture index (SHM), mean annual temperature (MAT), DD > 5 (degree days above 5°C), Eref (Atmospheric evaporative demand), mean coldest month temperature (MCMT), and frost free period (FFP). For all variants with MAF at least 10%, we split genotypes into two variables corresponding to the two alleles (i.e., a homozygote for the major allele would be 0,0, a heterozygote would be 0,1, etc.), Then, for each of the eight climate variables, we ran logistic regression with the climate variable as an independent variable and genotype as the response. Because population structure could largely be approximated by latitude (see Results), we included latitude as a second independent variable to account for population structure. We also ran a logistic regression using only latitude, and no climate variables, as the independent variable. To evaluate the significance of *p*-values, we used a Benjamini and Hochberg (1995) false discovery rate correction for the number of variants examined. Genes showing signs of local adaptation were annotated using BLASTP searches of the translated open reading frame to the NR database.

## Results

### Targeted Capture Sequencing

**FIGURE 2:**
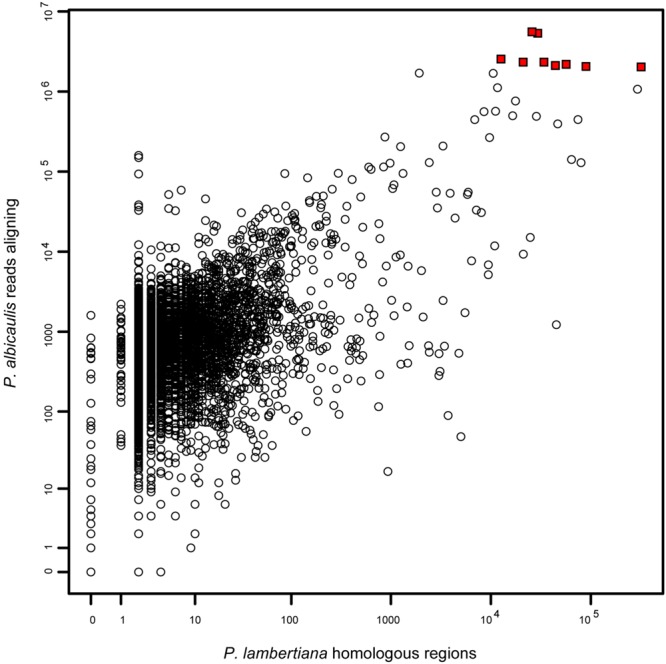
**Coverage in *Pinus albicaulis* (aligned reads among all 48 samples) per transcript (median = 735), as a function of homologous regions in the *P. lambertiana* genome (median = 2) (log-log plot).** Representation of transcript sequences was correlated among datasets, indicating that probes matching a large number of *P. albicaulis* reads are likely highly repetitive in the genome. The nine transcripts indicated by red squares all captured over two million reads each and together represent >50% of all aligning reads. The cause of this overrepresentation is that these regions are among the top 0.25% most repetitive regions in the genome, all with over 10,000 homologous regions found. Removal of a small number of highly repetitive probes in future studies would substantially increase the coverage of all other loci.

We observed 390,910,265 reads among the 48 samples, and all Illumina reads have been submitted to SRA (Bioproject Accession PRJNA300288). Most of these reads (87%) did not align to targeted transcripts. Of those that did align, a majority (54%) aligned to just 9 transcripts (0.1% of all targeted transcripts), and 82% of aligning reads matched 1% of all targeted transcripts. By searching for homology to the recently released draft genome of the closely related congener *P. lambertiana*, we observed 1,637,029 regions showing homology to one of our probes. This distribution was also highly skewed, with five transcripts representing a majority (52%) of homologous regions. Most (54%) transcripts had exactly two homologous regions, indicating a single-copy gene because all transcripts had two non-overlapping probes. Coverage in *P. albicaulis* was highly correlated with repetitiveness in *P. lambertiana* (*R*^2^ = 0.16; *p* < 10^-15^; **Figure 2**), indicating that a small set of probes captured an inordinate number of reads because they match highly repetitive sequences.

### Genotype Data

**FIGURE 3:**
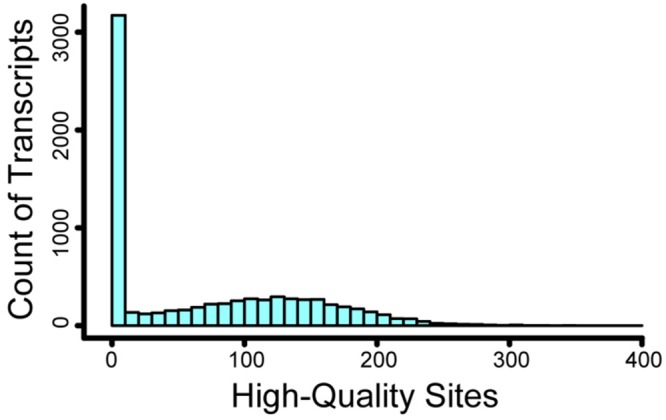
**Histogram of high-quality sites per transcript.** For over 3000 transcripts, we recovered no high-quality sites. For the remaining transcripts, the number of high-quality sites was approximately normally distributed with a mean of 119.

Of 106,011,302 sites (sites with non-zero coverage per individual, summed over all accessions), 46.2% were excluded due to insufficient depth (1–3× coverage), and 3.3% were excluded due to excessive depth (>50× coverage) and therefore increased likelihood of parology. After additional quality filtering, we observed 528,873 high-quality nucleotide positions passing all filters, including 440,670 high-quality sites within targeted regions (of 1,569,800 targeted nucleotides) and 88,203 sites in flanking regions. We observed no high-quality sites for 3,042 of the transcripts, and we excluded 359 transcripts for Hardy–Weinberg violation. For the remaining 4,452 transcripts, the number of high-quality sites was approximately normally distributed with mean 118.8 and standard deviation 60.9 (**Figure 3**). Among these 4,452 successful transcripts, a majority (2,420) had high-quality sites at 100 or more of the 200 targeted contiguous positions. Across the 528,873 high-quality positions, we observed 12,390 segregating polymorphisms, including 2,163 variants with MAF > = 10%. There were 9,570 non-coding variants, including 719 non-coding indels and 8,851 non-coding single-nucleotide polymorphisms. The remaining 2,820 variants were associated with coding regions, of which 1,739 were non-synonymous, 852 were synonymous, 61 were nonsense, 27 were in-frame indels, 76 were frameshift indels, and 65 could not be unambiguously annotated because they overlapped more than one open reading frame. Genome-wide, π is 0.26% and Tajima’s *D* is -1.47. Tajima’s *D* was -1.43 at non-coding single-nucleotide polymorphisms and -1.42 at synonymous sites, reflecting demographic effects without the influence of natural selection. In contrast, Tajima’s *D* is -1.76 at non-synonymous sites, -1.69 at frameshift indels, and -2.00 at nonsense sites; these lower values likely reflect the effects of purifying selection keeping deleterious alleles rare. Among genes, π ranges from 0.00 to 3.39% (0.00–1.70% for transcripts with ≥100 high quality sites) and Tajima’s *D* ranges from –2.42 to 2.83 (Supplementary Table S2). One of our samples (accession 42, **Figure 1**) had fewer high-quality reads than the rest (2.2 million, versus a minimum of 4.5 million for all others); as a result, it had missing genotypes for more than half of all high-quality segregating sites, and for this reason accession 42 was excluded from subsequent sample-specific analyses. Overall, 12.85% of genotypes were missing at segregating sites; after excluding accession 42, 11.96% of genotypes were missing.

### Geographical Partitioning of Genetic Diversity

**FIGURE 4:**
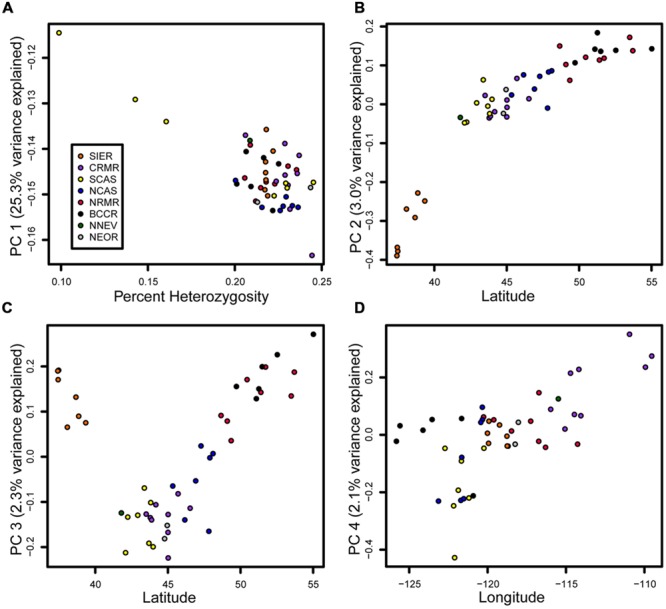
**Principal components (PCs) tied to heterozygosity, latitude, and longitude.** Samples are colored by geographic group as in **Figure 1**. **(A)** PC 1 vs. heterozygosity. **(B)** PC2 vs. latitude. **(C)** PC3 vs. latitude. **(D)** PC4 vs. longitude.

The PCA largely separates samples in a manner consistent with geographical location (**Figure 4**). The first principle component (PC), explaining 25.3% of the variance, was strongly correlated with heterozygosity (*R*^2^ = 0.57, *p* < 10^-9^). Strikingly, three samples stood out as having low heterozygosity (0.099–0.161%, mean = 0.134%) relative to all other samples (0.200–0.245%, mean = 0.222%), and these samples all originated from southwestern Oregon (accessions 33, 34, and 45). The second PC, explaining 3.0% of the variance, was strongly correlated with latitude (*R*^2^ = 0.81, *p* < 10^-15^). However, its main effect was to separate the seven SIER samples from all other samples (*R*^2^ = 0.79, *p* < 10^-15^). The third PC, explaining 2.3% of the variation, was strongly correlated with latitude among the non-SIER samples (*R*^2^ = 0.84, *p* < 10^-15^). The fourth PC, explaining 2.1% of the variation, was correlated with longitude (*R*^2^ = 0.42, *p* < 10^-6^). All remaining PCs explained less than 2% of the variation and were ignored.

Mean heterozygosity within geographic groupings ranged from 0.190% in SCAS to 0.230% in CRMR (**Table 1**). Pairwise F_ST_ values between geographic groupings ranged from 0.001 to 0.056, and the highest values were seen between SIER and other groups (**Table 2**). These results mirror the PCA results, in which the clearest population division was between SIER and the rest (pairwise F_ST_ = 0.033). Nearly all comparisons showed at least one variant with pairwise F_ST_ > 0.7, and several comparisons, mostly those including SIER, showed variants with pairwise F_ST_ over 0.8 or even 0.9 (**Table 2**). These high-F_ST_ outliers could reflect geographically varying selection.

**Table 1:** Mean per-individual observed heterozygosities for the eight designated geographic groups organized from highest to lowest heterozygosity.

Group	Sample size analyzed	Heterozygosity (%)
CRMR	9	0.2297
NEOR	2	0.2283
NCAS	7	0.2241
NRMR	8	0.2186
SIER	7	0.2185
BCCR	6	0.2134
NNEV	1	0.2087
SCAS	7	0.1899

**Table 2:** Pairwise *F*_ST_ among all groups, and outlier genes showing unusually high *F*_ST_ in each comparison.

Group 1	Group 2	Mean *F*_ST_	Genes with *F*_ST_ > 0.8	Annotation
SIER	BCCR	0.0560	comp64228c0	Zinc metalloprotease
			comp64269c0	Ataxin
			comp69504c0	Uncharacterized
SIER	NRMR	0.0457	comp70837c0	Potassium transporter 5
			comp65717c0	Calcineurin-like metallo-phosphoesterase
			comp56653c0	Pgr5 like protein
			comp43956c0	Starch synthase 3
			comp67607c0	5′-nucleotidase domain-containing protein
			comp43662c0	ADP-glucose pyrophosphorylase subunit
SIER	CRMR	0.0327	comp65717c0	Calcineurin-like metallo-phosphoesterase
			comp63892c0	Uncharacterized
			comp66180c0	Synaptotagmin-5
			comp67607c0	5′-nucleotidase domain-containing protein
			comp69349c0	LysM domain receptor-like kinase
			comp43662c0	ADP-glucose pyrophosphorylase subunit
SIER	NCAS	0.0361	comp67607c0	5′-nucleotidase domain-containing protein
			comp60424c0	Pentatricopeptide repeat-containing protein
			comp43662c0	ADP-glucose pyrophosphorylase subunit
			comp68278c0	Universal stress protein A-like protein
SIER	SCAS	0.0387	comp71122c0	Ubiquitin-like-specific protease
			comp70837c0	Potassium transporter 5
			comp69943c0	Shikimate dehydrogenase/3-dehydroquinate dehydratase
			comp67607c0	5′-nucleotidase domain-containing protein
			comp68250c0	UBX domain protein
			comp60736c2	5′-3′ exoribonuclease
			comp64269c0	Ataxin
BCCR	NCAS	0.0096	na	na
BCCR	SCAS	0.0266	comp78339c0	F-box protein
			comp70216c0	No apical meristem-like protein
BCCR	NRMR	0.0050	na	na
BCCR	CRMR	0.0249	na	na
NCAS	SCAS	0.0068	na	na
NCAS	NRMR	0.0013	na	na
NCAS	CRMR	0.0047	na	na
SCAS	NRMR	0.0186	comp67394c0	Uncharacterized
SCAS	CRMR	0.0121	na	na
NRMR	CRMR	0.0106	na	na

*Transcript names and their corresponding annotation using BLASTP searches of the translated open reading frame to the NR database are provided.*

**FIGURE 5:**
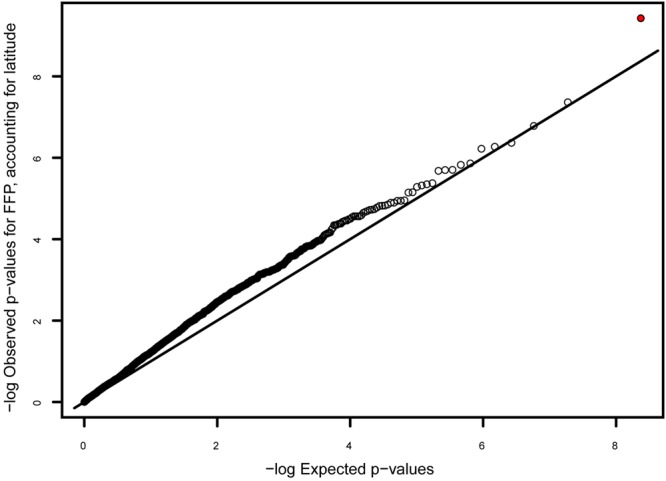
**Q–Q plot of *p*-values for the correlation between genotype and frost free period (FFP), after accounting for latitude.** The genotype with the best correlation (indicated with red circle) occurs in gene comp61071c1, encoding a putative non-lysosomal glucosylceramidase, and has a low, though not significant, *p*-value (*p* = 8 × 10^-5^; α = 2 × 10^-5^).

None of the eight climate variables were significantly correlated with any variant after accounting for latitude under Benjamini–Hochberg correction (α for lowest-ranked SNP = 0.05/2163 = 2.3 × 10^-5^). The only *p*-value less than 10^-4^ was for the correlation between FFP and a variant in gene comp61071c1, encoding a putative non-lysosomal glucosylceramidase (*p* = 8.05 × 10^-5^; **Figure 5**). There were 13 SNPs in 12 genes showing a significant correlation with latitude after Benjamini–Hochberg correction (**Table 3**), with the best correlation seen in gene comp70653c0, encoding a putative GTP diphosphokinase RSH1 (*p* = 1.5 × 10^-5^; Supplementary Figure S2). Four of these genes had been identified as outliers in the F_ST_ analysis (**Table 2**), and three were in known LD blocks (see below; Supplementary Table S2).

**Table 3:** SNPs showing a significant correlation with latitude after Benjamini–Hochberg correction.

Gene	SNP Position	*p*-value	High *F*_ST_	LD block	ORF	Annotation
comp70653c0	145	1.54 × 10^-5^	No	No	901 aa	GTP diphosphokinase RSH1
comp59876c0	241	3.60 × 10^-5^	No	No	1238 aa	RNA polymerase
comp65093c0	116	6.22 × 10^-5^	No	No	356 aa	Jasmonate-zim-domain protein
comp43662c0	129	7.40 × 10^-5^	Yes	No	524 aa	ADP-glucose pyrophosphorylase subunit
comp78339c0	70	7.73 × 10^-5^	Yes	Yes	451 aa	F-box protein
comp64269c0	105; 224	1.53 × 10^-4^; 8.25 × 10^-5^	Yes	No	446 aa	Ataxin
comp67607c0	197	9.08 × 10^-5^	Yes	No	694 aa	5′-nucleotidase domain-containing protein
comp69788c0	87	1.58 × 10^-4^	No	No	377 aa	Transcription factor
comp68536c0	205	1.75 × 10^-4^	No	Yes	364 aa	Uncharacterized
comp53319c0	146	2.20 × 10^-4^	No	Yes	No	NA
comp64449c0	146	2.33 × 10^-4^	No	No	No	NA
comp67186c0	173	2.59 × 10^-4^	No	No	355 aa	Werner Syndrome-like exonuclease

### Linkage Disequilibrium

We excluded SIER and NNEV from LD analysis due to divergence from other groups. There were 80 pairs of variants on 106 genes with MAF > = 10% showing high LD with Δ_AB_ at least 0.15. These could be grouped in 43 blocks of 2–9 genes each, with each gene in a block showing high LD with at least one other gene in that block. Genes in the same block are likely to be closely physically linked (**Table 4**). The highest observed value of Δ_AB_ between different genes was 0.27 (*r*^2^ = 0.84), between comp61838c0 and comp68040c0.

**Table 4:** Blocks of high linkage disequilibrium (LD), in which genes show LD of at least 0.15 with at least one other gene in the block.

Count	Genes
9	comp67636c0,comp58128c0,comp35949c0,comp72449c0,comp60766c0,comp61270c0,comp68485c0,comp64341c1,comp68536c0
5	comp68325c0,comp65744c0,comp64425c0,comp78339c0,comp64268c0
4	comp62424c0,comp65919c0,comp64896c0,comp56342c0
4	comp70822c0,comp68040c0,comp68019c0,comp61838c0
3	comp69667c0,comp65771c1,comp69440c0
3	comp44144c1,comp45755c0,comp63251c0
3	comp65180c0,comp62519c1,comp67420c0
3	comp55192c0,comp63411c0,comp70660c1
3	comp70381c0,comp64546c0,comp62234c0
3	comp63248c0,comp71610c0,comp67467c0
2	34 pairs of markers

*Ten blocks included three or more markers, while 34 blocks included only two markers (listed in Supplementary Table S2).*

## Discussion

### Development of Genomic Markers for Whitebark Pine

Next-generation sequencing has fostered the development of methods that permit rapid and direct genotyping for large numbers of non-model individuals. At present, these genotyping methods are based on divergent approaches that have been used for decades to reduce the complexity of genomes – reduction via restriction-digestion, and reduction via targeted hybridization. Of the two approaches, restriction-digestion methods are usually less expensive, and so are quickly becoming a method of choice for linkage mapping of defined pedigrees, identifying SNP variation for use on other genotyping platforms, for directly assessing diversity in crop germplasm, and screening natural populations (summarized in Davey et al., 2011; Narum et al., 2013).

While restriction-based approaches are powerful in these kinds of narrow applications, restriction methods have drawbacks that make them less attractive as a marker for gene conservation activities. First, since these methods sample across a large number of potential targets, low genomic coverage sequencing produces data sets that have missing data. This issue can be exacerbated in conifer genomes, where the large numbers of restriction sites decrease the probability of sampling any particular locus across every individual in a study. Additionally, restriction-site methods can impart a selection bias at specific restriction sites that excludes lineage-specific variation (Arnold et al., 2013; Gautier et al., 2013). Simulations have shown that the scale of the problem is proportional to the number of restriction sites used for sampling (Arnold et al., 2013); this means that popular two enzyme methods (e.g., GBS, ddRAD) should show stronger biases than single enzyme methods (e.g., RAD). Even in ideal situations where the genome is sampled without bias, the resulting data sets are primarily composed of genetic elements with no known or presumed function, due to the low proportional representation of genes in large genomes (Karam et al., 2015).

Hybridization-based enrichment methods are more expensive, but they come closest to satisfying requirements of co-dominant, highly transferrable genetic markers within species (Neves et al., 2013; Müller et al., 2015; Pavy et al., 2016) and even among species and closely related genera (Weitemier et al., 2014). Because hybridization methods target known loci, there is an expectation for a nearly complete data set because targets differing at a small number of positions are captured with equal efficiency.

Our study highlights two of the challenges in developing target capture probes from poorly characterized genomes; the inclusion of high-copy targets in bait design, and low capture efficiencies. The first challenge, inclusion of high-copy targets in the bait pool (e.g., **Figure 2**), appears to be related to the evolutionary divergence within the genus *Pinus*, and specifically the large genome size variation within *Pinus*. Our choice of loci for bait design was based on loci that yielded high-quality information from a similarly designed target enrichment study of Loblolly Pine (Neves et al., 2013). Loblolly pine and whitebark pine are congeners, but they are members of separate subgenera that differ substantially in genome size; for example, loblolly pine is a member of *Subg. Pinus*, a group with an average genome size of 24.5 Gb, while whitebark pine is a member of *Subg. Strobus*, a group with an average genome size of 29.6 Gb^6^ (Joyner et al., 2001). By choosing enrichment targets from a smaller genomed organism (Loblolly pine, 22 Gb; Neale et al., 2014), it appears that we inadvertently selected elements that have contributed to the genome expansion in Whitebark pine (27 Gb) and other members of *Subg. Strobus*. By comparing the bait pool to the recently released draft genome of the close relative sugar pine (*Subg. Strobus*; genome size = ∼30 Gb), we have identified these specific over-represented sequences as highly repetitive elements (Supplementary Table S3). Simply eliminating the top 100 high-depth repetitive targets from future bait pools would free up an estimated 85% of the total on-target sequencing to low copy targets, and this would produce a more efficient and transferrable assay.

The second challenge, low capture efficiency, is more problematic in our experiment because only 13% of the post-enrichment reads could be aligned to the target transcript sequences. We believe that this low efficiency stems from three different sources that had a large additive effect: (a) probe design from a first-generation transcriptome assembly; (b) enrichment from highly multiplexed reactions with high genomic complexity; and (c) inefficient blocking of adapters. Capture efficiencies from well-curated genomes often range from 50 to 75% (Mamanova et al., 2010), and can approach 90% in the case of small genomes (e.g., the 0.5 Gb genome of *Populus*; Zhou and Holliday, 2012). In contrast, target capture reactions from less-well characterized genomes (Weitemier et al., 2014) and early transcriptome assemblies (Bi et al., 2012) tend to show significantly lower on-target efficiencies, often as low as 20%. In these instances, poor target capture can be a product of structural errors in the original reference sequence (indels; mis-assembled contigs), or the presence of introns within the coding exons used for probe design (Neves et al., 2013). These types of design errors are likely responsible for the 39% of transcript probes that yielded insufficient sequence data for analysis (**Figure 3**). The recent availability of the sugar pine genome offers the most efficient approach for pre-screening markers to eliminate high copy sequence motifs and intron-spanning targets from the bait pool. The second possible source – low efficiency from high multiplexing in capture reactions – has been reported as a complicating factor in at least two studies (Bi et al., 2012; Neves et al., 2013), although it has been ruled out as a complication in other studies (Portik et al., 2015). Our capture experiments included 12 pooled libraries per tube, and this may be too high, particularly given the size and complexity of the target genomes. A lower level of multiplexing seems warranted, although this comes at an increased cost that has to be balanced with the sample sizes needed for population-level studies. The third possible source – inefficient blocking of adapters – is maybe an important and underappreciated factor in low on-target efficiency. A recent study by Portik et al. (in review) identifies blocking probes as the most important factor for on-target sequence efficiency, as the ‘universal’ blocking oligonucleotides used in commercial target enrichment kits show lower performance than newer generation blocking oligonucleotides. These authors suggest that blocking efficiency may be most important in organisms with large genomes; if so, whitebark pine and other pines should provide a rigorous test of the impact of different blocking oligonucleotides on enrichment efficiency.

For assessing germplasm diversity, the ‘ideal’ genetic marker should be highly variable, codominant, provide unbiased coverage across the genome, and show a high degree of transferability between studies (Schoen and Brown, 1993; Bataillon et al., 1996; De Beukelaer et al., 2012). This latter aspect is critical for species like whitebark pine, as data gathered from narrowly focused studies need to contribute to larger questions of global diversity and uniqueness. Data sets derived from targeted enrichment often meet expectations of Hardy-Weinberg and linkage equilibrium (Tennessen et al., 2012; Neves et al., 2013; Müller et al., 2015) and genome evolution, and our data set is no exception, even with our poor on-target capture efficiency and non-uniform bait representation. Because probes target a finite number of pre-selected loci, this method is amenable to developing a standardized vocabulary of genes and polymorphic sites for screening conservation collections. The resulting information can be understood in the richer context of genome organization (e.g., variation at known regions) or presumed gene function. Like restriction-based methods, SNPs can be characterized for substitution type (transition; transversion); however, SNPs revealed by target enrichment permit additional inferences to be made of the impact on translation (non-synonymous, silent, and synonymous substitutions), presumed functional consequences (conservative vs. radical replacements), and relationships to additional information on the timing and tissue-specificity of expression. Target-capture approaches build a rich body of information that bears not only on population-level variation, but also potential gene function and relevance to management.

### Insights into Variation and Differentiation from Genome-Wide Screening

Our population genetic results are consistent with a demographic history of recent expansion from one or more glacial refugia into a spatially restricted subalpine habitat. Our estimate of π (0.26%) is lower than a previous estimate for whitebark derived from 12 individuals at 167 orthologous gene fragments (π = 0.33%; Eckert et al., 2013), and it is lower than comparable values estimated for other alpine and montane conifers (Mosca et al., 2012; Müller et al., 2015). Our estimate of lower diversity is likely a consequence of different locus selection and sample sizes between studies, as well as the true low diversity reflected in the whitebark genome, which are an outcome of a recent glacial bottleneck and/or the continued rarity of the species even during interglacial periods owing to its specialized habitat.

The strong signature of population expansion is evident in our sample and selection of loci, as indicated by the low (≪-1) Tajima’s *D* value. Our estimate is much lower than a prior estimate based on a smaller sample of loci and individuals (*D* = -0.346; Eckert et al., 2013), and estimates of this scale are indicative of a species that has increased effective numbers of individuals by orders of magnitude since the Pleistocene, such as the situation reflected in humans (Tennessen et al., 2012). We observe the greatest genetic diversity in the Central Rocky Mountain Range, Northeastern Oregon, and the Northern Cascades, suggesting that these regions may have harbored the refugia (**Table 1**). Similarly, the allozyme results of Jorgensen and Hamrick (1997) indicated the greatest genetic diversity in the Rocky Mountains, the Sierras, and the Mount Rainier area. Likewise, Richardson et al. (2002) found three geographically distinct mtDNA haplotypes, with two regions of haplotype co-occurrence (Washington Cascades and Idaho Rockies), suggesting these regions were either refugia or zones of secondary contact. We also observe greatly reduced genetic diversity in three trees from the Southern Oregon Cascades, a trend that was previously noted (Jorgensen and Hamrick, 1997; Richardson et al., 2002), indicating that this region was recolonized from refugia by a limited number of individuals, and/or has experienced a more recent bottleneck.

We observe relatively low genetic divergence among populations, as noted previously in this species (Bruederle et al., 2001; Krakowski et al., 2003; Bower and Aitken, 2008). Wind-born pollen is likely the main component of gene flow (Richardson et al., 2002). The most genetically divergent population, the Sierras, was also the most geographically distant from the other samples. Given the isolation and genetic distinctiveness of the Sierras (**Figure 4**), this population may harbor unique variation that may be adaptive in its southern, high-elevation habitat. The genetic similarity among the other populations reflects long-distance gene flow via wind-blown pollen as well as the recent shared ancestry of populations in refugia. Our PC analysis shows that genetic differences among samples are correlated with three intuitive metrics: overall heterozygosity, latitude, and longitude (**Figure 4**). Even accounting for the three low-diversity Oregon Cascades samples (PC 1) and the divergent Sierra samples (PC 2), there was a correlation with both latitude (PC 3) and longitude (PC 4), suggesting that gene flow is spatially constrained and has occurred in both north–south and east–west directions. Several observations underscore the north-south cline in particular: two PCs (2 and 3) were tied to latitude, both showed a stronger correlation than PC 4 did to longitude, and both explained more variance than PC 4. Thus, genetic structure appears to be slightly more substantial in the north–south direction and gene flow may be more common along the east–west axis, perhaps because of prevailing wind directions. In contrast, maternally inherited mtDNA, which is not affected by wind dispersal, shows a stronger east–west divide than a north–south divide (Richardson et al., 2002).

Because our approach involves genotyping thousands of nuclear genes, we can search for the relatively small proportion of genes subject to geographically varying selection (Müller et al., 2015). Although F_ST_ was low for most SNPs, a few SNPs showed very high (>0.8) F_ST_ values (**Table 2**), which may reflect selection pressures that differ among geographic groups. We also identified 12 genes with variants showing a significant correlation with latitude (**Table 3**; Supplementary Figure S1). Although latitude is inexorably confounded with population structure, these markers are intriguing candidates for further validation as underlying local adaptation. In addition, gene comp61071c1 showed an intriguing, though not significant, correlation with frost-free period (**Figure 5**). If these markers represent functionally relevant variation, it could be in the targeted genes themselves or unknown linked genes. The fact that we observed several genes in LD (**Table 4**; Supplementary Table S2), despite an approximate genome-wide density of 1 successfully genotyped transcript per 7 Mb, suggests either that large (>100 kb) haplotype blocks in LD are common, or that coding genes are physically clustered. In either case, a marker showing the signature of selection might be reflecting functional diversity at one of several closely linked genes.

### Potential Applications to Related Pine Species

Whitebark pine is one of eight North American pine species that are allied with *Pinus* subsection *Strobus*, a group commonly referred to as the ‘five-needle pines’ (DeGiorgio et al., 2014). This group includes species that occupy a diversity of habitats, ranging from alpine/high-elevation landscapes (*P. albicaulis; P. flexilis; P. strobiformis; P. ayacahuite*), lower elevation western (*P. lambertiana; P. monticola*) and eastern (*P. strobus*) forests, and narrowly restricted geographic endemics (*P. chiapensis*). These species share much in common, as all show susceptibility to blister rust infection and the deleterious impacts of insect outbreaks, fire, and changing climates (Keane et al., 2011). Similarly, all eight species are the focus of active conservation efforts to preserve existing diversity, some are the focus of active breeding efforts to improve blister rust resistance, and genetic markers are needed to improve the efficiency of these activities. North American five-needle pines are all remarkably similar at the level of orthologous DNA sequences (Syring et al., 2007; Eckert et al., 2013), as they typically show >96% identity at exonic and intronic regions. Divergence at this scale is not prohibitory for target enrichment (Neves et al., 2013), so we predict that these probes will show a high degree of transferability to other five-needle pine species. Adopting a target enrichment approach would enable the development of common genetic resources across these related species. We are currently examining the efficiency of hybridization in North American five-needle pines, as well as more distantly related species of conservation concern, such as foxtail pine (*P. balfouriana*) and bristlecone pines (*P. aristata, P. longaeva*).

## Conclusion

Our goal was to test whether hybridization-based targeted enrichment baits designed from loblolly pine could be adapted to enriching targets from the threatened congener *P. albicaulis* (whitebark pine). Baits were synthesized to cover ∼2 Mbp (0.006% of the genome), and these were hybridized in multiplex pools of genomic DNA and sequenced on the HiSeq 2000. In our study, only a small fraction of the reads (13%) mapped to bait target sequences; also, a small proportion of probes accounted for the vast majority of on-target sequences, indicating that our probe pool included sequences that were homologous to high-copy repeats in the whitebark pine genome. Despite these complications, this effort still provided acceptable coverage for 4,452 loci and >528,800 nucleotide positions, allowed us to identify single nucleotide variants, permitted the direct measurement of heterozogosity, and aided in the identification of regional differentiation and evidence for selection from a test panel of 48 trees. Our results identify trees from the Sierra Range as distinctive within the whitebark pine gene pool, making them high priority targets for germplasm conservation efforts. As shown here and in other studies, hybridization-based target enrichment is a robust method for enriching target genes from the complex ‘giga-genomes’ of conifers, one that provides data that is easily integrated into germplasm management, and has the potential to be extended across related species.

## Author Contributions

JS and RC proposed, funded, and collected data for the research. Both authors contributed to data analysis and wrote significant sections of the paper. JT did much of the data analysis and developed most of the figures, as well as wrote significant portions of the manuscript. TJ did most of the lab work including prepping the library sequences and running the hybridization reactions. She also made contribution to the editing of the manuscript. JW provided a pre-publication copy of the whitebark pine transcriptome which was essential for developing the probe sequences as well as provided consultation on probe selection. CS-D aided in the collection of samples and with DNA extractions. She helped develop **Figure 1**, put together the Literature Cited section, and proofread the manuscript.

## Conflict of Interest Statement

The authors declare that the research was conducted in the absence of any commercial or financial relationships that could be construed as a potential conflict of interest.

## Footnotes

1 http://dendrome.ucdavis.edu/ftp/Genome_Data/genome/pinerefseq/Pita/mito/

2 http://treegenesdb.org/ftp/Transcriptome_Data/transcriptome/Pial/v1.0/

3 https://gc3f.uoregon.edu/

4 http://pinegenome.org/pinerefseq/

5 http://tinyurl.com/ClimateNA

6 http://data.kew.org/cvalues/

## References

[B1] Altschul S. F. Gish W. Miller W. Myers E. W. Lipman D. J. (1990). Basic local alignment search tool. *J. Mol. Biol.* 215 403–410. 10.1016/S0022-2836(05)80360-2 2231712

[B2] Arno S. F. Hoff R. J. (1989). *Silvics of Whitebark Pine (Pinus albicaulis). General Technical Report INT-253.* Ogden, UT: U.S. Department of Agriculture, Forest Service, Intermountain Research Station 14.

[B3] Arnold B. Corbett-Detig R. Hartl D. Bomblies K. (2013). RADseq underestimates diversity and introduces genealogical biases due to nonrandom haplotype sampling. *Mol. Ecol.* 22 3179–3190. 10.1111/mec.12276 23551379

[B4] Aubry C. A. Goheen D. J. Shoal R. Z. Ohlson T. Lorenz T. J. Bower A. D. Mehmel C. Sniezko R.A. (2008). *WhitebarkPine restoration Strategy for the Pacific Northwest Region 2009-2013.* Washington DC, USA: U.S. Department of Agriculture, Forest Service 96 p.

[B5] Bataillon T. M. David J. L. Schoen D. J. (1996). Neutral genetic markers and conservation genetics: simulated germplasm collections. *Genetics* 144 409–417. 8878704 10.1093/genetics/144.1.409 PMC1207513

[B6] Benjamini Y. Hochberg Y. (1995). Controlling the false discovery rate: a practical and powerful approach to multiple testing. *J. R. Statist. Soc. Ser. B.* 57 289–300.

[B7] Bi K. Vanderpool D. Singhal S. Linderoth T. Moritz C. Good J. M. (2012). Transcriptome-based exon capture enables highly cost-effective comparative genomic data collection at moderate evolutionary scales. *BMC Genomics* 13:403 10.1186/1471-2164-13-403 PMC3472323 22900609

[B8] Bockino N. K. Tinker D. B. (2012). Interactions of white pine blister rust and mountain pine beetle in whitebark pine ecosystems in the southern Greater Yellowstone Area. *Nat. Area. J.* 32 31–40. 10.3375/043.032/0105

[B9] Bower A. D. Aitken S. N. (2007). Mating system and inbreeding depression in whitebark pine (*Pinus albicaulis* Engelm.). *Tree Genet. Genomes* 3 379–388. 10.1007/s11295-007-0082-4

[B10] Bower A. D. Aitken S. N. (2008). Ecological genetics and seed transfer guidelines for *Pinus albicaulis* (Pinaceae). *Am. J. Bot.* 95 66–76. 10.3732/ajb.95.1.66 21632316

[B11] Bruederle L. P. Rogers D. L. Krutovskii K. V. Politov D. V. (2001). “Population genetics and evolutionary implications,” in *Whitebark Pine Communities: Ecology and Restoration* eds Tomback D. F. Arno S. F. Keane R. E. (Washington, DC, USA: Island Press) 137–153.

[B12] COSEWIC (2010). “COSEWIC assessment and status report on the Whitebark pine (*Pinus albicaulis*) in Canada,” ed. Committee on the Status of Endangered Wildlife in Canada (Ottawa: COSEWIC) 44.

[B13] Cronn R. Knaus B. J. Liston A. Maughan P. J. Parks M. Syring J. V. (2012). Targeted enrichment strategies for next-generation plant biology. *Am. J. Bot.* 99 291–311. 10.3732/ajb.1100356 22312117

[B14] Davey J. W. Hohenlohe P. A. Etter P. D. Boone J. Q. Catchen J. M. Blaxter M. L. (2011). Genome-wide genetic marker discovery and genotyping using next-generation sequencing. *Nat. Rev. Genet.* 12 499–510. 10.1038/nrg3012 21681211

[B15] De Beukelaer H. Smýkal P. Davenport G. F. Fack V. (2012). Core Hunter II: fast core subset selection based on multiple genetic diversity measures using Mixed Replica search. *BMC Bioinformatics* 13:312 10.1186/1471-2105-13-312 PMC3554476 23174036

[B16] DeGiorgio M. Syring J. Eckert A. J. Liston A. Cronn R. Neale D. B. (2014). An empirical evaluation of two-stage species tree inference strategies using a multilocus dataset from North American pines. *BMC Evol. Biol.* 14:67 10.1186/1471-2148-14-67 PMC4021425 24678701

[B17] Eckert A. J. Bower A. D. Jermstad K. D. Wegrzyn J. L. Knaus B. J. Syring J. V. (2013). Multilocus analyses reveal little evidence for lineage-wide adaptive evolution within major clades of soft pines (*Pinus* subgenus Strobus). *Mol. Ecol.* 22 5635–5650. 10.1111/mec.12514 24134614

[B18] Ellison A. M. Bank M. S. Clinton B. D. Colburn E. A. Elliott K. Ford C. R. (2005). Loss of foundation species: consequences for the structure and dynamics of forested ecosystems. *Front. Ecol. Environ.* 3 479–486. 10.1890/1540-9295(2005)003

[B19] Farnes P. E. (1990). “SNOTEL and snow course data: describing the hydrology of whitebark pine ecosystems,” in *Proceedings of a Symposium on Whitebark Pine Ecosystems: Ecology and Management of a High-Mountain Resource, General Technical Report INT-GTR-270* eds Schmidt W. C. McDonald K. J. (Ogden, UT: U.S. Department of Agriculture, Forest Service) 29–31.

[B20] Furnier G. R. Knowles P. Clyde M. A. Dancik B. P. (1987). Effects of avian seed dispersal on the genetic structure of whitebark pine populations. *Evolution* 3 607–612. 10.2307/2409262 28563807

[B21] Gautier M. Gharbi K. Cezard T. Foucaud J. Kerdelhué C. Pudlo P. (2013). The effect of RAD allele dropout on the estimation of genetic variation within and between populations. *Mol. Ecol.* 22 3165–3178. 10.1111/mec.12089 23110526

[B22] Gibson K. Skov K. Kegley S. Jorgensen C. Smith S. Witcosky J. (2008). *Mountain Pine Beetle Impacts in High-Elevation Five-Needle Pines: Current Trends and Challenges* Report R1-08-020 Missoula, MT: U.S. Department of Agriculture, Forest Service, Northern Region Forest Health Protection 40.

[B23] Gnirke A. Melnikov A. Maguire J. Rogov P. LeProust E. M. Brockman W. (2009). Solution hybrid selection with ultra-long oligonucleotides for massively parallel targeted sequencing. *Nat. Biotechnol.* 27 182–189. 10.1038/nbt.1523 19182786 PMC2663421

[B24] Hamann A. Wang T. Spittlehouse D. L. Murdock T. Q. (2013). A comprehensive, high-resolution database of historical and projected climate surfaces for western North America. *Bull. Am. Meteorol. Soc.* 94 1307–1309. 10.1175/BAMS-D-12-00145.1

[B25] Hebert F. O. Renaut S. Bernatchez L. (2013). Targeted sequence capture and resequencing implies a predominant role of regulatory regions in the divergence of a sympatric lake whitefish species pair (*Coregonus clupeaformis*). *Mol. Ecol.* 22 4896–4914. 10.1111/mec.12447 23962219

[B26] IUCN (2015). *The IUCN Red List of Threatened Species*. *Version 2015–2014.* Available at: http://www.iucnredlist.org

[B27] Jones M. R. Good J. M. (2016). Targeted capture in evolutionary and ecological genomics. *Mol. Ecol.* 25 185–202. 10.1111/mec.13304 26137993 PMC4823023

[B28] Jorgensen S. M. Hamrick J. L. (1997). Biogeography and population genetics of whitebark pine, *Pinus albicaulis*. *Can. J. For. Res.* 27 1574–1585. 10.1139/x97-118

[B29] Joyner K. L. Wang X. R. Johnston J. S. Price H. J. Williams C. G. (2001). DNA content for Asian pines parallels new world relatives. *Can. J. Bot.* 79 192–196. 10.1139/b00-151

[B30] Karam M. J. Lefèvre F. Dagher-Kharrat M. B. Pinosio S. Vendramin G. (2015). Genomic exploration and molecular marker development in a large and complex conifer genome using RADseq and mRNAseq. *Mol. Ecol. Resour.* 15 601–612. 10.1111/1755-0998.12329 25224750

[B31] Keane R. E. Arno S. F. Brown J. K. Tomback D. F. (1990). Modelling stand dynamics in whitebark pine (*Pinus albicaulis*) forests. *Ecol. Model.* 51 73–95. 10.1016/0304-3800(90)90059-P

[B32] Keane R. E. Tomback D. F. Murray M. P. Smith Cyndi M. (eds) (2011). “The future of high-elevation, five-needle white pines in Western North America,” in *Proceedings of the High Five Symposium RMRS-P-63* (Fort Collins, CO: U.S. Department of Agriculture, Forest Service, Rocky Mountain Research Station) 376.

[B33] Kendall K. C. Keane R. E. (2001). “Whitebark pine decline: infection, mortality, and population trends,” in *Whitebark Pine Communities: Ecology and Restoration* eds Tomback D. F. Arno S. F. Keane R. E. (Washington, DC: Island Press) 221–242.

[B34] Kent W. J. (2002). BLAT—the BLAST-like alignment tool. *Genome Res.* 12 656–664. 10.1101/gr.229202 11932250 PMC187518

[B35] Krakowski J. Aitken S. N. El-Kassaby Y. A. (2003). Inbreeding and conservation genetics in whitebark pine. *Conserv. Genet.* 4 581–593. 10.1023/A:1025667700479

[B36] Li H. Durbin R. (2009). Fast and accurate short read alignment with Burrows–Wheeler transform. *Bioinformatics* 25 1754–1760. 10.1093/bioinformatics/btp324 19451168 PMC2705234

[B37] Li H. Handsaker B. Wysoker A. Fennell T. Ruan J. Homer N. (2009). The sequence alignment/map format and SAMtools. *Bioinformatics* 25 2078–2079. 10.1093/bioinformatics/btp352 19505943 PMC2723002

[B38] Little E. L. (1971). *Atlas of the United States Trees: Conifers and Important Hardwoods* Vol. 1 Washington, DC: Department of Agriculture, Miscellaneous Publication 1146

[B39] Mahalovich M. F. Hipkins V. D. (2011). “Molecular genetic variation in whitebark pine (*Pinus albicaulis* Engelm.) in the Inland West,” in *Proceedings of the High Five Symposium RMRS-P-63 The Future of High-Elevation Five-Needle White Pines in Western North America. Missoula, MT* eds Keane R. E. Tomback D. F. Murray M. P. Smith C. M (Fort Collins, CO: U.S. Department of Agriculture, Forest Service, Rocky Mountain Research Station) 118–132.

[B40] Mamanova L. Coffey A. J. Scott C. E. Kozarewa I. Turner E. H. Kumar A. (2010). Target-enrichment strategies for next-generation sequencing. *Nat. Methods* 7 111–118. 10.1038/nmeth.1419 20111037

[B41] Mattson D. Kendall K. Reinhart D. (2001). “Whitebark pine, grizzly bears, and red squirrels,” in *Whitebark Pine Communities: Ecology and Restoration* eds Tomback D. F. Arno S. F. Keane R. E. (Washington, DC: Island Press) 121–136.

[B42] Millar C. I. Westfall R. D. Delany D. L. Bokach M. J. Flint A. L. Flint L. E. (2012). Forest mortality in high-elevation whitebark pine (*Pinus albicaulis*) forests of eastern California, USA; influence of environmental context, bark beetles, climatic water deficit, and warming. *Can. J. For. Res.* 42 749–765. 10.1139/x2012-031

[B43] Mills L. S. Soulé M. E. Doak D. F. (1993). The keystone-species concept in ecology and conservation. *BioScience* 43 219–224. 10.2307/1312122

[B44] Morse A. M. Peterson D. G. Islam-Faridi M. N. Smith K. E. Magbanua Z. Garcia S. A. (2009). Evolution of genome size and complexity in *Pinus*. *PLoS ONE* 4:e4332 10.1371/journal.pone.0004332 PMC2633040 19194510

[B45] Mosca E. Eckert A. J. Liechty J. D. Wegrzyn J. L. La Porta N. Vendramin G. G. (2012). Contrasting patterns of nucleotide diversity for four conifers of Alpine European forests. *Evol. Appl.* 5 762–775. 10.1111/j.1752-4571.2012.00256.x 23144662 PMC3492901

[B46] Müller T. Freund F. Wildhagen H. Schmid K. J. (2015). Targeted re-sequencing of five Douglas-fir provenances reveals population structure and putative target genes of positive selection. *Tree Genet. Genomes* 11 1–17. 10.006//s11295-014-0816-z

[B47] Narum S. R. Buerkle C. A. Davey J. W. Miller M. R. Hohenlohe P. A. (2013). Genotyping-by-sequencing in ecological and conservation genomics. *Mol. Ecol.* 22 2841–2847. 10.1111/mec.12350 23711105 PMC3935057

[B48] Neale D. B. Wegrzyn J. L. Stevens K. A. Zimin A. V. Puiu D. Crepeau M. W. (2014). Decoding the massive genome of loblolly pine using haploid DNA and novel assembly strategies. *Genome Biol.* 15:R59 10.1186/gb-2014-15-3-r59 PMC4053751 24647006

[B49] Neves L. G. Davis J. M. Barbazuk W. B. Kirst M. (2013). Whole-exome targeted sequencing of the uncharacterized pine genome. *Plant J.* 75 146–156. 10.1111/tpj.12193 23551702

[B50] Pavy N. Gagnon F. Deschênes A. Boyle B. Beaulieu J. Bousquet J. (2016). Development of highly reliable in silico SNP resource and genotyping assay from exome capture and sequencing: an example from black spruce (*Picea mariana*). *Mol. Ecol. Resour.* 16 588–598. 10.1111/1755-0998.12468 26391535

[B51] Petit R. J. Hampe A. (2006). Some evolutionary consequences of being a tree. *Annu. Rev. Ecol. Evol. Syst.* 347 187–214. 10.2307/annurev.ecolsys.37.091305.110215

[B52] Portik D. M. Smith L. L. Bi K. (2015). An evaluation of transcriptome-based exon capture for frog phylogenomics across multiple scales of divergence (Class: Amphibia, Order: Anura). *BioRxiv* 10.1101/031468 27241806

[B53] Price R. Liston A. Strauss S. (2000). “Phylogeny and systematics of *Pinus*,” in *Ecology and Biogeography of Pinus* ed. Richardson D. (Cambridge: Cambridge University Press) 49–68.

[B54] Richardson B. A. Brunsfeld S. Klopfenstein N. B. (2002). DNA from bird-dispersed seed and wind-disseminated pollen provides insights into postglacial colonization and population genetic structure of whitebark pine (*Pinus albicaulis*). *Mol. Ecol.* 11 215–227. 10.1046/j.1365-294X.2002.01435.x 11856423

[B55] Rogers D. L. Millar C. I. Westfall R. D. (1999). Fine-scale genetic structure of whitebark pine (*Pinus albicaulis*): associations with watershed and growth form. *Evolution* 53 74–90. 10.2307/2640921 28565192

[B56] Schoen D. J. Brown A. (1993). Conservation of allelic richness in wild crop relatives is aided by assessment of genetic markers. *Proc. Natl. Acad. Sci. U.S.A.* 90 10623–10627. 10.1073/pnas.90.22.10623 8248153 PMC47829

[B57] Shafer A. B. A. Cullingham C. I. Cote S. D. Coltman D. W. (2010). Of glaciers and refugia: a decade of study sheds new light on phylogeography of northwestern North America. *Mol. Ecol.* 19 4589–4621. 10.1111/j.1365-294X.2010.04828.x 20849561

[B58] Shoal R. Aubry C. (2004). *The Status of Whitebark Pine on Four National Forests in Washington State.* Olympia, WA: US Department of Agriculture, Forest Service, Pacific Northwest Region, Olympic National Forest.

[B59] Smit A. Hubley R. Green P. (2014). *RepeatMasker Open-4.0* 2013–2015. Available at: http://www.repeatmasker.org

[B60] Stajich J. E. Hahn M. W. (2005). Disentangling the effects of demography and selection in human history. *Mol. Biol. Evol.* 22 63–73. 10.1093/molbev/msh252 15356276

[B61] Syring J. Farrell K. Businský R. Cronn R. Liston A. (2007). Widespread genealogical nonmonophyly in species of *Pinus* subgenus Strobus. *Syst. Biol.* 56 163–181. 10.1080/10635150701258787 17454973

[B62] Tennessen J. A. Bigham A. W. O’Connor T. D. Fu W. Kenny E. E. Gravel S. (2012). Evolution and functional impact of rare coding variation from deep sequencing of human exomes. *Science* 337 64–69. 10.1126/science.1219240 22604720 PMC3708544

[B63] Tennessen J. A. Govindarajulu R. Liston A. Ashman T.-L. (2013). Targeted sequence capture provides insight into genome structure and genetics of male sterility in a gynodioecious diploid strawberry, Fragaria vesca ssp. bracteata (Rosaceae). *G3* 3 1341–1351. 10.1534/g3.113.006288 23749450 PMC3737174

[B64] Tomback D. F. (2005). “The impact of seed dispersal by Clark’s nutcracker on whitebark pine: multi-scale perspective on a high mountain mutualism,” in *Mountain Ecosystems: Studies in Treeline Ecology* eds Broll G. Kepline B. (Berlin: Springer) 181–201.

[B65] Tomback D. F. Arno S. F. Keane R. E. (2001). “The compelling case for management intervention,” in *Whitebark Pine Communities: Ecology and Restoration* eds Tomback D. F. Arno S. F. Keane R. E. (Washington, DC: Island Press) 3–28.

[B66] US Fish and Wildlife Service (2011). “*Endangered and Threatened Wildlife and Plants; 12-Month Finding on A Petition to List Pinus Albicaulis As Endangered Or Threatened With Critical Habitat*” Federal Register Docket ID: FWS-R6-ES-2010-0047 Available at: www.fdderalregister.gov/articles/2010/12/14/2010-30573/endangered-and-threatend-wildlife-and-plants 12-months-finding-on-a-petiton-to-list-the-north

[B67] Warwell M. V. Rehfeldt G. E. Crookston N. L. (2007). “Modeling contemporary climate profiles of whitebark pine (*Pinus albicaulis*) and predicting responses to global warming,” in *Proceedings of the Conference Whitebark Pine: A Pacific Coast Perspective* (Pacific Northwest Region, NA: U.S. Department of Agriculture, Forest Service) 139–142.

[B68] Weitemier K. Straub S. C. Cronn R. C. Fishbein M. Schmickl R. McDonnell A. (2014). Hyb-Seq: combining target enrichment and genome skimming for plant phylogenomics. *Appl. Plant Sci.* 2:1400042 10.3732/apps.1400042 PMC4162667 25225629

[B69] Wilson B. (2007). *Status of the Whitebark Pine (Pinus albicaulis) in Alberta.* Wildlife Status Report No. 63. Edmonton, AB: Alberta Sustainable Resource Development and Alberta Conservation Association 22 10.5962/bhl.title.114087

[B70] Zhou L. Holliday J. A. (2012). Targeted enrichment of the black cottonwood (*Populus trichocarpa*) gene space using sequence capture. *BMC Genomics* 13:703 10.1186/1471-2164-13-703 PMC3542275 23241106

