# Supplementary material for: Targeted Capture Sequencing in Whitebark Pine Reveals Range-Wide Demographic and Adaptive Patterns Despite Challenges of a Large, Repetitive Genome
Source: Front Plant Sci. 2016 Apr 21;7:484. doi: 10.3389/fpls.2016.00484 (PMC4838605; doi:10.3389/fpls.2016.00484)
Supplement: Supplementary file 1 [file Data_Sheet_1.DOCX]

Supplementary Material

**Targeted capture sequencing in whitebark pine reveals range-wide demographic and adaptive patterns despite challenges of a large, repetitive genome**

John V. Syring*, Jacob A. Tennessen, Tara N. Jennings, Jill Wegrzyn, Camille Scelfo-Dalbey, Richard Cronn

*** Correspondence:** John Syring: [jsyring@linfield.edu](mailto:jsrying@linfield.edu)

**Supplementary Figure 1**. Flowchart showing steps used in development, isolation, and analysis of targeted genomic regions in whitebark pine, *Pinus albicaulis*.

**
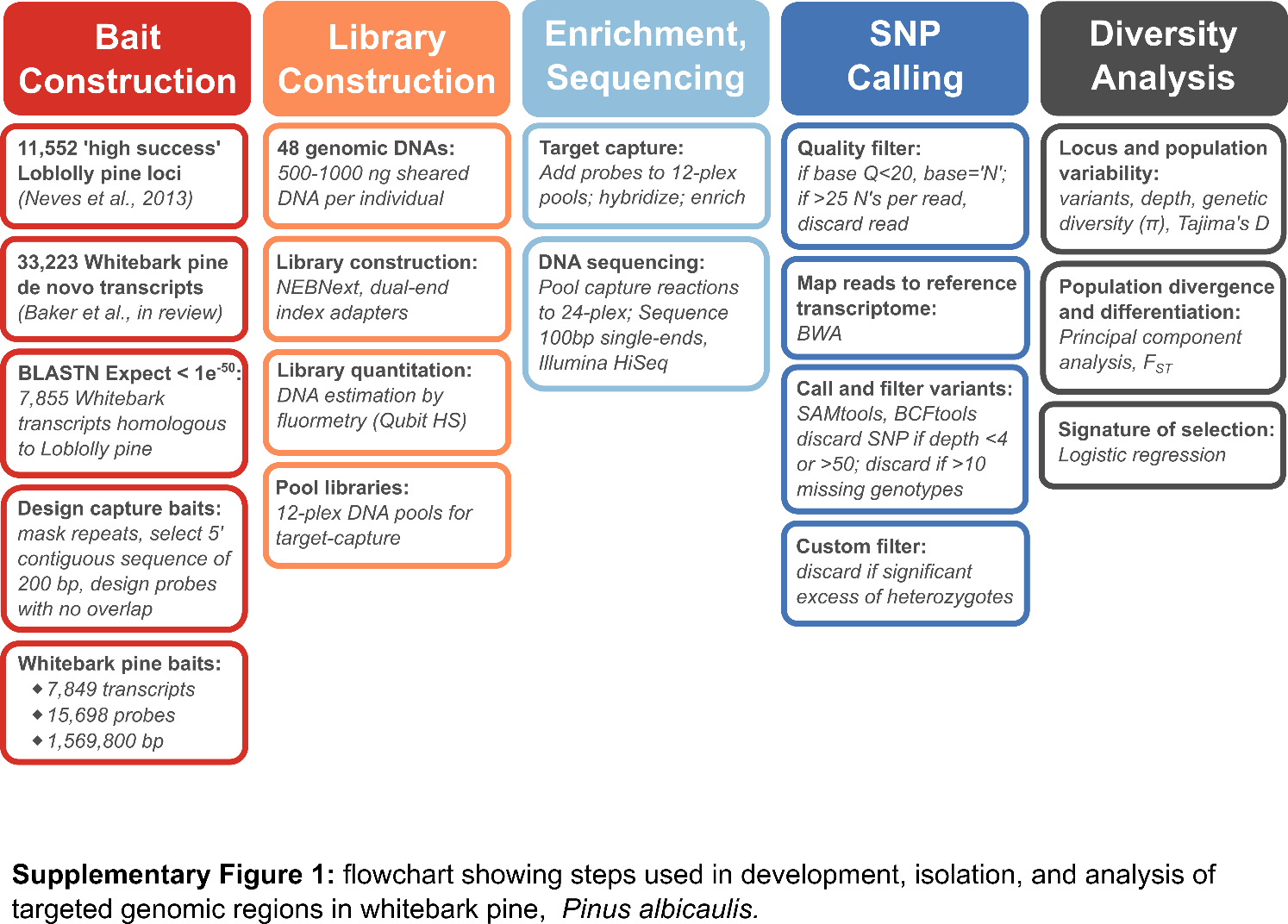
**

**Supplementary Figure 2**. Distribution of comp70653c0_145, the variant showing the most significant correlation with latitude, across the sampled geographical range of *P. albicaulis*. Major allele homozygotes = squares, heterozygotes = circles in squares, minor allele homozygotes = shaded circles. Small symbols = missing data (presumed major allele homozygotes). Colors indicate geographical sub-sample.

**Supplementary Table 1.** Collection information and source material used for DNA for Whitebark Pine samples.

| **No.** | **Longitude (W)** | **Latitude (N)** | **Administrative Unit, State/Province, Country** |  | **Tissue Source^1^** |
| --- | --- | --- | --- | --- | --- |
| 1 | 119°56'25" | 38°51'28" | Lake Tahoe Basin Management Unit, California, USA |  | E |
| 2 | 109°31'57" | 45°00'17" | Absaroka-Beartooth Wilderness, Gallatin National Forest, Montana, USA |  | E |
| 3 | 120°00'41" | 38°40'09" | Eldorado National Forest, California, USA |  | E |
| 4 | 114°15'45" | 49°21'22" | Table Mountain Regional Park, Alberta, CAN |  | E |
| 5 | 119°36'17" | 53°42'25" | Willmore Wilderness Park, Alberta, CAN |  | E |
| 6 | 114°03'08" | 45°42'05" | Bitterroot National Forest, Montana, USA |  | N |
| 7 | 114°12'58" | 46°30'07" | Bitterroot National Forest, Montana, USA |  | N |
| 8 | 116°41'10" | 48°56'31" | Panhandle National Forests, Idaho, USA |  | N |
| 9 | 120°06'34" | 53°20'34" | Sunbeam Creek Ecological Reserve, British Columbia, CAN |  | N |
| 10 | 117°04'40" | 51°16'29" | Kicking Horse Mountain Resort, British Columbia, CAN |  | N |
| 11 | 116°29'55" | 51°43'02" | Banff National Park, British Columbia, CAN |  | N |
| 12 | 116°04'52" | 50°12'54" | Whitetail lake Recreation Site, British Columbia, CAN |  | N |
| 13 | 118°41'37" | 37°28'33" | Inyo National Forest, California, USA |  | N |
| 14 | 118°46'03" | 37°24'59" | Inyo National Forest, California, USA |  | N |
| 15 | 119°14'25" | 38°04'35" | Humboldt-Toiyabe National Forest, California, USA |  | E |
| 16 | 118°45'08" | 37°26'30" | Inyo National Forest, California, USA |  | E |
| 17 | 115°58'35'' | 45°00'50" | Payette National Forest, Idaho, USA |  | N |
| 18 | 119°55'02" | 39°20'38" | Humboldt-Toiyabe National Forest, Nevada, USA |  | E |
| 19 | 115°29'19" | 41°47'42" | Humboldt-Toiyabe National Forest, Nevada, USA |  | E |
| 20 | 121°39'32" | 51°05'21" | Lime Ridge, Marble Range, British Columbia, CAN |  | E |
| 21 | 109°56'06" | 45°01'09" | Custer Gallatin National Forest, Montana, USA |  | N |
| 22 | 125°35'21" | 55°01'02" | Mt. Sidney Williams, Middle River Range, British Columbia, CAN |  | E |
| 23 | 125°49'00" | 52°32'00" | Heckman Pass, Rainbow Range, British Columbia, CAN |  | E |
| 24 | 120°55'00" | 49°43'00" | Mt. Thynne, Okanagan Range, British Columbia, CAN |  | E |
| 25 | 110°56'59" | 43°30'19" | Bridger-Teton National Forest, Wyoming, USA |  | N |
| 26 | 121°71'15" | 45°33'13" | Mt. Hood National Forest, Oregon, USA |  | N |
| 27 | 121°52'16" | 43°49'08" | Deschutes National Forest, Oregon, USA |  | N |
| 28 | 121°40'19" | 43°58'59" | Deschutes National Forest, Oregon, USA |  | N |
| 29 | 121°11'28" | 43°41'38" | Deschutes National Forest, Oregon, USA |  | N |
| 30 | 120°14'06" | 42°14'10" | Freemont National Forest, Oregon, USA |  | N |
| 31 | 121°31'19" | 46°09'50" | Gifford Pinchot National Forest, Washington, USA |  | N |
| 32 | 123°08'02" | 47°49'12" | Olympic National Forest, Washington, USA |  | N |
| 33 | 122°43'12" | 42°05'10" | Rogue River National Forest, Oregon, USA |  | N |
| 34 | 122°06'11" | 43°22'44" | Umpqua National Forest, Oregon, USA |  | N |
| 35 | 118°14'38" | 44°57'14" | Wallowa-Whitman National Forest, Oregon, USA |  | N |
| 36 | 118°02'24" | 44°46'23" | Wallowa-Whitman National Forest, Oregon, USA |  | N |
| 37 | 120°19'52" | 48°06'25" | Wenatchee National Forest, Washington, USA |  | N |
| 38 | 120°20'13" | 47°53'17" | Wenatchee National Forest, Washington, USA |  | N |
| 39 | 120°26'02" | 47°16'44" | Wenatchee National Forest, Washington, USA |  | N |
| 40 | 118°28'30" | 48°39'32" | Colville National Forest, Washington, USA |  | N |
| 41 | 124°07'19" | 51°29'28" | Chilcotin Range, British Columbia, CAN |  | N |
| 42 | 122°54'00" | 50°03'36" | Blackcomb Mountain, Fitzsimmons Range, British Columbia, CAN |  | N |
| 43 | 123°32'24" | 51°15'47" | Chilcotin Range, British Columbia, CAN |  | N |
| 44 | 121°39'04" | 46°54'54" | Mt. Rainier National Park, Washington, USA |  | N |
| 45 | 122°09'58" | 42°55'26" | Crater Lake National Park, Oregon, USA |  | N |
| 46 | 115°04'05" | 44°10'12" | Sawtooth Wilderness, Sawtooth National Forest, Idaho, USA |  | N |
| 47 | 114°10'52" | 43°47'20" | Sawtooth National Forest, Idaho, USA |  | N |
| 48 | 114°42'50" | 43°52'34" | Sawtooth National Forest, Idaho, USA |  | N |

**^1^ Tissue Source:** source of DNA for sequencing was embryo (E) or needle (N) tissue

**Supplementary Table 2.** Blocks of high linkage disequilibrium (LD), in which genes show LD of at least 0.15 with at least one other gene in the block.

| **Count** | **Genes** |
| --- | --- |
| 9 | comp67636c0,comp58128c0,comp35949c0,comp72449c0,comp60766c0,comp61270c0,comp68485c0,comp64341c1,comp68536c0 |
| 5 | comp68325c0,comp65744c0,comp64425c0,comp78339c0,comp64268c0 |
| 4 | comp62424c0,comp65919c0,comp64896c0,comp56342c0 |
| 4 | comp70822c0,comp68040c0,comp68019c0,comp61838c0 |
| 3 | comp69667c0,comp65771c1,comp69440c0 |
| 3 | comp44144c1,comp45755c0,comp63251c0 |
| 3 | comp65180c0,comp62519c1,comp67420c0 |
| 3 | comp55192c0,comp63411c0,comp70660c1 |
| 3 | comp70381c0,comp64546c0,comp62234c0 |
| 3 | comp63248c0,comp71610c0,comp67467c0 |
| 2 | comp69497c0,comp67456c0 |
| 2 | comp58055c0,comp69072c0 |
| 2 | comp60120c0,comp64794c0 |
| 2 | comp48027c1,comp56441c0 |
| 2 | comp51362c0,comp59591c0 |
| 2 | comp45176c0,comp53159c0 |
| 2 | comp63753c0,comp55596c0 |
| 2 | comp58431c0,comp66706c0 |
| 2 | comp55082c0,comp72404c0 |
| 2 | comp57186c0,comp69449c0 |
| 2 | comp72215c0,comp60587c0 |
| 2 | comp67120c0,comp67530c0 |
| 2 | comp46404c0,comp67257c0 |
| 2 | comp69692c0,comp36375c0 |
| 2 | comp70150c2,comp53319c0 |
| 2 | comp75608c0,comp50976c0 |
| 2 | comp54451c0,comp67628c0 |
| 2 | comp57190c0,comp68172c0 |
| 2 | comp72315c0,comp69001c0 |
| 2 | comp56283c0,comp67583c0 |
| 2 | comp64752c1,comp54325c0 |
| 2 | comp61069c0,comp65299c0 |
| 2 | comp62795c0,comp65335c1 |
| 2 | comp62187c0,comp66623c0 |
| 2 | comp69543c0,comp70311c0 |
| 2 | comp74970c0,comp43981c0 |
| 2 | comp71398c1,comp58414c0 |
| 2 | comp62764c0,comp63675c0 |
| 2 | comp60764c1,comp71383c0 |
| 2 | comp48272c1,comp48272c0 |
| 2 | comp70147c0,comp68433c0 |
| 2 | comp59505c0,comp68421c0 |
| 2 | comp58820c0,comp69300c0 |

**Supplementary Table 3.** Extent of homologous sequence in sugar pine to each whitebark pine transcript. Number of reads aligning to each *P. albicaulis* transcript (across all 48 samples), and number of identified regions showing homology to hybridization probes in the *P. lambertiana* genome (across two nonoverlapping probe sequences per transcript). The table is organized from the highest number of homologous regions to the lowest number of homologous regions in *P. lambertiana*.

| **Transcript** | ***P. albicaulis* reads aligning** | ***P. lambertiana* homologous regions** |
| --- | --- | --- |
| comp72251c0 | 2026428 | 317327 |
| comp70954c0 | 1068932 | 292899 |
| comp54346c0 | 2046949 | 89144 |
| comp837686c0 | 129218 | 79844 |
| comp4872c0 | 446487 | 74386 |
| comp64532c0 | 141525 | 64100 |
| comp72756c0 | 2188363 | 56560 |
| comp72728c0 | 394474 | 46665 |
| comp72686c0 | 1224 | 45242 |
| comp72751c0 | 2113754 | 44067 |
| comp71797c0 | 2326433 | 34078 |
| comp72585c0 | 5338262 | 29402 |
| comp70766c0 | 490233 | 28551 |
| comp72660c0 | 5609940 | 25810 |
| comp55353c0 | 15062 | 24797 |
| comp55840c0 | 9301 | 21136 |
| comp72712c0 | 2327599 | 20941 |
| comp71622c1 | 761729 | 17652 |
| comp70595c0 | 498400 | 16532 |
| comp64874c0 | 2539042 | 12639 |
| comp72613c0 | 1116132 | 11661 |
| comp52626c2 | 567321 | 11148 |
| comp65692c1 | 11818 | 10923 |
| comp52626c0 | 1686960 | 10555 |
| comp69341c1 | 266089 | 9707 |
| comp68817c0 | 6854 | 9446 |
| comp589568c0 | 5157 | 9435 |
| comp72747c0 | 560777 | 8544 |
| comp19389c0 | 30788 | 8035 |
| comp71858c0 | 33071 | 7167 |
| comp62392c0 | 445791 | 6900 |
| comp61599c0 | 7683 | 6413 |
| comp68357c0 | 55296 | 5938 |
| comp72059c0 | 51835 | 5833 |
| comp40693c0 | 1729 | 5512 |
| comp5745c0 | 48 | 5020 |
| comp65844c0 | 538 | 4791 |
| comp67971c0 | 26292 | 4389 |
| comp72740c0 | 53612 | 3916 |
| comp68368c0 | 89 | 3736 |
| comp83021c0 | 660 | 3435 |
| comp55639c0 | 2455 | 3328 |
| comp57485c0 | 208739 | 3321 |
| comp61583c0 | 319 | 3102 |
| comp66460c0 | 532 | 3032 |
| comp59679c0 | 283 | 3021 |
| comp44478c0 | 35181 | 2922 |
| comp60491c3 | 55347 | 2843 |
| comp71461c0 | 555 | 2422 |
| comp68151c0 | 129770 | 2421 |
| comp53019c0 | 668 | 2381 |
| comp68547c0 | 1528 | 2101 |
| comp67645c0 | 5794 | 2022 |
| comp56291c0 | 1698226 | 1922 |
| comp68316c0 | 670 | 1524 |
| comp56293c0 | 6594 | 1485 |
| comp39136c0 | 405 | 1471 |
| comp51182c0 | 2039 | 1455 |
| comp63649c1 | 94844 | 1322 |
| comp57701c1 | 392 | 1258 |
| comp59845c0 | 205137 | 1250 |
| comp65205c1 | 9093 | 1241 |
| comp698c0 | 8562 | 1126 |
| comp65859c0 | 1586 | 1076 |
| comp72091c0 | 68395 | 1073 |
| comp59432c0 | 61502 | 1041 |
| comp688149c0 | 2469 | 1016 |
| comp68321c0 | 127491 | 980 |
| comp68653c2 | 17 | 933 |
| comp72432c1 | 6620 | 899 |
| comp53042c0 | 270225 | 871 |
| comp45061c0 | 114298 | 845 |
| comp53911c0 | 4184 | 826 |
| comp63557c0 | 14529 | 777 |
| comp67821c1 | 878 | 777 |
| comp59285c0 | 291 | 770 |
| comp1832236c0 | 22365 | 758 |
| comp52031c0 | 115 | 753 |
| comp55302c0 | 1617 | 675 |
| comp70531c0 | 1304 | 641 |
| comp24601c0 | 106612 | 633 |
| comp1947c0 | 1956 | 602 |
| comp67778c0 | 113868 | 600 |
| comp63852c0 | 20204 | 523 |
| comp72421c0 | 503 | 523 |
| comp66870c0 | 7059 | 519 |
| comp62781c0 | 242 | 502 |
| comp71772c0 | 48186 | 499 |
| comp72682c0 | 764 | 490 |
| comp53492c0 | 447 | 486 |
| comp59851c0 | 8840 | 485 |
| comp63079c0 | 1486 | 478 |
| comp50957c0 | 815 | 455 |
| comp534556c0 | 635 | 451 |
| comp46533c0 | 535 | 447 |
| comp77131c0 | 638 | 410 |
| comp67097c0 | 79603 | 409 |
| comp53371c1 | 4779 | 408 |
| comp71461c2 | 575 | 399 |
| comp71862c0 | 795 | 398 |
| comp43265c0 | 3859 | 388 |
| comp51869c0 | 438 | 385 |
| comp63120c0 | 653 | 372 |
| comp58182c0 | 258 | 369 |
| comp64684c0 | 36992 | 362 |
| comp65902c1 | 25312 | 348 |
| comp71410c1 | 11421 | 348 |
| comp70832c0 | 4820 | 323 |
| comp1176217c0 | 1395 | 309 |
| comp52023c0 | 92 | 302 |
| comp1144403c0 | 95263 | 297 |
| comp72175c0 | 194 | 286 |
| comp2969048c0 | 14277 | 281 |
| comp51559c0 | 16214 | 269 |
| comp62165c0 | 119 | 268 |
| comp69749c0 | 1044 | 268 |
| comp61964c0 | 333 | 266 |
| comp55590c0 | 60268 | 258 |
| comp69833c0 | 13180 | 258 |
| comp54041c0 | 12413 | 255 |
| comp69338c0 | 14154 | 254 |
| comp62734c0 | 1605 | 250 |
| comp71830c0 | 1417 | 250 |
| comp68569c0 | 16772 | 248 |
| comp70018c0 | 1193 | 241 |
| comp60101c0 | 939 | 237 |
| comp72631c0 | 4057 | 237 |
| comp72037c0 | 9070 | 235 |
| comp58439c0 | 652 | 227 |
| comp64907c0 | 27706 | 225 |
| comp68209c0 | 24018 | 225 |
| comp61532c0 | 12868 | 223 |
| comp70081c0 | 979 | 219 |
| comp64045c0 | 1244 | 218 |
| comp66921c0 | 49577 | 210 |
| comp72477c0 | 1475 | 209 |
| comp57246c0 | 2165 | 208 |
| comp67332c0 | 614 | 208 |
| comp69129c0 | 356 | 201 |
| comp72744c0 | 38785 | 201 |
| comp64851c0 | 35338 | 198 |
| comp71724c0 | 1499 | 197 |
| comp66125c0 | 577 | 194 |
| comp68893c0 | 20566 | 194 |
| comp35490c0 | 21641 | 191 |
| comp57735c0 | 134 | 186 |
| comp67447c0 | 184 | 184 |
| comp65131c0 | 49113 | 179 |
| comp27485c0 | 20699 | 177 |
| comp68133c3 | 1545 | 171 |
| comp59137c0 | 12924 | 169 |
| comp64128c0 | 5905 | 167 |
| comp62050c0 | 667 | 166 |
| comp60575c0 | 870 | 165 |
| comp70067c0 | 766 | 165 |
| comp73793c0 | 7979 | 161 |
| comp72655c1 | 11838 | 160 |
| comp66736c0 | 3244 | 158 |
| comp58426c1 | 1251 | 157 |
| comp69546c0 | 259 | 156 |
| comp42718c0 | 644 | 154 |
| comp72697c0 | 41140 | 153 |
| comp52357c0 | 43384 | 152 |
| comp68553c0 | 1243 | 152 |
| comp71366c0 | 2117 | 150 |
| comp60946c0 | 1455 | 149 |
| comp2241670c0 | 83703 | 146 |
| comp44262c0 | 56 | 141 |
| comp65133c1 | 2058 | 140 |
| comp67765c0 | 414 | 136 |
| comp72737c0 | 985 | 134 |
| comp72563c0 | 346 | 132 |
| comp68304c0 | 1904 | 130 |
| comp71531c1 | 17973 | 130 |
| comp71593c0 | 14430 | 129 |
| comp44000c0 | 1440 | 127 |
| comp63333c1 | 7855 | 127 |
| comp51162c0 | 579 | 121 |
| comp52039c0 | 901 | 121 |
| comp72182c0 | 1059 | 121 |
| comp68765c0 | 447 | 120 |
| comp70351c0 | 291 | 120 |
| comp62216c0 | 802 | 119 |
| comp63091c0 | 88 | 117 |
| comp66997c0 | 349 | 117 |
| comp70435c0 | 458 | 117 |
| comp63288c1 | 1653 | 116 |
| comp67049c0 | 19349 | 116 |
| comp71556c1 | 442 | 113 |
| comp55305c0 | 5526 | 111 |
| comp68537c0 | 20798 | 111 |
| comp72688c0 | 7013 | 111 |
| comp57687c1 | 1287 | 110 |
| comp54335c0 | 1145 | 109 |
| comp59626c0 | 801 | 109 |
| comp72727c0 | 25021 | 109 |
| comp69438c0 | 30017 | 108 |
| comp70127c0 | 23975 | 108 |
| comp69695c1 | 1868 | 107 |
| comp62462c0 | 77 | 105 |
| comp70644c0 | 26547 | 105 |
| comp71429c0 | 699 | 105 |
| comp72082c0 | 9497 | 105 |
| comp72274c0 | 300 | 104 |
| comp45236c0 | 28373 | 102 |
| comp54558c0 | 2105 | 101 |
| comp55734c0 | 11859 | 100 |
| comp61443c0 | 13644 | 100 |
| comp65057c0 | 1987 | 100 |
| comp62758c0 | 11524 | 99 |
| comp71127c0 | 659 | 99 |
| comp67289c2 | 1170 | 98 |
| comp58426c0 | 2224 | 97 |
| comp63881c0 | 789 | 97 |
| comp53580c0 | 2410 | 96 |
| comp65199c0 | 11530 | 96 |
| comp69575c0 | 1073 | 95 |
| comp72123c0 | 4688 | 95 |
| comp72586c0 | 1933 | 94 |
| comp68558c0 | 24362 | 92 |
| comp63881c1 | 863 | 91 |
| comp63023c0 | 952 | 90 |
| comp68860c0 | 107 | 88 |
| comp70162c0 | 2264 | 88 |
| comp70789c1 | 37169 | 88 |
| comp70950c0 | 401 | 88 |
| comp61839c0 | 18007 | 87 |
| comp58579c0 | 2831 | 86 |
| comp69964c0 | 94533 | 86 |
| comp59793c0 | 3416 | 85 |
| comp70697c0 | 17178 | 85 |
| comp60041c0 | 285 | 83 |
| comp71665c0 | 16662 | 83 |
| comp66501c0 | 1079 | 82 |
| comp58280c0 | 219 | 81 |
| comp63138c0 | 15126 | 80 |
| comp68103c0 | 353 | 80 |
| comp68782c0 | 172 | 80 |
| comp68772c0 | 9313 | 79 |
| comp58351c0 | 9771 | 78 |
| comp67774c0 | 4010 | 78 |
| comp71527c0 | 5506 | 78 |
| comp72297c1 | 17396 | 78 |
| comp60059c0 | 2564 | 77 |
| comp71878c0 | 6973 | 77 |
| comp64956c0 | 2847 | 76 |
| comp43529c0 | 1534 | 75 |
| comp59922c0 | 445 | 75 |
| comp60065c0 | 10863 | 75 |
| comp66715c0 | 5497 | 75 |
| comp69062c0 | 15231 | 75 |
| comp71689c0 | 678 | 75 |
| comp67643c0 | 1098 | 74 |
| comp68012c0 | 3762 | 74 |
| comp3167305c0 | 5140 | 73 |
| comp51270c0 | 117 | 73 |
| comp59085c0 | 2212 | 73 |
| comp61109c1 | 2345 | 73 |
| comp69508c1 | 26216 | 73 |
| comp63438c0 | 4768 | 72 |
| comp63809c0 | 338 | 72 |
| comp64459c0 | 982 | 72 |
| comp68095c0 | 9148 | 72 |
| comp68692c0 | 1573 | 72 |
| comp70232c0 | 2254 | 72 |
| comp58170c2 | 1636 | 71 |
| comp66754c0 | 603 | 71 |
| comp67296c0 | 1315 | 71 |
| comp69754c0 | 2230 | 71 |
| comp70223c1 | 12382 | 71 |
| comp55648c0 | 1551 | 70 |
| comp62879c0 | 13546 | 70 |
| comp62960c0 | 6624 | 70 |
| comp64705c0 | 5660 | 70 |
| comp65167c0 | 2354 | 70 |
| comp69131c0 | 1677 | 70 |
| comp47833c0 | 17875 | 69 |
| comp62674c0 | 2990 | 69 |
| comp67893c0 | 2156 | 69 |
| comp687c0 | 244 | 69 |
| comp70458c1 | 165 | 69 |
| comp70515c0 | 31365 | 68 |
| comp71318c0 | 6261 | 68 |
| comp60531c0 | 2376 | 67 |
| comp1897882c0 | 1342 | 66 |
| comp45557c0 | 13229 | 66 |
| comp51709c0 | 4934 | 66 |
| comp62767c0 | 5666 | 66 |
| comp64853c0 | 2279 | 66 |
| comp68608c0 | 3398 | 66 |
| comp42704c1 | 7300 | 64 |
| comp51082c0 | 37 | 64 |
| comp56228c0 | 1642 | 64 |
| comp59071c0 | 417 | 64 |
| comp61608c0 | 1991 | 64 |
| comp63447c0 | 349 | 64 |
| comp70476c0 | 2576 | 64 |
| comp71140c2 | 13006 | 64 |
| comp43117c0 | 17806 | 63 |
| comp60959c0 | 1874 | 63 |
| comp65743c1 | 5205 | 63 |
| comp54951c0 | 5693 | 62 |
| comp57173c0 | 97 | 62 |
| comp58503c0 | 1636 | 61 |
| comp72140c0 | 9624 | 61 |
| comp257413c0 | 10176 | 60 |
| comp53674c0 | 2313 | 60 |
| comp69833c1 | 1055 | 60 |
| comp70826c0 | 3908 | 60 |
| comp45352c0 | 4092 | 59 |
| comp54046c0 | 86 | 59 |
| comp59118c0 | 90 | 59 |
| comp60908c1 | 130 | 59 |
| comp57823c1 | 2001 | 58 |
| comp61952c0 | 4806 | 58 |
| comp68210c2 | 768 | 58 |
| comp71402c0 | 1869 | 58 |
| comp71434c0 | 1672 | 58 |
| comp1128253c0 | 800 | 57 |
| comp43430c0 | 391 | 57 |
| comp43035c0 | 336 | 56 |
| comp56209c0 | 2190 | 56 |
| comp61317c0 | 294 | 56 |
| comp61929c0 | 2960 | 56 |
| comp64271c0 | 34110 | 56 |
| comp67485c1 | 5114 | 56 |
| comp69164c0 | 15073 | 56 |
| comp70382c0 | 2394 | 56 |
| comp72086c1 | 9914 | 56 |
| comp60908c0 | 1083 | 55 |
| comp71773c0 | 2832 | 55 |
| comp72342c0 | 382 | 55 |
| comp22976c0 | 2471 | 54 |
| comp53791c0 | 608 | 54 |
| comp60370c0 | 8665 | 54 |
| comp64075c0 | 26 | 54 |
| comp65944c0 | 939 | 54 |
| comp66882c0 | 2801 | 54 |
| comp67136c0 | 1561 | 54 |
| comp68242c0 | 195 | 54 |
| comp72603c0 | 788 | 54 |
| comp64354c0 | 800 | 53 |
| comp71405c0 | 151 | 53 |
| comp71627c0 | 1176 | 53 |
| comp72709c0 | 9743 | 53 |
| comp44373c0 | 7939 | 52 |
| comp59371c0 | 29550 | 52 |
| comp65206c0 | 15818 | 52 |
| comp66198c0 | 51 | 52 |
| comp70755c0 | 1358 | 52 |
| comp758244c0 | 4706 | 52 |
| comp63448c1 | 128 | 51 |
| comp67691c0 | 1302 | 51 |
| comp60918c0 | 7082 | 50 |
| comp63879c0 | 1232 | 50 |
| comp67223c1 | 5235 | 50 |
| comp71620c0 | 1013 | 50 |
| comp723668c0 | 17257 | 50 |
| comp58004c0 | 1147 | 49 |
| comp58447c0 | 1255 | 49 |
| comp61260c0 | 1145 | 49 |
| comp62341c0 | 1180 | 49 |
| comp68573c0 | 3073 | 49 |
| comp69543c0 | 2390 | 49 |
| comp43387c0 | 734 | 48 |
| comp52740c1 | 12631 | 48 |
| comp52935c0 | 864 | 48 |
| comp63377c1 | 1572 | 48 |
| comp64088c0 | 3287 | 48 |
| comp64243c0 | 1515 | 48 |
| comp64841c0 | 521 | 48 |
| comp70459c0 | 951 | 48 |
| comp72092c1 | 15350 | 48 |
| comp72313c0 | 1904 | 48 |
| comp75175c0 | 1879 | 48 |
| comp1364549c0 | 26656 | 47 |
| comp49584c0 | 17066 | 47 |
| comp53381c0 | 765 | 47 |
| comp60987c0 | 332 | 47 |
| comp62803c0 | 2377 | 47 |
| comp66165c0 | 234 | 47 |
| comp68077c0 | 2065 | 47 |
| comp68238c0 | 196 | 47 |
| comp69676c1 | 1779 | 47 |
| comp63975c0 | 1009 | 46 |
| comp68196c0 | 37 | 46 |
| comp69351c0 | 1518 | 46 |
| comp70692c1 | 3425 | 46 |
| comp71147c0 | 771 | 46 |
| comp44531c0 | 8218 | 45 |
| comp49007c0 | 970 | 45 |
| comp58674c0 | 937 | 45 |
| comp64933c0 | 1464 | 45 |
| comp70408c0 | 1742 | 45 |
| comp51910c0 | 585 | 44 |
| comp61116c0 | 7671 | 44 |
| comp65061c0 | 267 | 44 |
| comp65623c0 | 407 | 44 |
| comp67323c1 | 31 | 44 |
| comp71251c0 | 655 | 44 |
| comp71528c0 | 569 | 44 |
| comp72575c2 | 1332 | 44 |
| comp72643c0 | 2279 | 44 |
| comp56584c0 | 26 | 43 |
| comp57443c0 | 4919 | 43 |
| comp58294c0 | 293 | 43 |
| comp59867c2 | 935 | 43 |
| comp64365c0 | 1906 | 43 |
| comp64408c0 | 382 | 43 |
| comp65623c1 | 246 | 43 |
| comp69447c1 | 2602 | 43 |
| comp69503c0 | 86 | 43 |
| comp70135c1 | 236 | 43 |
| comp72605c0 | 23380 | 43 |
| comp59478c1 | 3118 | 42 |
| comp60070c1 | 1552 | 42 |
| comp61793c0 | 1060 | 42 |
| comp62364c0 | 3968 | 42 |
| comp69757c0 | 579 | 42 |
| comp70296c0 | 1080 | 42 |
| comp72520c1 | 14576 | 42 |
| comp39333c0 | 14558 | 41 |
| comp64212c0 | 11071 | 41 |
| comp66771c1 | 44 | 41 |
| comp68100c0 | 254 | 41 |
| comp70407c0 | 351 | 41 |
| comp70748c2 | 395 | 41 |
| comp72078c0 | 19330 | 41 |
| comp2775c0 | 7364 | 40 |
| comp42704c0 | 2964 | 40 |
| comp56274c0 | 6361 | 40 |
| comp59554c0 | 1313 | 40 |
| comp59751c1 | 98 | 40 |
| comp60855c0 | 1524 | 40 |
| comp62495c1 | 5403 | 40 |
| comp64703c0 | 943 | 40 |
| comp70024c0 | 17180 | 40 |
| comp70461c0 | 7599 | 40 |
| comp70839c0 | 2984 | 40 |
| comp43269c0 | 1563 | 39 |
| comp64682c0 | 748 | 39 |
| comp66435c0 | 356 | 39 |
| comp67769c1 | 21398 | 39 |
| comp69212c0 | 52 | 39 |
| comp70055c0 | 942 | 39 |
| comp71767c0 | 2038 | 39 |
| comp71943c0 | 616 | 39 |
| comp72524c0 | 3282 | 39 |
| comp2610c0 | 360 | 38 |
| comp49365c0 | 4836 | 38 |
| comp50308c0 | 2358 | 38 |
| comp51438c0 | 2532 | 38 |
| comp55992c0 | 2618 | 38 |
| comp61157c0 | 5473 | 38 |
| comp62106c0 | 2343 | 38 |
| comp62890c0 | 3275 | 38 |
| comp64219c0 | 332 | 38 |
| comp64333c0 | 397 | 38 |
| comp64350c0 | 6487 | 38 |
| comp64357c0 | 600 | 38 |
| comp65829c0 | 668 | 38 |
| comp69128c0 | 2331 | 38 |
| comp69597c0 | 58 | 38 |
| comp70547c0 | 751 | 38 |
| comp70783c0 | 3139 | 38 |
| comp72179c0 | 5161 | 38 |
| comp44101c0 | 3203 | 37 |
| comp61109c0 | 193 | 37 |
| comp69163c6 | 1755 | 37 |
| comp69698c0 | 6593 | 37 |
| comp70555c0 | 7095 | 37 |
| comp72632c0 | 2206 | 37 |
| comp29951c0 | 4093 | 36 |
| comp52393c0 | 526 | 36 |
| comp55201c0 | 1138 | 36 |
| comp56746c0 | 2004 | 36 |
| comp58290c0 | 1971 | 36 |
| comp60491c0 | 7501 | 36 |
| comp63687c1 | 933 | 36 |
| comp65657c0 | 3351 | 36 |
| comp66055c0 | 1875 | 36 |
| comp68911c0 | 9610 | 36 |
| comp71733c0 | 2822 | 36 |
| comp71996c1 | 1803 | 36 |
| comp1212323c0 | 234 | 35 |
| comp59360c0 | 1036 | 35 |
| comp60729c0 | 1221 | 35 |
| comp62221c1 | 1327 | 35 |
| comp64161c0 | 541 | 35 |
| comp64420c0 | 2689 | 35 |
| comp64534c1 | 1023 | 35 |
| comp65470c0 | 5703 | 35 |
| comp67042c0 | 3555 | 35 |
| comp69892c0 | 2646 | 35 |
| comp70041c0 | 3034 | 35 |
| comp71082c0 | 911 | 35 |
| comp71599c0 | 1081 | 35 |
| comp71826c1 | 2356 | 35 |
| comp72184c0 | 1524 | 35 |
| comp25141c0 | 4209 | 34 |
| comp35268c0 | 300 | 34 |
| comp57306c0 | 760 | 34 |
| comp59318c3 | 968 | 34 |
| comp63685c0 | 995 | 34 |
| comp63804c0 | 11422 | 34 |
| comp63868c0 | 9784 | 34 |
| comp64006c0 | 926 | 34 |
| comp64680c1 | 61 | 34 |
| comp66560c0 | 1047 | 34 |
| comp66700c0 | 1627 | 34 |
| comp67062c0 | 293 | 34 |
| comp68218c1 | 2976 | 34 |
| comp71544c1 | 2430 | 34 |
| comp71548c0 | 104 | 34 |
| comp43840c0 | 136 | 33 |
| comp56788c0 | 588 | 33 |
| comp60761c0 | 872 | 33 |
| comp61120c0 | 4263 | 33 |
| comp67986c0 | 372 | 33 |
| comp68219c0 | 198 | 33 |
| comp68386c0 | 2363 | 33 |
| comp72630c0 | 1005 | 33 |
| comp1646c0 | 1458 | 32 |
| comp5033c0 | 18688 | 32 |
| comp50968c0 | 92 | 32 |
| comp55092c0 | 1232 | 32 |
| comp5538c0 | 10302 | 32 |
| comp61404c0 | 2815 | 32 |
| comp61518c0 | 1205 | 32 |
| comp62028c0 | 2491 | 32 |
| comp65928c0 | 4255 | 32 |
| comp66146c0 | 66 | 32 |
| comp68680c0 | 7946 | 32 |
| comp69669c0 | 727 | 32 |
| comp70979c0 | 1948 | 32 |
| comp32400c0 | 26 | 31 |
| comp44651c0 | 841 | 31 |
| comp51921c0 | 193 | 31 |
| comp53215c1 | 1347 | 31 |
| comp65223c0 | 711 | 31 |
| comp66790c0 | 30651 | 31 |
| comp68163c0 | 2763 | 31 |
| comp68182c1 | 388 | 31 |
| comp69315c0 | 582 | 31 |
| comp71005c2 | 1318 | 31 |
| comp72283c0 | 2857 | 31 |
| comp72753c0 | 823 | 31 |
| comp53595c0 | 671 | 30 |
| comp54029c1 | 6536 | 30 |
| comp57466c0 | 966 | 30 |
| comp57660c0 | 1716 | 30 |
| comp57897c0 | 1370 | 30 |
| comp59139c0 | 15964 | 30 |
| comp59356c0 | 190 | 30 |
| comp62485c1 | 167 | 30 |
| comp66433c0 | 901 | 30 |
| comp67129c1 | 1357 | 30 |
| comp68891c0 | 1713 | 30 |
| comp68939c0 | 1111 | 30 |
| comp70914c2 | 580 | 30 |
| comp72210c0 | 8420 | 30 |
| comp72417c0 | 10367 | 30 |
| comp72522c0 | 3928 | 30 |
| comp7534c0 | 14213 | 30 |
| comp87814c0 | 1324 | 30 |
| comp57377c0 | 500 | 29 |
| comp59221c0 | 1852 | 29 |
| comp59867c1 | 2092 | 29 |
| comp63035c1 | 484 | 29 |
| comp64371c0 | 2308 | 29 |
| comp66074c0 | 2758 | 29 |
| comp68222c0 | 794 | 29 |
| comp69054c1 | 68 | 29 |
| comp69580c0 | 7068 | 29 |
| comp70495c0 | 1099 | 29 |
| comp71259c0 | 685 | 29 |
| comp71269c0 | 806 | 29 |
| comp72423c1 | 4013 | 29 |
| comp72591c2 | 8512 | 29 |
| comp72609c1 | 444 | 29 |
| comp72739c1 | 2063 | 29 |
| comp1146949c0 | 1197 | 28 |
| comp51731c0 | 174 | 28 |
| comp63060c0 | 75 | 28 |
| comp63161c0 | 1593 | 28 |
| comp64605c4 | 153 | 28 |
| comp66772c0 | 2842 | 28 |
| comp66973c0 | 369 | 28 |
| comp67972c1 | 1479 | 28 |
| comp69460c0 | 13844 | 28 |
| comp69867c0 | 3570 | 28 |
| comp70621c0 | 732 | 28 |
| comp71732c0 | 2120 | 28 |
| comp71906c0 | 8167 | 28 |
| comp72267c0 | 407 | 28 |
| comp2935c0 | 3320 | 27 |
| comp42991c0 | 594 | 27 |
| comp44574c0 | 16 | 27 |
| comp54517c0 | 13 | 27 |
| comp55562c0 | 1641 | 27 |
| comp60968c0 | 1492 | 27 |
| comp61340c0 | 218 | 27 |
| comp625318c0 | 2656 | 27 |
| comp63860c0 | 2425 | 27 |
| comp66339c1 | 3556 | 27 |
| comp67700c0 | 560 | 27 |
| comp68027c0 | 919 | 27 |
| comp683924c0 | 299 | 27 |
| comp69319c0 | 5781 | 27 |
| comp69420c0 | 896 | 27 |
| comp72371c0 | 2508 | 27 |
| comp72476c1 | 4188 | 27 |
| comp72500c1 | 6073 | 27 |
| comp72657c0 | 1863 | 27 |
| comp1208460c0 | 556 | 26 |
| comp48164c0 | 1160 | 26 |
| comp49337c0 | 184 | 26 |
| comp56143c0 | 1416 | 26 |
| comp58149c1 | 1334 | 26 |
| comp59704c2 | 641 | 26 |
| comp60759c0 | 293 | 26 |
| comp62942c0 | 523 | 26 |
| comp63014c0 | 2886 | 26 |
| comp64144c0 | 2740 | 26 |
| comp65048c0 | 565 | 26 |
| comp65617c0 | 313 | 26 |
| comp65619c0 | 5661 | 26 |
| comp66092c0 | 1486 | 26 |
| comp67483c0 | 849 | 26 |
| comp68225c1 | 925 | 26 |
| comp70194c2 | 573 | 26 |
| comp70840c0 | 654 | 26 |
| comp71613c1 | 3893 | 26 |
| comp71736c1 | 2977 | 26 |
| comp72543c0 | 979 | 26 |
| comp31724c0 | 636 | 25 |
| comp44083c0 | 119 | 25 |
| comp52740c0 | 586 | 25 |
| comp53212c0 | 2128 | 25 |
| comp53982c0 | 2645 | 25 |
| comp54632c0 | 1009 | 25 |
| comp58109c0 | 631 | 25 |
| comp59077c2 | 2593 | 25 |
| comp60098c0 | 4213 | 25 |
| comp60744c0 | 3722 | 25 |
| comp61370c0 | 2919 | 25 |
| comp62724c0 | 979 | 25 |
| comp63545c0 | 280 | 25 |
| comp63617c0 | 1165 | 25 |
| comp68681c0 | 2424 | 25 |
| comp69676c3 | 998 | 25 |
| comp70441c0 | 14193 | 25 |
| comp71548c2 | 310 | 25 |
| comp71634c0 | 12580 | 25 |
| comp72156c0 | 3442 | 25 |
| comp41865c0 | 2593 | 24 |
| comp43906c0 | 297 | 24 |
| comp49030c0 | 454 | 24 |
| comp55364c0 | 368 | 24 |
| comp57509c0 | 380 | 24 |
| comp58370c0 | 782 | 24 |
| comp61212c1 | 96 | 24 |
| comp64687c0 | 4097 | 24 |
| comp65796c0 | 5553 | 24 |
| comp66408c0 | 935 | 24 |
| comp66966c0 | 155 | 24 |
| comp67399c1 | 143 | 24 |
| comp68746c0 | 123 | 24 |
| comp68970c0 | 2621 | 24 |
| comp69570c0 | 5260 | 24 |
| comp70158c0 | 5757 | 24 |
| comp70479c1 | 932 | 24 |
| comp70605c0 | 553 | 24 |
| comp70881c0 | 294 | 24 |
| comp71985c0 | 1404 | 24 |
| comp72754c1 | 389 | 24 |
| comp757644c0 | 1777 | 24 |
| comp130c0 | 269 | 23 |
| comp33363c0 | 1114 | 23 |
| comp44829c0 | 142 | 23 |
| comp51701c0 | 319 | 23 |
| comp52097c0 | 2835 | 23 |
| comp55112c0 | 7786 | 23 |
| comp55214c0 | 899 | 23 |
| comp56403c1 | 61 | 23 |
| comp56673c0 | 1056 | 23 |
| comp58116c0 | 6720 | 23 |
| comp61647c0 | 496 | 23 |
| comp62576c0 | 478 | 23 |
| comp63925c0 | 1063 | 23 |
| comp64359c0 | 820 | 23 |
| comp65128c1 | 1211 | 23 |
| comp66458c0 | 8581 | 23 |
| comp66689c0 | 1151 | 23 |
| comp66792c0 | 348 | 23 |
| comp66951c0 | 1068 | 23 |
| comp67227c0 | 810 | 23 |
| comp67271c0 | 591 | 23 |
| comp67886c1 | 428 | 23 |
| comp68384c0 | 1378 | 23 |
| comp68889c0 | 638 | 23 |
| comp69098c0 | 2339 | 23 |
| comp69579c0 | 360 | 23 |
| comp70455c0 | 1814 | 23 |
| comp71166c0 | 979 | 23 |
| comp71604c1 | 1690 | 23 |
| comp71837c0 | 2623 | 23 |
| comp72052c4 | 647 | 23 |
| comp72199c1 | 1243 | 23 |
| comp72419c0 | 116 | 23 |
| comp72588c0 | 1943 | 23 |
| comp72706c0 | 2108 | 23 |
| comp4043c0 | 958 | 22 |
| comp43030c0 | 3320 | 22 |
| comp43701c0 | 715 | 22 |
| comp49166c0 | 771 | 22 |
| comp50614c0 | 447 | 22 |
| comp52061c0 | 6994 | 22 |
| comp55398c1 | 149 | 22 |
| comp56084c0 | 1808 | 22 |
| comp57098c0 | 4453 | 22 |
| comp58293c1 | 373 | 22 |
| comp58853c0 | 544 | 22 |
| comp61006c0 | 2206 | 22 |
| comp61261c1 | 554 | 22 |
| comp63894c0 | 2447 | 22 |
| comp64138c0 | 1795 | 22 |
| comp64225c0 | 1301 | 22 |
| comp64250c1 | 839 | 22 |
| comp65317c0 | 1049 | 22 |
| comp66061c0 | 7277 | 22 |
| comp66503c0 | 2870 | 22 |
| comp68215c0 | 196 | 22 |
| comp68481c0 | 1301 | 22 |
| comp69181c0 | 1885 | 22 |
| comp69595c0 | 823 | 22 |
| comp70155c0 | 1909 | 22 |
| comp71304c0 | 2278 | 22 |
| comp71751c0 | 331 | 22 |
| comp71807c0 | 1820 | 22 |
| comp72733c0 | 3354 | 22 |
| comp72734c0 | 878 | 22 |
| comp23260c0 | 1430 | 21 |
| comp36284c0 | 2684 | 21 |
| comp43207c0 | 100 | 21 |
| comp49249c0 | 146 | 21 |
| comp51863c0 | 1806 | 21 |
| comp52451c0 | 500 | 21 |
| comp53751c0 | 162 | 21 |
| comp54250c1 | 6 | 21 |
| comp55802c0 | 717 | 21 |
| comp55973c0 | 898 | 21 |
| comp56308c0 | 39 | 21 |
| comp57774c0 | 1982 | 21 |
| comp60229c0 | 2120 | 21 |
| comp60272c1 | 607 | 21 |
| comp60702c0 | 2249 | 21 |
| comp60713c0 | 842 | 21 |
| comp60771c1 | 2244 | 21 |
| comp61065c0 | 696 | 21 |
| comp61297c1 | 1121 | 21 |
| comp61870c0 | 2664 | 21 |
| comp62537c0 | 1182 | 21 |
| comp64158c0 | 1147 | 21 |
| comp64272c0 | 4630 | 21 |
| comp65588c0 | 91 | 21 |
| comp66510c1 | 840 | 21 |
| comp66887c1 | 69 | 21 |
| comp67484c1 | 349 | 21 |
| comp68459c0 | 536 | 21 |
| comp68599c0 | 70 | 21 |
| comp69051c0 | 3889 | 21 |
| comp69279c0 | 448 | 21 |
| comp70060c0 | 707 | 21 |
| comp70750c0 | 534 | 21 |
| comp71704c0 | 8629 | 21 |
| comp72097c0 | 1090 | 21 |
| comp72204c0 | 1565 | 21 |
| comp72246c0 | 191 | 21 |
| comp1145922c0 | 1201 | 20 |
| comp38606c0 | 345 | 20 |
| comp46419c0 | 951 | 20 |
| comp50472c0 | 230 | 20 |
| comp53823c0 | 50 | 20 |
| comp56329c0 | 78 | 20 |
| comp57501c0 | 1229 | 20 |
| comp58064c0 | 376 | 20 |
| comp59719c0 | 357 | 20 |
| comp61528c0 | 409 | 20 |
| comp64880c0 | 570 | 20 |
| comp65202c0 | 9263 | 20 |
| comp66814c0 | 688 | 20 |
| comp67293c0 | 1291 | 20 |
| comp67497c0 | 2204 | 20 |
| comp68493c0 | 70 | 20 |
| comp68732c0 | 315 | 20 |
| comp69927c0 | 638 | 20 |
| comp70000c1 | 33 | 20 |
| comp72241c0 | 129 | 20 |
| comp72335c4 | 1476 | 20 |
| comp72625c0 | 2947 | 20 |
| comp851492c0 | 1512 | 20 |
| comp8821c0 | 1750 | 20 |
| comp1708748c0 | 128 | 19 |
| comp43747c0 | 244 | 19 |
| comp51477c0 | 54 | 19 |
| comp53677c0 | 1415 | 19 |
| comp56828c0 | 8203 | 19 |
| comp58436c0 | 545 | 19 |
| comp60070c0 | 69 | 19 |
| comp62976c0 | 596 | 19 |
| comp64488c0 | 861 | 19 |
| comp64780c0 | 2086 | 19 |
| comp65793c1 | 1398 | 19 |
| comp66791c0 | 562 | 19 |
| comp67201c0 | 3563 | 19 |
| comp67883c0 | 953 | 19 |
| comp68032c0 | 4136 | 19 |
| comp68111c0 | 723 | 19 |
| comp6991c0 | 1231 | 19 |
| comp70236c0 | 367 | 19 |
| comp70833c0 | 748 | 19 |
| comp71139c0 | 923 | 19 |
| comp71526c0 | 186 | 19 |
| comp71548c1 | 2590 | 19 |
| comp71611c0 | 39 | 19 |
| comp72571c0 | 215 | 19 |
| comp72633c0 | 2301 | 19 |
| comp72637c0 | 734 | 19 |
| comp75334c0 | 504 | 19 |
| comp1054760c0 | 7050 | 18 |
| comp36138c0 | 291 | 18 |
| comp42581c0 | 64 | 18 |
| comp43733c0 | 8486 | 18 |
| comp44188c0 | 5629 | 18 |
| comp45318c0 | 1765 | 18 |
| comp47663c0 | 1794 | 18 |
| comp50482c0 | 624 | 18 |
| comp57246c1 | 871 | 18 |
| comp575492c0 | 774 | 18 |
| comp57644c0 | 3174 | 18 |
| comp57995c0 | 4014 | 18 |
| comp59544c1 | 1264 | 18 |
| comp59925c0 | 141 | 18 |
| comp60074c0 | 325 | 18 |
| comp60175c0 | 1098 | 18 |
| comp60337c0 | 1713 | 18 |
| comp60508c0 | 996 | 18 |
| comp61291c0 | 557 | 18 |
| comp61423c0 | 2677 | 18 |
| comp62104c0 | 2829 | 18 |
| comp62302c0 | 683 | 18 |
| comp65358c0 | 82 | 18 |
| comp65648c0 | 77 | 18 |
| comp66019c1 | 8 | 18 |
| comp66022c0 | 1808 | 18 |
| comp66490c1 | 714 | 18 |
| comp6673c0 | 300 | 18 |
| comp66771c0 | 12 | 18 |
| comp66961c0 | 2069 | 18 |
| comp67233c0 | 1753 | 18 |
| comp67258c0 | 1107 | 18 |
| comp68633c0 | 166 | 18 |
| comp70050c0 | 412 | 18 |
| comp70250c0 | 1219 | 18 |
| comp70282c0 | 1562 | 18 |
| comp71132c0 | 891 | 18 |
| comp71322c0 | 6010 | 18 |
| comp71915c0 | 1300 | 18 |
| comp72316c0 | 195 | 18 |
| comp72483c0 | 5084 | 18 |
| comp91678c0 | 485 | 18 |
| comp14237c0 | 195 | 17 |
| comp35726c0 | 508 | 17 |
| comp38840c0 | 469 | 17 |
| comp39020c0 | 7019 | 17 |
| comp43628c0 | 801 | 17 |
| comp43956c0 | 1008 | 17 |
| comp44944c0 | 1001 | 17 |
| comp45161c0 | 172 | 17 |
| comp50500c0 | 5577 | 17 |
| comp54305c0 | 285 | 17 |
| comp54886c1 | 927 | 17 |
| comp57490c0 | 65 | 17 |
| comp60449c0 | 823 | 17 |
| comp60491c2 | 3617 | 17 |
| comp61201c0 | 387 | 17 |
| comp61477c0 | 1030 | 17 |
| comp62838c1 | 482 | 17 |
| comp63316c0 | 405 | 17 |
| comp63400c0 | 305 | 17 |
| comp63973c0 | 648 | 17 |
| comp64491c1 | 5243 | 17 |
| comp64586c0 | 502 | 17 |
| comp64929c0 | 5970 | 17 |
| comp65233c2 | 782 | 17 |
| comp65259c0 | 1003 | 17 |
| comp65292c0 | 231 | 17 |
| comp66155c0 | 102 | 17 |
| comp66415c0 | 3502 | 17 |
| comp66786c0 | 320 | 17 |
| comp67657c0 | 1150 | 17 |
| comp68150c0 | 358 | 17 |
| comp68169c0 | 1924 | 17 |
| comp68726c0 | 20784 | 17 |
| comp68811c1 | 1700 | 17 |
| comp69312c0 | 259 | 17 |
| comp69399c0 | 3535 | 17 |
| comp69603c0 | 911 | 17 |
| comp69791c0 | 2515 | 17 |
| comp69837c0 | 542 | 17 |
| comp69914c0 | 5139 | 17 |
| comp69970c0 | 108 | 17 |
| comp70043c0 | 689 | 17 |
| comp70127c2 | 720 | 17 |
| comp70724c0 | 1582 | 17 |
| comp70770c0 | 1831 | 17 |
| comp70921c0 | 636 | 17 |
| comp71077c0 | 3959 | 17 |
| comp71336c0 | 349 | 17 |
| comp71408c0 | 230 | 17 |
| comp71892c0 | 837 | 17 |
| comp72011c0 | 3789 | 17 |
| comp72218c0 | 2549 | 17 |
| comp72237c1 | 1237 | 17 |
| comp72287c0 | 9470 | 17 |
| comp72310c0 | 750 | 17 |
| comp72386c0 | 4716 | 17 |
| comp72539c0 | 441 | 17 |
| comp2644c0 | 846 | 16 |
| comp28213c0 | 38 | 16 |
| comp42880c1 | 296 | 16 |
| comp53188c0 | 793 | 16 |
| comp53437c0 | 1114 | 16 |
| comp55836c0 | 363 | 16 |
| comp56674c0 | 2257 | 16 |
| comp58360c1 | 2743 | 16 |
| comp59006c0 | 3149 | 16 |
| comp59816c0 | 1221 | 16 |
| comp59964c0 | 386 | 16 |
| comp60103c2 | 1825 | 16 |
| comp605623c0 | 132 | 16 |
| comp63014c1 | 21511 | 16 |
| comp63094c0 | 1125 | 16 |
| comp64013c1 | 335 | 16 |
| comp64524c0 | 1779 | 16 |
| comp64943c0 | 97 | 16 |
| comp65378c0 | 1296 | 16 |
| comp65382c0 | 6701 | 16 |
| comp66096c0 | 2311 | 16 |
| comp66589c0 | 1628 | 16 |
| comp67792c0 | 563 | 16 |
| comp67930c0 | 17965 | 16 |
| comp67983c1 | 1236 | 16 |
| comp68218c0 | 105 | 16 |
| comp68271c0 | 622 | 16 |
| comp68348c0 | 223 | 16 |
| comp68378c0 | 477 | 16 |
| comp68675c0 | 193 | 16 |
| comp68786c0 | 731 | 16 |
| comp69163c5 | 69 | 16 |
| comp69972c0 | 324 | 16 |
| comp70882c1 | 856 | 16 |
| comp71245c1 | 258 | 16 |
| comp71504c0 | 334 | 16 |
| comp71525c0 | 5576 | 16 |
| comp71735c0 | 1015 | 16 |
| comp71756c0 | 1562 | 16 |
| comp72320c0 | 912 | 16 |
| comp72391c0 | 6217 | 16 |
| comp72431c0 | 2094 | 16 |
| comp72536c0 | 291 | 16 |
| comp72656c0 | 687 | 16 |
| comp74047c0 | 4736 | 16 |
| comp12812c0 | 390 | 15 |
| comp24461c0 | 372 | 15 |
| comp42638c0 | 1003 | 15 |
| comp43354c0 | 485 | 15 |
| comp44808c1 | 2207 | 15 |
| comp46972c0 | 350 | 15 |
| comp49545c0 | 185 | 15 |
| comp50639c0 | 80 | 15 |
| comp51175c0 | 446 | 15 |
| comp51706c1 | 1036 | 15 |
| comp52820c1 | 3197 | 15 |
| comp53827c0 | 2538 | 15 |
| comp55145c0 | 2491 | 15 |
| comp55263c0 | 4045 | 15 |
| comp56569c0 | 437 | 15 |
| comp56592c0 | 198 | 15 |
| comp56858c0 | 6475 | 15 |
| comp57611c0 | 601 | 15 |
| comp59164c1 | 88 | 15 |
| comp61007c0 | 1379 | 15 |
| comp6225c0 | 647 | 15 |
| comp62784c0 | 3073 | 15 |
| comp62813c0 | 1875 | 15 |
| comp62900c0 | 235 | 15 |
| comp63141c1 | 1295 | 15 |
| comp63428c0 | 785 | 15 |
| comp63429c0 | 681 | 15 |
| comp63454c0 | 1504 | 15 |
| comp63509c0 | 510 | 15 |
| comp64260c0 | 1322 | 15 |
| comp64516c0 | 1329 | 15 |
| comp64544c0 | 793 | 15 |
| comp64847c0 | 172 | 15 |
| comp64944c0 | 1164 | 15 |
| comp66182c0 | 1994 | 15 |
| comp66427c0 | 1503 | 15 |
| comp66812c1 | 109 | 15 |
| comp67611c0 | 3009 | 15 |
| comp67922c0 | 433 | 15 |
| comp67959c0 | 1953 | 15 |
| comp68233c0 | 1288 | 15 |
| comp68597c2 | 3147 | 15 |
| comp68790c1 | 2008 | 15 |
| comp69261c0 | 1941 | 15 |
| comp69367c0 | 944 | 15 |
| comp70280c0 | 299 | 15 |
| comp70369c0 | 234 | 15 |
| comp70384c0 | 211 | 15 |
| comp70577c2 | 2410 | 15 |
| comp70969c0 | 6143 | 15 |
| comp71064c0 | 522 | 15 |
| comp71444c0 | 2690 | 15 |
| comp71999c0 | 364 | 15 |
| comp72472c0 | 415 | 15 |
| comp72544c0 | 235 | 15 |
| comp72592c0 | 1190 | 15 |
| comp72611c0 | 915 | 15 |
| comp3638c0 | 2501 | 14 |
| comp42526c0 | 977 | 14 |
| comp43555c0 | 2983 | 14 |
| comp45028c0 | 1204 | 14 |
| comp51351c0 | 782 | 14 |
| comp52160c0 | 795 | 14 |
| comp54149c0 | 1638 | 14 |
| comp56021c0 | 301 | 14 |
| comp57044c0 | 1056 | 14 |
| comp57365c0 | 418 | 14 |
| comp57663c0 | 3788 | 14 |
| comp57715c0 | 258 | 14 |
| comp58332c1 | 516 | 14 |
| comp58677c0 | 693 | 14 |
| comp58712c1 | 1566 | 14 |
| comp59475c0 | 474 | 14 |
| comp59751c0 | 6463 | 14 |
| comp60236c0 | 1830 | 14 |
| comp60813c0 | 549 | 14 |
| comp61881c0 | 499 | 14 |
| comp62082c0 | 725 | 14 |
| comp62297c1 | 1293 | 14 |
| comp62370c0 | 4278 | 14 |
| comp62625c0 | 1501 | 14 |
| comp63291c0 | 1145 | 14 |
| comp63513c1 | 1932 | 14 |
| comp64380c0 | 1912 | 14 |
| comp64946c1 | 702 | 14 |
| comp65200c0 | 4755 | 14 |
| comp65550c1 | 812 | 14 |
| comp66546c0 | 217 | 14 |
| comp66610c0 | 2761 | 14 |
| comp66732c0 | 1056 | 14 |
| comp67353c0 | 1390 | 14 |
| comp67489c0 | 2736 | 14 |
| comp67732c0 | 4642 | 14 |
| comp68064c5 | 1582 | 14 |
| comp68149c0 | 252 | 14 |
| comp68335c0 | 376 | 14 |
| comp68504c1 | 73 | 14 |
| comp68526c0 | 1075 | 14 |
| comp68577c0 | 660 | 14 |
| comp68922c0 | 2194 | 14 |
| comp69079c1 | 97 | 14 |
| comp69244c0 | 2968 | 14 |
| comp69457c0 | 158 | 14 |
| comp69648c0 | 906 | 14 |
| comp69902c0 | 267 | 14 |
| comp69984c0 | 4751 | 14 |
| comp70180c0 | 13260 | 14 |
| comp70197c0 | 692 | 14 |
| comp70236c4 | 3470 | 14 |
| comp71839c0 | 701 | 14 |
| comp72147c0 | 6876 | 14 |
| comp72160c0 | 2845 | 14 |
| comp72268c0 | 197 | 14 |
| comp72466c0 | 733 | 14 |
| comp72671c0 | 2203 | 14 |
| comp72714c0 | 2689 | 14 |
| comp1155c0 | 1029 | 13 |
| comp44020c0 | 766 | 13 |
| comp44877c0 | 715 | 13 |
| comp45050c1 | 1373 | 13 |
| comp47864c0 | 1009 | 13 |
| comp50121c0 | 901 | 13 |
| comp52446c0 | 2653 | 13 |
| comp52471c0 | 497 | 13 |
| comp53653c0 | 1687 | 13 |
| comp54311c0 | 1978 | 13 |
| comp54572c0 | 1365 | 13 |
| comp55120c0 | 2029 | 13 |
| comp55207c0 | 1280 | 13 |
| comp55298c1 | 630 | 13 |
| comp55530c0 | 913 | 13 |
| comp55988c0 | 1295 | 13 |
| comp55996c0 | 452 | 13 |
| comp56345c1 | 388 | 13 |
| comp56435c0 | 1420 | 13 |
| comp57820c0 | 315 | 13 |
| comp58446c0 | 1525 | 13 |
| comp58483c0 | 1698 | 13 |
| comp58491c0 | 1424 | 13 |
| comp58557c0 | 836 | 13 |
| comp59001c0 | 3334 | 13 |
| comp59144c0 | 3000 | 13 |
| comp59348c0 | 675 | 13 |
| comp59356c1 | 823 | 13 |
| comp60752c3 | 2667 | 13 |
| comp60892c0 | 338 | 13 |
| comp61729c1 | 1185 | 13 |
| comp61796c0 | 73 | 13 |
| comp63009c0 | 254 | 13 |
| comp63465c0 | 940 | 13 |
| comp63568c0 | 1114 | 13 |
| comp64353c0 | 759 | 13 |
| comp65025c0 | 980 | 13 |
| comp65318c0 | 3335 | 13 |
| comp65647c0 | 34 | 13 |
| comp66133c0 | 24449 | 13 |
| comp66408c1 | 594 | 13 |
| comp67155c0 | 7499 | 13 |
| comp67567c0 | 4364 | 13 |
| comp67776c0 | 1902 | 13 |
| comp68161c0 | 1169 | 13 |
| comp68659c0 | 1433 | 13 |
| comp68717c0 | 590 | 13 |
| comp68903c0 | 1375 | 13 |
| comp69046c0 | 3029 | 13 |
| comp69205c0 | 2787 | 13 |
| comp70375c1 | 1538 | 13 |
| comp70445c0 | 43 | 13 |
| comp70474c0 | 14347 | 13 |
| comp70948c0 | 3662 | 13 |
| comp70980c0 | 485 | 13 |
| comp71360c0 | 1173 | 13 |
| comp71420c0 | 339 | 13 |
| comp71462c0 | 100 | 13 |
| comp71851c0 | 574 | 13 |
| comp71958c0 | 2964 | 13 |
| comp72207c0 | 7032 | 13 |
| comp72257c0 | 1839 | 13 |
| comp72304c0 | 1590 | 13 |
| comp72353c0 | 45615 | 13 |
| comp72448c0 | 3274 | 13 |
| comp72465c0 | 1650 | 13 |
| comp72628c0 | 1036 | 13 |
| comp72679c0 | 377 | 13 |
| comp1073c0 | 565 | 12 |
| comp35450c0 | 369 | 12 |
| comp42882c0 | 2465 | 12 |
| comp43443c0 | 74 | 12 |
| comp43598c0 | 173 | 12 |
| comp43827c0 | 1470 | 12 |
| comp44040c0 | 705 | 12 |
| comp44144c0 | 768 | 12 |
| comp45756c0 | 231 | 12 |
| comp50733c0 | 2326 | 12 |
| comp51377c0 | 880 | 12 |
| comp52743c0 | 5443 | 12 |
| comp53336c0 | 1134 | 12 |
| comp53812c0 | 809 | 12 |
| comp54950c0 | 854 | 12 |
| comp55733c0 | 561 | 12 |
| comp56102c1 | 849 | 12 |
| comp56127c0 | 322 | 12 |
| comp57261c0 | 400 | 12 |
| comp58126c0 | 27 | 12 |
| comp58193c0 | 281 | 12 |
| comp58247c0 | 116 | 12 |
| comp58467c0 | 834 | 12 |
| comp58595c0 | 429 | 12 |
| comp58645c1 | 1091 | 12 |
| comp59141c0 | 1045 | 12 |
| comp59433c0 | 1322 | 12 |
| comp59592c0 | 368 | 12 |
| comp60072c0 | 357 | 12 |
| comp60653c0 | 237 | 12 |
| comp60956c0 | 625 | 12 |
| comp61439c0 | 352 | 12 |
| comp61548c0 | 1695 | 12 |
| comp619695c0 | 1375 | 12 |
| comp62029c0 | 1376 | 12 |
| comp62570c0 | 391 | 12 |
| comp62825c0 | 1178 | 12 |
| comp62922c0 | 342 | 12 |
| comp62923c0 | 137 | 12 |
| comp63007c0 | 623 | 12 |
| comp63267c0 | 1029 | 12 |
| comp63677c0 | 679 | 12 |
| comp64308c0 | 241 | 12 |
| comp64659c1 | 189 | 12 |
| comp64753c0 | 156 | 12 |
| comp64783c0 | 338 | 12 |
| comp64852c0 | 234 | 12 |
| comp64928c0 | 465 | 12 |
| comp65533c0 | 844 | 12 |
| comp66386c4 | 2173 | 12 |
| comp66517c0 | 541 | 12 |
| comp67175c0 | 2482 | 12 |
| comp67321c0 | 1479 | 12 |
| comp67651c1 | 1634 | 12 |
| comp68208c0 | 1540 | 12 |
| comp68910c0 | 8970 | 12 |
| comp69169c1 | 206 | 12 |
| comp69746c0 | 627 | 12 |
| comp69873c0 | 744 | 12 |
| comp69876c0 | 4349 | 12 |
| comp69988c0 | 608 | 12 |
| comp70089c0 | 273 | 12 |
| comp70127c1 | 745 | 12 |
| comp70292c2 | 852 | 12 |
| comp70609c0 | 560 | 12 |
| comp70728c0 | 376 | 12 |
| comp70984c0 | 392 | 12 |
| comp70986c0 | 608 | 12 |
| comp71032c0 | 1437 | 12 |
| comp71464c0 | 3810 | 12 |
| comp71548c3 | 410 | 12 |
| comp71576c0 | 5312 | 12 |
| comp71715c0 | 1507 | 12 |
| comp71764c0 | 1751 | 12 |
| comp71968c0 | 1279 | 12 |
| comp72085c0 | 759 | 12 |
| comp72202c0 | 928 | 12 |
| comp72400c0 | 2191 | 12 |
| comp72416c0 | 1424 | 12 |
| comp72664c0 | 985 | 12 |
| comp72749c0 | 443 | 12 |
| comp34332c1 | 870 | 11 |
| comp35564c0 | 1193 | 11 |
| comp35778c0 | 12 | 11 |
| comp36512c0 | 916 | 11 |
| comp43796c0 | 3476 | 11 |
| comp45225c0 | 2728 | 11 |
| comp50322c0 | 1995 | 11 |
| comp50386c0 | 345 | 11 |
| comp51372c0 | 2164 | 11 |
| comp51936c1 | 152 | 11 |
| comp52474c0 | 2951 | 11 |
| comp53917c3 | 30 | 11 |
| comp54807c0 | 1094 | 11 |
| comp55752c0 | 96 | 11 |
| comp56706c0 | 937 | 11 |
| comp56853c0 | 2726 | 11 |
| comp56949c0 | 137 | 11 |
| comp58111c0 | 2157 | 11 |
| comp58661c0 | 110 | 11 |
| comp59141c2 | 49 | 11 |
| comp59272c0 | 543 | 11 |
| comp59522c0 | 4629 | 11 |
| comp59904c0 | 427 | 11 |
| comp60075c0 | 19 | 11 |
| comp60812c0 | 358 | 11 |
| comp61011c0 | 455 | 11 |
| comp61047c1 | 2289 | 11 |
| comp61272c0 | 1757 | 11 |
| comp61296c0 | 4373 | 11 |
| comp61537c0 | 403 | 11 |
| comp61674c0 | 5329 | 11 |
| comp61707c0 | 1819 | 11 |
| comp62277c0 | 433 | 11 |
| comp62437c0 | 488 | 11 |
| comp62492c0 | 566 | 11 |
| comp63039c1 | 140 | 11 |
| comp63151c0 | 3277 | 11 |
| comp63916c0 | 656 | 11 |
| comp64421c0 | 1341 | 11 |
| comp64434c0 | 450 | 11 |
| comp64592c0 | 83 | 11 |
| comp64761c0 | 1220 | 11 |
| comp65363c1 | 86 | 11 |
| comp66583c1 | 643 | 11 |
| comp66592c0 | 702 | 11 |
| comp66620c0 | 342 | 11 |
| comp66812c2 | 955 | 11 |
| comp66944c0 | 481 | 11 |
| comp66973c1 | 3678 | 11 |
| comp67166c0 | 1520 | 11 |
| comp67224c0 | 1201 | 11 |
| comp67410c1 | 563 | 11 |
| comp68511c0 | 208 | 11 |
| comp68691c0 | 4274 | 11 |
| comp68702c1 | 1596 | 11 |
| comp69088c0 | 361 | 11 |
| comp69163c0 | 1195 | 11 |
| comp69423c0 | 508 | 11 |
| comp69457c1 | 228 | 11 |
| comp69607c3 | 581 | 11 |
| comp69725c0 | 421 | 11 |
| comp69877c0 | 1627 | 11 |
| comp69903c0 | 895 | 11 |
| comp69995c0 | 2840 | 11 |
| comp70257c0 | 1386 | 11 |
| comp70346c0 | 523 | 11 |
| comp70375c0 | 1957 | 11 |
| comp70781c0 | 986 | 11 |
| comp70913c0 | 220 | 11 |
| comp70961c0 | 766 | 11 |
| comp71174c0 | 456 | 11 |
| comp71267c0 | 1097 | 11 |
| comp71487c1 | 1099 | 11 |
| comp71650c0 | 15152 | 11 |
| comp71902c0 | 1188 | 11 |
| comp71988c0 | 12621 | 11 |
| comp72192c0 | 404 | 11 |
| comp72253c0 | 338 | 11 |
| comp72307c0 | 2376 | 11 |
| comp72492c0 | 239 | 11 |
| comp72604c0 | 4642 | 11 |
| comp72858c0 | 107 | 11 |
| comp860579c0 | 473 | 11 |
| comp864168c0 | 2872 | 11 |
| comp2261c0 | 69 | 10 |
| comp2400770c0 | 1555 | 10 |
| comp263998c0 | 764 | 10 |
| comp35421c0 | 279 | 10 |
| comp35754c0 | 934 | 10 |
| comp37335c0 | 162 | 10 |
| comp40334c0 | 572 | 10 |
| comp43160c0 | 1278 | 10 |
| comp43270c0 | 2044 | 10 |
| comp44808c0 | 805 | 10 |
| comp45439c0 | 502 | 10 |
| comp47803c0 | 85 | 10 |
| comp51435c0 | 196 | 10 |
| comp51488c0 | 1618 | 10 |
| comp51505c0 | 153 | 10 |
| comp51544c0 | 466 | 10 |
| comp52747c0 | 328 | 10 |
| comp53359c0 | 2952 | 10 |
| comp55398c0 | 1581 | 10 |
| comp55561c0 | 1018 | 10 |
| comp56480c0 | 693 | 10 |
| comp56735c0 | 416 | 10 |
| comp568790c0 | 1857 | 10 |
| comp57215c0 | 147 | 10 |
| comp57550c0 | 1374 | 10 |
| comp57732c0 | 699 | 10 |
| comp57859c0 | 373 | 10 |
| comp57919c0 | 1077 | 10 |
| comp58225c0 | 1307 | 10 |
| comp58231c0 | 328 | 10 |
| comp58640c1 | 256 | 10 |
| comp58986c0 | 425 | 10 |
| comp59077c0 | 2757 | 10 |
| comp59528c0 | 328 | 10 |
| comp60521c0 | 819 | 10 |
| comp60650c0 | 3126 | 10 |
| comp61019c0 | 184 | 10 |
| comp61126c0 | 359 | 10 |
| comp61724c0 | 294 | 10 |
| comp62008c0 | 620 | 10 |
| comp62109c0 | 1137 | 10 |
| comp62110c1 | 829 | 10 |
| comp62234c0 | 2242 | 10 |
| comp62524c0 | 117 | 10 |
| comp62843c0 | 1908 | 10 |
| comp63035c0 | 488 | 10 |
| comp63123c0 | 563 | 10 |
| comp63187c0 | 221 | 10 |
| comp63230c0 | 1589 | 10 |
| comp63682c0 | 180 | 10 |
| comp63744c0 | 778 | 10 |
| comp63776c1 | 2 | 10 |
| comp63857c0 | 264 | 10 |
| comp63957c0 | 558 | 10 |
| comp63974c1 | 1172 | 10 |
| comp64543c0 | 2449 | 10 |
| comp64604c0 | 202 | 10 |
| comp64980c0 | 267 | 10 |
| comp65690c0 | 372 | 10 |
| comp65803c0 | 909 | 10 |
| comp65993c0 | 2250 | 10 |
| comp66041c0 | 1211 | 10 |
| comp6636c0 | 933 | 10 |
| comp66776c2 | 2413 | 10 |
| comp66944c1 | 102 | 10 |
| comp67071c0 | 726 | 10 |
| comp67201c1 | 2490 | 10 |
| comp67212c0 | 322 | 10 |
| comp67553c1 | 3844 | 10 |
| comp67555c0 | 244 | 10 |
| comp67566c0 | 2585 | 10 |
| comp67756c0 | 609 | 10 |
| comp67850c0 | 383 | 10 |
| comp67921c0 | 88 | 10 |
| comp68204c0 | 1076 | 10 |
| comp68225c0 | 1144 | 10 |
| comp68232c0 | 511 | 10 |
| comp68238c3 | 14 | 10 |
| comp68342c0 | 1849 | 10 |
| comp68820c0 | 2887 | 10 |
| comp68952c0 | 312 | 10 |
| comp69017c0 | 695 | 10 |
| comp69165c0 | 1410 | 10 |
| comp69242c0 | 1382 | 10 |
| comp69375c0 | 2902 | 10 |
| comp69539c0 | 1168 | 10 |
| comp69816c0 | 530 | 10 |
| comp69834c1 | 430 | 10 |
| comp69967c0 | 1612 | 10 |
| comp69973c0 | 833 | 10 |
| comp69996c0 | 712 | 10 |
| comp70059c0 | 159 | 10 |
| comp70294c0 | 6 | 10 |
| comp70400c0 | 1299 | 10 |
| comp70613c0 | 962 | 10 |
| comp70679c0 | 3103 | 10 |
| comp70694c0 | 3948 | 10 |
| comp70712c0 | 348 | 10 |
| comp71218c0 | 837 | 10 |
| comp71644c0 | 984 | 10 |
| comp71758c1 | 207 | 10 |
| comp71833c0 | 1738 | 10 |
| comp71961c0 | 3385 | 10 |
| comp72065c2 | 487 | 10 |
| comp72194c0 | 2230 | 10 |
| comp72286c0 | 441 | 10 |
| comp72310c1 | 1786 | 10 |
| comp72495c0 | 779 | 10 |
| comp72502c0 | 783 | 10 |
| comp72540c1 | 4962 | 10 |
| comp72545c0 | 322 | 10 |
| comp72574c0 | 2522 | 10 |
| comp72638c0 | 1090 | 10 |
| comp72669c0 | 576 | 10 |
| comp72707c0 | 1419 | 10 |
| comp857529c0 | 1053 | 10 |
| comp2734c0 | 164 | 9 |
| comp35610c0 | 121 | 9 |
| comp36068c0 | 4823 | 9 |
| comp36197c0 | 1549 | 9 |
| comp43517c0 | 898 | 9 |
| comp44637c0 | 2280 | 9 |
| comp45060c0 | 820 | 9 |
| comp45303c0 | 3121 | 9 |
| comp45974c0 | 869 | 9 |
| comp472849c0 | 41 | 9 |
| comp51042c0 | 2274 | 9 |
| comp51463c0 | 1769 | 9 |
| comp51675c0 | 1558 | 9 |
| comp52383c0 | 168 | 9 |
| comp52606c0 | 1197 | 9 |
| comp52690c0 | 146 | 9 |
| comp52740c2 | 1753 | 9 |
| comp55130c0 | 238 | 9 |
| comp55193c2 | 75 | 9 |
| comp552330c0 | 1127 | 9 |
| comp55696c0 | 337 | 9 |
| comp56094c0 | 113 | 9 |
| comp56118c0 | 667 | 9 |
| comp56655c0 | 1407 | 9 |
| comp57323c0 | 1668 | 9 |
| comp57574c0 | 508 | 9 |
| comp57575c0 | 305 | 9 |
| comp57605c0 | 1544 | 9 |
| comp57621c0 | 71 | 9 |
| comp57651c0 | 221 | 9 |
| comp57786c1 | 1368 | 9 |
| comp58356c0 | 819 | 9 |
| comp58619c0 | 1972 | 9 |
| comp58699c0 | 4118 | 9 |
| comp58873c0 | 1273 | 9 |
| comp59111c0 | 201 | 9 |
| comp59319c0 | 320 | 9 |
| comp59373c0 | 1794 | 9 |
| comp60144c0 | 1813 | 9 |
| comp60529c0 | 339 | 9 |
| comp60832c0 | 3910 | 9 |
| comp60843c0 | 994 | 9 |
| comp60923c0 | 513 | 9 |
| comp61121c0 | 1420 | 9 |
| comp61453c0 | 748 | 9 |
| comp61505c0 | 1575 | 9 |
| comp61802c0 | 2783 | 9 |
| comp61928c0 | 239 | 9 |
| comp61980c0 | 804 | 9 |
| comp62325c0 | 564 | 9 |
| comp62671c0 | 431 | 9 |
| comp62919c0 | 431 | 9 |
| comp62920c0 | 928 | 9 |
| comp63370c0 | 1230 | 9 |
| comp63719c0 | 822 | 9 |
| comp63797c0 | 831 | 9 |
| comp63813c0 | 1205 | 9 |
| comp63887c0 | 69 | 9 |
| comp63911c1 | 578 | 9 |
| comp63964c0 | 1 | 9 |
| comp64592c2 | 166 | 9 |
| comp65287c0 | 1343 | 9 |
| comp65434c1 | 577 | 9 |
| comp65608c0 | 378 | 9 |
| comp65674c1 | 316 | 9 |
| comp65783c0 | 1316 | 9 |
| comp65977c0 | 108 | 9 |
| comp66015c0 | 940 | 9 |
| comp66148c0 | 1902 | 9 |
| comp6635c0 | 92 | 9 |
| comp66377c0 | 913 | 9 |
| comp66493c0 | 2436 | 9 |
| comp66833c0 | 2026 | 9 |
| comp66880c0 | 4211 | 9 |
| comp67228c0 | 1352 | 9 |
| comp67377c0 | 574 | 9 |
| comp67400c0 | 2038 | 9 |
| comp67681c0 | 973 | 9 |
| comp67733c0 | 2340 | 9 |
| comp67854c0 | 1364 | 9 |
| comp67941c0 | 525 | 9 |
| comp68061c0 | 1256 | 9 |
| comp68402c0 | 674 | 9 |
| comp68478c0 | 837 | 9 |
| comp68552c0 | 1080 | 9 |
| comp68653c1 | 3516 | 9 |
| comp68910c1 | 2758 | 9 |
| comp68997c0 | 1228 | 9 |
| comp68998c0 | 88 | 9 |
| comp69163c2 | 1215 | 9 |
| comp69204c1 | 3352 | 9 |
| comp69274c0 | 1652 | 9 |
| comp69289c0 | 946 | 9 |
| comp69468c0 | 2239 | 9 |
| comp69633c1 | 572 | 9 |
| comp69819c1 | 1351 | 9 |
| comp69821c0 | 247 | 9 |
| comp69885c0 | 2990 | 9 |
| comp69921c0 | 1210 | 9 |
| comp70166c0 | 897 | 9 |
| comp70264c0 | 1087 | 9 |
| comp70283c0 | 3967 | 9 |
| comp70610c0 | 1288 | 9 |
| comp70636c0 | 394 | 9 |
| comp70689c0 | 2061 | 9 |
| comp70798c2 | 3449 | 9 |
| comp70830c0 | 377 | 9 |
| comp70888c0 | 251 | 9 |
| comp71030c0 | 596 | 9 |
| comp71047c0 | 140 | 9 |
| comp71055c0 | 96 | 9 |
| comp71255c0 | 889 | 9 |
| comp71509c2 | 866 | 9 |
| comp71702c0 | 1813 | 9 |
| comp71874c0 | 1487 | 9 |
| comp71996c0 | 446 | 9 |
| comp72124c0 | 1930 | 9 |
| comp72138c3 | 1424 | 9 |
| comp72231c0 | 1153 | 9 |
| comp72249c0 | 642 | 9 |
| comp72335c5 | 2052 | 9 |
| comp72425c0 | 2091 | 9 |
| comp72684c0 | 32 | 9 |
| comp72696c0 | 2580 | 9 |
| comp72741c0 | 1996 | 9 |
| comp8290c0 | 933 | 9 |
| comp888865c0 | 1963 | 9 |
| comp1370472c0 | 642 | 8 |
| comp1792827c0 | 782 | 8 |
| comp2368c0 | 2026 | 8 |
| comp35641c0 | 118 | 8 |
| comp35725c0 | 1137 | 8 |
| comp35773c0 | 3486 | 8 |
| comp35898c0 | 235 | 8 |
| comp36007c0 | 2695 | 8 |
| comp36057c0 | 977 | 8 |
| comp36590c0 | 1379 | 8 |
| comp37066c0 | 1344 | 8 |
| comp42856c0 | 1016 | 8 |
| comp42885c0 | 2293 | 8 |
| comp42922c0 | 717 | 8 |
| comp43527c0 | 982 | 8 |
| comp43534c0 | 918 | 8 |
| comp43562c0 | 944 | 8 |
| comp43812c0 | 806 | 8 |
| comp43845c0 | 739 | 8 |
| comp44077c0 | 2775 | 8 |
| comp44189c0 | 402 | 8 |
| comp45117c0 | 442 | 8 |
| comp46061c0 | 144 | 8 |
| comp48842c0 | 156 | 8 |
| comp50344c0 | 915 | 8 |
| comp50991c0 | 1190 | 8 |
| comp51446c0 | 112 | 8 |
| comp51573c0 | 777 | 8 |
| comp51813c0 | 291 | 8 |
| comp52038c0 | 927 | 8 |
| comp53485c0 | 3133 | 8 |
| comp53806c0 | 1714 | 8 |
| comp53855c0 | 1349 | 8 |
| comp54206c0 | 331 | 8 |
| comp54493c0 | 511 | 8 |
| comp56139c0 | 666 | 8 |
| comp56317c0 | 2980 | 8 |
| comp56492c0 | 1651 | 8 |
| comp56571c0 | 313 | 8 |
| comp56762c0 | 1182 | 8 |
| comp56796c0 | 1152 | 8 |
| comp57057c1 | 75 | 8 |
| comp57061c0 | 469 | 8 |
| comp57141c0 | 423 | 8 |
| comp57335c0 | 1785 | 8 |
| comp57340c0 | 56 | 8 |
| comp57781c0 | 473 | 8 |
| comp57782c0 | 144 | 8 |
| comp57870c0 | 1791 | 8 |
| comp57929c0 | 530 | 8 |
| comp58251c0 | 1150 | 8 |
| comp58328c0 | 365 | 8 |
| comp58751c1 | 799 | 8 |
| comp58820c0 | 1123 | 8 |
| comp59296c0 | 183 | 8 |
| comp59627c0 | 338 | 8 |
| comp59674c0 | 357 | 8 |
| comp60114c2 | 1392 | 8 |
| comp60159c1 | 619 | 8 |
| comp60764c1 | 3531 | 8 |
| comp61040c0 | 2350 | 8 |
| comp61071c1 | 2187 | 8 |
| comp61227c0 | 1046 | 8 |
| comp61520c0 | 161 | 8 |
| comp61905c1 | 1952 | 8 |
| comp62021c0 | 437 | 8 |
| comp62670c0 | 417 | 8 |
| comp62814c0 | 4731 | 8 |
| comp62987c0 | 472 | 8 |
| comp63045c1 | 613 | 8 |
| comp63084c0 | 3409 | 8 |
| comp63219c0 | 818 | 8 |
| comp63296c0 | 1279 | 8 |
| comp63481c0 | 729 | 8 |
| comp63673c0 | 1179 | 8 |
| comp63835c0 | 4231 | 8 |
| comp63922c0 | 1342 | 8 |
| comp63956c0 | 1882 | 8 |
| comp64032c0 | 691 | 8 |
| comp64484c0 | 550 | 8 |
| comp64658c0 | 536 | 8 |
| comp64680c0 | 916 | 8 |
| comp64716c0 | 766 | 8 |
| comp64988c0 | 910 | 8 |
| comp65032c0 | 637 | 8 |
| comp65209c0 | 1173 | 8 |
| comp65539c0 | 718 | 8 |
| comp65550c0 | 1075 | 8 |
| comp65646c0 | 3485 | 8 |
| comp65819c5 | 40 | 8 |
| comp65968c0 | 808 | 8 |
| comp66159c0 | 2148 | 8 |
| comp66165c1 | 1511 | 8 |
| comp66477c0 | 479 | 8 |
| comp66634c0 | 1395 | 8 |
| comp66634c1 | 130 | 8 |
| comp66890c0 | 3375 | 8 |
| comp66978c0 | 263 | 8 |
| comp67060c0 | 280 | 8 |
| comp67326c0 | 4540 | 8 |
| comp67399c0 | 5068 | 8 |
| comp67416c0 | 1524 | 8 |
| comp67602c0 | 647 | 8 |
| comp67914c0 | 1450 | 8 |
| comp67938c0 | 832 | 8 |
| comp68039c0 | 775 | 8 |
| comp68064c6 | 1349 | 8 |
| comp68206c0 | 1007 | 8 |
| comp68365c0 | 6409 | 8 |
| comp68438c0 | 1101 | 8 |
| comp68521c0 | 158 | 8 |
| comp68702c0 | 242 | 8 |
| comp68712c5 | 2588 | 8 |
| comp68770c2 | 651 | 8 |
| comp68789c0 | 627 | 8 |
| comp68882c0 | 1950 | 8 |
| comp68914c0 | 506 | 8 |
| comp68968c0 | 1265 | 8 |
| comp69074c0 | 96 | 8 |
| comp69115c0 | 551 | 8 |
| comp69351c3 | 505 | 8 |
| comp69373c0 | 564 | 8 |
| comp69467c0 | 621 | 8 |
| comp69633c0 | 638 | 8 |
| comp69720c0 | 3895 | 8 |
| comp69801c0 | 455 | 8 |
| comp70007c0 | 73 | 8 |
| comp70122c0 | 335 | 8 |
| comp70216c0 | 1638 | 8 |
| comp70271c1 | 1079 | 8 |
| comp70309c0 | 2167 | 8 |
| comp70316c1 | 690 | 8 |
| comp70718c0 | 1494 | 8 |
| comp70798c1 | 1548 | 8 |
| comp70799c0 | 6076 | 8 |
| comp70818c0 | 1566 | 8 |
| comp70945c2 | 276 | 8 |
| comp71009c0 | 2844 | 8 |
| comp71079c0 | 311 | 8 |
| comp71090c0 | 1098 | 8 |
| comp71160c0 | 191 | 8 |
| comp71192c0 | 948 | 8 |
| comp71286c0 | 720 | 8 |
| comp71600c0 | 31 | 8 |
| comp71672c0 | 423 | 8 |
| comp71742c0 | 885 | 8 |
| comp71757c0 | 327 | 8 |
| comp71786c0 | 2003 | 8 |
| comp71804c0 | 820 | 8 |
| comp71827c0 | 308 | 8 |
| comp71863c0 | 3993 | 8 |
| comp71890c0 | 685 | 8 |
| comp71894c1 | 465 | 8 |
| comp71917c1 | 182 | 8 |
| comp71980c0 | 960 | 8 |
| comp71982c0 | 1985 | 8 |
| comp72009c0 | 564 | 8 |
| comp72226c0 | 373 | 8 |
| comp72273c0 | 603 | 8 |
| comp72367c0 | 405 | 8 |
| comp72385c0 | 233 | 8 |
| comp72494c0 | 2033 | 8 |
| comp72719c0 | 2238 | 8 |
| comp9097c0 | 2070 | 8 |
| comp23078c0 | 510 | 7 |
| comp248c0 | 105 | 7 |
| comp35620c0 | 164 | 7 |
| comp36107c0 | 255 | 7 |
| comp37074c0 | 246 | 7 |
| comp39739c0 | 250 | 7 |
| comp41556c0 | 71 | 7 |
| comp42846c0 | 2338 | 7 |
| comp43158c0 | 1286 | 7 |
| comp43567c0 | 498 | 7 |
| comp43720c0 | 574 | 7 |
| comp43993c0 | 495 | 7 |
| comp45176c0 | 1145 | 7 |
| comp45746c1 | 1167 | 7 |
| comp46035c0 | 1237 | 7 |
| comp47083c0 | 632 | 7 |
| comp47846c0 | 702 | 7 |
| comp49019c0 | 704 | 7 |
| comp51061c0 | 1237 | 7 |
| comp51318c0 | 709 | 7 |
| comp51458c0 | 2011 | 7 |
| comp51502c0 | 663 | 7 |
| comp52295c0 | 1313 | 7 |
| comp52688c1 | 3219 | 7 |
| comp52794c0 | 1505 | 7 |
| comp53232c0 | 539 | 7 |
| comp53414c0 | 378 | 7 |
| comp53917c1 | 210 | 7 |
| comp53983c0 | 1287 | 7 |
| comp544729c0 | 432 | 7 |
| comp54892c0 | 2111 | 7 |
| comp54921c0 | 329 | 7 |
| comp55276c0 | 654 | 7 |
| comp55619c0 | 1164 | 7 |
| comp55799c0 | 69 | 7 |
| comp55872c0 | 1677 | 7 |
| comp55979c1 | 48 | 7 |
| comp56156c0 | 111 | 7 |
| comp56256c0 | 303 | 7 |
| comp56425c0 | 680 | 7 |
| comp56697c0 | 5492 | 7 |
| comp56812c0 | 377 | 7 |
| comp57013c2 | 2072 | 7 |
| comp57102c0 | 316 | 7 |
| comp57290c0 | 507 | 7 |
| comp57337c0 | 868 | 7 |
| comp57361c0 | 1033 | 7 |
| comp58040c0 | 2084 | 7 |
| comp58071c0 | 552 | 7 |
| comp58293c0 | 182 | 7 |
| comp58295c0 | 1784 | 7 |
| comp58310c0 | 74 | 7 |
| comp58530c0 | 1472 | 7 |
| comp591515c0 | 680 | 7 |
| comp59193c0 | 1046 | 7 |
| comp59675c1 | 1156 | 7 |
| comp60002c0 | 1182 | 7 |
| comp60174c0 | 1468 | 7 |
| comp60302c1 | 99 | 7 |
| comp60712c0 | 1326 | 7 |
| comp61046c0 | 58633 | 7 |
| comp61125c0 | 1344 | 7 |
| comp61274c0 | 1350 | 7 |
| comp61416c0 | 2303 | 7 |
| comp61602c0 | 452 | 7 |
| comp61973c0 | 537 | 7 |
| comp61991c1 | 141 | 7 |
| comp62059c0 | 243 | 7 |
| comp62076c0 | 625 | 7 |
| comp62482c0 | 378 | 7 |
| comp62538c1 | 368 | 7 |
| comp62556c0 | 758 | 7 |
| comp62583c0 | 422 | 7 |
| comp62966c0 | 1309 | 7 |
| comp63253c0 | 784 | 7 |
| comp63535c0 | 118 | 7 |
| comp63664c0 | 340 | 7 |
| comp63789c0 | 1141 | 7 |
| comp64439c0 | 44 | 7 |
| comp64549c1 | 702 | 7 |
| comp64810c0 | 2913 | 7 |
| comp64895c0 | 118 | 7 |
| comp64961c0 | 1199 | 7 |
| comp65047c0 | 3356 | 7 |
| comp65197c0 | 89 | 7 |
| comp65233c1 | 14 | 7 |
| comp65251c5 | 344 | 7 |
| comp65276c0 | 265 | 7 |
| comp65290c0 | 1291 | 7 |
| comp65303c0 | 1391 | 7 |
| comp65373c0 | 1572 | 7 |
| comp65472c0 | 360 | 7 |
| comp65555c0 | 1072 | 7 |
| comp65659c1 | 540 | 7 |
| comp65675c0 | 1943 | 7 |
| comp65728c0 | 190 | 7 |
| comp65948c0 | 34 | 7 |
| comp66186c0 | 1517 | 7 |
| comp66208c0 | 1366 | 7 |
| comp66242c0 | 671 | 7 |
| comp66302c0 | 548 | 7 |
| comp66373c0 | 2144 | 7 |
| comp66663c0 | 1147 | 7 |
| comp66950c0 | 1068 | 7 |
| comp67019c0 | 942 | 7 |
| comp67059c0 | 1647 | 7 |
| comp67134c0 | 1050 | 7 |
| comp67491c0 | 332 | 7 |
| comp67587c0 | 71 | 7 |
| comp67617c0 | 529 | 7 |
| comp67677c0 | 1061 | 7 |
| comp67735c0 | 1210 | 7 |
| comp68009c0 | 478 | 7 |
| comp68060c0 | 172 | 7 |
| comp68084c0 | 3360 | 7 |
| comp68110c0 | 135 | 7 |
| comp68278c0 | 1838 | 7 |
| comp68300c0 | 359 | 7 |
| comp68381c0 | 1165 | 7 |
| comp68396c0 | 851 | 7 |
| comp68605c0 | 329 | 7 |
| comp68672c1 | 380 | 7 |
| comp68701c0 | 6860 | 7 |
| comp68711c1 | 3736 | 7 |
| comp68768c2 | 130 | 7 |
| comp68850c0 | 898 | 7 |
| comp68944c0 | 2013 | 7 |
| comp69034c0 | 100 | 7 |
| comp69058c0 | 1044 | 7 |
| comp69069c0 | 1863 | 7 |
| comp69166c0 | 1570 | 7 |
| comp69922c0 | 2928 | 7 |
| comp69924c0 | 1137 | 7 |
| comp69952c0 | 1070 | 7 |
| comp70046c0 | 4138 | 7 |
| comp70206c0 | 53 | 7 |
| comp70217c0 | 154 | 7 |
| comp70518c0 | 982 | 7 |
| comp70575c0 | 430 | 7 |
| comp70788c0 | 2254 | 7 |
| comp70850c0 | 231 | 7 |
| comp70966c0 | 1052 | 7 |
| comp71149c0 | 197 | 7 |
| comp71299c0 | 1869 | 7 |
| comp71329c0 | 221 | 7 |
| comp71354c0 | 3035 | 7 |
| comp71483c0 | 497 | 7 |
| comp71508c0 | 219 | 7 |
| comp71519c0 | 593 | 7 |
| comp71522c2 | 2464 | 7 |
| comp71582c0 | 759 | 7 |
| comp71710c0 | 1511 | 7 |
| comp71940c0 | 639 | 7 |
| comp72052c2 | 527 | 7 |
| comp72076c0 | 2003 | 7 |
| comp72104c1 | 1393 | 7 |
| comp72166c0 | 4636 | 7 |
| comp72245c0 | 622 | 7 |
| comp72359c0 | 982 | 7 |
| comp72384c1 | 1583 | 7 |
| comp72399c0 | 1461 | 7 |
| comp72418c0 | 759 | 7 |
| comp72512c0 | 1663 | 7 |
| comp72620c0 | 661 | 7 |
| comp72650c0 | 551 | 7 |
| comp9511c0 | 1060 | 7 |
| comp1005576c0 | 769 | 6 |
| comp10314c0 | 535 | 6 |
| comp1171391c0 | 1923 | 6 |
| comp123c0 | 1016 | 6 |
| comp131015c0 | 565 | 6 |
| comp1399474c0 | 898 | 6 |
| comp1444538c0 | 5493 | 6 |
| comp1894120c0 | 1221 | 6 |
| comp24559c0 | 795 | 6 |
| comp29367c0 | 253 | 6 |
| comp2951c0 | 1085 | 6 |
| comp31002c0 | 1449 | 6 |
| comp35274c0 | 1173 | 6 |
| comp35818c0 | 982 | 6 |
| comp35883c0 | 1567 | 6 |
| comp38650c0 | 942 | 6 |
| comp39045c0 | 2081 | 6 |
| comp41118c0 | 118 | 6 |
| comp41255c0 | 7203 | 6 |
| comp42725c0 | 319 | 6 |
| comp43734c0 | 681 | 6 |
| comp43941c0 | 158 | 6 |
| comp44177c0 | 326 | 6 |
| comp44965c0 | 291 | 6 |
| comp48151c0 | 247 | 6 |
| comp51073c0 | 772 | 6 |
| comp51317c0 | 32466 | 6 |
| comp51584c0 | 1341 | 6 |
| comp51589c0 | 1181 | 6 |
| comp51838c0 | 1446 | 6 |
| comp51846c0 | 707 | 6 |
| comp51918c0 | 586 | 6 |
| comp52006c0 | 1020 | 6 |
| comp52459c0 | 473 | 6 |
| comp52629c0 | 1688 | 6 |
| comp53074c0 | 653 | 6 |
| comp54721c0 | 1163 | 6 |
| comp54984c0 | 2423 | 6 |
| comp55193c1 | 86 | 6 |
| comp55295c0 | 291 | 6 |
| comp55310c1 | 342 | 6 |
| comp55391c0 | 393 | 6 |
| comp56036c0 | 1656 | 6 |
| comp56102c0 | 2412 | 6 |
| comp56160c0 | 22 | 6 |
| comp56403c0 | 49 | 6 |
| comp56517c0 | 1718 | 6 |
| comp56599c0 | 388 | 6 |
| comp56817c1 | 215 | 6 |
| comp57603c0 | 879 | 6 |
| comp57627c0 | 1102 | 6 |
| comp57695c0 | 648 | 6 |
| comp57866c0 | 368 | 6 |
| comp57996c0 | 11225 | 6 |
| comp58106c0 | 1024 | 6 |
| comp58128c0 | 816 | 6 |
| comp58233c0 | 1464 | 6 |
| comp58241c0 | 187 | 6 |
| comp58416c1 | 136 | 6 |
| comp58747c0 | 70 | 6 |
| comp58796c0 | 478 | 6 |
| comp58863c0 | 164 | 6 |
| comp59250c0 | 1021 | 6 |
| comp59375c0 | 675 | 6 |
| comp59399c0 | 1374 | 6 |
| comp59469c0 | 795 | 6 |
| comp59595c0 | 386 | 6 |
| comp59704c0 | 9 | 6 |
| comp59870c0 | 997 | 6 |
| comp59961c0 | 2363 | 6 |
| comp59982c0 | 1021 | 6 |
| comp60068c0 | 634 | 6 |
| comp60203c0 | 1711 | 6 |
| comp60244c0 | 1455 | 6 |
| comp60255c1 | 319 | 6 |
| comp60433c0 | 332 | 6 |
| comp60606c0 | 1588 | 6 |
| comp60824c0 | 159 | 6 |
| comp60929c0 | 967 | 6 |
| comp60986c0 | 50 | 6 |
| comp61166c0 | 272 | 6 |
| comp61398c0 | 245 | 6 |
| comp61411c0 | 623 | 6 |
| comp61786c0 | 1186 | 6 |
| comp61972c0 | 1507 | 6 |
| comp62037c0 | 26 | 6 |
| comp62258c0 | 747 | 6 |
| comp62354c0 | 1324 | 6 |
| comp625416c0 | 704 | 6 |
| comp62617c0 | 86 | 6 |
| comp62716c0 | 2857 | 6 |
| comp62757c0 | 560 | 6 |
| comp62773c0 | 691 | 6 |
| comp62844c0 | 1268 | 6 |
| comp62863c0 | 549 | 6 |
| comp62914c0 | 372 | 6 |
| comp62972c0 | 303 | 6 |
| comp63053c0 | 1204 | 6 |
| comp63277c0 | 678 | 6 |
| comp63278c0 | 1102 | 6 |
| comp63376c1 | 1353 | 6 |
| comp63554c0 | 1099 | 6 |
| comp63569c0 | 1927 | 6 |
| comp63706c0 | 222 | 6 |
| comp63734c0 | 704 | 6 |
| comp63769c0 | 1696 | 6 |
| comp63808c0 | 156 | 6 |
| comp63814c0 | 757 | 6 |
| comp638434c0 | 514 | 6 |
| comp63849c0 | 339 | 6 |
| comp63946c0 | 895 | 6 |
| comp64059c0 | 1944 | 6 |
| comp64080c0 | 2906 | 6 |
| comp64285c1 | 424 | 6 |
| comp64388c0 | 1118 | 6 |
| comp64442c0 | 2085 | 6 |
| comp64453c0 | 119 | 6 |
| comp64485c0 | 899 | 6 |
| comp64642c0 | 1215 | 6 |
| comp64899c0 | 696 | 6 |
| comp64908c0 | 875 | 6 |
| comp65021c0 | 860 | 6 |
| comp65087c0 | 395 | 6 |
| comp65275c0 | 3482 | 6 |
| comp65293c0 | 1089 | 6 |
| comp65312c0 | 686 | 6 |
| comp65446c0 | 4283 | 6 |
| comp65500c0 | 992 | 6 |
| comp65732c0 | 868 | 6 |
| comp65743c2 | 367 | 6 |
| comp65778c0 | 1126 | 6 |
| comp65864c0 | 926 | 6 |
| comp65876c0 | 3032 | 6 |
| comp65986c0 | 574 | 6 |
| comp65990c2 | 1348 | 6 |
| comp66025c0 | 858 | 6 |
| comp66141c0 | 773 | 6 |
| comp66142c1 | 116 | 6 |
| comp66204c0 | 1400 | 6 |
| comp66222c0 | 941 | 6 |
| comp66261c0 | 783 | 6 |
| comp66295c0 | 4 | 6 |
| comp66305c0 | 505 | 6 |
| comp66349c0 | 660 | 6 |
| comp66494c0 | 371 | 6 |
| comp66619c0 | 1199 | 6 |
| comp66695c0 | 624 | 6 |
| comp66706c0 | 930 | 6 |
| comp66783c0 | 453 | 6 |
| comp66806c0 | 62 | 6 |
| comp66831c0 | 1734 | 6 |
| comp66847c0 | 515 | 6 |
| comp66902c0 | 231 | 6 |
| comp66905c2 | 654 | 6 |
| comp66935c1 | 1549 | 6 |
| comp66945c0 | 648 | 6 |
| comp67021c0 | 58 | 6 |
| comp67257c0 | 854 | 6 |
| comp67440c0 | 253 | 6 |
| comp67457c0 | 2432 | 6 |
| comp67545c0 | 2440 | 6 |
| comp67565c0 | 2025 | 6 |
| comp67579c1 | 714 | 6 |
| comp67655c0 | 706 | 6 |
| comp67694c0 | 861 | 6 |
| comp67760c0 | 333 | 6 |
| comp67764c0 | 429 | 6 |
| comp68034c0 | 3366 | 6 |
| comp68120c0 | 190 | 6 |
| comp68135c0 | 617 | 6 |
| comp68165c0 | 3050 | 6 |
| comp68239c0 | 50 | 6 |
| comp68258c0 | 888 | 6 |
| comp68362c0 | 2540 | 6 |
| comp68411c0 | 2180 | 6 |
| comp68427c0 | 226 | 6 |
| comp68486c0 | 603 | 6 |
| comp68531c0 | 7321 | 6 |
| comp68535c1 | 185 | 6 |
| comp68567c0 | 540 | 6 |
| comp68612c0 | 499 | 6 |
| comp68726c1 | 892 | 6 |
| comp68744c0 | 110 | 6 |
| comp68827c0 | 499 | 6 |
| comp68983c0 | 854 | 6 |
| comp68984c0 | 541 | 6 |
| comp69037c1 | 338 | 6 |
| comp69150c0 | 238 | 6 |
| comp69437c0 | 2495 | 6 |
| comp69484c0 | 909 | 6 |
| comp69524c0 | 2058 | 6 |
| comp69534c0 | 3401 | 6 |
| comp69550c0 | 415 | 6 |
| comp69567c0 | 878 | 6 |
| comp69793c1 | 1078 | 6 |
| comp69849c0 | 500 | 6 |
| comp69878c0 | 555 | 6 |
| comp69896c0 | 974 | 6 |
| comp70002c0 | 750 | 6 |
| comp70077c0 | 153 | 6 |
| comp70230c0 | 84 | 6 |
| comp70344c0 | 1137 | 6 |
| comp70379c1 | 465 | 6 |
| comp70398c0 | 1574 | 6 |
| comp70469c0 | 1916 | 6 |
| comp70512c0 | 330 | 6 |
| comp70519c0 | 676 | 6 |
| comp70563c0 | 1038 | 6 |
| comp70604c1 | 771 | 6 |
| comp70738c0 | 306 | 6 |
| comp70790c0 | 2357 | 6 |
| comp70801c0 | 3177 | 6 |
| comp70844c0 | 1558 | 6 |
| comp70900c0 | 493 | 6 |
| comp70978c0 | 627 | 6 |
| comp71013c1 | 158 | 6 |
| comp71033c0 | 2489 | 6 |
| comp71035c0 | 1838 | 6 |
| comp71070c0 | 867 | 6 |
| comp711349c0 | 57 | 6 |
| comp71265c1 | 3531 | 6 |
| comp71303c0 | 457 | 6 |
| comp71355c0 | 988 | 6 |
| comp71449c1 | 579 | 6 |
| comp71450c0 | 1267 | 6 |
| comp71523c0 | 413 | 6 |
| comp71556c0 | 1066 | 6 |
| comp71774c0 | 301 | 6 |
| comp71855c0 | 2273 | 6 |
| comp72005c0 | 320 | 6 |
| comp72020c0 | 2094 | 6 |
| comp72099c0 | 4374 | 6 |
| comp72121c0 | 431 | 6 |
| comp72168c0 | 238 | 6 |
| comp72209c0 | 280 | 6 |
| comp72220c1 | 2049 | 6 |
| comp72235c0 | 1615 | 6 |
| comp72292c0 | 364 | 6 |
| comp72297c2 | 319 | 6 |
| comp72324c1 | 2338 | 6 |
| comp72341c0 | 648 | 6 |
| comp72521c0 | 796 | 6 |
| comp72550c0 | 1118 | 6 |
| comp72635c0 | 397 | 6 |
| comp72646c0 | 744 | 6 |
| comp72720c0 | 453 | 6 |
| comp73543c0 | 528 | 6 |
| comp8648c0 | 428 | 6 |
| comp873599c0 | 1663 | 6 |
| comp1073c1 | 381 | 5 |
| comp11620c0 | 1042 | 5 |
| comp150c0 | 1400 | 5 |
| comp1514884c0 | 663 | 5 |
| comp178c0 | 824 | 5 |
| comp23201c0 | 1283 | 5 |
| comp247887c0 | 1146 | 5 |
| comp29261c0 | 3632 | 5 |
| comp30759c0 | 571 | 5 |
| comp310661c0 | 95 | 5 |
| comp340047c0 | 985 | 5 |
| comp34830c0 | 603 | 5 |
| comp35393c0 | 1358 | 5 |
| comp35579c0 | 149 | 5 |
| comp38789c0 | 558 | 5 |
| comp43114c0 | 6352 | 5 |
| comp43517c1 | 482 | 5 |
| comp43590c1 | 622 | 5 |
| comp43782c0 | 149 | 5 |
| comp43864c0 | 533 | 5 |
| comp44082c0 | 247 | 5 |
| comp44147c0 | 571 | 5 |
| comp45124c0 | 619 | 5 |
| comp45922c0 | 824 | 5 |
| comp48272c0 | 676 | 5 |
| comp48521c0 | 1736 | 5 |
| comp49821c0 | 536 | 5 |
| comp50041c2 | 853 | 5 |
| comp50985c0 | 834 | 5 |
| comp51442c0 | 668 | 5 |
| comp51500c0 | 1336 | 5 |
| comp51697c0 | 2121 | 5 |
| comp52075c0 | 782 | 5 |
| comp52152c0 | 1321 | 5 |
| comp52292c0 | 130 | 5 |
| comp52349c1 | 324 | 5 |
| comp52527c0 | 1150 | 5 |
| comp52621c0 | 1023 | 5 |
| comp52656c0 | 613 | 5 |
| comp53105c0 | 99 | 5 |
| comp53847c0 | 507 | 5 |
| comp54916c0 | 1883 | 5 |
| comp55784c0 | 256 | 5 |
| comp55891c0 | 3668 | 5 |
| comp55944c0 | 197 | 5 |
| comp56170c0 | 473 | 5 |
| comp56287c0 | 149 | 5 |
| comp56514c0 | 75 | 5 |
| comp56800c0 | 1000 | 5 |
| comp56823c1 | 4036 | 5 |
| comp56877c1 | 865 | 5 |
| comp56960c0 | 281 | 5 |
| comp57057c0 | 266 | 5 |
| comp57560c0 | 527 | 5 |
| comp57811c0 | 1129 | 5 |
| comp57981c0 | 1397 | 5 |
| comp58250c0 | 299 | 5 |
| comp58361c0 | 1235 | 5 |
| comp58692c0 | 919 | 5 |
| comp58733c0 | 650 | 5 |
| comp58769c0 | 1507 | 5 |
| comp58941c0 | 1928 | 5 |
| comp59483c0 | 1923 | 5 |
| comp59698c0 | 755 | 5 |
| comp59760c0 | 3410 | 5 |
| comp59801c0 | 1477 | 5 |
| comp59894c0 | 137 | 5 |
| comp59996c0 | 450 | 5 |
| comp60044c0 | 1227 | 5 |
| comp60307c0 | 585 | 5 |
| comp60448c1 | 169 | 5 |
| comp60465c0 | 2588 | 5 |
| comp60803c0 | 131 | 5 |
| comp60937c0 | 970 | 5 |
| comp61013c0 | 479 | 5 |
| comp61023c0 | 1900 | 5 |
| comp61175c0 | 58 | 5 |
| comp61279c0 | 287 | 5 |
| comp61413c0 | 345 | 5 |
| comp61524c0 | 706 | 5 |
| comp61842c0 | 28 | 5 |
| comp61843c0 | 713 | 5 |
| comp61905c0 | 865 | 5 |
| comp61922c1 | 614 | 5 |
| comp62122c0 | 387 | 5 |
| comp62213c0 | 80 | 5 |
| comp62297c0 | 146 | 5 |
| comp62308c0 | 216 | 5 |
| comp62346c0 | 242 | 5 |
| comp62405c0 | 967 | 5 |
| comp62550c0 | 440 | 5 |
| comp62684c0 | 2334 | 5 |
| comp62727c0 | 261 | 5 |
| comp62821c0 | 2464 | 5 |
| comp63070c0 | 363 | 5 |
| comp63193c0 | 1969 | 5 |
| comp63206c0 | 661 | 5 |
| comp63316c3 | 946 | 5 |
| comp63392c0 | 487 | 5 |
| comp63584c0 | 155 | 5 |
| comp63603c0 | 1220 | 5 |
| comp63764c0 | 81 | 5 |
| comp63926c0 | 514 | 5 |
| comp64064c0 | 670 | 5 |
| comp64129c0 | 1829 | 5 |
| comp64207c0 | 1899 | 5 |
| comp64234c0 | 1009 | 5 |
| comp64378c0 | 968 | 5 |
| comp64446c0 | 205 | 5 |
| comp64615c0 | 536 | 5 |
| comp64631c0 | 472 | 5 |
| comp64645c0 | 1344 | 5 |
| comp64689c0 | 168 | 5 |
| comp64710c0 | 480 | 5 |
| comp64885c0 | 535 | 5 |
| comp64894c0 | 1087 | 5 |
| comp64931c0 | 81 | 5 |
| comp65026c0 | 1223 | 5 |
| comp65066c0 | 149 | 5 |
| comp65103c0 | 857 | 5 |
| comp65150c0 | 707 | 5 |
| comp65176c0 | 22 | 5 |
| comp65211c0 | 247 | 5 |
| comp65386c0 | 1185 | 5 |
| comp65440c0 | 306 | 5 |
| comp65452c0 | 1163 | 5 |
| comp65474c0 | 486 | 5 |
| comp65891c2 | 4711 | 5 |
| comp66012c0 | 777 | 5 |
| comp66142c0 | 310 | 5 |
| comp66260c0 | 338 | 5 |
| comp66265c0 | 1671 | 5 |
| comp66275c0 | 65 | 5 |
| comp66275c1 | 63 | 5 |
| comp66367c0 | 59 | 5 |
| comp66451c0 | 168 | 5 |
| comp66482c0 | 159 | 5 |
| comp66526c0 | 527 | 5 |
| comp66588c0 | 1489 | 5 |
| comp66626c0 | 1223 | 5 |
| comp66678c0 | 631 | 5 |
| comp66699c0 | 183 | 5 |
| comp66741c0 | 869 | 5 |
| comp66822c1 | 15 | 5 |
| comp6683c0 | 51750 | 5 |
| comp66856c1 | 52 | 5 |
| comp66905c0 | 436 | 5 |
| comp66917c1 | 639 | 5 |
| comp6716c0 | 186 | 5 |
| comp67375c0 | 687 | 5 |
| comp67390c0 | 734 | 5 |
| comp67406c0 | 1405 | 5 |
| comp67504c0 | 204 | 5 |
| comp67594c0 | 848 | 5 |
| comp67610c0 | 673 | 5 |
| comp67627c0 | 549 | 5 |
| comp67650c0 | 1225 | 5 |
| comp67654c0 | 1225 | 5 |
| comp67752c0 | 753 | 5 |
| comp67791c0 | 643 | 5 |
| comp67827c0 | 589 | 5 |
| comp67831c0 | 121 | 5 |
| comp67846c0 | 721 | 5 |
| comp67858c1 | 528 | 5 |
| comp67894c0 | 1169 | 5 |
| comp67915c0 | 1406 | 5 |
| comp68041c0 | 3348 | 5 |
| comp68053c0 | 204 | 5 |
| comp68523c0 | 1466 | 5 |
| comp68525c0 | 505 | 5 |
| comp68708c0 | 357 | 5 |
| comp68819c0 | 3437 | 5 |
| comp68852c0 | 427 | 5 |
| comp68864c0 | 625 | 5 |
| comp68931c1 | 1963 | 5 |
| comp68990c1 | 2872 | 5 |
| comp69096c0 | 350 | 5 |
| comp69137c0 | 6148 | 5 |
| comp69190c0 | 889 | 5 |
| comp69207c0 | 1060 | 5 |
| comp69257c0 | 1919 | 5 |
| comp69262c0 | 351 | 5 |
| comp69368c0 | 78 | 5 |
| comp69445c1 | 493 | 5 |
| comp69494c0 | 1590 | 5 |
| comp69514c0 | 197 | 5 |
| comp69672c3 | 1186 | 5 |
| comp69678c0 | 1533 | 5 |
| comp69705c0 | 846 | 5 |
| comp69826c0 | 371 | 5 |
| comp69960c0 | 470 | 5 |
| comp70044c0 | 483 | 5 |
| comp70220c0 | 346 | 5 |
| comp70276c0 | 1299 | 5 |
| comp70389c0 | 2706 | 5 |
| comp70389c1 | 2496 | 5 |
| comp70406c0 | 953 | 5 |
| comp70532c0 | 953 | 5 |
| comp70589c0 | 34616 | 5 |
| comp70622c0 | 830 | 5 |
| comp70628c0 | 3244 | 5 |
| comp70651c0 | 1361 | 5 |
| comp70819c0 | 596 | 5 |
| comp70837c0 | 763 | 5 |
| comp70841c0 | 1180 | 5 |
| comp70845c0 | 886 | 5 |
| comp71046c0 | 1083 | 5 |
| comp71057c0 | 926 | 5 |
| comp71081c0 | 11097 | 5 |
| comp71116c0 | 169 | 5 |
| comp71128c0 | 1197 | 5 |
| comp71133c2 | 295 | 5 |
| comp71146c0 | 771 | 5 |
| comp71162c0 | 2368 | 5 |
| comp71180c0 | 1516 | 5 |
| comp71249c0 | 738 | 5 |
| comp71261c0 | 505 | 5 |
| comp71398c2 | 1483 | 5 |
| comp71440c0 | 1023 | 5 |
| comp71442c0 | 956 | 5 |
| comp71482c0 | 561 | 5 |
| comp71513c0 | 938 | 5 |
| comp71564c0 | 880 | 5 |
| comp71643c0 | 785 | 5 |
| comp71662c0 | 715 | 5 |
| comp71681c0 | 834 | 5 |
| comp71901c0 | 186 | 5 |
| comp71914c0 | 495 | 5 |
| comp71964c0 | 3818 | 5 |
| comp71975c0 | 2563 | 5 |
| comp71992c0 | 645 | 5 |
| comp72046c0 | 2563 | 5 |
| comp72050c0 | 264 | 5 |
| comp72157c0 | 1361 | 5 |
| comp72220c0 | 859 | 5 |
| comp72285c0 | 179 | 5 |
| comp72489c0 | 2013 | 5 |
| comp72508c0 | 1292 | 5 |
| comp72518c0 | 602 | 5 |
| comp72561c1 | 1015 | 5 |
| comp72572c0 | 394 | 5 |
| comp72610c0 | 1077 | 5 |
| comp72647c0 | 1199 | 5 |
| comp72661c0 | 1639 | 5 |
| comp72695c0 | 2672 | 5 |
| comp72724c0 | 1071 | 5 |
| comp73067c0 | 503 | 5 |
| comp75521c0 | 654 | 5 |
| comp788395c0 | 315 | 5 |
| comp978318c0 | 1154 | 5 |
| comp1081207c0 | 138 | 4 |
| comp1148062c0 | 246 | 4 |
| comp1197548c0 | 277 | 4 |
| comp1383283c0 | 1821 | 4 |
| comp154c0 | 62 | 4 |
| comp1732451c0 | 1506 | 4 |
| comp17555c0 | 1252 | 4 |
| comp21260c0 | 1055 | 4 |
| comp2263868c0 | 653 | 4 |
| comp23575c0 | 1358 | 4 |
| comp23671c0 | 406 | 4 |
| comp2432187c0 | 990 | 4 |
| comp2472024c0 | 831 | 4 |
| comp256774c0 | 1227 | 4 |
| comp33c0 | 447 | 4 |
| comp34079c0 | 713 | 4 |
| comp34105c0 | 601 | 4 |
| comp35402c0 | 453 | 4 |
| comp35491c0 | 312 | 4 |
| comp35681c0 | 127 | 4 |
| comp35749c0 | 130 | 4 |
| comp35836c0 | 849 | 4 |
| comp35871c0 | 748 | 4 |
| comp36037c0 | 653 | 4 |
| comp36081c0 | 4504 | 4 |
| comp36098c0 | 1269 | 4 |
| comp36380c0 | 806 | 4 |
| comp37709c0 | 1135 | 4 |
| comp39653c0 | 87 | 4 |
| comp41292c0 | 351 | 4 |
| comp41409c0 | 609 | 4 |
| comp41671c0 | 407 | 4 |
| comp41746c0 | 2340 | 4 |
| comp42391c0 | 1057 | 4 |
| comp42511c0 | 93 | 4 |
| comp42876c0 | 1666 | 4 |
| comp43408c0 | 1141 | 4 |
| comp43436c0 | 1112 | 4 |
| comp43479c0 | 66 | 4 |
| comp43541c0 | 188 | 4 |
| comp43619c0 | 731 | 4 |
| comp43790c0 | 395 | 4 |
| comp43836c0 | 1990 | 4 |
| comp43984c0 | 357 | 4 |
| comp44003c1 | 504 | 4 |
| comp44088c0 | 1176 | 4 |
| comp44179c0 | 203 | 4 |
| comp44181c0 | 83 | 4 |
| comp44199c1 | 600 | 4 |
| comp44340c0 | 1085 | 4 |
| comp44724c0 | 1397 | 4 |
| comp44896c0 | 1782 | 4 |
| comp44988c0 | 725 | 4 |
| comp45101c0 | 1105 | 4 |
| comp45188c0 | 319 | 4 |
| comp45214c0 | 3412 | 4 |
| comp45274c1 | 347 | 4 |
| comp45465c1 | 388 | 4 |
| comp45755c0 | 676 | 4 |
| comp45991c0 | 1246 | 4 |
| comp47155c0 | 11770 | 4 |
| comp47264c0 | 423 | 4 |
| comp47305c0 | 1235 | 4 |
| comp47305c1 | 820 | 4 |
| comp47771c0 | 731 | 4 |
| comp48557c0 | 429 | 4 |
| comp48712c0 | 366 | 4 |
| comp49054c0 | 438 | 4 |
| comp49504c0 | 1646 | 4 |
| comp50474c0 | 437 | 4 |
| comp50500c1 | 1004 | 4 |
| comp50662c0 | 188 | 4 |
| comp51021c0 | 561 | 4 |
| comp51196c0 | 1119 | 4 |
| comp51375c0 | 370 | 4 |
| comp51441c0 | 281 | 4 |
| comp51466c0 | 744 | 4 |
| comp51557c0 | 478 | 4 |
| comp51566c0 | 492 | 4 |
| comp51646c0 | 202 | 4 |
| comp51683c0 | 1030 | 4 |
| comp51750c0 | 1539 | 4 |
| comp51857c0 | 1099 | 4 |
| comp51979c0 | 135 | 4 |
| comp51992c0 | 1808 | 4 |
| comp52096c0 | 447 | 4 |
| comp52440c0 | 44 | 4 |
| comp52440c1 | 730 | 4 |
| comp52495c0 | 1416 | 4 |
| comp52571c0 | 1169 | 4 |
| comp52581c0 | 773 | 4 |
| comp52583c0 | 444 | 4 |
| comp52625c0 | 1455 | 4 |
| comp527055c0 | 409 | 4 |
| comp52710c0 | 267 | 4 |
| comp52772c0 | 470 | 4 |
| comp52891c0 | 424 | 4 |
| comp52962c0 | 717 | 4 |
| comp52992c0 | 133 | 4 |
| comp53339c1 | 1689 | 4 |
| comp53768c0 | 1196 | 4 |
| comp53817c1 | 6 | 4 |
| comp53977c0 | 1634 | 4 |
| comp54210c0 | 651 | 4 |
| comp54309c0 | 1274 | 4 |
| comp54616c0 | 997 | 4 |
| comp54711c0 | 1310 | 4 |
| comp55046c0 | 875 | 4 |
| comp55065c0 | 873 | 4 |
| comp55099c0 | 281 | 4 |
| comp55281c0 | 2731 | 4 |
| comp55549c0 | 1908 | 4 |
| comp55584c0 | 401 | 4 |
| comp55843c0 | 1429 | 4 |
| comp55873c0 | 2705 | 4 |
| comp56091c0 | 531 | 4 |
| comp56106c0 | 1759 | 4 |
| comp56168c0 | 1144 | 4 |
| comp56178c0 | 77 | 4 |
| comp56205c0 | 269 | 4 |
| comp56254c0 | 1739 | 4 |
| comp56283c0 | 1190 | 4 |
| comp56299c0 | 1158 | 4 |
| comp56513c0 | 315 | 4 |
| comp56582c0 | 237 | 4 |
| comp56638c0 | 507 | 4 |
| comp56659c0 | 433 | 4 |
| comp56714c0 | 1293 | 4 |
| comp56888c1 | 1210 | 4 |
| comp57234c0 | 265 | 4 |
| comp57240c1 | 541 | 4 |
| comp57357c0 | 1291 | 4 |
| comp57369c3 | 106 | 4 |
| comp57451c1 | 1080 | 4 |
| comp57513c0 | 559 | 4 |
| comp57576c0 | 2655 | 4 |
| comp57604c0 | 52 | 4 |
| comp57631c0 | 3111 | 4 |
| comp57714c0 | 977 | 4 |
| comp57727c0 | 376 | 4 |
| comp57935c1 | 2277 | 4 |
| comp58026c0 | 748 | 4 |
| comp58120c0 | 473 | 4 |
| comp58214c0 | 954 | 4 |
| comp58262c0 | 623 | 4 |
| comp58318c0 | 615 | 4 |
| comp58598c0 | 597 | 4 |
| comp58678c0 | 294 | 4 |
| comp58746c0 | 1611 | 4 |
| comp58788c0 | 358 | 4 |
| comp58801c1 | 117 | 4 |
| comp58833c0 | 2009 | 4 |
| comp58862c0 | 1601 | 4 |
| comp58901c0 | 730 | 4 |
| comp58928c1 | 746 | 4 |
| comp58951c0 | 991 | 4 |
| comp59013c0 | 1209 | 4 |
| comp59053c0 | 1825 | 4 |
| comp59070c0 | 306 | 4 |
| comp59078c0 | 1805 | 4 |
| comp59218c0 | 1126 | 4 |
| comp59271c1 | 2977 | 4 |
| comp59318c0 | 262 | 4 |
| comp59331c0 | 667 | 4 |
| comp59458c0 | 863 | 4 |
| comp59504c0 | 293 | 4 |
| comp59521c0 | 461 | 4 |
| comp59570c0 | 649 | 4 |
| comp59585c0 | 602 | 4 |
| comp59733c1 | 1162 | 4 |
| comp59959c0 | 2468 | 4 |
| comp59978c0 | 677 | 4 |
| comp60030c1 | 27 | 4 |
| comp60164c0 | 603 | 4 |
| comp60208c0 | 965 | 4 |
| comp60220c3 | 111 | 4 |
| comp60330c0 | 978 | 4 |
| comp60376c1 | 176 | 4 |
| comp60391c0 | 515 | 4 |
| comp60393c0 | 495 | 4 |
| comp60409c0 | 2380 | 4 |
| comp60420c0 | 814 | 4 |
| comp60420c1 | 599 | 4 |
| comp60470c0 | 68 | 4 |
| comp60525c0 | 161 | 4 |
| comp60541c0 | 202 | 4 |
| comp60611c0 | 54 | 4 |
| comp60633c0 | 569 | 4 |
| comp60640c0 | 646 | 4 |
| comp60650c1 | 712 | 4 |
| comp61019c1 | 675 | 4 |
| comp61052c0 | 377 | 4 |
| comp61055c0 | 1192 | 4 |
| comp61055c1 | 0 | 4 |
| comp61100c0 | 647 | 4 |
| comp61104c0 | 1459 | 4 |
| comp61128c0 | 1606 | 4 |
| comp61167c0 | 597 | 4 |
| comp61176c0 | 484 | 4 |
| comp61338c0 | 1729 | 4 |
| comp61431c1 | 1376 | 4 |
| comp61440c0 | 581 | 4 |
| comp61569c0 | 726 | 4 |
| comp61667c0 | 346 | 4 |
| comp61696c0 | 564 | 4 |
| comp61712c0 | 2530 | 4 |
| comp61827c1 | 1784 | 4 |
| comp61982c0 | 577 | 4 |
| comp62131c0 | 532 | 4 |
| comp621759c0 | 1076 | 4 |
| comp62184c0 | 298 | 4 |
| comp62206c0 | 325 | 4 |
| comp62261c0 | 780 | 4 |
| comp62349c0 | 1174 | 4 |
| comp62428c0 | 281 | 4 |
| comp62429c0 | 785 | 4 |
| comp62435c1 | 166 | 4 |
| comp62462c3 | 1042 | 4 |
| comp62489c1 | 106 | 4 |
| comp62497c0 | 416 | 4 |
| comp62516c0 | 621 | 4 |
| comp62528c0 | 1241 | 4 |
| comp62572c0 | 1409 | 4 |
| comp62676c0 | 1236 | 4 |
| comp62677c0 | 886 | 4 |
| comp62688c0 | 1240 | 4 |
| comp62728c0 | 1272 | 4 |
| comp62779c0 | 293 | 4 |
| comp62782c0 | 166 | 4 |
| comp62818c1 | 1262 | 4 |
| comp62855c1 | 206 | 4 |
| comp62899c0 | 113 | 4 |
| comp62977c0 | 564 | 4 |
| comp62997c0 | 569 | 4 |
| comp63004c0 | 1657 | 4 |
| comp63021c0 | 471 | 4 |
| comp63029c0 | 712 | 4 |
| comp63098c0 | 1148 | 4 |
| comp63113c0 | 905 | 4 |
| comp63137c3 | 371 | 4 |
| comp63201c0 | 2667 | 4 |
| comp63221c0 | 1076 | 4 |
| comp63231c0 | 213 | 4 |
| comp63266c0 | 4587 | 4 |
| comp63330c0 | 711 | 4 |
| comp63361c0 | 763 | 4 |
| comp63364c1 | 2489 | 4 |
| comp63382c0 | 46 | 4 |
| comp63385c0 | 77 | 4 |
| comp63451c0 | 144 | 4 |
| comp63486c0 | 434 | 4 |
| comp63497c0 | 492 | 4 |
| comp63525c0 | 763 | 4 |
| comp63532c1 | 712 | 4 |
| comp63556c0 | 58 | 4 |
| comp63577c0 | 2206 | 4 |
| comp63588c0 | 713 | 4 |
| comp63701c0 | 989 | 4 |
| comp63710c0 | 1451 | 4 |
| comp63806c0 | 212 | 4 |
| comp63869c0 | 197 | 4 |
| comp64054c0 | 1516 | 4 |
| comp64118c0 | 275 | 4 |
| comp64120c0 | 1225 | 4 |
| comp64146c0 | 1831 | 4 |
| comp64179c0 | 2788 | 4 |
| comp64220c0 | 1506 | 4 |
| comp64224c0 | 839 | 4 |
| comp64240c0 | 1156 | 4 |
| comp64283c1 | 360 | 4 |
| comp64291c1 | 915 | 4 |
| comp64377c0 | 445 | 4 |
| comp64391c0 | 613 | 4 |
| comp64435c0 | 644 | 4 |
| comp64491c0 | 2185 | 4 |
| comp64494c0 | 928 | 4 |
| comp64510c0 | 1716 | 4 |
| comp64632c0 | 513 | 4 |
| comp64654c0 | 3628 | 4 |
| comp64664c0 | 737 | 4 |
| comp64678c0 | 543 | 4 |
| comp64725c0 | 455 | 4 |
| comp64762c0 | 1881 | 4 |
| comp64763c0 | 519 | 4 |
| comp64806c0 | 733 | 4 |
| comp64832c0 | 582 | 4 |
| comp64843c0 | 105 | 4 |
| comp64859c0 | 741 | 4 |
| comp64881c0 | 638 | 4 |
| comp64958c0 | 286 | 4 |
| comp64997c0 | 26 | 4 |
| comp65056c0 | 340 | 4 |
| comp65077c0 | 1062 | 4 |
| comp65114c0 | 2828 | 4 |
| comp65124c0 | 73 | 4 |
| comp65143c0 | 764 | 4 |
| comp65170c0 | 521 | 4 |
| comp65213c0 | 246 | 4 |
| comp65218c0 | 922 | 4 |
| comp65354c1 | 263 | 4 |
| comp65375c0 | 800 | 4 |
| comp65404c0 | 2816 | 4 |
| comp65476c0 | 654 | 4 |
| comp65478c0 | 357 | 4 |
| comp65494c0 | 246 | 4 |
| comp65531c0 | 276 | 4 |
| comp65549c0 | 2663 | 4 |
| comp65576c0 | 157 | 4 |
| comp65626c0 | 724 | 4 |
| comp65712c0 | 2922 | 4 |
| comp65732c1 | 1019 | 4 |
| comp65739c0 | 1878 | 4 |
| comp65768c0 | 550 | 4 |
| comp65797c0 | 487 | 4 |
| comp65801c0 | 1255 | 4 |
| comp65804c0 | 348 | 4 |
| comp65807c1 | 2997 | 4 |
| comp65813c0 | 1747 | 4 |
| comp65827c0 | 470 | 4 |
| comp65833c1 | 709 | 4 |
| comp65847c0 | 671 | 4 |
| comp65889c0 | 1831 | 4 |
| comp65915c0 | 2232 | 4 |
| comp65951c0 | 1313 | 4 |
| comp65954c1 | 8532 | 4 |
| comp65971c0 | 972 | 4 |
| comp66003c0 | 631 | 4 |
| comp66008c0 | 887 | 4 |
| comp66019c0 | 158 | 4 |
| comp66033c0 | 2296 | 4 |
| comp66050c0 | 1287 | 4 |
| comp66065c0 | 477 | 4 |
| comp66073c0 | 2302 | 4 |
| comp66074c1 | 1475 | 4 |
| comp66079c0 | 1480 | 4 |
| comp66080c1 | 1679 | 4 |
| comp66120c0 | 506 | 4 |
| comp66147c0 | 451 | 4 |
| comp66151c0 | 142 | 4 |
| comp66220c0 | 346 | 4 |
| comp66252c0 | 1372 | 4 |
| comp66382c2 | 1927 | 4 |
| comp66428c0 | 321 | 4 |
| comp66470c0 | 107 | 4 |
| comp66481c2 | 1803 | 4 |
| comp66506c0 | 1275 | 4 |
| comp66548c0 | 877 | 4 |
| comp66676c0 | 2121 | 4 |
| comp66698c0 | 379 | 4 |
| comp66718c0 | 642 | 4 |
| comp66724c0 | 1553 | 4 |
| comp66739c0 | 542 | 4 |
| comp66760c0 | 3532 | 4 |
| comp66765c0 | 16 | 4 |
| comp66765c1 | 3372 | 4 |
| comp66786c1 | 490 | 4 |
| comp66860c0 | 546 | 4 |
| comp66913c0 | 1094 | 4 |
| comp66938c1 | 501 | 4 |
| comp67036c0 | 974 | 4 |
| comp67082c0 | 300 | 4 |
| comp67119c0 | 139 | 4 |
| comp67142c0 | 1282 | 4 |
| comp67152c0 | 300 | 4 |
| comp67198c0 | 842 | 4 |
| comp67202c0 | 102 | 4 |
| comp67215c0 | 976 | 4 |
| comp67282c0 | 1519 | 4 |
| comp67285c0 | 183 | 4 |
| comp67301c0 | 1115 | 4 |
| comp67307c0 | 1189 | 4 |
| comp67327c0 | 544 | 4 |
| comp67361c0 | 1356 | 4 |
| comp67370c0 | 1427 | 4 |
| comp67396c0 | 452 | 4 |
| comp67525c0 | 435 | 4 |
| comp67541c0 | 435 | 4 |
| comp67556c0 | 538 | 4 |
| comp67603c0 | 1395 | 4 |
| comp67608c0 | 664 | 4 |
| comp67631c0 | 236 | 4 |
| comp67652c0 | 593 | 4 |
| comp67692c0 | 936 | 4 |
| comp67746c0 | 277 | 4 |
| comp67754c0 | 243 | 4 |
| comp67788c0 | 563 | 4 |
| comp67803c0 | 553 | 4 |
| comp67805c0 | 1100 | 4 |
| comp67813c0 | 183 | 4 |
| comp67814c0 | 165 | 4 |
| comp67815c0 | 1153 | 4 |
| comp67838c1 | 97 | 4 |
| comp68001c0 | 1115 | 4 |
| comp68044c0 | 677 | 4 |
| comp68047c0 | 372 | 4 |
| comp68049c0 | 854 | 4 |
| comp68064c0 | 761 | 4 |
| comp68124c0 | 408 | 4 |
| comp68146c0 | 291 | 4 |
| comp68159c0 | 1603 | 4 |
| comp68173c0 | 197 | 4 |
| comp68187c0 | 1549 | 4 |
| comp68213c0 | 1050 | 4 |
| comp68226c0 | 451 | 4 |
| comp68228c0 | 1431 | 4 |
| comp68344c0 | 2537 | 4 |
| comp68366c0 | 1824 | 4 |
| comp68389c0 | 990 | 4 |
| comp68419c0 | 1155 | 4 |
| comp68424c0 | 831 | 4 |
| comp68515c0 | 548 | 4 |
| comp68536c0 | 1346 | 4 |
| comp68607c0 | 1073 | 4 |
| comp68712c4 | 607 | 4 |
| comp68767c0 | 576 | 4 |
| comp68826c0 | 131 | 4 |
| comp68828c0 | 1093 | 4 |
| comp68858c0 | 443 | 4 |
| comp68940c0 | 1465 | 4 |
| comp68955c0 | 982 | 4 |
| comp68987c0 | 905 | 4 |
| comp69014c0 | 467 | 4 |
| comp69039c0 | 1251 | 4 |
| comp69139c0 | 400 | 4 |
| comp69179c0 | 287 | 4 |
| comp69185c0 | 1362 | 4 |
| comp69203c0 | 17 | 4 |
| comp69256c0 | 390 | 4 |
| comp69263c1 | 2540 | 4 |
| comp69301c0 | 531 | 4 |
| comp69310c1 | 318 | 4 |
| comp69314c0 | 552 | 4 |
| comp69316c0 | 2331 | 4 |
| comp69326c0 | 289 | 4 |
| comp69415c0 | 1140 | 4 |
| comp69433c0 | 1221 | 4 |
| comp69435c0 | 576 | 4 |
| comp69520c0 | 1265 | 4 |
| comp69541c0 | 33 | 4 |
| comp69560c0 | 399 | 4 |
| comp69563c1 | 541 | 4 |
| comp69565c1 | 505 | 4 |
| comp69584c0 | 319 | 4 |
| comp69592c0 | 964 | 4 |
| comp69611c0 | 784 | 4 |
| comp69619c0 | 1914 | 4 |
| comp69650c0 | 702 | 4 |
| comp69663c0 | 849 | 4 |
| comp69708c0 | 976 | 4 |
| comp69715c0 | 359 | 4 |
| comp69717c0 | 730 | 4 |
| comp69732c1 | 2290 | 4 |
| comp69762c0 | 218 | 4 |
| comp69778c0 | 3003 | 4 |
| comp69792c0 | 2814 | 4 |
| comp69870c0 | 583 | 4 |
| comp69888c0 | 993 | 4 |
| comp69897c0 | 3410 | 4 |
| comp69915c0 | 1745 | 4 |
| comp69968c0 | 805 | 4 |
| comp69985c0 | 471 | 4 |
| comp70040c0 | 384 | 4 |
| comp70076c0 | 157 | 4 |
| comp70084c0 | 1220 | 4 |
| comp70091c0 | 1123 | 4 |
| comp70092c0 | 495 | 4 |
| comp70142c3 | 939 | 4 |
| comp70192c0 | 212 | 4 |
| comp70231c0 | 66 | 4 |
| comp70262c0 | 1054 | 4 |
| comp70311c0 | 4350 | 4 |
| comp70314c0 | 919 | 4 |
| comp70361c0 | 701 | 4 |
| comp70423c0 | 2798 | 4 |
| comp70436c0 | 683 | 4 |
| comp70543c0 | 1614 | 4 |
| comp70546c0 | 574 | 4 |
| comp70655c1 | 141 | 4 |
| comp70684c0 | 1154 | 4 |
| comp70693c0 | 854 | 4 |
| comp70765c0 | 414 | 4 |
| comp70780c0 | 624 | 4 |
| comp70785c2 | 639 | 4 |
| comp70821c0 | 138 | 4 |
| comp70874c0 | 1025 | 4 |
| comp70893c2 | 699 | 4 |
| comp70967c0 | 1605 | 4 |
| comp70999c2 | 1574 | 4 |
| comp71042c0 | 168 | 4 |
| comp71044c0 | 1036 | 4 |
| comp71093c0 | 873 | 4 |
| comp71136c0 | 2287 | 4 |
| comp71141c0 | 1229 | 4 |
| comp71152c0 | 53 | 4 |
| comp71227c0 | 1686 | 4 |
| comp71258c0 | 640 | 4 |
| comp71276c0 | 155 | 4 |
| comp71291c0 | 2490 | 4 |
| comp71305c0 | 1850 | 4 |
| comp71326c0 | 61 | 4 |
| comp71372c0 | 769 | 4 |
| comp71393c0 | 365 | 4 |
| comp71425c1 | 1317 | 4 |
| comp71427c0 | 1749 | 4 |
| comp71463c0 | 143 | 4 |
| comp71476c0 | 727 | 4 |
| comp71497c0 | 2651 | 4 |
| comp71600c1 | 263 | 4 |
| comp71617c0 | 222 | 4 |
| comp71652c0 | 980 | 4 |
| comp71655c0 | 257 | 4 |
| comp71683c0 | 536 | 4 |
| comp71694c0 | 628 | 4 |
| comp71698c2 | 1117 | 4 |
| comp71738c0 | 842 | 4 |
| comp71747c0 | 1378 | 4 |
| comp71810c0 | 1608 | 4 |
| comp71824c0 | 1126 | 4 |
| comp71828c0 | 1298 | 4 |
| comp71829c0 | 222 | 4 |
| comp71831c0 | 1245 | 4 |
| comp71845c0 | 1710 | 4 |
| comp71857c0 | 690 | 4 |
| comp71869c0 | 1073 | 4 |
| comp71873c0 | 717 | 4 |
| comp71945c0 | 1253 | 4 |
| comp71962c0 | 3448 | 4 |
| comp71978c0 | 1438 | 4 |
| comp71986c1 | 1041 | 4 |
| comp72007c0 | 285 | 4 |
| comp72110c0 | 374 | 4 |
| comp72114c0 | 229 | 4 |
| comp72154c1 | 1213 | 4 |
| comp72161c1 | 996 | 4 |
| comp72197c0 | 357 | 4 |
| comp72225c1 | 920 | 4 |
| comp72272c0 | 344 | 4 |
| comp72275c0 | 166 | 4 |
| comp72299c0 | 889 | 4 |
| comp72336c1 | 1171 | 4 |
| comp72450c0 | 2158 | 4 |
| comp72455c0 | 164 | 4 |
| comp72456c0 | 1058 | 4 |
| comp72487c0 | 368 | 4 |
| comp72519c0 | 990 | 4 |
| comp72541c0 | 297 | 4 |
| comp72570c1 | 1998 | 4 |
| comp72583c0 | 969 | 4 |
| comp72590c0 | 1098 | 4 |
| comp72595c1 | 437 | 4 |
| comp72616c0 | 1926 | 4 |
| comp72685c0 | 1183 | 4 |
| comp72694c1 | 560 | 4 |
| comp72699c1 | 2273 | 4 |
| comp72722c0 | 393 | 4 |
| comp72730c0 | 1725 | 4 |
| comp72750c0 | 4997 | 4 |
| comp72781c0 | 1307 | 4 |
| comp757138c0 | 391 | 4 |
| comp80062c0 | 1455 | 4 |
| comp82047c0 | 209 | 4 |
| comp823111c0 | 583 | 4 |
| comp83717c0 | 318 | 4 |
| comp887409c0 | 1250 | 4 |
| comp998679c0 | 351 | 4 |
| comp1026778c0 | 215 | 3 |
| comp1155948c0 | 550 | 3 |
| comp1314554c0 | 102 | 3 |
| comp1401600c0 | 107 | 3 |
| comp1558c0 | 2004 | 3 |
| comp1844995c0 | 1134 | 3 |
| comp2267445c0 | 128 | 3 |
| comp26008c0 | 6097 | 3 |
| comp2714236c0 | 255 | 3 |
| comp28073c0 | 63 | 3 |
| comp3425930c0 | 1107 | 3 |
| comp34492c0 | 665 | 3 |
| comp35318c0 | 497 | 3 |
| comp35330c0 | 1783 | 3 |
| comp35393c1 | 70 | 3 |
| comp35523c0 | 1161 | 3 |
| comp35552c0 | 67 | 3 |
| comp35719c0 | 660 | 3 |
| comp35771c0 | 746 | 3 |
| comp35781c0 | 984 | 3 |
| comp36065c0 | 1045 | 3 |
| comp36333c0 | 289 | 3 |
| comp38438c0 | 936 | 3 |
| comp39668c0 | 1131 | 3 |
| comp40024c0 | 686 | 3 |
| comp40364c0 | 196 | 3 |
| comp40491c0 | 678 | 3 |
| comp42901c0 | 574 | 3 |
| comp43004c0 | 914 | 3 |
| comp43037c0 | 574 | 3 |
| comp43287c0 | 627 | 3 |
| comp43358c0 | 53 | 3 |
| comp43522c0 | 747 | 3 |
| comp43594c0 | 579 | 3 |
| comp43595c0 | 773 | 3 |
| comp43609c0 | 524 | 3 |
| comp43675c0 | 805 | 3 |
| comp43916c0 | 696 | 3 |
| comp44100c0 | 512 | 3 |
| comp44121c0 | 90 | 3 |
| comp44128c0 | 546 | 3 |
| comp44194c0 | 972 | 3 |
| comp44323c0 | 1008 | 3 |
| comp44405c1 | 13 | 3 |
| comp44614c0 | 778 | 3 |
| comp44909c0 | 1368 | 3 |
| comp45136c0 | 9 | 3 |
| comp45379c0 | 667 | 3 |
| comp46078c0 | 1127 | 3 |
| comp46581c0 | 844 | 3 |
| comp47171c0 | 1078 | 3 |
| comp47400c0 | 1007 | 3 |
| comp47818c0 | 418 | 3 |
| comp48272c1 | 2153 | 3 |
| comp48589c0 | 738 | 3 |
| comp48645c0 | 265 | 3 |
| comp48910c0 | 686 | 3 |
| comp50416c0 | 239 | 3 |
| comp50678c0 | 1025 | 3 |
| comp506879c0 | 344 | 3 |
| comp51015c0 | 181 | 3 |
| comp51300c0 | 340 | 3 |
| comp51364c0 | 375 | 3 |
| comp51498c0 | 772 | 3 |
| comp51649c0 | 163 | 3 |
| comp51680c0 | 868 | 3 |
| comp51684c0 | 914 | 3 |
| comp51688c0 | 944 | 3 |
| comp51751c0 | 1122 | 3 |
| comp51807c0 | 142 | 3 |
| comp51949c0 | 1660 | 3 |
| comp51994c0 | 578 | 3 |
| comp52029c0 | 245 | 3 |
| comp52248c0 | 96 | 3 |
| comp52369c0 | 1303 | 3 |
| comp52528c0 | 634 | 3 |
| comp52607c0 | 830 | 3 |
| comp52726c0 | 990 | 3 |
| comp52841c0 | 223 | 3 |
| comp52872c0 | 176 | 3 |
| comp53003c0 | 578 | 3 |
| comp53146c0 | 202 | 3 |
| comp53308c2 | 326 | 3 |
| comp53337c1 | 338 | 3 |
| comp53371c0 | 356 | 3 |
| comp53402c0 | 477 | 3 |
| comp53446c1 | 516 | 3 |
| comp535894c0 | 508 | 3 |
| comp53600c0 | 417 | 3 |
| comp53637c0 | 594 | 3 |
| comp54340c0 | 1183 | 3 |
| comp54467c0 | 714 | 3 |
| comp54535c0 | 1171 | 3 |
| comp54791c1 | 898 | 3 |
| comp54816c0 | 1866 | 3 |
| comp55033c0 | 370 | 3 |
| comp55147c0 | 2888 | 3 |
| comp55172c0 | 329 | 3 |
| comp55257c0 | 381 | 3 |
| comp55420c0 | 4858 | 3 |
| comp55604c0 | 1699 | 3 |
| comp55611c1 | 14 | 3 |
| comp55661c0 | 2608 | 3 |
| comp55776c0 | 427 | 3 |
| comp55948c1 | 185 | 3 |
| comp55951c0 | 108 | 3 |
| comp55981c0 | 821 | 3 |
| comp56107c0 | 1145 | 3 |
| comp56119c0 | 284 | 3 |
| comp56132c0 | 586 | 3 |
| comp56244c0 | 115 | 3 |
| comp56253c0 | 389 | 3 |
| comp56360c0 | 1564 | 3 |
| comp56460c0 | 500 | 3 |
| comp56485c0 | 999 | 3 |
| comp56513c1 | 2256 | 3 |
| comp56578c0 | 1091 | 3 |
| comp56716c0 | 1014 | 3 |
| comp56784c0 | 524 | 3 |
| comp56802c0 | 659 | 3 |
| comp56817c0 | 385 | 3 |
| comp56908c0 | 216 | 3 |
| comp57121c0 | 498 | 3 |
| comp57129c0 | 28 | 3 |
| comp57148c0 | 610 | 3 |
| comp57186c0 | 1124 | 3 |
| comp57239c1 | 530 | 3 |
| comp57282c0 | 2513 | 3 |
| comp57391c0 | 625 | 3 |
| comp57394c0 | 349 | 3 |
| comp57427c0 | 364 | 3 |
| comp57433c0 | 1224 | 3 |
| comp57527c0 | 326 | 3 |
| comp57538c0 | 1118 | 3 |
| comp57621c1 | 133 | 3 |
| comp57674c0 | 496 | 3 |
| comp57697c0 | 935 | 3 |
| comp57704c0 | 528 | 3 |
| comp57729c0 | 569 | 3 |
| comp57745c0 | 386 | 3 |
| comp57759c1 | 3175 | 3 |
| comp57775c0 | 702 | 3 |
| comp57797c0 | 1227 | 3 |
| comp57885c0 | 1165 | 3 |
| comp57893c0 | 1092 | 3 |
| comp57906c0 | 1517 | 3 |
| comp57952c0 | 774 | 3 |
| comp57959c0 | 1006 | 3 |
| comp57975c0 | 863 | 3 |
| comp58048c0 | 2255 | 3 |
| comp58112c0 | 835 | 3 |
| comp58167c0 | 1669 | 3 |
| comp58282c0 | 1206 | 3 |
| comp58353c1 | 2665 | 3 |
| comp58383c0 | 498 | 3 |
| comp58403c0 | 453 | 3 |
| comp58488c0 | 3359 | 3 |
| comp58504c0 | 868 | 3 |
| comp58527c0 | 897 | 3 |
| comp58568c0 | 383 | 3 |
| comp58597c0 | 681 | 3 |
| comp58621c0 | 657 | 3 |
| comp58623c0 | 995 | 3 |
| comp58760c1 | 760 | 3 |
| comp58776c0 | 178 | 3 |
| comp58897c0 | 1156 | 3 |
| comp58989c0 | 194 | 3 |
| comp59023c0 | 969 | 3 |
| comp59037c0 | 891 | 3 |
| comp59069c0 | 1316 | 3 |
| comp59090c0 | 85 | 3 |
| comp59174c0 | 195 | 3 |
| comp59223c0 | 633 | 3 |
| comp59300c0 | 235 | 3 |
| comp59357c0 | 1189 | 3 |
| comp59372c0 | 592 | 3 |
| comp59413c0 | 702 | 3 |
| comp59418c0 | 69 | 3 |
| comp59461c0 | 1244 | 3 |
| comp59464c0 | 396 | 3 |
| comp59579c0 | 616 | 3 |
| comp59709c0 | 2084 | 3 |
| comp59718c0 | 10 | 3 |
| comp59725c0 | 96 | 3 |
| comp59730c0 | 104 | 3 |
| comp59783c0 | 601 | 3 |
| comp59795c0 | 419 | 3 |
| comp59811c0 | 602 | 3 |
| comp59864c0 | 489 | 3 |
| comp60162c0 | 1553 | 3 |
| comp60228c0 | 506 | 3 |
| comp60272c0 | 308 | 3 |
| comp60290c0 | 338 | 3 |
| comp60303c0 | 2276 | 3 |
| comp60414c0 | 660 | 3 |
| comp60489c0 | 705 | 3 |
| comp60513c1 | 334 | 3 |
| comp60596c0 | 1518 | 3 |
| comp60617c0 | 1596 | 3 |
| comp60630c0 | 675 | 3 |
| comp60645c0 | 302 | 3 |
| comp60662c0 | 530 | 3 |
| comp60670c0 | 1434 | 3 |
| comp60696c0 | 658 | 3 |
| comp60715c1 | 2332 | 3 |
| comp60717c0 | 992 | 3 |
| comp60807c0 | 1669 | 3 |
| comp60810c1 | 88 | 3 |
| comp60834c0 | 928 | 3 |
| comp60839c0 | 22 | 3 |
| comp60849c2 | 4 | 3 |
| comp60850c0 | 261 | 3 |
| comp60853c0 | 460 | 3 |
| comp60912c0 | 195 | 3 |
| comp60950c0 | 345 | 3 |
| comp61011c1 | 183 | 3 |
| comp61051c0 | 808 | 3 |
| comp61068c0 | 153 | 3 |
| comp61103c0 | 240 | 3 |
| comp61123c0 | 189 | 3 |
| comp61132c0 | 676 | 3 |
| comp61150c0 | 997 | 3 |
| comp61152c0 | 845 | 3 |
| comp61156c0 | 857 | 3 |
| comp61166c1 | 381 | 3 |
| comp61169c1 | 114 | 3 |
| comp61184c0 | 591 | 3 |
| comp61188c0 | 285 | 3 |
| comp61196c0 | 1050 | 3 |
| comp61250c0 | 1693 | 3 |
| comp61313c0 | 979 | 3 |
| comp61324c0 | 1064 | 3 |
| comp61350c0 | 656 | 3 |
| comp61365c0 | 565 | 3 |
| comp61473c0 | 923 | 3 |
| comp61489c2 | 692 | 3 |
| comp61515c0 | 638 | 3 |
| comp61563c0 | 1582 | 3 |
| comp61611c3 | 1063 | 3 |
| comp618690c0 | 192 | 3 |
| comp61890c0 | 940 | 3 |
| comp61925c0 | 1123 | 3 |
| comp61930c0 | 3881 | 3 |
| comp61975c0 | 274 | 3 |
| comp61987c0 | 2060 | 3 |
| comp61991c0 | 1540 | 3 |
| comp62035c0 | 857 | 3 |
| comp62043c1 | 1332 | 3 |
| comp62112c0 | 516 | 3 |
| comp62121c0 | 296 | 3 |
| comp62147c0 | 339 | 3 |
| comp62178c1 | 714 | 3 |
| comp62186c0 | 1390 | 3 |
| comp62280c0 | 148 | 3 |
| comp62299c0 | 462 | 3 |
| comp62322c0 | 1032 | 3 |
| comp62329c0 | 454 | 3 |
| comp62334c0 | 210 | 3 |
| comp62449c0 | 589 | 3 |
| comp62465c0 | 1258 | 3 |
| comp62479c0 | 1225 | 3 |
| comp62504c0 | 1294 | 3 |
| comp62526c0 | 1891 | 3 |
| comp62545c0 | 1284 | 3 |
| comp62567c0 | 56 | 3 |
| comp62618c0 | 243 | 3 |
| comp62661c1 | 113 | 3 |
| comp62662c0 | 659 | 3 |
| comp62818c0 | 171 | 3 |
| comp62829c0 | 761 | 3 |
| comp62842c0 | 542 | 3 |
| comp62908c0 | 500 | 3 |
| comp62952c0 | 436 | 3 |
| comp62980c0 | 1951 | 3 |
| comp63011c0 | 559 | 3 |
| comp63033c0 | 291 | 3 |
| comp63133c0 | 32 | 3 |
| comp63249c1 | 143 | 3 |
| comp63269c0 | 17 | 3 |
| comp63303c0 | 674 | 3 |
| comp63321c0 | 1561 | 3 |
| comp63324c0 | 389 | 3 |
| comp63371c0 | 143 | 3 |
| comp63440c0 | 345 | 3 |
| comp63466c0 | 732 | 3 |
| comp63475c1 | 1451 | 3 |
| comp63477c0 | 687 | 3 |
| comp63494c0 | 774 | 3 |
| comp63535c1 | 278 | 3 |
| comp63572c0 | 525 | 3 |
| comp63614c0 | 2353 | 3 |
| comp63634c0 | 529 | 3 |
| comp63665c0 | 195 | 3 |
| comp63676c0 | 549 | 3 |
| comp63746c0 | 648 | 3 |
| comp63841c0 | 694 | 3 |
| comp63872c0 | 10550 | 3 |
| comp63911c0 | 364 | 3 |
| comp63930c0 | 734 | 3 |
| comp63936c0 | 716 | 3 |
| comp63957c1 | 123 | 3 |
| comp63989c0 | 128 | 3 |
| comp64002c0 | 1708 | 3 |
| comp64113c1 | 505 | 3 |
| comp64124c0 | 746 | 3 |
| comp64144c1 | 1309 | 3 |
| comp64147c0 | 34 | 3 |
| comp64168c0 | 1079 | 3 |
| comp64177c0 | 875 | 3 |
| comp64209c0 | 460 | 3 |
| comp64213c0 | 483 | 3 |
| comp64216c0 | 801 | 3 |
| comp64218c4 | 3414 | 3 |
| comp64228c0 | 2284 | 3 |
| comp64255c0 | 1979 | 3 |
| comp64267c0 | 70 | 3 |
| comp64267c1 | 202 | 3 |
| comp64269c0 | 2258 | 3 |
| comp64389c0 | 161 | 3 |
| comp64447c0 | 192 | 3 |
| comp64538c0 | 518 | 3 |
| comp64546c0 | 1226 | 3 |
| comp64549c0 | 2592 | 3 |
| comp64553c0 | 807 | 3 |
| comp64563c0 | 2110 | 3 |
| comp64580c0 | 378 | 3 |
| comp64620c0 | 1485 | 3 |
| comp64669c0 | 1408 | 3 |
| comp64737c0 | 683 | 3 |
| comp64749c0 | 714 | 3 |
| comp64774c0 | 596 | 3 |
| comp64776c0 | 635 | 3 |
| comp64791c0 | 837 | 3 |
| comp64865c0 | 1021 | 3 |
| comp64886c0 | 807 | 3 |
| comp64934c0 | 917 | 3 |
| comp64996c0 | 1026 | 3 |
| comp65020c0 | 276 | 3 |
| comp65034c0 | 1024 | 3 |
| comp65060c0 | 1431 | 3 |
| comp65092c0 | 3319 | 3 |
| comp65141c0 | 298 | 3 |
| comp65189c0 | 723 | 3 |
| comp65229c0 | 166 | 3 |
| comp65231c0 | 1088 | 3 |
| comp65286c0 | 2003 | 3 |
| comp65325c0 | 268 | 3 |
| comp65330c0 | 1432 | 3 |
| comp65361c1 | 788 | 3 |
| comp65399c0 | 199 | 3 |
| comp65408c0 | 731 | 3 |
| comp65464c0 | 638 | 3 |
| comp65508c0 | 978 | 3 |
| comp65521c0 | 126 | 3 |
| comp65535c0 | 1064 | 3 |
| comp65569c0 | 836 | 3 |
| comp65609c0 | 1890 | 3 |
| comp65633c0 | 200 | 3 |
| comp65634c0 | 762 | 3 |
| comp65664c0 | 448 | 3 |
| comp65687c0 | 357 | 3 |
| comp65741c0 | 1152 | 3 |
| comp65748c0 | 106 | 3 |
| comp65767c1 | 694 | 3 |
| comp65786c0 | 1748 | 3 |
| comp65810c0 | 530 | 3 |
| comp65887c0 | 103 | 3 |
| comp65920c0 | 686 | 3 |
| comp65939c0 | 252 | 3 |
| comp65940c0 | 1302 | 3 |
| comp65953c0 | 907 | 3 |
| comp66017c1 | 191 | 3 |
| comp66046c0 | 1762 | 3 |
| comp66052c0 | 666 | 3 |
| comp66106c0 | 414 | 3 |
| comp66122c0 | 1344 | 3 |
| comp66149c1 | 2371 | 3 |
| comp66171c0 | 822 | 3 |
| comp66180c0 | 925 | 3 |
| comp66207c0 | 590 | 3 |
| comp66211c0 | 345 | 3 |
| comp66223c0 | 711 | 3 |
| comp66286c0 | 1047 | 3 |
| comp66292c0 | 28 | 3 |
| comp66311c0 | 1522 | 3 |
| comp66322c0 | 687 | 3 |
| comp66385c0 | 559 | 3 |
| comp66410c0 | 1610 | 3 |
| comp66440c0 | 77 | 3 |
| comp66446c0 | 479 | 3 |
| comp66448c0 | 983 | 3 |
| comp66466c0 | 38 | 3 |
| comp66535c0 | 426 | 3 |
| comp66542c0 | 271 | 3 |
| comp66557c0 | 1341 | 3 |
| comp66573c0 | 188 | 3 |
| comp66601c4 | 1913 | 3 |
| comp66605c0 | 1531 | 3 |
| comp66614c1 | 39 | 3 |
| comp66615c0 | 658 | 3 |
| comp66646c2 | 736 | 3 |
| comp66679c0 | 237 | 3 |
| comp66705c0 | 1202 | 3 |
| comp66725c0 | 1759 | 3 |
| comp66729c0 | 760 | 3 |
| comp66746c0 | 522 | 3 |
| comp66793c0 | 3001 | 3 |
| comp66805c0 | 106 | 3 |
| comp66815c0 | 147 | 3 |
| comp66823c0 | 411 | 3 |
| comp66825c0 | 1398 | 3 |
| comp66848c1 | 714 | 3 |
| comp66865c0 | 191 | 3 |
| comp66900c0 | 102 | 3 |
| comp66989c0 | 663 | 3 |
| comp67049c1 | 880 | 3 |
| comp67081c0 | 588 | 3 |
| comp67116c0 | 608 | 3 |
| comp67130c6 | 435 | 3 |
| comp67137c0 | 1181 | 3 |
| comp67140c0 | 1080 | 3 |
| comp67143c0 | 269 | 3 |
| comp67219c1 | 270 | 3 |
| comp67225c0 | 826 | 3 |
| comp67264c0 | 843 | 3 |
| comp67269c0 | 2227 | 3 |
| comp67300c0 | 629 | 3 |
| comp67342c1 | 1276 | 3 |
| comp67379c0 | 863 | 3 |
| comp67394c0 | 637 | 3 |
| comp67419c0 | 158 | 3 |
| comp67441c0 | 509 | 3 |
| comp67474c0 | 1306 | 3 |
| comp67485c2 | 253 | 3 |
| comp67499c0 | 1102 | 3 |
| comp67509c1 | 251 | 3 |
| comp67583c0 | 1913 | 3 |
| comp67591c0 | 28 | 3 |
| comp67625c0 | 353 | 3 |
| comp67718c0 | 103 | 3 |
| comp67906c0 | 1575 | 3 |
| comp67925c0 | 2110 | 3 |
| comp67961c0 | 886 | 3 |
| comp67965c0 | 234 | 3 |
| comp67983c0 | 335 | 3 |
| comp68028c0 | 628 | 3 |
| comp68052c0 | 995 | 3 |
| comp68134c0 | 729 | 3 |
| comp68175c0 | 11 | 3 |
| comp68201c1 | 594 | 3 |
| comp68275c0 | 93 | 3 |
| comp68283c0 | 785 | 3 |
| comp68311c0 | 248 | 3 |
| comp68334c0 | 389 | 3 |
| comp68461c0 | 117 | 3 |
| comp68472c0 | 115 | 3 |
| comp68544c0 | 1362 | 3 |
| comp68629c0 | 988 | 3 |
| comp68636c0 | 1611 | 3 |
| comp68647c0 | 42 | 3 |
| comp68683c0 | 203 | 3 |
| comp68687c0 | 693 | 3 |
| comp68712c1 | 601 | 3 |
| comp68723c1 | 855 | 3 |
| comp68756c0 | 1089 | 3 |
| comp68771c0 | 190 | 3 |
| comp68842c0 | 328 | 3 |
| comp68867c0 | 815 | 3 |
| comp68873c0 | 330 | 3 |
| comp68892c0 | 133 | 3 |
| comp68907c0 | 388 | 3 |
| comp68981c0 | 1568 | 3 |
| comp69043c1 | 1570 | 3 |
| comp69089c0 | 1434 | 3 |
| comp69103c1 | 87 | 3 |
| comp69195c0 | 1541 | 3 |
| comp69199c0 | 114 | 3 |
| comp69213c0 | 609 | 3 |
| comp69259c0 | 130 | 3 |
| comp69307c0 | 345 | 3 |
| comp69325c0 | 660 | 3 |
| comp693c1 | 512 | 3 |
| comp69413c0 | 496 | 3 |
| comp69443c0 | 450 | 3 |
| comp69495c0 | 3329 | 3 |
| comp69522c0 | 85 | 3 |
| comp69531c1 | 669 | 3 |
| comp69544c0 | 1385 | 3 |
| comp69552c0 | 151 | 3 |
| comp69574c0 | 848 | 3 |
| comp69583c0 | 168 | 3 |
| comp69686c0 | 232 | 3 |
| comp69838c0 | 1796 | 3 |
| comp69887c0 | 243 | 3 |
| comp69899c0 | 165 | 3 |
| comp69957c0 | 654 | 3 |
| comp69977c1 | 780 | 3 |
| comp69994c0 | 474 | 3 |
| comp69998c0 | 1421 | 3 |
| comp70068c0 | 148 | 3 |
| comp70103c0 | 928 | 3 |
| comp70170c0 | 554 | 3 |
| comp70202c0 | 687 | 3 |
| comp70275c0 | 511 | 3 |
| comp70333c0 | 665 | 3 |
| comp70347c0 | 440 | 3 |
| comp70499c0 | 703 | 3 |
| comp70567c0 | 276 | 3 |
| comp70579c0 | 80 | 3 |
| comp70630c0 | 391 | 3 |
| comp70631c0 | 526 | 3 |
| comp70669c0 | 621 | 3 |
| comp70672c0 | 79 | 3 |
| comp70706c1 | 3936 | 3 |
| comp70719c0 | 255 | 3 |
| comp70747c0 | 4035 | 3 |
| comp70793c0 | 457 | 3 |
| comp70959c0 | 240 | 3 |
| comp71002c0 | 148 | 3 |
| comp71025c2 | 351 | 3 |
| comp71066c0 | 1467 | 3 |
| comp71101c0 | 55 | 3 |
| comp71129c0 | 1875 | 3 |
| comp71211c0 | 1307 | 3 |
| comp71225c0 | 302 | 3 |
| comp71250c1 | 576 | 3 |
| comp71300c0 | 3065 | 3 |
| comp71309c1 | 941 | 3 |
| comp71319c0 | 777 | 3 |
| comp71384c0 | 779 | 3 |
| comp71415c0 | 85 | 3 |
| comp71421c0 | 201 | 3 |
| comp71459c0 | 363 | 3 |
| comp71484c0 | 996 | 3 |
| comp71490c0 | 351 | 3 |
| comp71494c0 | 262 | 3 |
| comp71509c3 | 1197 | 3 |
| comp71609c3 | 1005 | 3 |
| comp71653c0 | 181 | 3 |
| comp71667c0 | 263 | 3 |
| comp71706c0 | 265 | 3 |
| comp71784c0 | 1249 | 3 |
| comp71815c0 | 494 | 3 |
| comp71883c0 | 1951 | 3 |
| comp71891c0 | 802 | 3 |
| comp71898c0 | 870 | 3 |
| comp71949c0 | 1065 | 3 |
| comp71973c1 | 2561 | 3 |
| comp72021c0 | 212 | 3 |
| comp72031c0 | 876 | 3 |
| comp72039c0 | 1579 | 3 |
| comp72062c0 | 796 | 3 |
| comp72136c0 | 1024 | 3 |
| comp72161c0 | 2549 | 3 |
| comp72254c0 | 242 | 3 |
| comp72303c0 | 242 | 3 |
| comp72305c1 | 541 | 3 |
| comp72323c0 | 3008 | 3 |
| comp72348c0 | 303 | 3 |
| comp72363c0 | 631 | 3 |
| comp72381c0 | 207 | 3 |
| comp72404c0 | 1204 | 3 |
| comp72407c0 | 830 | 3 |
| comp72499c0 | 253 | 3 |
| comp72578c0 | 1815 | 3 |
| comp72579c0 | 135 | 3 |
| comp72594c0 | 640 | 3 |
| comp72618c0 | 627 | 3 |
| comp72665c0 | 891 | 3 |
| comp72835c0 | 1065 | 3 |
| comp73266c0 | 367 | 3 |
| comp73606c0 | 725 | 3 |
| comp74320c0 | 727 | 3 |
| comp74488c0 | 594 | 3 |
| comp74745c0 | 283 | 3 |
| comp74827c0 | 489 | 3 |
| comp82051c0 | 222 | 3 |
| comp83793c0 | 644 | 3 |
| comp853817c0 | 337 | 3 |
| comp8571c0 | 2222 | 3 |
| comp983642c0 | 233 | 3 |
| comp100343c0 | 4863 | 2 |
| comp102235c0 | 2182 | 2 |
| comp104946c0 | 924 | 2 |
| comp10585c0 | 184 | 2 |
| comp1085468c0 | 1175 | 2 |
| comp1137149c0 | 1565 | 2 |
| comp11395c0 | 1193 | 2 |
| comp1148084c0 | 19 | 2 |
| comp1150c0 | 140 | 2 |
| comp1169143c0 | 729 | 2 |
| comp1172108c0 | 1172 | 2 |
| comp1176734c0 | 824 | 2 |
| comp1227392c0 | 498 | 2 |
| comp1236c0 | 22 | 2 |
| comp12383c0 | 1303 | 2 |
| comp1288c0 | 759 | 2 |
| comp1364521c0 | 80 | 2 |
| comp1368889c0 | 1686 | 2 |
| comp13887c0 | 573 | 2 |
| comp1401432c0 | 1285 | 2 |
| comp1401533c0 | 551 | 2 |
| comp1410321c0 | 485 | 2 |
| comp1462956c0 | 808 | 2 |
| comp1564429c0 | 662 | 2 |
| comp1611c0 | 1084 | 2 |
| comp1636190c0 | 949 | 2 |
| comp16720c0 | 1191 | 2 |
| comp1673c0 | 588 | 2 |
| comp1749125c0 | 138 | 2 |
| comp1876001c0 | 557 | 2 |
| comp19224c0 | 233 | 2 |
| comp1937c0 | 1079 | 2 |
| comp19447c0 | 541 | 2 |
| comp200150c0 | 462 | 2 |
| comp20610c0 | 490 | 2 |
| comp20774c0 | 1941 | 2 |
| comp21496c1 | 999 | 2 |
| comp21796c0 | 80 | 2 |
| comp2182c0 | 881 | 2 |
| comp2182c1 | 861 | 2 |
| comp22537c0 | 494 | 2 |
| comp227215c0 | 34 | 2 |
| comp2274186c0 | 1060 | 2 |
| comp23245c0 | 29 | 2 |
| comp2331279c0 | 642 | 2 |
| comp23704c0 | 36 | 2 |
| comp23805c0 | 1343 | 2 |
| comp2593c0 | 7 | 2 |
| comp25956c0 | 267 | 2 |
| comp270045c0 | 1387 | 2 |
| comp2711622c0 | 653 | 2 |
| comp27408c0 | 355 | 2 |
| comp2769c0 | 1889 | 2 |
| comp2826c0 | 242 | 2 |
| comp29473c0 | 820 | 2 |
| comp29637c0 | 277 | 2 |
| comp2969c0 | 0 | 2 |
| comp3033c0 | 2657 | 2 |
| comp30926c0 | 609 | 2 |
| comp314806c0 | 2179 | 2 |
| comp32475c0 | 378 | 2 |
| comp331110c0 | 446 | 2 |
| comp34229c0 | 1289 | 2 |
| comp34233c0 | 871 | 2 |
| comp34283c0 | 170 | 2 |
| comp34387c0 | 2559 | 2 |
| comp34602c0 | 21 | 2 |
| comp34718c0 | 403 | 2 |
| comp35230c0 | 719 | 2 |
| comp35234c0 | 1141 | 2 |
| comp35244c0 | 1048 | 2 |
| comp35256c0 | 903 | 2 |
| comp35271c0 | 584 | 2 |
| comp35280c0 | 1173 | 2 |
| comp35285c0 | 1498 | 2 |
| comp35294c0 | 1573 | 2 |
| comp35295c0 | 364 | 2 |
| comp35314c0 | 1538 | 2 |
| comp35315c0 | 630 | 2 |
| comp35316c0 | 594 | 2 |
| comp35372c0 | 143 | 2 |
| comp35382c0 | 351 | 2 |
| comp35417c0 | 1373 | 2 |
| comp35425c0 | 713 | 2 |
| comp35429c0 | 876 | 2 |
| comp35440c0 | 1053 | 2 |
| comp35457c0 | 221 | 2 |
| comp35461c0 | 330 | 2 |
| comp35470c0 | 714 | 2 |
| comp35471c0 | 426 | 2 |
| comp35472c0 | 393 | 2 |
| comp35483c0 | 1157 | 2 |
| comp35487c0 | 423 | 2 |
| comp35494c0 | 703 | 2 |
| comp35500c0 | 1102 | 2 |
| comp35511c0 | 642 | 2 |
| comp35512c0 | 354 | 2 |
| comp35515c0 | 232 | 2 |
| comp35518c0 | 720 | 2 |
| comp35520c0 | 526 | 2 |
| comp35526c0 | 2164 | 2 |
| comp35527c0 | 413 | 2 |
| comp35535c0 | 323 | 2 |
| comp35545c0 | 207 | 2 |
| comp35548c0 | 2252 | 2 |
| comp35550c0 | 255 | 2 |
| comp35553c0 | 691 | 2 |
| comp35554c0 | 195 | 2 |
| comp35570c0 | 111 | 2 |
| comp35574c0 | 1125 | 2 |
| comp35583c0 | 308 | 2 |
| comp35586c0 | 592 | 2 |
| comp35594c0 | 2238 | 2 |
| comp35601c0 | 613 | 2 |
| comp35603c0 | 764 | 2 |
| comp35608c0 | 254 | 2 |
| comp35613c0 | 700 | 2 |
| comp35621c0 | 1480 | 2 |
| comp35634c0 | 267 | 2 |
| comp35639c0 | 1386 | 2 |
| comp35644c0 | 2468 | 2 |
| comp35653c0 | 1443 | 2 |
| comp35667c0 | 890 | 2 |
| comp35669c0 | 953 | 2 |
| comp35670c0 | 27 | 2 |
| comp35678c1 | 1161 | 2 |
| comp35679c0 | 1322 | 2 |
| comp35693c0 | 100 | 2 |
| comp35702c0 | 610 | 2 |
| comp35707c0 | 1099 | 2 |
| comp35708c0 | 976 | 2 |
| comp35711c0 | 345 | 2 |
| comp35723c0 | 12217 | 2 |
| comp35734c0 | 716 | 2 |
| comp35735c0 | 814 | 2 |
| comp35739c0 | 997 | 2 |
| comp35745c0 | 1282 | 2 |
| comp35748c0 | 334 | 2 |
| comp35750c0 | 1737 | 2 |
| comp35764c0 | 500 | 2 |
| comp35780c0 | 402 | 2 |
| comp35822c0 | 1534 | 2 |
| comp35850c0 | 1546 | 2 |
| comp35868c0 | 1418 | 2 |
| comp35869c0 | 2062 | 2 |
| comp35875c0 | 1982 | 2 |
| comp358763c0 | 410 | 2 |
| comp35899c0 | 373 | 2 |
| comp35940c0 | 1262 | 2 |
| comp35949c0 | 929 | 2 |
| comp35965c0 | 102 | 2 |
| comp35975c0 | 118 | 2 |
| comp35998c0 | 505 | 2 |
| comp36012c0 | 560 | 2 |
| comp36047c0 | 216 | 2 |
| comp36087c0 | 501 | 2 |
| comp36093c0 | 799 | 2 |
| comp36112c0 | 447 | 2 |
| comp36121c0 | 1156 | 2 |
| comp36126c0 | 1189 | 2 |
| comp36161c0 | 625 | 2 |
| comp36172c0 | 478 | 2 |
| comp36212c0 | 148 | 2 |
| comp36226c0 | 305 | 2 |
| comp36243c0 | 744 | 2 |
| comp36248c0 | 458 | 2 |
| comp36259c0 | 1084 | 2 |
| comp36273c0 | 765 | 2 |
| comp36275c0 | 713 | 2 |
| comp36279c0 | 495 | 2 |
| comp36292c0 | 1408 | 2 |
| comp36294c0 | 922 | 2 |
| comp36295c0 | 181 | 2 |
| comp36303c0 | 662 | 2 |
| comp36327c0 | 1003 | 2 |
| comp36331c0 | 949 | 2 |
| comp36333c1 | 646 | 2 |
| comp36375c0 | 1259 | 2 |
| comp36644c0 | 121 | 2 |
| comp36661c0 | 554 | 2 |
| comp36726c0 | 141 | 2 |
| comp37076c0 | 882 | 2 |
| comp370819c0 | 537 | 2 |
| comp37156c0 | 191 | 2 |
| comp37298c0 | 906 | 2 |
| comp3765c0 | 567 | 2 |
| comp38111c0 | 586 | 2 |
| comp382493c0 | 1152 | 2 |
| comp38893c0 | 1050 | 2 |
| comp39012c0 | 3018 | 2 |
| comp390704c0 | 1069 | 2 |
| comp39477c0 | 1148 | 2 |
| comp39598c0 | 142 | 2 |
| comp39633c0 | 437 | 2 |
| comp39634c0 | 215 | 2 |
| comp39849c0 | 1760 | 2 |
| comp40348c0 | 50 | 2 |
| comp40425c0 | 2606 | 2 |
| comp40901c0 | 656 | 2 |
| comp41167c0 | 1770 | 2 |
| comp411696c0 | 1102 | 2 |
| comp41274c0 | 652 | 2 |
| comp4139c0 | 863 | 2 |
| comp41463c0 | 871 | 2 |
| comp41607c0 | 1165 | 2 |
| comp41635c0 | 390 | 2 |
| comp4178c0 | 2352 | 2 |
| comp41862c0 | 942 | 2 |
| comp41881c0 | 736 | 2 |
| comp41891c0 | 883 | 2 |
| comp42366c0 | 196 | 2 |
| comp42404c0 | 962 | 2 |
| comp42438c0 | 1081 | 2 |
| comp42446c0 | 265 | 2 |
| comp42551c0 | 741 | 2 |
| comp42561c0 | 2628 | 2 |
| comp42635c0 | 1373 | 2 |
| comp42646c0 | 1003 | 2 |
| comp42688c0 | 278 | 2 |
| comp42785c0 | 1144 | 2 |
| comp42811c0 | 1141 | 2 |
| comp42848c0 | 2132 | 2 |
| comp42865c0 | 471 | 2 |
| comp42871c0 | 1184 | 2 |
| comp42887c0 | 416 | 2 |
| comp42891c0 | 1316 | 2 |
| comp42898c0 | 629 | 2 |
| comp42910c0 | 395 | 2 |
| comp42916c0 | 425 | 2 |
| comp42927c0 | 762 | 2 |
| comp42949c0 | 1635 | 2 |
| comp43000c0 | 779 | 2 |
| comp43014c0 | 462 | 2 |
| comp43017c0 | 711 | 2 |
| comp43019c0 | 905 | 2 |
| comp43021c0 | 170 | 2 |
| comp43031c0 | 1051 | 2 |
| comp43032c0 | 1062 | 2 |
| comp43051c0 | 926 | 2 |
| comp43056c0 | 894 | 2 |
| comp43077c0 | 812 | 2 |
| comp43085c1 | 292 | 2 |
| comp43086c0 | 72 | 2 |
| comp43097c0 | 190 | 2 |
| comp43098c0 | 1343 | 2 |
| comp43099c0 | 632 | 2 |
| comp43107c0 | 955 | 2 |
| comp43110c0 | 901 | 2 |
| comp43132c0 | 387 | 2 |
| comp43134c0 | 283 | 2 |
| comp43140c0 | 177 | 2 |
| comp43152c0 | 595 | 2 |
| comp43154c0 | 421 | 2 |
| comp43157c0 | 850 | 2 |
| comp43183c0 | 1330 | 2 |
| comp43194c0 | 1217 | 2 |
| comp43198c0 | 1148 | 2 |
| comp43203c0 | 1012 | 2 |
| comp43204c0 | 547 | 2 |
| comp43214c0 | 859 | 2 |
| comp43230c0 | 634 | 2 |
| comp43263c0 | 838 | 2 |
| comp43264c0 | 798 | 2 |
| comp43266c0 | 197 | 2 |
| comp43285c0 | 104 | 2 |
| comp43321c0 | 525 | 2 |
| comp43323c0 | 581 | 2 |
| comp43327c0 | 954 | 2 |
| comp43340c0 | 1116 | 2 |
| comp43341c0 | 281 | 2 |
| comp43347c0 | 653 | 2 |
| comp43356c0 | 1535 | 2 |
| comp43367c0 | 1123 | 2 |
| comp43370c0 | 871 | 2 |
| comp43382c0 | 720 | 2 |
| comp43393c0 | 214 | 2 |
| comp43413c0 | 411 | 2 |
| comp43424c0 | 1569 | 2 |
| comp43428c0 | 175 | 2 |
| comp43452c0 | 227 | 2 |
| comp43501c0 | 1960 | 2 |
| comp43516c0 | 474 | 2 |
| comp43531c0 | 1211 | 2 |
| comp43535c0 | 76 | 2 |
| comp43537c1 | 814 | 2 |
| comp43560c0 | 254 | 2 |
| comp43568c0 | 361 | 2 |
| comp43569c0 | 84 | 2 |
| comp43578c0 | 309 | 2 |
| comp43584c0 | 1374 | 2 |
| comp43591c0 | 1493 | 2 |
| comp43593c0 | 1021 | 2 |
| comp435974c0 | 40 | 2 |
| comp43605c0 | 268 | 2 |
| comp43606c0 | 185 | 2 |
| comp43611c0 | 594 | 2 |
| comp43622c0 | 130 | 2 |
| comp43643c0 | 820 | 2 |
| comp43652c0 | 408 | 2 |
| comp43660c0 | 840 | 2 |
| comp43662c0 | 1507 | 2 |
| comp43687c0 | 945 | 2 |
| comp43691c0 | 1246 | 2 |
| comp43695c0 | 537 | 2 |
| comp43696c0 | 665 | 2 |
| comp43697c0 | 927 | 2 |
| comp43712c0 | 600 | 2 |
| comp43714c0 | 513 | 2 |
| comp43727c0 | 382 | 2 |
| comp437412c0 | 2096 | 2 |
| comp43749c0 | 1569 | 2 |
| comp43754c0 | 352 | 2 |
| comp43760c0 | 1165 | 2 |
| comp43762c0 | 870 | 2 |
| comp43766c0 | 173 | 2 |
| comp43770c0 | 654 | 2 |
| comp43791c0 | 574 | 2 |
| comp43792c0 | 133 | 2 |
| comp43829c1 | 1073 | 2 |
| comp43846c0 | 1131 | 2 |
| comp43868c0 | 714 | 2 |
| comp43882c0 | 93420 | 2 |
| comp43886c0 | 1034 | 2 |
| comp43901c0 | 546 | 2 |
| comp43907c0 | 352 | 2 |
| comp43918c0 | 509 | 2 |
| comp43919c0 | 180 | 2 |
| comp43921c0 | 948 | 2 |
| comp43937c0 | 1799 | 2 |
| comp43951c0 | 1404 | 2 |
| comp43981c0 | 938 | 2 |
| comp43983c0 | 689 | 2 |
| comp43992c0 | 98 | 2 |
| comp44005c0 | 776 | 2 |
| comp44006c0 | 1130 | 2 |
| comp44007c0 | 510 | 2 |
| comp44010c0 | 103 | 2 |
| comp44025c0 | 426 | 2 |
| comp44032c0 | 223 | 2 |
| comp44035c0 | 382 | 2 |
| comp44046c0 | 772 | 2 |
| comp44057c0 | 903 | 2 |
| comp44064c0 | 1245 | 2 |
| comp44072c0 | 673 | 2 |
| comp44073c0 | 385 | 2 |
| comp44075c0 | 129 | 2 |
| comp44082c1 | 630 | 2 |
| comp44084c0 | 1534 | 2 |
| comp44097c0 | 73 | 2 |
| comp44112c0 | 710 | 2 |
| comp44126c0 | 578 | 2 |
| comp44144c1 | 1220 | 2 |
| comp44145c0 | 977 | 2 |
| comp44145c1 | 194 | 2 |
| comp44145c2 | 209 | 2 |
| comp44151c0 | 1185 | 2 |
| comp44161c0 | 1037 | 2 |
| comp44173c0 | 2013 | 2 |
| comp44196c0 | 1058 | 2 |
| comp44266c0 | 473 | 2 |
| comp44292c0 | 929 | 2 |
| comp44328c0 | 289 | 2 |
| comp44331c0 | 234 | 2 |
| comp44393c0 | 961 | 2 |
| comp44405c0 | 106 | 2 |
| comp44432c0 | 321 | 2 |
| comp44438c0 | 193 | 2 |
| comp44443c0 | 456 | 2 |
| comp44460c0 | 118 | 2 |
| comp44463c0 | 1277 | 2 |
| comp44592c0 | 372 | 2 |
| comp44619c0 | 71 | 2 |
| comp44652c0 | 193 | 2 |
| comp44713c0 | 267 | 2 |
| comp44755c0 | 282 | 2 |
| comp44775c0 | 1172 | 2 |
| comp44788c0 | 476 | 2 |
| comp44793c0 | 1072 | 2 |
| comp44798c0 | 1057 | 2 |
| comp44799c0 | 821 | 2 |
| comp44829c1 | 254 | 2 |
| comp44861c0 | 427 | 2 |
| comp44863c1 | 357 | 2 |
| comp44870c0 | 378 | 2 |
| comp44871c0 | 1204 | 2 |
| comp44889c0 | 1204 | 2 |
| comp44895c0 | 463 | 2 |
| comp44932c0 | 529 | 2 |
| comp44935c0 | 732 | 2 |
| comp44991c0 | 699 | 2 |
| comp44997c0 | 914 | 2 |
| comp44997c1 | 105 | 2 |
| comp45008c0 | 1202 | 2 |
| comp45023c0 | 955 | 2 |
| comp45025c0 | 863 | 2 |
| comp45050c0 | 793 | 2 |
| comp45073c1 | 64 | 2 |
| comp45104c0 | 458 | 2 |
| comp45123c0 | 725 | 2 |
| comp45165c0 | 417 | 2 |
| comp45196c0 | 400 | 2 |
| comp45226c0 | 1295 | 2 |
| comp45353c0 | 713 | 2 |
| comp45372c0 | 1282 | 2 |
| comp45376c0 | 778 | 2 |
| comp45379c1 | 628 | 2 |
| comp45414c0 | 830 | 2 |
| comp45416c0 | 680 | 2 |
| comp45450c0 | 342 | 2 |
| comp45465c0 | 1757 | 2 |
| comp45563c0 | 987 | 2 |
| comp45751c0 | 1710 | 2 |
| comp45818c0 | 1395 | 2 |
| comp45860c0 | 921 | 2 |
| comp45936c0 | 461 | 2 |
| comp46009c0 | 1389 | 2 |
| comp46015c0 | 236 | 2 |
| comp46137c0 | 126 | 2 |
| comp46153c0 | 905 | 2 |
| comp46215c0 | 539 | 2 |
| comp463c1 | 2788 | 2 |
| comp46404c0 | 1480 | 2 |
| comp46424c0 | 19 | 2 |
| comp46540c0 | 352 | 2 |
| comp465492c0 | 1433 | 2 |
| comp46863c0 | 684 | 2 |
| comp46971c0 | 918 | 2 |
| comp472049c0 | 1452 | 2 |
| comp47246c0 | 265 | 2 |
| comp47327c0 | 846 | 2 |
| comp47441c0 | 604 | 2 |
| comp47475c0 | 2032 | 2 |
| comp480268c0 | 967 | 2 |
| comp48027c0 | 1047 | 2 |
| comp48027c1 | 1084 | 2 |
| comp48036c0 | 266 | 2 |
| comp48130c0 | 383 | 2 |
| comp48351c0 | 2145 | 2 |
| comp48455c0 | 1029 | 2 |
| comp48484c1 | 1012 | 2 |
| comp48967c0 | 456 | 2 |
| comp49077c0 | 89 | 2 |
| comp49259c0 | 1272 | 2 |
| comp495718c0 | 118 | 2 |
| comp49865c0 | 96 | 2 |
| comp49874c0 | 1640 | 2 |
| comp49894c1 | 485 | 2 |
| comp50027c0 | 784 | 2 |
| comp50113c0 | 1033 | 2 |
| comp50170c0 | 3159 | 2 |
| comp50182c0 | 687 | 2 |
| comp50197c0 | 383 | 2 |
| comp50393c0 | 521 | 2 |
| comp50717c0 | 780 | 2 |
| comp50733c1 | 231 | 2 |
| comp50946c0 | 412 | 2 |
| comp50951c0 | 322 | 2 |
| comp50972c0 | 446 | 2 |
| comp50976c0 | 1141 | 2 |
| comp50999c0 | 1316 | 2 |
| comp51018c1 | 12 | 2 |
| comp51022c0 | 465 | 2 |
| comp51080c0 | 439 | 2 |
| comp51098c0 | 2006 | 2 |
| comp51105c0 | 1829 | 2 |
| comp51112c0 | 1125 | 2 |
| comp51131c0 | 596 | 2 |
| comp51144c0 | 712 | 2 |
| comp51159c0 | 544 | 2 |
| comp51164c0 | 922 | 2 |
| comp51166c0 | 1611 | 2 |
| comp51176c0 | 77 | 2 |
| comp51191c0 | 1524 | 2 |
| comp51202c0 | 178 | 2 |
| comp51210c0 | 1434 | 2 |
| comp51249c0 | 574 | 2 |
| comp51252c0 | 230 | 2 |
| comp51259c0 | 261 | 2 |
| comp51263c0 | 802 | 2 |
| comp51266c0 | 320 | 2 |
| comp51275c0 | 978 | 2 |
| comp51279c0 | 1285 | 2 |
| comp51282c0 | 611 | 2 |
| comp51315c0 | 723 | 2 |
| comp51330c0 | 1226 | 2 |
| comp51340c0 | 832 | 2 |
| comp51353c0 | 193 | 2 |
| comp51359c0 | 1162 | 2 |
| comp51360c0 | 480 | 2 |
| comp51362c0 | 1986 | 2 |
| comp51367c0 | 507 | 2 |
| comp51391c0 | 604 | 2 |
| comp51394c0 | 197 | 2 |
| comp51404c0 | 1019 | 2 |
| comp51406c0 | 1221 | 2 |
| comp51413c0 | 347 | 2 |
| comp51420c0 | 2156 | 2 |
| comp51424c0 | 1626 | 2 |
| comp51426c0 | 807 | 2 |
| comp51439c0 | 2274 | 2 |
| comp51443c0 | 1106 | 2 |
| comp51443c1 | 1493 | 2 |
| comp51453c0 | 479 | 2 |
| comp51459c0 | 127 | 2 |
| comp51486c0 | 616 | 2 |
| comp51490c0 | 1406 | 2 |
| comp51497c0 | 1743 | 2 |
| comp51503c0 | 627 | 2 |
| comp51529c0 | 132 | 2 |
| comp51530c0 | 597 | 2 |
| comp51531c0 | 1051 | 2 |
| comp51534c0 | 288 | 2 |
| comp51555c0 | 95 | 2 |
| comp51560c0 | 984 | 2 |
| comp51575c0 | 695 | 2 |
| comp51578c0 | 670 | 2 |
| comp51581c0 | 480 | 2 |
| comp51590c0 | 751 | 2 |
| comp51601c0 | 298 | 2 |
| comp51602c0 | 318 | 2 |
| comp51605c0 | 981 | 2 |
| comp51606c0 | 789 | 2 |
| comp51619c0 | 542 | 2 |
| comp51622c0 | 730 | 2 |
| comp51624c0 | 721 | 2 |
| comp51627c0 | 734 | 2 |
| comp51631c0 | 35 | 2 |
| comp51638c1 | 248 | 2 |
| comp51641c0 | 1242 | 2 |
| comp51656c0 | 134 | 2 |
| comp51657c0 | 539 | 2 |
| comp51660c0 | 1051 | 2 |
| comp51664c0 | 121 | 2 |
| comp51687c0 | 1004 | 2 |
| comp51690c0 | 437 | 2 |
| comp51691c0 | 198 | 2 |
| comp51703c0 | 795 | 2 |
| comp51714c0 | 518 | 2 |
| comp51715c0 | 957 | 2 |
| comp51732c0 | 1346 | 2 |
| comp51733c0 | 46 | 2 |
| comp51738c0 | 247 | 2 |
| comp51741c0 | 2477 | 2 |
| comp51767c0 | 503 | 2 |
| comp51780c0 | 447 | 2 |
| comp51783c0 | 44 | 2 |
| comp51787c0 | 1333 | 2 |
| comp51788c0 | 543 | 2 |
| comp51790c0 | 542 | 2 |
| comp51795c0 | 82 | 2 |
| comp51797c0 | 1162 | 2 |
| comp51798c0 | 355 | 2 |
| comp51801c0 | 516 | 2 |
| comp51805c0 | 580 | 2 |
| comp51809c0 | 1195 | 2 |
| comp51826c0 | 510 | 2 |
| comp51843c0 | 974 | 2 |
| comp51845c0 | 185 | 2 |
| comp51856c0 | 1462 | 2 |
| comp51878c0 | 624 | 2 |
| comp51884c0 | 815 | 2 |
| comp51891c0 | 448 | 2 |
| comp51904c0 | 3466 | 2 |
| comp51907c0 | 742 | 2 |
| comp51922c0 | 1106 | 2 |
| comp51928c0 | 291 | 2 |
| comp51929c0 | 157 | 2 |
| comp51937c0 | 834 | 2 |
| comp51939c0 | 835 | 2 |
| comp51942c0 | 426 | 2 |
| comp51956c0 | 759 | 2 |
| comp51960c0 | 720 | 2 |
| comp51969c0 | 794 | 2 |
| comp51974c0 | 403 | 2 |
| comp51975c0 | 439 | 2 |
| comp51983c0 | 1039 | 2 |
| comp51985c0 | 575 | 2 |
| comp51988c0 | 47 | 2 |
| comp51990c0 | 401 | 2 |
| comp51993c0 | 1006 | 2 |
| comp52003c0 | 601 | 2 |
| comp52005c0 | 416 | 2 |
| comp52008c0 | 443 | 2 |
| comp52012c0 | 1102 | 2 |
| comp52019c0 | 468 | 2 |
| comp52023c1 | 1639 | 2 |
| comp52025c0 | 176 | 2 |
| comp52034c0 | 996 | 2 |
| comp52057c0 | 261 | 2 |
| comp52076c0 | 160 | 2 |
| comp52081c0 | 1495 | 2 |
| comp52086c0 | 946 | 2 |
| comp52088c0 | 952 | 2 |
| comp52099c0 | 91 | 2 |
| comp52105c0 | 987 | 2 |
| comp52106c0 | 202 | 2 |
| comp52120c0 | 745 | 2 |
| comp52123c0 | 288 | 2 |
| comp52130c0 | 843 | 2 |
| comp52131c0 | 77 | 2 |
| comp52139c0 | 201 | 2 |
| comp52160c1 | 737 | 2 |
| comp52167c0 | 899 | 2 |
| comp52171c0 | 939 | 2 |
| comp52176c0 | 398 | 2 |
| comp52214c0 | 2215 | 2 |
| comp52223c0 | 1484 | 2 |
| comp52239c0 | 51 | 2 |
| comp52266c0 | 838 | 2 |
| comp52276c0 | 238 | 2 |
| comp52312c0 | 413 | 2 |
| comp52327c0 | 500 | 2 |
| comp52334c0 | 519 | 2 |
| comp52347c0 | 432 | 2 |
| comp52375c0 | 1089 | 2 |
| comp52403c0 | 638 | 2 |
| comp52421c0 | 456 | 2 |
| comp52432c0 | 1357 | 2 |
| comp52434c0 | 411 | 2 |
| comp52476c0 | 789 | 2 |
| comp52483c0 | 895 | 2 |
| comp52488c0 | 359 | 2 |
| comp52513c0 | 125 | 2 |
| comp52522c0 | 868 | 2 |
| comp52534c0 | 670 | 2 |
| comp52537c0 | 1004 | 2 |
| comp52553c0 | 608 | 2 |
| comp52559c0 | 203 | 2 |
| comp52602c0 | 2 | 2 |
| comp52604c1 | 730 | 2 |
| comp52609c0 | 406 | 2 |
| comp52635c0 | 390 | 2 |
| comp52639c0 | 1953 | 2 |
| comp52649c0 | 457 | 2 |
| comp52659c0 | 774 | 2 |
| comp52661c0 | 392 | 2 |
| comp52661c1 | 225 | 2 |
| comp52664c0 | 437 | 2 |
| comp52665c0 | 637 | 2 |
| comp52666c0 | 141 | 2 |
| comp52671c0 | 230 | 2 |
| comp526857c0 | 1305 | 2 |
| comp52701c0 | 731 | 2 |
| comp52706c0 | 1231 | 2 |
| comp52707c0 | 914 | 2 |
| comp52726c1 | 132 | 2 |
| comp52750c0 | 1084 | 2 |
| comp52767c0 | 95 | 2 |
| comp52769c0 | 980 | 2 |
| comp52773c0 | 142 | 2 |
| comp52784c0 | 356 | 2 |
| comp52787c0 | 220 | 2 |
| comp52865c0 | 911 | 2 |
| comp52874c0 | 1101 | 2 |
| comp52877c0 | 550 | 2 |
| comp52878c0 | 528 | 2 |
| comp52887c0 | 591 | 2 |
| comp52890c0 | 1542 | 2 |
| comp52912c0 | 579 | 2 |
| comp52915c0 | 214 | 2 |
| comp52978c0 | 209 | 2 |
| comp52985c0 | 548 | 2 |
| comp53036c0 | 741 | 2 |
| comp53039c0 | 1233 | 2 |
| comp53048c0 | 390 | 2 |
| comp53048c1 | 77 | 2 |
| comp53058c0 | 166 | 2 |
| comp53111c0 | 1148 | 2 |
| comp53130c0 | 923 | 2 |
| comp53130c1 | 1166 | 2 |
| comp53159c0 | 1164 | 2 |
| comp53163c0 | 929 | 2 |
| comp53164c0 | 1424 | 2 |
| comp53202c0 | 175 | 2 |
| comp53225c0 | 282 | 2 |
| comp53247c0 | 1418 | 2 |
| comp53261c0 | 1620 | 2 |
| comp53291c0 | 1651 | 2 |
| comp53308c1 | 7488 | 2 |
| comp53319c0 | 1032 | 2 |
| comp53325c0 | 643 | 2 |
| comp53327c0 | 182 | 2 |
| comp53372c0 | 940 | 2 |
| comp53408c0 | 653 | 2 |
| comp53487c0 | 1982 | 2 |
| comp53539c0 | 1144 | 2 |
| comp53554c0 | 565 | 2 |
| comp53558c0 | 139 | 2 |
| comp53595c1 | 1319 | 2 |
| comp53659c0 | 128 | 2 |
| comp53662c0 | 1836 | 2 |
| comp53694c0 | 1205 | 2 |
| comp53716c0 | 1264 | 2 |
| comp53720c0 | 1527 | 2 |
| comp53736c0 | 1065 | 2 |
| comp53753c0 | 703 | 2 |
| comp538570c0 | 548 | 2 |
| comp53889c0 | 115 | 2 |
| comp53906c0 | 392 | 2 |
| comp53933c1 | 568 | 2 |
| comp53949c0 | 512 | 2 |
| comp53960c0 | 589 | 2 |
| comp53963c0 | 1029 | 2 |
| comp54026c0 | 42 | 2 |
| comp54052c0 | 1121 | 2 |
| comp54080c0 | 551 | 2 |
| comp54099c0 | 1017 | 2 |
| comp54112c0 | 282 | 2 |
| comp541261c0 | 331 | 2 |
| comp54144c0 | 1551 | 2 |
| comp54172c0 | 822 | 2 |
| comp54197c0 | 1276 | 2 |
| comp54203c0 | 570 | 2 |
| comp54208c0 | 937 | 2 |
| comp54228c0 | 693 | 2 |
| comp54286c0 | 1386 | 2 |
| comp54325c0 | 779 | 2 |
| comp54334c0 | 653 | 2 |
| comp54388c0 | 205 | 2 |
| comp54393c0 | 635 | 2 |
| comp54409c0 | 616 | 2 |
| comp54451c0 | 1105 | 2 |
| comp54466c0 | 2098 | 2 |
| comp54483c0 | 678 | 2 |
| comp54496c0 | 290 | 2 |
| comp54504c0 | 46 | 2 |
| comp54523c0 | 518 | 2 |
| comp54534c0 | 1268 | 2 |
| comp54539c0 | 644 | 2 |
| comp54583c0 | 954 | 2 |
| comp54621c1 | 1183 | 2 |
| comp54676c0 | 2151 | 2 |
| comp54701c0 | 1230 | 2 |
| comp54769c0 | 651 | 2 |
| comp54792c0 | 1060 | 2 |
| comp54817c0 | 1398 | 2 |
| comp54854c0 | 637 | 2 |
| comp54902c0 | 1802 | 2 |
| comp54944c0 | 725 | 2 |
| comp54948c0 | 174 | 2 |
| comp54999c0 | 999 | 2 |
| comp55035c0 | 608 | 2 |
| comp55036c0 | 518 | 2 |
| comp55050c0 | 510 | 2 |
| comp55082c0 | 1319 | 2 |
| comp55085c0 | 171 | 2 |
| comp55093c0 | 1434 | 2 |
| comp55094c0 | 717 | 2 |
| comp55106c0 | 1021 | 2 |
| comp55117c0 | 1114 | 2 |
| comp55124c0 | 768 | 2 |
| comp55127c0 | 448 | 2 |
| comp55129c0 | 196 | 2 |
| comp55132c0 | 829 | 2 |
| comp55142c0 | 1742 | 2 |
| comp55157c0 | 338 | 2 |
| comp55169c0 | 1620 | 2 |
| comp55171c0 | 440 | 2 |
| comp55174c0 | 629 | 2 |
| comp55176c1 | 955 | 2 |
| comp55188c0 | 543 | 2 |
| comp55192c0 | 1169 | 2 |
| comp55196c0 | 594 | 2 |
| comp55198c0 | 463 | 2 |
| comp55208c0 | 36 | 2 |
| comp55219c0 | 544 | 2 |
| comp55224c0 | 3530 | 2 |
| comp55227c0 | 481 | 2 |
| comp55246c0 | 322 | 2 |
| comp55246c1 | 66 | 2 |
| comp55285c0 | 998 | 2 |
| comp55306c0 | 123 | 2 |
| comp55312c0 | 191 | 2 |
| comp55313c0 | 1654 | 2 |
| comp55319c0 | 270 | 2 |
| comp55322c0 | 132 | 2 |
| comp55327c0 | 976 | 2 |
| comp55328c0 | 596 | 2 |
| comp55362c0 | 1199 | 2 |
| comp55375c0 | 1291 | 2 |
| comp55402c0 | 1202 | 2 |
| comp55405c0 | 461 | 2 |
| comp55407c0 | 1046 | 2 |
| comp55456c0 | 578 | 2 |
| comp55462c0 | 819 | 2 |
| comp55469c0 | 769 | 2 |
| comp55480c0 | 1168 | 2 |
| comp55498c0 | 1252 | 2 |
| comp55502c0 | 588 | 2 |
| comp55503c0 | 1169 | 2 |
| comp55504c0 | 859 | 2 |
| comp55520c0 | 759 | 2 |
| comp55538c0 | 953 | 2 |
| comp55547c0 | 530 | 2 |
| comp55558c0 | 327 | 2 |
| comp55577c0 | 380 | 2 |
| comp55580c0 | 1353 | 2 |
| comp55589c0 | 780 | 2 |
| comp55596c0 | 928 | 2 |
| comp55602c0 | 303 | 2 |
| comp55609c0 | 708 | 2 |
| comp55610c0 | 789 | 2 |
| comp55611c0 | 479 | 2 |
| comp55612c0 | 763 | 2 |
| comp55613c0 | 217 | 2 |
| comp55622c0 | 96 | 2 |
| comp55626c0 | 1039 | 2 |
| comp55627c0 | 935 | 2 |
| comp55644c0 | 253 | 2 |
| comp55655c0 | 243 | 2 |
| comp55664c0 | 1570 | 2 |
| comp55668c0 | 1211 | 2 |
| comp55677c0 | 1304 | 2 |
| comp55682c0 | 2308 | 2 |
| comp55683c0 | 269 | 2 |
| comp55690c0 | 869 | 2 |
| comp55695c0 | 1003 | 2 |
| comp55707c0 | 789 | 2 |
| comp55709c0 | 249 | 2 |
| comp55730c0 | 933 | 2 |
| comp55736c0 | 1375 | 2 |
| comp55761c0 | 266 | 2 |
| comp55798c0 | 323 | 2 |
| comp55805c0 | 641 | 2 |
| comp55809c0 | 1132 | 2 |
| comp55819c0 | 195 | 2 |
| comp55832c0 | 871 | 2 |
| comp55834c0 | 858 | 2 |
| comp55834c1 | 93 | 2 |
| comp55857c0 | 1561 | 2 |
| comp55866c0 | 449 | 2 |
| comp55871c0 | 1290 | 2 |
| comp55883c0 | 265 | 2 |
| comp55909c0 | 954 | 2 |
| comp55917c1 | 592 | 2 |
| comp55921c0 | 752 | 2 |
| comp55924c0 | 428 | 2 |
| comp55927c0 | 731 | 2 |
| comp55931c0 | 784 | 2 |
| comp55942c0 | 718 | 2 |
| comp55968c0 | 2326 | 2 |
| comp55972c0 | 603 | 2 |
| comp55975c0 | 89 | 2 |
| comp55987c0 | 367 | 2 |
| comp55997c0 | 906 | 2 |
| comp56001c0 | 1582 | 2 |
| comp56030c0 | 202 | 2 |
| comp56039c0 | 73 | 2 |
| comp56047c0 | 1521 | 2 |
| comp56058c0 | 1461 | 2 |
| comp56066c0 | 1570 | 2 |
| comp56069c0 | 915 | 2 |
| comp56095c0 | 208 | 2 |
| comp56125c0 | 497 | 2 |
| comp56128c0 | 136 | 2 |
| comp56133c0 | 833 | 2 |
| comp56149c0 | 458 | 2 |
| comp56152c0 | 1060 | 2 |
| comp56155c0 | 1603 | 2 |
| comp56160c1 | 1035 | 2 |
| comp56164c0 | 36 | 2 |
| comp56180c0 | 616 | 2 |
| comp56182c0 | 1558 | 2 |
| comp56184c0 | 184 | 2 |
| comp56188c0 | 1033 | 2 |
| comp56193c0 | 2201 | 2 |
| comp56194c0 | 813 | 2 |
| comp56201c0 | 425 | 2 |
| comp56229c0 | 339 | 2 |
| comp56243c0 | 571 | 2 |
| comp56258c0 | 647 | 2 |
| comp56277c0 | 599 | 2 |
| comp56284c0 | 392 | 2 |
| comp56300c0 | 141 | 2 |
| comp56316c0 | 518 | 2 |
| comp56320c0 | 570 | 2 |
| comp56335c0 | 235 | 2 |
| comp56338c0 | 1472 | 2 |
| comp56342c0 | 1270 | 2 |
| comp56347c0 | 99 | 2 |
| comp56348c0 | 334 | 2 |
| comp56357c0 | 728 | 2 |
| comp56377c0 | 672 | 2 |
| comp56384c1 | 482 | 2 |
| comp56411c0 | 1147 | 2 |
| comp56419c0 | 619 | 2 |
| comp56423c0 | 397 | 2 |
| comp56427c0 | 298 | 2 |
| comp56441c0 | 1666 | 2 |
| comp56454c0 | 886 | 2 |
| comp56455c0 | 436 | 2 |
| comp56456c0 | 641 | 2 |
| comp56457c0 | 319 | 2 |
| comp56469c0 | 1138 | 2 |
| comp56475c0 | 923 | 2 |
| comp56477c0 | 1046 | 2 |
| comp56503c1 | 1680 | 2 |
| comp56515c0 | 218 | 2 |
| comp56518c1 | 348 | 2 |
| comp56521c0 | 207 | 2 |
| comp56552c0 | 324 | 2 |
| comp56585c0 | 463 | 2 |
| comp56585c1 | 791 | 2 |
| comp56597c0 | 413 | 2 |
| comp56598c0 | 312 | 2 |
| comp56613c0 | 895 | 2 |
| comp56617c0 | 568 | 2 |
| comp56630c0 | 469 | 2 |
| comp56631c0 | 1050 | 2 |
| comp56649c0 | 83 | 2 |
| comp56653c0 | 1365 | 2 |
| comp56656c1 | 412 | 2 |
| comp56734c0 | 104 | 2 |
| comp56735c1 | 411 | 2 |
| comp56761c0 | 994 | 2 |
| comp56772c0 | 373 | 2 |
| comp56810c0 | 643 | 2 |
| comp56826c0 | 198 | 2 |
| comp56830c0 | 971 | 2 |
| comp56832c0 | 775 | 2 |
| comp56832c1 | 100 | 2 |
| comp56834c0 | 566 | 2 |
| comp56835c0 | 82 | 2 |
| comp56837c0 | 2020 | 2 |
| comp56846c0 | 621 | 2 |
| comp56851c0 | 311 | 2 |
| comp56852c0 | 902 | 2 |
| comp56856c1 | 810 | 2 |
| comp56860c0 | 499 | 2 |
| comp56878c0 | 627 | 2 |
| comp56882c0 | 596 | 2 |
| comp56903c0 | 927 | 2 |
| comp56905c0 | 449 | 2 |
| comp56913c0 | 600 | 2 |
| comp56915c0 | 300 | 2 |
| comp56935c0 | 1219 | 2 |
| comp56939c0 | 785 | 2 |
| comp56963c0 | 673 | 2 |
| comp56963c1 | 98 | 2 |
| comp56964c0 | 841 | 2 |
| comp56965c0 | 324 | 2 |
| comp56966c0 | 188 | 2 |
| comp56968c0 | 407 | 2 |
| comp56977c0 | 694 | 2 |
| comp56990c0 | 619 | 2 |
| comp56998c0 | 621 | 2 |
| comp57006c1 | 1194 | 2 |
| comp57010c0 | 1029 | 2 |
| comp57013c0 | 425 | 2 |
| comp57015c0 | 338 | 2 |
| comp57019c0 | 1354 | 2 |
| comp57028c0 | 226 | 2 |
| comp57062c0 | 2305 | 2 |
| comp57064c0 | 566 | 2 |
| comp57074c0 | 729 | 2 |
| comp57079c0 | 1217 | 2 |
| comp57086c0 | 782 | 2 |
| comp57088c0 | 1397 | 2 |
| comp57094c0 | 390 | 2 |
| comp57106c0 | 397 | 2 |
| comp57118c0 | 569 | 2 |
| comp57122c0 | 1406 | 2 |
| comp57135c0 | 1021 | 2 |
| comp57137c0 | 771 | 2 |
| comp57156c0 | 540 | 2 |
| comp57163c0 | 363 | 2 |
| comp57166c0 | 656 | 2 |
| comp57176c0 | 818 | 2 |
| comp57179c0 | 471 | 2 |
| comp57185c0 | 1039 | 2 |
| comp57190c0 | 1157 | 2 |
| comp57204c0 | 479 | 2 |
| comp57207c0 | 507 | 2 |
| comp57213c0 | 80 | 2 |
| comp57219c0 | 1307 | 2 |
| comp57231c0 | 415 | 2 |
| comp57239c0 | 756 | 2 |
| comp57263c0 | 1069 | 2 |
| comp57281c0 | 1541 | 2 |
| comp57287c0 | 1039 | 2 |
| comp57296c0 | 322 | 2 |
| comp57303c0 | 1194 | 2 |
| comp57330c0 | 1049 | 2 |
| comp57371c0 | 1304 | 2 |
| comp57373c0 | 601 | 2 |
| comp57382c0 | 745 | 2 |
| comp57420c0 | 1658 | 2 |
| comp57450c0 | 377 | 2 |
| comp57457c0 | 270 | 2 |
| comp57481c0 | 941 | 2 |
| comp57494c0 | 671 | 2 |
| comp57494c1 | 959 | 2 |
| comp57511c0 | 134 | 2 |
| comp57514c0 | 217 | 2 |
| comp57517c0 | 1221 | 2 |
| comp57518c0 | 15 | 2 |
| comp57549c0 | 1456 | 2 |
| comp57558c0 | 936 | 2 |
| comp57578c0 | 1380 | 2 |
| comp57579c0 | 937 | 2 |
| comp57582c0 | 4 | 2 |
| comp57592c0 | 418 | 2 |
| comp57595c0 | 630 | 2 |
| comp57614c0 | 993 | 2 |
| comp57624c0 | 498 | 2 |
| comp57629c0 | 1068 | 2 |
| comp57634c0 | 827 | 2 |
| comp57641c0 | 780 | 2 |
| comp57648c0 | 466 | 2 |
| comp57649c0 | 806 | 2 |
| comp57664c0 | 965 | 2 |
| comp57669c0 | 898 | 2 |
| comp57671c0 | 905 | 2 |
| comp57671c1 | 800 | 2 |
| comp57685c0 | 448 | 2 |
| comp57694c0 | 1049 | 2 |
| comp57706c0 | 657 | 2 |
| comp57708c0 | 76 | 2 |
| comp57711c0 | 994 | 2 |
| comp57712c0 | 221 | 2 |
| comp57723c0 | 912 | 2 |
| comp57725c0 | 1130 | 2 |
| comp57736c0 | 232 | 2 |
| comp57749c0 | 194 | 2 |
| comp57759c0 | 2118 | 2 |
| comp57761c0 | 481 | 2 |
| comp57762c0 | 551 | 2 |
| comp57765c0 | 1627 | 2 |
| comp57766c0 | 114 | 2 |
| comp57768c0 | 840 | 2 |
| comp57770c0 | 313 | 2 |
| comp57775c1 | 128 | 2 |
| comp57786c0 | 585 | 2 |
| comp57788c0 | 2 | 2 |
| comp57794c0 | 873 | 2 |
| comp57795c0 | 948 | 2 |
| comp57798c0 | 438 | 2 |
| comp57799c0 | 1253 | 2 |
| comp57813c0 | 273 | 2 |
| comp57828c0 | 293 | 2 |
| comp57833c0 | 158 | 2 |
| comp57834c0 | 322 | 2 |
| comp57837c0 | 503 | 2 |
| comp57846c0 | 516 | 2 |
| comp57850c0 | 1381 | 2 |
| comp57901c0 | 525 | 2 |
| comp57903c0 | 10 | 2 |
| comp57908c0 | 511 | 2 |
| comp57909c0 | 934 | 2 |
| comp57911c0 | 33 | 2 |
| comp57916c0 | 666 | 2 |
| comp57931c0 | 971 | 2 |
| comp57935c0 | 918 | 2 |
| comp57947c0 | 874 | 2 |
| comp57956c0 | 495 | 2 |
| comp57964c0 | 531 | 2 |
| comp57974c0 | 721 | 2 |
| comp57989c0 | 139 | 2 |
| comp58005c0 | 239 | 2 |
| comp58009c0 | 901 | 2 |
| comp58018c0 | 372 | 2 |
| comp58035c0 | 80 | 2 |
| comp58039c0 | 2516 | 2 |
| comp58042c0 | 463 | 2 |
| comp58044c0 | 1007 | 2 |
| comp58046c0 | 1397 | 2 |
| comp58047c0 | 314 | 2 |
| comp58055c0 | 1511 | 2 |
| comp58056c0 | 2984 | 2 |
| comp58060c0 | 1214 | 2 |
| comp58068c0 | 1558 | 2 |
| comp58081c0 | 723 | 2 |
| comp58082c0 | 481 | 2 |
| comp58083c0 | 1342 | 2 |
| comp58091c0 | 1472 | 2 |
| comp58094c0 | 1142 | 2 |
| comp58096c0 | 880 | 2 |
| comp58097c0 | 641 | 2 |
| comp58098c0 | 448 | 2 |
| comp58104c0 | 727 | 2 |
| comp58108c0 | 1142 | 2 |
| comp58134c0 | 524 | 2 |
| comp58147c0 | 1277 | 2 |
| comp58159c0 | 314 | 2 |
| comp58160c1 | 1218 | 2 |
| comp58171c0 | 1933 | 2 |
| comp58175c0 | 1052 | 2 |
| comp58178c0 | 882 | 2 |
| comp58190c0 | 272 | 2 |
| comp58209c0 | 241 | 2 |
| comp58213c0 | 1445 | 2 |
| comp58215c0 | 35 | 2 |
| comp58221c0 | 491 | 2 |
| comp58223c0 | 417 | 2 |
| comp58236c0 | 175 | 2 |
| comp58247c1 | 719 | 2 |
| comp58254c0 | 1095 | 2 |
| comp58261c0 | 753 | 2 |
| comp58263c0 | 490 | 2 |
| comp58268c0 | 564 | 2 |
| comp58269c0 | 2547 | 2 |
| comp58270c0 | 513 | 2 |
| comp58272c0 | 1817 | 2 |
| comp58277c0 | 579 | 2 |
| comp58277c1 | 846 | 2 |
| comp58283c0 | 430 | 2 |
| comp58300c0 | 843 | 2 |
| comp58306c0 | 2505 | 2 |
| comp58308c0 | 184 | 2 |
| comp58320c0 | 992 | 2 |
| comp58337c0 | 745 | 2 |
| comp58341c0 | 315 | 2 |
| comp58342c0 | 135 | 2 |
| comp58345c0 | 844 | 2 |
| comp58355c1 | 1081 | 2 |
| comp58357c0 | 186 | 2 |
| comp58364c0 | 1131 | 2 |
| comp58366c0 | 733 | 2 |
| comp58368c0 | 569 | 2 |
| comp58371c0 | 839 | 2 |
| comp58384c0 | 1229 | 2 |
| comp58387c0 | 731 | 2 |
| comp58388c0 | 707 | 2 |
| comp58389c0 | 1178 | 2 |
| comp58409c0 | 256 | 2 |
| comp58413c0 | 755 | 2 |
| comp58414c0 | 1376 | 2 |
| comp58425c0 | 1459 | 2 |
| comp58431c0 | 899 | 2 |
| comp58435c0 | 373 | 2 |
| comp58457c0 | 1478 | 2 |
| comp58464c0 | 382 | 2 |
| comp58478c0 | 650 | 2 |
| comp58480c0 | 1469 | 2 |
| comp58484c0 | 1173 | 2 |
| comp58500c0 | 460 | 2 |
| comp58513c0 | 2239 | 2 |
| comp58520c0 | 565 | 2 |
| comp58524c0 | 539 | 2 |
| comp58529c0 | 767 | 2 |
| comp58540c0 | 188 | 2 |
| comp58559c0 | 416 | 2 |
| comp58560c0 | 797 | 2 |
| comp58561c1 | 885 | 2 |
| comp58562c1 | 375 | 2 |
| comp58563c0 | 2115 | 2 |
| comp58565c0 | 1124 | 2 |
| comp58572c0 | 310 | 2 |
| comp58574c0 | 293 | 2 |
| comp58585c0 | 488 | 2 |
| comp58596c0 | 777 | 2 |
| comp58601c0 | 1278 | 2 |
| comp58604c0 | 394 | 2 |
| comp58605c0 | 304 | 2 |
| comp58641c0 | 1212 | 2 |
| comp58654c0 | 746 | 2 |
| comp58656c0 | 1246 | 2 |
| comp58671c1 | 408 | 2 |
| comp58673c0 | 1255 | 2 |
| comp58680c0 | 42 | 2 |
| comp58688c0 | 856 | 2 |
| comp58723c0 | 1632 | 2 |
| comp58730c0 | 951 | 2 |
| comp58732c0 | 608 | 2 |
| comp58735c0 | 426 | 2 |
| comp58742c0 | 846 | 2 |
| comp58754c0 | 596 | 2 |
| comp58755c0 | 929 | 2 |
| comp58756c0 | 275 | 2 |
| comp58760c0 | 504 | 2 |
| comp58763c0 | 601 | 2 |
| comp58773c0 | 983 | 2 |
| comp58774c0 | 1759 | 2 |
| comp58794c0 | 891 | 2 |
| comp58800c0 | 465 | 2 |
| comp58801c0 | 245 | 2 |
| comp58806c0 | 807 | 2 |
| comp58818c0 | 261 | 2 |
| comp58823c0 | 547 | 2 |
| comp58849c0 | 687 | 2 |
| comp58855c0 | 398 | 2 |
| comp58856c0 | 700 | 2 |
| comp58867c0 | 2304 | 2 |
| comp58873c1 | 878 | 2 |
| comp58886c0 | 2244 | 2 |
| comp58892c0 | 409 | 2 |
| comp58896c0 | 105 | 2 |
| comp58909c0 | 1428 | 2 |
| comp58922c0 | 1513 | 2 |
| comp58928c0 | 743 | 2 |
| comp58931c0 | 479 | 2 |
| comp58932c0 | 63 | 2 |
| comp58948c0 | 975 | 2 |
| comp58973c0 | 573 | 2 |
| comp58980c0 | 683 | 2 |
| comp59005c0 | 742 | 2 |
| comp59008c0 | 1173 | 2 |
| comp59017c0 | 1925 | 2 |
| comp59026c0 | 560 | 2 |
| comp59029c0 | 411 | 2 |
| comp59036c0 | 526 | 2 |
| comp59041c0 | 659 | 2 |
| comp59054c0 | 532 | 2 |
| comp59063c0 | 780 | 2 |
| comp59077c1 | 590 | 2 |
| comp59087c0 | 863 | 2 |
| comp59092c0 | 837 | 2 |
| comp59100c0 | 2568 | 2 |
| comp59102c0 | 660 | 2 |
| comp59124c0 | 972 | 2 |
| comp59125c0 | 354 | 2 |
| comp59128c0 | 1150 | 2 |
| comp59130c0 | 318 | 2 |
| comp59134c0 | 578 | 2 |
| comp59149c0 | 1565 | 2 |
| comp59151c0 | 464 | 2 |
| comp59155c2 | 36275 | 2 |
| comp59158c0 | 755 | 2 |
| comp59162c0 | 1287 | 2 |
| comp59168c0 | 177 | 2 |
| comp59171c0 | 474 | 2 |
| comp59188c0 | 172 | 2 |
| comp59192c0 | 560 | 2 |
| comp59199c0 | 741 | 2 |
| comp59204c0 | 860 | 2 |
| comp59205c0 | 544 | 2 |
| comp59213c0 | 960 | 2 |
| comp59217c0 | 102 | 2 |
| comp59248c0 | 595 | 2 |
| comp59265c0 | 637 | 2 |
| comp59266c0 | 415 | 2 |
| comp59269c1 | 563 | 2 |
| comp59270c0 | 1682 | 2 |
| comp59277c0 | 295 | 2 |
| comp59288c0 | 916 | 2 |
| comp59289c0 | 677 | 2 |
| comp59297c0 | 911 | 2 |
| comp59313c0 | 1857 | 2 |
| comp59320c0 | 288 | 2 |
| comp59326c0 | 1808 | 2 |
| comp59332c0 | 982 | 2 |
| comp59345c0 | 86 | 2 |
| comp59349c0 | 1102 | 2 |
| comp59362c0 | 1004 | 2 |
| comp59367c0 | 492 | 2 |
| comp59368c0 | 594 | 2 |
| comp59378c0 | 134 | 2 |
| comp59400c0 | 840 | 2 |
| comp59407c0 | 468 | 2 |
| comp59407c1 | 468 | 2 |
| comp59408c0 | 272 | 2 |
| comp59408c1 | 212 | 2 |
| comp59423c0 | 916 | 2 |
| comp59426c1 | 250 | 2 |
| comp59435c0 | 694 | 2 |
| comp59437c0 | 457 | 2 |
| comp59441c0 | 654 | 2 |
| comp59442c0 | 633 | 2 |
| comp59443c0 | 923 | 2 |
| comp59445c0 | 212 | 2 |
| comp59448c0 | 117 | 2 |
| comp59457c0 | 335 | 2 |
| comp59468c0 | 828 | 2 |
| comp59471c0 | 591 | 2 |
| comp59473c0 | 604 | 2 |
| comp59474c0 | 910 | 2 |
| comp59480c0 | 897 | 2 |
| comp59484c0 | 722 | 2 |
| comp59486c0 | 2449 | 2 |
| comp59491c0 | 595 | 2 |
| comp59498c0 | 39 | 2 |
| comp59503c0 | 2906 | 2 |
| comp59505c0 | 38452 | 2 |
| comp59509c0 | 40 | 2 |
| comp59510c0 | 917 | 2 |
| comp59511c0 | 879 | 2 |
| comp59512c0 | 976 | 2 |
| comp59514c0 | 135 | 2 |
| comp59515c0 | 731 | 2 |
| comp59518c0 | 61 | 2 |
| comp59521c1 | 0 | 2 |
| comp59523c0 | 227 | 2 |
| comp59525c1 | 379 | 2 |
| comp59527c0 | 401 | 2 |
| comp59533c1 | 773 | 2 |
| comp59535c0 | 302 | 2 |
| comp59538c0 | 1088 | 2 |
| comp59539c0 | 1366 | 2 |
| comp59561c0 | 1256 | 2 |
| comp59562c0 | 64 | 2 |
| comp59567c1 | 715 | 2 |
| comp59571c0 | 222 | 2 |
| comp59582c0 | 618 | 2 |
| comp59591c0 | 1059 | 2 |
| comp59599c0 | 1037 | 2 |
| comp59602c0 | 794 | 2 |
| comp59603c0 | 1245 | 2 |
| comp59605c0 | 1247 | 2 |
| comp59614c0 | 120 | 2 |
| comp59628c0 | 328 | 2 |
| comp59641c0 | 111 | 2 |
| comp59645c0 | 672 | 2 |
| comp59647c0 | 609 | 2 |
| comp59653c0 | 203 | 2 |
| comp59654c0 | 357 | 2 |
| comp59660c0 | 662 | 2 |
| comp59662c0 | 192 | 2 |
| comp59664c0 | 3916 | 2 |
| comp59670c0 | 1658 | 2 |
| comp59676c0 | 39 | 2 |
| comp59678c0 | 1632 | 2 |
| comp59694c0 | 994 | 2 |
| comp59697c0 | 106 | 2 |
| comp59703c0 | 826 | 2 |
| comp59705c0 | 1150 | 2 |
| comp59716c0 | 96 | 2 |
| comp59717c0 | 844 | 2 |
| comp59721c0 | 626 | 2 |
| comp59722c0 | 100 | 2 |
| comp59728c0 | 1160 | 2 |
| comp59731c0 | 1146 | 2 |
| comp59732c0 | 497 | 2 |
| comp59736c0 | 1400 | 2 |
| comp59742c0 | 649 | 2 |
| comp59745c0 | 382 | 2 |
| comp59756c0 | 1178 | 2 |
| comp59762c0 | 333 | 2 |
| comp59765c0 | 291 | 2 |
| comp59767c0 | 85 | 2 |
| comp59773c0 | 564 | 2 |
| comp59779c0 | 504 | 2 |
| comp59787c0 | 179 | 2 |
| comp59792c0 | 806 | 2 |
| comp59800c0 | 779 | 2 |
| comp59813c0 | 830 | 2 |
| comp59815c0 | 1404 | 2 |
| comp59826c0 | 248 | 2 |
| comp59830c0 | 263 | 2 |
| comp59832c0 | 880 | 2 |
| comp59843c0 | 1566 | 2 |
| comp59849c0 | 830 | 2 |
| comp59857c0 | 1408 | 2 |
| comp59863c0 | 777 | 2 |
| comp59865c0 | 167 | 2 |
| comp59866c0 | 318 | 2 |
| comp59876c0 | 1519 | 2 |
| comp59877c0 | 832 | 2 |
| comp59881c0 | 363 | 2 |
| comp59885c0 | 1172 | 2 |
| comp59895c0 | 12 | 2 |
| comp59901c0 | 953 | 2 |
| comp59923c0 | 1079 | 2 |
| comp59927c0 | 2368 | 2 |
| comp59928c0 | 1504 | 2 |
| comp59929c1 | 28 | 2 |
| comp59932c0 | 1263 | 2 |
| comp59938c0 | 217 | 2 |
| comp59943c0 | 853 | 2 |
| comp59944c0 | 959 | 2 |
| comp59946c0 | 726 | 2 |
| comp59949c0 | 56 | 2 |
| comp59960c0 | 996 | 2 |
| comp59966c0 | 137 | 2 |
| comp59977c0 | 456 | 2 |
| comp59980c0 | 153 | 2 |
| comp59984c0 | 1013 | 2 |
| comp59994c0 | 180 | 2 |
| comp60015c0 | 387 | 2 |
| comp60019c0 | 690 | 2 |
| comp60022c0 | 916 | 2 |
| comp60023c0 | 319 | 2 |
| comp60026c0 | 278 | 2 |
| comp60030c0 | 42 | 2 |
| comp60060c0 | 865 | 2 |
| comp60062c0 | 507 | 2 |
| comp60074c1 | 183 | 2 |
| comp60076c0 | 216 | 2 |
| comp60078c0 | 449 | 2 |
| comp60083c0 | 1071 | 2 |
| comp60084c0 | 924 | 2 |
| comp60085c0 | 1109 | 2 |
| comp60087c0 | 443 | 2 |
| comp60091c0 | 164 | 2 |
| comp60100c0 | 1093 | 2 |
| comp60106c0 | 1317 | 2 |
| comp60111c0 | 397 | 2 |
| comp60115c0 | 744 | 2 |
| comp60117c0 | 1027 | 2 |
| comp60120c0 | 815 | 2 |
| comp60122c0 | 983 | 2 |
| comp60123c0 | 600 | 2 |
| comp60135c0 | 859 | 2 |
| comp60140c0 | 120 | 2 |
| comp60146c0 | 521 | 2 |
| comp60157c0 | 574 | 2 |
| comp60160c0 | 2130 | 2 |
| comp60161c0 | 322 | 2 |
| comp60163c0 | 462 | 2 |
| comp60166c0 | 365 | 2 |
| comp60168c0 | 873 | 2 |
| comp60169c0 | 2096 | 2 |
| comp60178c0 | 247 | 2 |
| comp60178c1 | 783 | 2 |
| comp60179c0 | 308 | 2 |
| comp60180c0 | 520 | 2 |
| comp60181c0 | 1034 | 2 |
| comp60190c0 | 883 | 2 |
| comp60191c0 | 1138 | 2 |
| comp60192c0 | 1233 | 2 |
| comp60202c0 | 328 | 2 |
| comp60210c0 | 322 | 2 |
| comp60213c0 | 1352 | 2 |
| comp60219c0 | 291 | 2 |
| comp60220c1 | 956 | 2 |
| comp60222c0 | 1219 | 2 |
| comp60235c0 | 1661 | 2 |
| comp60246c0 | 304 | 2 |
| comp60248c0 | 364 | 2 |
| comp60251c0 | 944 | 2 |
| comp60256c0 | 351 | 2 |
| comp60259c0 | 888 | 2 |
| comp60261c0 | 286 | 2 |
| comp60265c0 | 598 | 2 |
| comp60268c0 | 348 | 2 |
| comp60269c0 | 167 | 2 |
| comp60279c0 | 204 | 2 |
| comp60282c0 | 279 | 2 |
| comp60286c0 | 820 | 2 |
| comp60294c0 | 919 | 2 |
| comp60301c0 | 1361 | 2 |
| comp60304c0 | 1888 | 2 |
| comp60323c0 | 814 | 2 |
| comp60325c0 | 1044 | 2 |
| comp60327c0 | 404 | 2 |
| comp60329c0 | 1152 | 2 |
| comp60338c0 | 519 | 2 |
| comp60340c0 | 188 | 2 |
| comp60342c0 | 195 | 2 |
| comp60345c0 | 2481 | 2 |
| comp60351c0 | 855 | 2 |
| comp60354c0 | 921 | 2 |
| comp60358c0 | 402 | 2 |
| comp60361c0 | 20 | 2 |
| comp60362c0 | 683 | 2 |
| comp60363c0 | 484 | 2 |
| comp60367c0 | 585 | 2 |
| comp60368c0 | 417 | 2 |
| comp60374c0 | 545 | 2 |
| comp60378c0 | 994 | 2 |
| comp60381c0 | 1463 | 2 |
| comp60385c0 | 906 | 2 |
| comp60389c0 | 1556 | 2 |
| comp6039c0 | 221 | 2 |
| comp60401c0 | 103 | 2 |
| comp60406c0 | 886 | 2 |
| comp60408c0 | 666 | 2 |
| comp60411c0 | 480 | 2 |
| comp60412c0 | 1918 | 2 |
| comp60416c0 | 824 | 2 |
| comp60418c0 | 1598 | 2 |
| comp60419c0 | 502 | 2 |
| comp60421c0 | 454 | 2 |
| comp60424c0 | 767 | 2 |
| comp60427c0 | 847 | 2 |
| comp60428c0 | 517 | 2 |
| comp60432c0 | 217 | 2 |
| comp60439c0 | 2208 | 2 |
| comp60442c0 | 703 | 2 |
| comp60456c0 | 454 | 2 |
| comp60457c0 | 99 | 2 |
| comp60460c0 | 873 | 2 |
| comp60467c0 | 1244 | 2 |
| comp60471c0 | 1170 | 2 |
| comp60482c0 | 569 | 2 |
| comp60490c0 | 1086 | 2 |
| comp60495c0 | 296 | 2 |
| comp60505c0 | 601 | 2 |
| comp60507c0 | 794 | 2 |
| comp60513c2 | 666 | 2 |
| comp60519c0 | 266 | 2 |
| comp60520c0 | 634 | 2 |
| comp60525c2 | 237 | 2 |
| comp60526c0 | 1141 | 2 |
| comp60527c0 | 183 | 2 |
| comp60538c0 | 1012 | 2 |
| comp60545c0 | 165 | 2 |
| comp60548c0 | 852 | 2 |
| comp60562c0 | 182 | 2 |
| comp60563c0 | 1215 | 2 |
| comp60565c0 | 312 | 2 |
| comp60567c0 | 1102 | 2 |
| comp60569c0 | 931 | 2 |
| comp60578c0 | 959 | 2 |
| comp60579c0 | 812 | 2 |
| comp60581c0 | 523 | 2 |
| comp60584c0 | 685 | 2 |
| comp60587c0 | 1616 | 2 |
| comp60588c0 | 1702 | 2 |
| comp60591c0 | 309 | 2 |
| comp60595c0 | 284 | 2 |
| comp60602c0 | 2549 | 2 |
| comp60603c0 | 597 | 2 |
| comp60605c0 | 599 | 2 |
| comp60622c0 | 223 | 2 |
| comp60637c0 | 328 | 2 |
| comp60638c0 | 2840 | 2 |
| comp60657c0 | 689 | 2 |
| comp60658c0 | 566 | 2 |
| comp60661c0 | 678 | 2 |
| comp60663c0 | 1521 | 2 |
| comp60673c0 | 922 | 2 |
| comp60676c0 | 402 | 2 |
| comp60677c0 | 216 | 2 |
| comp60690c1 | 69 | 2 |
| comp60691c0 | 159 | 2 |
| comp60697c0 | 648 | 2 |
| comp60703c0 | 571 | 2 |
| comp60719c0 | 636 | 2 |
| comp60731c0 | 177 | 2 |
| comp60732c0 | 1265 | 2 |
| comp60733c0 | 792 | 2 |
| comp60736c2 | 913 | 2 |
| comp60745c0 | 427 | 2 |
| comp60746c0 | 1180 | 2 |
| comp60747c0 | 727 | 2 |
| comp60749c0 | 765 | 2 |
| comp60763c0 | 1196 | 2 |
| comp60764c0 | 906 | 2 |
| comp60766c0 | 1044 | 2 |
| comp60769c0 | 906 | 2 |
| comp60772c0 | 31 | 2 |
| comp60773c0 | 163 | 2 |
| comp60784c0 | 973 | 2 |
| comp60786c0 | 239 | 2 |
| comp60789c0 | 1159 | 2 |
| comp60791c0 | 626 | 2 |
| comp60793c0 | 1121 | 2 |
| comp60797c0 | 289 | 2 |
| comp60806c0 | 537 | 2 |
| comp60823c0 | 456 | 2 |
| comp60829c0 | 242 | 2 |
| comp60830c0 | 1152 | 2 |
| comp60833c0 | 1295 | 2 |
| comp60837c0 | 320 | 2 |
| comp60848c0 | 691 | 2 |
| comp60851c0 | 2196 | 2 |
| comp60859c0 | 23 | 2 |
| comp60866c0 | 896 | 2 |
| comp60875c0 | 323 | 2 |
| comp60890c0 | 1648 | 2 |
| comp60891c0 | 870 | 2 |
| comp60893c0 | 1279 | 2 |
| comp60907c0 | 256 | 2 |
| comp60920c0 | 908 | 2 |
| comp60922c0 | 438 | 2 |
| comp60924c0 | 1322 | 2 |
| comp60926c0 | 31 | 2 |
| comp60935c0 | 724 | 2 |
| comp60941c0 | 1625 | 2 |
| comp60942c0 | 1101 | 2 |
| comp60944c0 | 232 | 2 |
| comp60945c0 | 416 | 2 |
| comp60948c0 | 1489 | 2 |
| comp60949c0 | 161 | 2 |
| comp60951c0 | 1229 | 2 |
| comp60954c0 | 559 | 2 |
| comp60954c1 | 274 | 2 |
| comp60955c0 | 601 | 2 |
| comp60964c1 | 344 | 2 |
| comp60972c0 | 1484 | 2 |
| comp60974c0 | 1393 | 2 |
| comp60975c0 | 1472 | 2 |
| comp60978c0 | 896 | 2 |
| comp60980c1 | 305 | 2 |
| comp60988c0 | 576 | 2 |
| comp60991c0 | 652 | 2 |
| comp60994c1 | 417 | 2 |
| comp60997c0 | 694 | 2 |
| comp61000c0 | 822 | 2 |
| comp61004c0 | 1078 | 2 |
| comp61016c0 | 724 | 2 |
| comp61016c1 | 74 | 2 |
| comp61022c0 | 45 | 2 |
| comp61025c0 | 450 | 2 |
| comp61031c0 | 1453 | 2 |
| comp61032c0 | 241 | 2 |
| comp61036c0 | 7379 | 2 |
| comp61039c0 | 875 | 2 |
| comp61044c0 | 944 | 2 |
| comp61063c0 | 405 | 2 |
| comp61069c0 | 939 | 2 |
| comp61071c0 | 406 | 2 |
| comp61072c0 | 760 | 2 |
| comp61080c0 | 824 | 2 |
| comp61081c0 | 350 | 2 |
| comp61083c0 | 1049 | 2 |
| comp61084c0 | 628 | 2 |
| comp61092c0 | 252 | 2 |
| comp61094c0 | 241 | 2 |
| comp61097c0 | 2659 | 2 |
| comp61102c0 | 886 | 2 |
| comp61121c1 | 767 | 2 |
| comp61130c0 | 515 | 2 |
| comp61134c0 | 569 | 2 |
| comp61136c0 | 835 | 2 |
| comp61141c0 | 1243 | 2 |
| comp61143c0 | 345 | 2 |
| comp61144c0 | 811 | 2 |
| comp61146c0 | 438 | 2 |
| comp61153c0 | 682 | 2 |
| comp61164c0 | 583 | 2 |
| comp61169c0 | 709 | 2 |
| comp61172c0 | 472 | 2 |
| comp61177c0 | 1177 | 2 |
| comp61190c0 | 714 | 2 |
| comp61192c0 | 923 | 2 |
| comp61205c0 | 735 | 2 |
| comp61207c0 | 135 | 2 |
| comp61214c0 | 764 | 2 |
| comp61215c0 | 526 | 2 |
| comp61216c0 | 1392 | 2 |
| comp61218c0 | 1088 | 2 |
| comp61222c0 | 962 | 2 |
| comp61231c0 | 909 | 2 |
| comp61241c0 | 838 | 2 |
| comp61264c0 | 550 | 2 |
| comp61266c0 | 1031 | 2 |
| comp61268c0 | 138 | 2 |
| comp61270c0 | 824 | 2 |
| comp61275c0 | 1112 | 2 |
| comp61277c0 | 302 | 2 |
| comp61282c0 | 494 | 2 |
| comp61285c0 | 709 | 2 |
| comp61286c2 | 182 | 2 |
| comp61292c0 | 106 | 2 |
| comp61302c0 | 820 | 2 |
| comp61307c0 | 809 | 2 |
| comp61310c0 | 929 | 2 |
| comp61312c0 | 23 | 2 |
| comp61318c0 | 1398 | 2 |
| comp61343c0 | 1015 | 2 |
| comp61344c0 | 395 | 2 |
| comp61345c0 | 889 | 2 |
| comp61353c0 | 226 | 2 |
| comp61364c0 | 1178 | 2 |
| comp61372c0 | 806 | 2 |
| comp61379c0 | 251 | 2 |
| comp61383c0 | 629 | 2 |
| comp61383c1 | 746 | 2 |
| comp61385c0 | 469 | 2 |
| comp61386c0 | 1016 | 2 |
| comp61390c0 | 790 | 2 |
| comp61392c1 | 898 | 2 |
| comp61396c0 | 1089 | 2 |
| comp61402c0 | 362 | 2 |
| comp61414c0 | 1800 | 2 |
| comp61415c0 | 942 | 2 |
| comp61420c0 | 2561 | 2 |
| comp61431c0 | 535 | 2 |
| comp61436c0 | 989 | 2 |
| comp61455c0 | 888 | 2 |
| comp61458c0 | 31 | 2 |
| comp61460c0 | 1244 | 2 |
| comp61463c0 | 1065 | 2 |
| comp61464c0 | 275 | 2 |
| comp61466c0 | 600 | 2 |
| comp61482c0 | 471 | 2 |
| comp61489c0 | 3275 | 2 |
| comp61497c0 | 556 | 2 |
| comp61502c0 | 813 | 2 |
| comp61503c0 | 537 | 2 |
| comp61504c0 | 674 | 2 |
| comp61509c0 | 2062 | 2 |
| comp61510c0 | 403 | 2 |
| comp61523c0 | 1239 | 2 |
| comp61533c0 | 127 | 2 |
| comp61534c0 | 346 | 2 |
| comp61534c1 | 1063 | 2 |
| comp61535c0 | 4175 | 2 |
| comp61550c0 | 551 | 2 |
| comp61558c0 | 726 | 2 |
| comp61560c0 | 957 | 2 |
| comp61570c0 | 704 | 2 |
| comp61574c0 | 1052 | 2 |
| comp61578c0 | 1767 | 2 |
| comp61582c0 | 827 | 2 |
| comp61585c0 | 310 | 2 |
| comp61586c0 | 544 | 2 |
| comp61590c0 | 460 | 2 |
| comp61591c0 | 1171 | 2 |
| comp61592c0 | 2003 | 2 |
| comp61595c1 | 1088 | 2 |
| comp61598c0 | 1847 | 2 |
| comp61601c2 | 504 | 2 |
| comp61614c0 | 68 | 2 |
| comp61620c0 | 464 | 2 |
| comp61627c0 | 838 | 2 |
| comp61632c0 | 883 | 2 |
| comp61634c0 | 1116 | 2 |
| comp61636c0 | 294 | 2 |
| comp61644c0 | 366 | 2 |
| comp61648c0 | 1122 | 2 |
| comp61651c0 | 318 | 2 |
| comp61656c0 | 1079 | 2 |
| comp61658c0 | 55 | 2 |
| comp61659c0 | 845 | 2 |
| comp61666c0 | 626 | 2 |
| comp61676c0 | 444 | 2 |
| comp61677c0 | 446 | 2 |
| comp61682c0 | 1739 | 2 |
| comp61684c0 | 339 | 2 |
| comp61705c0 | 668 | 2 |
| comp61715c0 | 1077 | 2 |
| comp61716c0 | 272 | 2 |
| comp61722c0 | 1241 | 2 |
| comp61730c0 | 179 | 2 |
| comp61738c0 | 1011 | 2 |
| comp61745c0 | 329 | 2 |
| comp61750c0 | 1037 | 2 |
| comp61754c0 | 622 | 2 |
| comp61756c0 | 644 | 2 |
| comp61763c0 | 342 | 2 |
| comp61769c0 | 648 | 2 |
| comp61776c0 | 959 | 2 |
| comp61787c0 | 667 | 2 |
| comp61795c0 | 1494 | 2 |
| comp61805c0 | 545 | 2 |
| comp61810c0 | 91 | 2 |
| comp61810c1 | 463 | 2 |
| comp61824c0 | 1201 | 2 |
| comp61826c0 | 2926 | 2 |
| comp61827c0 | 318 | 2 |
| comp61829c0 | 1377 | 2 |
| comp61831c0 | 239 | 2 |
| comp61832c0 | 1586 | 2 |
| comp61838c0 | 1333 | 2 |
| comp61859c0 | 736 | 2 |
| comp61861c0 | 621 | 2 |
| comp61868c0 | 667 | 2 |
| comp61883c0 | 1254 | 2 |
| comp61886c0 | 688 | 2 |
| comp61887c0 | 862 | 2 |
| comp61896c0 | 872 | 2 |
| comp61901c1 | 85 | 2 |
| comp61902c0 | 242 | 2 |
| comp61910c0 | 266 | 2 |
| comp61912c0 | 551 | 2 |
| comp61914c0 | 316 | 2 |
| comp61936c0 | 544 | 2 |
| comp61937c0 | 1250 | 2 |
| comp61941c0 | 517 | 2 |
| comp61951c0 | 536 | 2 |
| comp61968c0 | 983 | 2 |
| comp61974c0 | 1360 | 2 |
| comp61976c1 | 1468 | 2 |
| comp61977c0 | 3145 | 2 |
| comp61978c0 | 1138 | 2 |
| comp61981c0 | 917 | 2 |
| comp61986c0 | 116 | 2 |
| comp62002c0 | 753 | 2 |
| comp62006c1 | 328 | 2 |
| comp62010c0 | 935 | 2 |
| comp62014c0 | 241 | 2 |
| comp62015c0 | 648 | 2 |
| comp62023c0 | 608 | 2 |
| comp62026c0 | 820 | 2 |
| comp62031c0 | 716 | 2 |
| comp62038c0 | 40 | 2 |
| comp62056c0 | 577 | 2 |
| comp62060c0 | 344 | 2 |
| comp62061c1 | 235 | 2 |
| comp62077c0 | 323 | 2 |
| comp62081c0 | 1752 | 2 |
| comp62092c0 | 2041 | 2 |
| comp62094c0 | 1451 | 2 |
| comp62105c0 | 964 | 2 |
| comp62111c1 | 729 | 2 |
| comp62113c0 | 297 | 2 |
| comp62123c0 | 1812 | 2 |
| comp62124c0 | 342 | 2 |
| comp62128c0 | 212 | 2 |
| comp62149c0 | 589 | 2 |
| comp62155c0 | 2164 | 2 |
| comp62162c0 | 1036 | 2 |
| comp62164c0 | 500 | 2 |
| comp62171c0 | 761 | 2 |
| comp62172c0 | 1022 | 2 |
| comp62178c0 | 1027 | 2 |
| comp62181c0 | 1136 | 2 |
| comp62187c0 | 837 | 2 |
| comp62195c0 | 324 | 2 |
| comp62199c0 | 1291 | 2 |
| comp62207c0 | 1056 | 2 |
| comp62209c0 | 841 | 2 |
| comp62218c0 | 502 | 2 |
| comp62220c0 | 534 | 2 |
| comp62221c0 | 12 | 2 |
| comp62225c0 | 245 | 2 |
| comp62227c1 | 964 | 2 |
| comp62228c0 | 24 | 2 |
| comp62233c0 | 118 | 2 |
| comp62236c0 | 600 | 2 |
| comp62239c0 | 94 | 2 |
| comp62240c0 | 749 | 2 |
| comp62244c0 | 554 | 2 |
| comp62270c0 | 201 | 2 |
| comp62278c0 | 590 | 2 |
| comp62281c0 | 587 | 2 |
| comp62284c0 | 244 | 2 |
| comp62285c0 | 646 | 2 |
| comp62289c0 | 501 | 2 |
| comp62293c0 | 953 | 2 |
| comp62294c0 | 558 | 2 |
| comp62296c0 | 710 | 2 |
| comp62306c0 | 323 | 2 |
| comp62307c0 | 1265 | 2 |
| comp62309c0 | 2014 | 2 |
| comp62311c0 | 232 | 2 |
| comp62312c0 | 1225 | 2 |
| comp62314c0 | 775 | 2 |
| comp62316c0 | 920 | 2 |
| comp62317c0 | 1491 | 2 |
| comp62323c0 | 1279 | 2 |
| comp62328c0 | 1890 | 2 |
| comp62331c0 | 861 | 2 |
| comp62333c0 | 409 | 2 |
| comp62335c0 | 200 | 2 |
| comp62337c0 | 1105 | 2 |
| comp62339c0 | 83 | 2 |
| comp62355c0 | 108 | 2 |
| comp6235c0 | 645 | 2 |
| comp62362c0 | 406 | 2 |
| comp62365c0 | 1021 | 2 |
| comp62372c0 | 385 | 2 |
| comp62373c0 | 1041 | 2 |
| comp62379c1 | 1242 | 2 |
| comp62385c0 | 526 | 2 |
| comp62389c0 | 438 | 2 |
| comp62394c0 | 1748 | 2 |
| comp62403c0 | 253 | 2 |
| comp62403c1 | 845 | 2 |
| comp62417c1 | 690 | 2 |
| comp62421c0 | 773 | 2 |
| comp62424c0 | 1216 | 2 |
| comp62427c0 | 67 | 2 |
| comp62431c0 | 68 | 2 |
| comp62433c0 | 2175 | 2 |
| comp62434c0 | 117 | 2 |
| comp62440c0 | 180 | 2 |
| comp62442c0 | 24 | 2 |
| comp62446c0 | 664 | 2 |
| comp62459c0 | 715 | 2 |
| comp62463c0 | 739 | 2 |
| comp62464c0 | 2146 | 2 |
| comp62466c0 | 617 | 2 |
| comp62473c0 | 114 | 2 |
| comp62478c0 | 221 | 2 |
| comp62486c0 | 203 | 2 |
| comp62489c0 | 1801 | 2 |
| comp62495c0 | 293 | 2 |
| comp62496c0 | 221 | 2 |
| comp62500c0 | 900 | 2 |
| comp62503c0 | 1284 | 2 |
| comp62518c0 | 361 | 2 |
| comp62519c1 | 1507 | 2 |
| comp62521c0 | 218 | 2 |
| comp62527c0 | 615 | 2 |
| comp62533c0 | 365 | 2 |
| comp62539c0 | 343 | 2 |
| comp62540c0 | 1875 | 2 |
| comp62541c0 | 454 | 2 |
| comp62548c0 | 1930 | 2 |
| comp62549c0 | 651 | 2 |
| comp62551c0 | 430 | 2 |
| comp62553c0 | 813 | 2 |
| comp62557c0 | 1404 | 2 |
| comp62558c0 | 375 | 2 |
| comp62573c0 | 1234 | 2 |
| comp62587c0 | 470 | 2 |
| comp62589c0 | 521 | 2 |
| comp62590c0 | 912 | 2 |
| comp62602c0 | 1368 | 2 |
| comp62606c0 | 1724 | 2 |
| comp62608c0 | 691 | 2 |
| comp62609c0 | 304 | 2 |
| comp62624c0 | 172 | 2 |
| comp62628c0 | 364 | 2 |
| comp62629c0 | 446 | 2 |
| comp62638c0 | 141 | 2 |
| comp62642c0 | 1177 | 2 |
| comp62643c0 | 165 | 2 |
| comp62653c0 | 1572 | 2 |
| comp62666c0 | 1624 | 2 |
| comp62667c0 | 612 | 2 |
| comp62675c0 | 2536 | 2 |
| comp62678c0 | 1292 | 2 |
| comp62681c0 | 758 | 2 |
| comp62682c0 | 391 | 2 |
| comp62686c0 | 298 | 2 |
| comp62689c0 | 554 | 2 |
| comp62691c0 | 966 | 2 |
| comp62693c0 | 562 | 2 |
| comp62701c0 | 623 | 2 |
| comp62708c0 | 905 | 2 |
| comp62712c0 | 465 | 2 |
| comp62718c0 | 1105 | 2 |
| comp62733c0 | 1422 | 2 |
| comp62739c0 | 849 | 2 |
| comp62741c0 | 581 | 2 |
| comp62745c0 | 343 | 2 |
| comp62746c0 | 1278 | 2 |
| comp62757c2 | 147 | 2 |
| comp62761c0 | 862 | 2 |
| comp62764c0 | 1294 | 2 |
| comp62768c0 | 1040 | 2 |
| comp62775c1 | 593 | 2 |
| comp62776c0 | 200 | 2 |
| comp62787c0 | 114 | 2 |
| comp62794c0 | 199 | 2 |
| comp62795c0 | 1018 | 2 |
| comp62808c0 | 1012 | 2 |
| comp62819c0 | 300 | 2 |
| comp62820c0 | 1138 | 2 |
| comp62826c0 | 408 | 2 |
| comp62828c0 | 1012 | 2 |
| comp62833c0 | 3085 | 2 |
| comp62835c0 | 1596 | 2 |
| comp62841c0 | 1388 | 2 |
| comp62850c0 | 750 | 2 |
| comp62851c1 | 914 | 2 |
| comp62857c0 | 164 | 2 |
| comp62866c0 | 726 | 2 |
| comp62867c0 | 1084 | 2 |
| comp62868c0 | 577 | 2 |
| comp62874c0 | 192 | 2 |
| comp62880c0 | 641 | 2 |
| comp62887c0 | 794 | 2 |
| comp62888c0 | 581 | 2 |
| comp62889c0 | 955 | 2 |
| comp62896c0 | 1319 | 2 |
| comp62899c1 | 444 | 2 |
| comp62902c0 | 626 | 2 |
| comp62911c0 | 1103 | 2 |
| comp62913c0 | 107 | 2 |
| comp62917c0 | 900 | 2 |
| comp62928c0 | 481 | 2 |
| comp62936c0 | 1319 | 2 |
| comp62941c0 | 548 | 2 |
| comp62943c0 | 946 | 2 |
| comp62947c0 | 366 | 2 |
| comp62951c0 | 125 | 2 |
| comp62953c0 | 882 | 2 |
| comp62961c0 | 1012 | 2 |
| comp62964c0 | 1111 | 2 |
| comp62967c0 | 1114 | 2 |
| comp62973c0 | 49 | 2 |
| comp62979c0 | 691 | 2 |
| comp62984c0 | 267 | 2 |
| comp62989c0 | 1118 | 2 |
| comp62990c0 | 1180 | 2 |
| comp62992c0 | 491 | 2 |
| comp62996c1 | 188 | 2 |
| comp63018c0 | 195 | 2 |
| comp63044c0 | 1085 | 2 |
| comp63045c0 | 4 | 2 |
| comp63048c0 | 673 | 2 |
| comp63052c0 | 505 | 2 |
| comp63056c0 | 900 | 2 |
| comp63063c0 | 1500 | 2 |
| comp63064c0 | 958 | 2 |
| comp63065c0 | 1150 | 2 |
| comp63066c0 | 270 | 2 |
| comp63068c0 | 348 | 2 |
| comp63078c0 | 57 | 2 |
| comp63082c0 | 902 | 2 |
| comp63086c0 | 304 | 2 |
| comp63087c0 | 824 | 2 |
| comp63088c0 | 1255 | 2 |
| comp63096c0 | 1359 | 2 |
| comp63101c0 | 1051 | 2 |
| comp63106c0 | 1342 | 2 |
| comp63109c0 | 1501 | 2 |
| comp63112c0 | 67 | 2 |
| comp63118c0 | 605 | 2 |
| comp63121c0 | 546 | 2 |
| comp63122c0 | 701 | 2 |
| comp63132c0 | 732 | 2 |
| comp63144c0 | 932 | 2 |
| comp63152c0 | 1307 | 2 |
| comp63154c0 | 1004 | 2 |
| comp63156c0 | 1455 | 2 |
| comp63160c0 | 376 | 2 |
| comp63168c0 | 757 | 2 |
| comp63173c0 | 774 | 2 |
| comp63175c0 | 879 | 2 |
| comp6317c0 | 1412 | 2 |
| comp63182c0 | 670 | 2 |
| comp63192c0 | 346 | 2 |
| comp63196c0 | 685 | 2 |
| comp63200c0 | 1527 | 2 |
| comp63202c0 | 459 | 2 |
| comp63203c0 | 712 | 2 |
| comp63208c0 | 368 | 2 |
| comp63209c0 | 891 | 2 |
| comp63218c0 | 59 | 2 |
| comp63220c0 | 408 | 2 |
| comp63224c1 | 298 | 2 |
| comp63232c0 | 265 | 2 |
| comp63237c0 | 2179 | 2 |
| comp63238c0 | 1785 | 2 |
| comp63239c0 | 1166 | 2 |
| comp63241c0 | 926 | 2 |
| comp63243c0 | 288 | 2 |
| comp63248c0 | 1559 | 2 |
| comp63251c0 | 1769 | 2 |
| comp63252c1 | 344 | 2 |
| comp63255c0 | 212 | 2 |
| comp63257c0 | 355 | 2 |
| comp63258c0 | 1163 | 2 |
| comp63261c0 | 399 | 2 |
| comp63262c0 | 796 | 2 |
| comp63271c0 | 1316 | 2 |
| comp63272c0 | 723 | 2 |
| comp63275c0 | 1202 | 2 |
| comp63276c0 | 126 | 2 |
| comp63282c0 | 1290 | 2 |
| comp63286c0 | 1126 | 2 |
| comp63286c1 | 1011 | 2 |
| comp63288c0 | 876 | 2 |
| comp63305c0 | 1751 | 2 |
| comp63306c0 | 307 | 2 |
| comp63313c0 | 856 | 2 |
| comp63323c0 | 1016 | 2 |
| comp63337c0 | 22 | 2 |
| comp63343c0 | 702 | 2 |
| comp63345c0 | 1899 | 2 |
| comp63353c0 | 991 | 2 |
| comp63359c0 | 1027 | 2 |
| comp63363c0 | 510 | 2 |
| comp63367c0 | 195 | 2 |
| comp63376c2 | 606 | 2 |
| comp63378c0 | 480 | 2 |
| comp63379c0 | 453 | 2 |
| comp63387c0 | 427 | 2 |
| comp63388c0 | 527 | 2 |
| comp63393c0 | 69 | 2 |
| comp63397c0 | 481 | 2 |
| comp63399c0 | 947 | 2 |
| comp63406c0 | 464 | 2 |
| comp63411c0 | 1247 | 2 |
| comp63413c0 | 414 | 2 |
| comp63415c0 | 313 | 2 |
| comp63417c0 | 385 | 2 |
| comp63419c0 | 1162 | 2 |
| comp63421c0 | 301 | 2 |
| comp63423c0 | 644 | 2 |
| comp63427c0 | 612 | 2 |
| comp63431c0 | 2454 | 2 |
| comp63435c0 | 1639 | 2 |
| comp63437c0 | 982 | 2 |
| comp63442c0 | 954 | 2 |
| comp63443c0 | 512 | 2 |
| comp63452c0 | 1461 | 2 |
| comp63476c0 | 959 | 2 |
| comp63480c0 | 648 | 2 |
| comp63485c0 | 4687 | 2 |
| comp63487c0 | 1112 | 2 |
| comp63490c0 | 1393 | 2 |
| comp63491c0 | 860 | 2 |
| comp63498c0 | 271 | 2 |
| comp63522c0 | 517 | 2 |
| comp63523c0 | 580 | 2 |
| comp63526c0 | 752 | 2 |
| comp63531c0 | 649 | 2 |
| comp63540c0 | 2472 | 2 |
| comp63540c1 | 779 | 2 |
| comp63544c0 | 1025 | 2 |
| comp63551c0 | 449 | 2 |
| comp63555c0 | 657 | 2 |
| comp63558c0 | 817 | 2 |
| comp63559c0 | 8989 | 2 |
| comp63566c0 | 1102 | 2 |
| comp63574c0 | 202 | 2 |
| comp63576c0 | 334 | 2 |
| comp63586c0 | 521 | 2 |
| comp63587c0 | 541 | 2 |
| comp63592c0 | 383 | 2 |
| comp63594c0 | 125 | 2 |
| comp63595c0 | 79 | 2 |
| comp63598c0 | 921 | 2 |
| comp63605c0 | 1489 | 2 |
| comp63612c0 | 225 | 2 |
| comp63615c0 | 1037 | 2 |
| comp63616c0 | 796 | 2 |
| comp63618c0 | 859 | 2 |
| comp63628c0 | 606 | 2 |
| comp63638c0 | 459 | 2 |
| comp63640c0 | 1173 | 2 |
| comp63644c0 | 607 | 2 |
| comp63645c1 | 689 | 2 |
| comp63650c0 | 465 | 2 |
| comp63654c0 | 267 | 2 |
| comp63656c0 | 2607 | 2 |
| comp63659c0 | 49 | 2 |
| comp63662c0 | 391 | 2 |
| comp63667c0 | 944 | 2 |
| comp63669c0 | 2073 | 2 |
| comp63675c0 | 707 | 2 |
| comp63678c0 | 195 | 2 |
| comp63679c1 | 1367 | 2 |
| comp63680c0 | 746 | 2 |
| comp63690c0 | 456 | 2 |
| comp63691c0 | 403 | 2 |
| comp63693c0 | 216 | 2 |
| comp63694c0 | 260 | 2 |
| comp63696c0 | 1684 | 2 |
| comp63702c0 | 552 | 2 |
| comp63711c0 | 434 | 2 |
| comp63726c0 | 334 | 2 |
| comp63726c1 | 29 | 2 |
| comp63727c0 | 586 | 2 |
| comp63728c0 | 1525 | 2 |
| comp63729c0 | 992 | 2 |
| comp63733c0 | 1214 | 2 |
| comp63737c0 | 1341 | 2 |
| comp63738c0 | 340 | 2 |
| comp63739c0 | 1969 | 2 |
| comp63740c0 | 447 | 2 |
| comp63741c0 | 642 | 2 |
| comp63743c0 | 1586 | 2 |
| comp63747c0 | 575 | 2 |
| comp63749c0 | 870 | 2 |
| comp63753c0 | 1106 | 2 |
| comp63760c0 | 1400 | 2 |
| comp63762c0 | 1090 | 2 |
| comp63766c0 | 523 | 2 |
| comp63768c0 | 353 | 2 |
| comp63776c0 | 1120 | 2 |
| comp63784c0 | 695 | 2 |
| comp63788c0 | 1309 | 2 |
| comp63790c0 | 275 | 2 |
| comp63791c0 | 408 | 2 |
| comp63793c0 | 375 | 2 |
| comp63794c0 | 721 | 2 |
| comp63801c0 | 199 | 2 |
| comp63803c0 | 456 | 2 |
| comp63811c0 | 1495 | 2 |
| comp63819c0 | 714 | 2 |
| comp638200c0 | 357 | 2 |
| comp63820c0 | 1394 | 2 |
| comp63824c0 | 383 | 2 |
| comp63833c0 | 1525 | 2 |
| comp63839c0 | 2148 | 2 |
| comp63842c0 | 626 | 2 |
| comp63843c0 | 547 | 2 |
| comp63845c0 | 763 | 2 |
| comp63848c0 | 617 | 2 |
| comp63850c0 | 838 | 2 |
| comp63858c0 | 467 | 2 |
| comp63863c0 | 1073 | 2 |
| comp63865c0 | 249 | 2 |
| comp63866c0 | 283 | 2 |
| comp63870c0 | 953 | 2 |
| comp63872c1 | 734 | 2 |
| comp63874c0 | 815 | 2 |
| comp63878c0 | 898 | 2 |
| comp63880c0 | 624 | 2 |
| comp63883c0 | 421 | 2 |
| comp63884c0 | 370 | 2 |
| comp63885c0 | 97 | 2 |
| comp63889c0 | 640 | 2 |
| comp63890c0 | 874 | 2 |
| comp63891c0 | 303 | 2 |
| comp63891c1 | 756 | 2 |
| comp63892c0 | 458 | 2 |
| comp63893c0 | 117 | 2 |
| comp63898c0 | 332 | 2 |
| comp63901c0 | 2080 | 2 |
| comp63904c0 | 573 | 2 |
| comp63906c0 | 900 | 2 |
| comp63914c0 | 516 | 2 |
| comp63918c0 | 132 | 2 |
| comp63919c0 | 317 | 2 |
| comp63923c0 | 686 | 2 |
| comp63925c1 | 596 | 2 |
| comp63931c0 | 1564 | 2 |
| comp63932c0 | 582 | 2 |
| comp63933c0 | 1120 | 2 |
| comp63939c0 | 568 | 2 |
| comp63943c0 | 816 | 2 |
| comp63943c1 | 338 | 2 |
| comp63944c0 | 211 | 2 |
| comp63948c0 | 192 | 2 |
| comp63949c0 | 594 | 2 |
| comp63952c0 | 1186 | 2 |
| comp63954c0 | 302 | 2 |
| comp63963c0 | 271 | 2 |
| comp63967c0 | 566 | 2 |
| comp63977c0 | 357 | 2 |
| comp63978c0 | 1117 | 2 |
| comp63981c0 | 1284 | 2 |
| comp63982c0 | 1223 | 2 |
| comp63986c0 | 636 | 2 |
| comp63997c0 | 211 | 2 |
| comp63999c0 | 293 | 2 |
| comp64008c0 | 2523 | 2 |
| comp64014c0 | 926 | 2 |
| comp64019c0 | 864 | 2 |
| comp64020c1 | 707 | 2 |
| comp64022c0 | 912 | 2 |
| comp64025c0 | 1503 | 2 |
| comp64027c0 | 868 | 2 |
| comp64031c0 | 1492 | 2 |
| comp64034c0 | 795 | 2 |
| comp64035c0 | 199 | 2 |
| comp64037c0 | 42 | 2 |
| comp64038c0 | 627 | 2 |
| comp64040c0 | 919 | 2 |
| comp64043c0 | 616 | 2 |
| comp64046c0 | 363 | 2 |
| comp64049c0 | 1005 | 2 |
| comp64050c0 | 488 | 2 |
| comp64053c0 | 72 | 2 |
| comp64055c0 | 894 | 2 |
| comp64065c0 | 241 | 2 |
| comp64078c0 | 166 | 2 |
| comp64083c0 | 905 | 2 |
| comp64087c0 | 504 | 2 |
| comp64089c0 | 191 | 2 |
| comp64091c0 | 989 | 2 |
| comp64095c0 | 2091 | 2 |
| comp64101c0 | 918 | 2 |
| comp64111c0 | 561 | 2 |
| comp64113c0 | 828 | 2 |
| comp64119c0 | 1443 | 2 |
| comp64130c0 | 748 | 2 |
| comp64133c0 | 1125 | 2 |
| comp64141c0 | 647 | 2 |
| comp64143c0 | 420 | 2 |
| comp64153c0 | 1560 | 2 |
| comp64154c0 | 423 | 2 |
| comp64164c0 | 1587 | 2 |
| comp64178c0 | 108 | 2 |
| comp64183c0 | 1443 | 2 |
| comp64190c0 | 159 | 2 |
| comp64191c0 | 1002 | 2 |
| comp64196c0 | 608 | 2 |
| comp64197c0 | 769 | 2 |
| comp64207c1 | 749 | 2 |
| comp64208c0 | 1268 | 2 |
| comp64209c1 | 84 | 2 |
| comp64210c0 | 1199 | 2 |
| comp64222c0 | 896 | 2 |
| comp64232c0 | 1674 | 2 |
| comp64238c0 | 78 | 2 |
| comp64246c0 | 269 | 2 |
| comp64249c0 | 314 | 2 |
| comp64262c0 | 463 | 2 |
| comp64265c0 | 1085 | 2 |
| comp64266c0 | 150 | 2 |
| comp64268c0 | 760 | 2 |
| comp64274c0 | 268 | 2 |
| comp64279c0 | 765 | 2 |
| comp64280c0 | 649 | 2 |
| comp64282c0 | 60 | 2 |
| comp64286c0 | 87 | 2 |
| comp64287c0 | 695 | 2 |
| comp64288c0 | 1127 | 2 |
| comp64290c0 | 418 | 2 |
| comp64294c0 | 793 | 2 |
| comp64298c0 | 160 | 2 |
| comp64303c0 | 91 | 2 |
| comp64309c0 | 943 | 2 |
| comp64312c0 | 887 | 2 |
| comp64316c0 | 421 | 2 |
| comp64319c0 | 1314 | 2 |
| comp64324c0 | 856 | 2 |
| comp64328c0 | 680 | 2 |
| comp64334c0 | 1246 | 2 |
| comp64337c1 | 876 | 2 |
| comp64341c1 | 1061 | 2 |
| comp64351c0 | 494 | 2 |
| comp64355c0 | 287 | 2 |
| comp64358c0 | 735 | 2 |
| comp64361c0 | 1480 | 2 |
| comp64367c0 | 513 | 2 |
| comp64376c0 | 1064 | 2 |
| comp64381c0 | 714 | 2 |
| comp64383c0 | 1639 | 2 |
| comp64386c0 | 849 | 2 |
| comp64390c0 | 584 | 2 |
| comp64393c0 | 1295 | 2 |
| comp64394c0 | 856 | 2 |
| comp64397c0 | 1533 | 2 |
| comp64402c0 | 531 | 2 |
| comp64407c0 | 312 | 2 |
| comp64410c0 | 1124 | 2 |
| comp64411c1 | 491 | 2 |
| comp64415c0 | 955 | 2 |
| comp64419c0 | 724 | 2 |
| comp64423c1 | 915 | 2 |
| comp64425c0 | 951 | 2 |
| comp64428c0 | 1701 | 2 |
| comp64431c1 | 529 | 2 |
| comp64441c0 | 220 | 2 |
| comp64443c1 | 162 | 2 |
| comp64449c0 | 1004 | 2 |
| comp64450c0 | 1317 | 2 |
| comp64457c0 | 28 | 2 |
| comp64468c0 | 1853 | 2 |
| comp64469c0 | 557 | 2 |
| comp64470c0 | 446 | 2 |
| comp64473c0 | 280 | 2 |
| comp64477c0 | 896 | 2 |
| comp64478c0 | 450 | 2 |
| comp64483c0 | 1412 | 2 |
| comp64495c0 | 541 | 2 |
| comp64497c0 | 1102 | 2 |
| comp64500c0 | 497 | 2 |
| comp64501c0 | 1731 | 2 |
| comp64508c0 | 1420 | 2 |
| comp64509c0 | 806 | 2 |
| comp64509c1 | 562 | 2 |
| comp64514c2 | 1510 | 2 |
| comp64518c0 | 362 | 2 |
| comp64521c0 | 545 | 2 |
| comp64525c0 | 839 | 2 |
| comp64528c0 | 891 | 2 |
| comp64534c0 | 162 | 2 |
| comp64541c0 | 323 | 2 |
| comp64552c0 | 436 | 2 |
| comp64561c0 | 613 | 2 |
| comp64562c0 | 470 | 2 |
| comp64568c0 | 662 | 2 |
| comp64571c0 | 842 | 2 |
| comp64576c0 | 1251 | 2 |
| comp64578c0 | 419 | 2 |
| comp64582c0 | 671 | 2 |
| comp64587c0 | 752 | 2 |
| comp64588c0 | 810 | 2 |
| comp64589c0 | 4 | 2 |
| comp64590c0 | 166 | 2 |
| comp64593c0 | 805 | 2 |
| comp64605c2 | 320 | 2 |
| comp64606c0 | 1567 | 2 |
| comp64607c0 | 611 | 2 |
| comp64618c0 | 1255 | 2 |
| comp64619c0 | 569 | 2 |
| comp64621c0 | 607 | 2 |
| comp64625c0 | 1207 | 2 |
| comp64627c0 | 1681 | 2 |
| comp64640c1 | 481 | 2 |
| comp64641c0 | 834 | 2 |
| comp64644c0 | 397 | 2 |
| comp64646c0 | 201 | 2 |
| comp64650c0 | 114 | 2 |
| comp64651c0 | 729 | 2 |
| comp64661c0 | 214 | 2 |
| comp64665c0 | 1199 | 2 |
| comp64667c0 | 584 | 2 |
| comp64670c0 | 140 | 2 |
| comp64671c0 | 711 | 2 |
| comp64675c0 | 716 | 2 |
| comp64690c0 | 1343 | 2 |
| comp64698c0 | 1230 | 2 |
| comp64704c0 | 317 | 2 |
| comp64706c0 | 889 | 2 |
| comp64709c0 | 1110 | 2 |
| comp64718c0 | 516 | 2 |
| comp64724c0 | 184 | 2 |
| comp64734c0 | 977 | 2 |
| comp64735c0 | 217 | 2 |
| comp64739c0 | 1143 | 2 |
| comp64742c0 | 939 | 2 |
| comp64746c0 | 827 | 2 |
| comp64748c0 | 43 | 2 |
| comp64752c1 | 1487 | 2 |
| comp64759c0 | 558 | 2 |
| comp64761c1 | 350 | 2 |
| comp64769c0 | 1359 | 2 |
| comp64771c0 | 495 | 2 |
| comp64773c0 | 480 | 2 |
| comp64778c0 | 1162 | 2 |
| comp64781c1 | 1015 | 2 |
| comp64782c0 | 644 | 2 |
| comp64794c0 | 1597 | 2 |
| comp64796c0 | 781 | 2 |
| comp64811c0 | 332 | 2 |
| comp64814c0 | 910 | 2 |
| comp64820c0 | 273 | 2 |
| comp64823c0 | 78 | 2 |
| comp64833c0 | 668 | 2 |
| comp64839c0 | 354 | 2 |
| comp64848c0 | 914 | 2 |
| comp64857c0 | 907 | 2 |
| comp64869c0 | 686 | 2 |
| comp64870c0 | 631 | 2 |
| comp64872c0 | 446 | 2 |
| comp64879c0 | 3371 | 2 |
| comp64882c0 | 235 | 2 |
| comp64882c1 | 573 | 2 |
| comp64890c0 | 355 | 2 |
| comp64896c0 | 1535 | 2 |
| comp64898c0 | 649 | 2 |
| comp64900c0 | 805 | 2 |
| comp64902c0 | 645 | 2 |
| comp64903c0 | 52 | 2 |
| comp64909c0 | 270 | 2 |
| comp64918c0 | 749 | 2 |
| comp64922c0 | 546 | 2 |
| comp64925c0 | 470 | 2 |
| comp64927c0 | 458 | 2 |
| comp64935c0 | 1838 | 2 |
| comp64940c0 | 78 | 2 |
| comp64942c0 | 1368 | 2 |
| comp64945c0 | 1085 | 2 |
| comp64948c0 | 467 | 2 |
| comp64949c1 | 414 | 2 |
| comp64962c0 | 1752 | 2 |
| comp64963c0 | 380 | 2 |
| comp64966c0 | 867 | 2 |
| comp64970c0 | 153 | 2 |
| comp64971c0 | 215 | 2 |
| comp64975c0 | 828 | 2 |
| comp64979c0 | 75 | 2 |
| comp64981c0 | 887 | 2 |
| comp64989c0 | 69 | 2 |
| comp65004c0 | 1455 | 2 |
| comp65006c0 | 1007 | 2 |
| comp65008c0 | 988 | 2 |
| comp65011c0 | 1188 | 2 |
| comp65013c0 | 2135 | 2 |
| comp65023c0 | 1283 | 2 |
| comp65030c0 | 1839 | 2 |
| comp65033c0 | 193 | 2 |
| comp65036c0 | 148 | 2 |
| comp65041c0 | 376 | 2 |
| comp65042c0 | 77 | 2 |
| comp65043c0 | 346 | 2 |
| comp65045c0 | 391 | 2 |
| comp65050c0 | 852 | 2 |
| comp65051c0 | 283 | 2 |
| comp65053c0 | 724 | 2 |
| comp65062c0 | 1605 | 2 |
| comp65064c0 | 1239 | 2 |
| comp65065c1 | 396 | 2 |
| comp65067c0 | 673 | 2 |
| comp65070c0 | 361 | 2 |
| comp65075c0 | 930 | 2 |
| comp65081c0 | 1660 | 2 |
| comp65082c0 | 2058 | 2 |
| comp65093c0 | 2692 | 2 |
| comp65095c0 | 984 | 2 |
| comp65112c0 | 285 | 2 |
| comp65117c0 | 195 | 2 |
| comp65127c0 | 676 | 2 |
| comp65129c0 | 673 | 2 |
| comp65130c1 | 1162 | 2 |
| comp65132c0 | 1536 | 2 |
| comp65133c0 | 1437 | 2 |
| comp65137c0 | 1304 | 2 |
| comp65142c0 | 1251 | 2 |
| comp65144c0 | 865 | 2 |
| comp65157c0 | 2770 | 2 |
| comp65160c0 | 710 | 2 |
| comp65165c0 | 1432 | 2 |
| comp65168c0 | 419 | 2 |
| comp65171c0 | 1438 | 2 |
| comp65174c0 | 1373 | 2 |
| comp65175c0 | 46 | 2 |
| comp65177c0 | 151 | 2 |
| comp65180c0 | 1100 | 2 |
| comp65183c0 | 378 | 2 |
| comp65194c0 | 1012 | 2 |
| comp65220c0 | 328 | 2 |
| comp65224c0 | 1282 | 2 |
| comp65226c0 | 1000 | 2 |
| comp65234c0 | 32824 | 2 |
| comp65236c0 | 176 | 2 |
| comp65249c0 | 1418 | 2 |
| comp65253c0 | 1016 | 2 |
| comp65262c0 | 1287 | 2 |
| comp65263c0 | 162 | 2 |
| comp65264c0 | 1728 | 2 |
| comp65265c0 | 1198 | 2 |
| comp65268c0 | 696 | 2 |
| comp65272c0 | 823 | 2 |
| comp65278c0 | 377 | 2 |
| comp65284c0 | 369 | 2 |
| comp65291c0 | 134 | 2 |
| comp65295c0 | 885 | 2 |
| comp65297c0 | 821 | 2 |
| comp65299c0 | 1156 | 2 |
| comp65302c0 | 2471 | 2 |
| comp65320c0 | 695 | 2 |
| comp65322c0 | 874 | 2 |
| comp65324c0 | 495 | 2 |
| comp65329c0 | 262 | 2 |
| comp65335c1 | 1597 | 2 |
| comp65336c0 | 1272 | 2 |
| comp65336c1 | 607 | 2 |
| comp65337c0 | 1348 | 2 |
| comp65342c1 | 247 | 2 |
| comp65344c0 | 1526 | 2 |
| comp65345c0 | 748 | 2 |
| comp65346c0 | 242 | 2 |
| comp65368c0 | 774 | 2 |
| comp6536c0 | 974 | 2 |
| comp65377c0 | 270 | 2 |
| comp65381c0 | 222 | 2 |
| comp65384c0 | 1834 | 2 |
| comp65387c0 | 1179 | 2 |
| comp65393c0 | 336 | 2 |
| comp65397c0 | 82 | 2 |
| comp65400c0 | 352 | 2 |
| comp65401c0 | 1225 | 2 |
| comp65403c0 | 327 | 2 |
| comp65407c0 | 623 | 2 |
| comp65410c0 | 1008 | 2 |
| comp65411c0 | 479 | 2 |
| comp65413c0 | 756 | 2 |
| comp65415c0 | 1438 | 2 |
| comp65422c0 | 729 | 2 |
| comp65424c0 | 653 | 2 |
| comp65427c0 | 483 | 2 |
| comp65429c0 | 569 | 2 |
| comp65434c0 | 345 | 2 |
| comp65436c0 | 904 | 2 |
| comp65439c0 | 1075 | 2 |
| comp65441c0 | 415 | 2 |
| comp65445c0 | 211 | 2 |
| comp65455c0 | 99 | 2 |
| comp65463c0 | 253 | 2 |
| comp65465c0 | 1071 | 2 |
| comp65467c0 | 1611 | 2 |
| comp65471c0 | 641 | 2 |
| comp65473c0 | 502 | 2 |
| comp65476c1 | 806 | 2 |
| comp65477c0 | 983 | 2 |
| comp65484c0 | 791 | 2 |
| comp65487c0 | 819 | 2 |
| comp65489c0 | 918 | 2 |
| comp65492c0 | 144 | 2 |
| comp65499c0 | 146 | 2 |
| comp65502c0 | 738 | 2 |
| comp65503c0 | 1429 | 2 |
| comp65505c0 | 1544 | 2 |
| comp65506c0 | 748 | 2 |
| comp65509c0 | 1020 | 2 |
| comp65511c0 | 795 | 2 |
| comp65514c0 | 115 | 2 |
| comp65515c0 | 1516 | 2 |
| comp65520c0 | 950 | 2 |
| comp65524c1 | 693 | 2 |
| comp65533c1 | 238 | 2 |
| comp65541c0 | 359 | 2 |
| comp65546c0 | 192 | 2 |
| comp65548c0 | 640 | 2 |
| comp65556c1 | 836 | 2 |
| comp65558c0 | 944 | 2 |
| comp65567c0 | 1235 | 2 |
| comp65572c0 | 1704 | 2 |
| comp65573c0 | 456 | 2 |
| comp65578c0 | 1020 | 2 |
| comp65587c0 | 2584 | 2 |
| comp65597c0 | 272 | 2 |
| comp65598c0 | 1418 | 2 |
| comp65600c0 | 573 | 2 |
| comp65606c0 | 1013 | 2 |
| comp65622c0 | 1125 | 2 |
| comp65630c0 | 2127 | 2 |
| comp65641c0 | 2089 | 2 |
| comp65650c0 | 721 | 2 |
| comp65651c0 | 1293 | 2 |
| comp65652c0 | 874 | 2 |
| comp65654c2 | 103 | 2 |
| comp65655c0 | 684 | 2 |
| comp65661c0 | 1187 | 2 |
| comp65669c0 | 940 | 2 |
| comp65674c0 | 1214 | 2 |
| comp65676c0 | 516 | 2 |
| comp65679c0 | 1420 | 2 |
| comp65688c0 | 1257 | 2 |
| comp65693c0 | 2118 | 2 |
| comp65697c0 | 286 | 2 |
| comp65703c0 | 1286 | 2 |
| comp65705c0 | 704 | 2 |
| comp65706c0 | 223 | 2 |
| comp65713c0 | 853 | 2 |
| comp65716c0 | 999 | 2 |
| comp65717c0 | 1174 | 2 |
| comp65725c0 | 250 | 2 |
| comp65726c0 | 446 | 2 |
| comp65733c0 | 445 | 2 |
| comp65735c0 | 1315 | 2 |
| comp65736c0 | 157 | 2 |
| comp65738c0 | 393 | 2 |
| comp65742c0 | 1005 | 2 |
| comp65744c0 | 977 | 2 |
| comp65749c0 | 31 | 2 |
| comp65756c0 | 389 | 2 |
| comp65757c0 | 761 | 2 |
| comp65759c0 | 319 | 2 |
| comp65760c0 | 181 | 2 |
| comp65762c0 | 1173 | 2 |
| comp65763c0 | 197 | 2 |
| comp65771c1 | 1526 | 2 |
| comp65772c0 | 230 | 2 |
| comp65775c0 | 340 | 2 |
| comp65782c0 | 244 | 2 |
| comp65784c0 | 671 | 2 |
| comp65785c0 | 1575 | 2 |
| comp65791c0 | 462 | 2 |
| comp65793c0 | 1068 | 2 |
| comp65794c0 | 82 | 2 |
| comp65805c0 | 224 | 2 |
| comp65806c0 | 1469 | 2 |
| comp65814c0 | 898 | 2 |
| comp65815c0 | 291 | 2 |
| comp65820c0 | 917 | 2 |
| comp65825c0 | 555 | 2 |
| comp65834c0 | 1018 | 2 |
| comp65840c0 | 754 | 2 |
| comp65841c0 | 744 | 2 |
| comp65848c0 | 1251 | 2 |
| comp65860c0 | 50 | 2 |
| comp65873c0 | 805 | 2 |
| comp65883c0 | 388 | 2 |
| comp65884c0 | 451 | 2 |
| comp65888c0 | 1838 | 2 |
| comp65896c0 | 1152 | 2 |
| comp65897c0 | 1137 | 2 |
| comp65897c1 | 98 | 2 |
| comp65900c0 | 272 | 2 |
| comp65903c0 | 189 | 2 |
| comp65908c0 | 816 | 2 |
| comp65912c1 | 2714 | 2 |
| comp65914c0 | 637 | 2 |
| comp65918c0 | 388 | 2 |
| comp65919c0 | 1537 | 2 |
| comp65921c0 | 978 | 2 |
| comp65931c0 | 1675 | 2 |
| comp65932c0 | 651 | 2 |
| comp65938c0 | 1118 | 2 |
| comp65941c0 | 373 | 2 |
| comp65945c0 | 1173 | 2 |
| comp65947c0 | 329 | 2 |
| comp65956c0 | 1365 | 2 |
| comp65960c0 | 1006 | 2 |
| comp65962c0 | 212 | 2 |
| comp65970c0 | 115 | 2 |
| comp65978c0 | 808 | 2 |
| comp65979c0 | 571 | 2 |
| comp65983c0 | 382 | 2 |
| comp65985c0 | 727 | 2 |
| comp65986c2 | 610 | 2 |
| comp65989c0 | 556 | 2 |
| comp65994c0 | 523 | 2 |
| comp65995c0 | 811 | 2 |
| comp65996c0 | 527 | 2 |
| comp65997c0 | 312 | 2 |
| comp66002c0 | 1625 | 2 |
| comp66006c0 | 916 | 2 |
| comp66009c0 | 510 | 2 |
| comp66011c0 | 973 | 2 |
| comp66013c0 | 552 | 2 |
| comp66016c0 | 1850 | 2 |
| comp66031c0 | 287 | 2 |
| comp66038c0 | 891 | 2 |
| comp66042c0 | 1610 | 2 |
| comp66049c0 | 1244 | 2 |
| comp66051c0 | 837 | 2 |
| comp66062c1 | 442 | 2 |
| comp66066c1 | 629 | 2 |
| comp66069c0 | 1731 | 2 |
| comp66077c0 | 145 | 2 |
| comp66081c0 | 928 | 2 |
| comp66084c0 | 825 | 2 |
| comp66087c0 | 232 | 2 |
| comp66094c0 | 135 | 2 |
| comp66095c0 | 811 | 2 |
| comp66100c1 | 1861 | 2 |
| comp66114c0 | 308 | 2 |
| comp66115c0 | 196 | 2 |
| comp66119c0 | 805 | 2 |
| comp66126c0 | 734 | 2 |
| comp66127c0 | 493 | 2 |
| comp66134c0 | 836 | 2 |
| comp66140c0 | 531 | 2 |
| comp66143c0 | 892 | 2 |
| comp66145c0 | 171 | 2 |
| comp66146c1 | 1183 | 2 |
| comp66153c0 | 1174 | 2 |
| comp66157c0 | 682 | 2 |
| comp66161c0 | 251 | 2 |
| comp66164c0 | 1033 | 2 |
| comp66175c0 | 806 | 2 |
| comp66176c0 | 503 | 2 |
| comp66177c0 | 222 | 2 |
| comp66188c0 | 1404 | 2 |
| comp66189c0 | 2547 | 2 |
| comp66192c0 | 2117 | 2 |
| comp66193c0 | 598 | 2 |
| comp66195c0 | 385 | 2 |
| comp66203c0 | 352 | 2 |
| comp66205c0 | 984 | 2 |
| comp66209c0 | 537 | 2 |
| comp66212c0 | 1499 | 2 |
| comp66217c1 | 1623 | 2 |
| comp66217c2 | 982 | 2 |
| comp66219c0 | 613 | 2 |
| comp66229c0 | 650 | 2 |
| comp66230c0 | 771 | 2 |
| comp66233c0 | 514 | 2 |
| comp66237c0 | 993 | 2 |
| comp66239c0 | 389 | 2 |
| comp66241c0 | 739 | 2 |
| comp66249c0 | 1616 | 2 |
| comp66254c0 | 508 | 2 |
| comp66262c0 | 733 | 2 |
| comp66266c0 | 241 | 2 |
| comp66267c0 | 1603 | 2 |
| comp66268c0 | 263 | 2 |
| comp66271c1 | 692 | 2 |
| comp66276c0 | 972 | 2 |
| comp66284c0 | 182 | 2 |
| comp66290c0 | 668 | 2 |
| comp66291c0 | 454 | 2 |
| comp66304c0 | 940 | 2 |
| comp66308c0 | 799 | 2 |
| comp66315c0 | 460 | 2 |
| comp66316c0 | 840 | 2 |
| comp66327c0 | 874 | 2 |
| comp66333c0 | 333 | 2 |
| comp66337c0 | 506 | 2 |
| comp66340c0 | 506 | 2 |
| comp66346c0 | 218 | 2 |
| comp66348c2 | 1126 | 2 |
| comp66352c0 | 462 | 2 |
| comp66354c0 | 684 | 2 |
| comp66355c0 | 461 | 2 |
| comp66357c0 | 719 | 2 |
| comp66358c0 | 189 | 2 |
| comp66360c0 | 657 | 2 |
| comp66361c0 | 1480 | 2 |
| comp66365c0 | 260 | 2 |
| comp66366c0 | 751 | 2 |
| comp66381c0 | 308 | 2 |
| comp66383c0 | 293 | 2 |
| comp66385c1 | 645 | 2 |
| comp66390c0 | 549 | 2 |
| comp66398c0 | 711 | 2 |
| comp66401c0 | 896 | 2 |
| comp66404c0 | 433 | 2 |
| comp66413c0 | 675 | 2 |
| comp66418c0 | 1100 | 2 |
| comp66420c0 | 402 | 2 |
| comp66432c0 | 442 | 2 |
| comp66438c0 | 470 | 2 |
| comp66439c0 | 748 | 2 |
| comp66447c0 | 818 | 2 |
| comp66449c0 | 363 | 2 |
| comp66456c0 | 2479 | 2 |
| comp66457c0 | 326 | 2 |
| comp66459c0 | 629 | 2 |
| comp66461c0 | 2740 | 2 |
| comp66473c0 | 2124 | 2 |
| comp66476c0 | 747 | 2 |
| comp66483c0 | 1591 | 2 |
| comp66484c0 | 1075 | 2 |
| comp66487c0 | 456 | 2 |
| comp66488c0 | 551 | 2 |
| comp66498c0 | 320 | 2 |
| comp66499c0 | 405 | 2 |
| comp66510c0 | 821 | 2 |
| comp66511c0 | 80 | 2 |
| comp66513c0 | 638 | 2 |
| comp66514c0 | 775 | 2 |
| comp66520c0 | 2029 | 2 |
| comp66522c0 | 668 | 2 |
| comp66524c0 | 435 | 2 |
| comp66527c0 | 245 | 2 |
| comp66528c0 | 440 | 2 |
| comp66531c0 | 310 | 2 |
| comp66536c0 | 388 | 2 |
| comp66539c0 | 98 | 2 |
| comp66540c0 | 410 | 2 |
| comp66543c0 | 1473 | 2 |
| comp66551c0 | 1147 | 2 |
| comp66553c0 | 985 | 2 |
| comp66554c1 | 2406 | 2 |
| comp66561c0 | 579 | 2 |
| comp66562c0 | 2580 | 2 |
| comp66565c0 | 1362 | 2 |
| comp66567c0 | 165 | 2 |
| comp66571c0 | 823 | 2 |
| comp66571c1 | 616 | 2 |
| comp66575c0 | 1078 | 2 |
| comp66576c1 | 586 | 2 |
| comp66580c0 | 180 | 2 |
| comp66587c0 | 1229 | 2 |
| comp66590c0 | 695 | 2 |
| comp66596c0 | 633 | 2 |
| comp66597c0 | 609 | 2 |
| comp66600c0 | 1630 | 2 |
| comp66602c0 | 77 | 2 |
| comp66604c1 | 552 | 2 |
| comp66606c0 | 191 | 2 |
| comp66609c1 | 160 | 2 |
| comp66612c0 | 1215 | 2 |
| comp66614c0 | 129 | 2 |
| comp66617c0 | 889 | 2 |
| comp66621c0 | 1021 | 2 |
| comp66623c0 | 798 | 2 |
| comp66627c0 | 376 | 2 |
| comp66628c0 | 437 | 2 |
| comp66638c0 | 542 | 2 |
| comp66642c0 | 686 | 2 |
| comp66646c1 | 994 | 2 |
| comp66648c0 | 796 | 2 |
| comp66655c0 | 204 | 2 |
| comp66657c0 | 169 | 2 |
| comp66667c0 | 827 | 2 |
| comp66668c0 | 450 | 2 |
| comp66675c0 | 801 | 2 |
| comp66680c0 | 335 | 2 |
| comp66681c3 | 967 | 2 |
| comp66685c4 | 1492 | 2 |
| comp66686c0 | 795 | 2 |
| comp66697c0 | 535 | 2 |
| comp66701c1 | 304 | 2 |
| comp66704c0 | 1043 | 2 |
| comp66708c0 | 403 | 2 |
| comp66719c0 | 935 | 2 |
| comp66727c0 | 323 | 2 |
| comp66728c0 | 78 | 2 |
| comp66731c0 | 1421 | 2 |
| comp66733c0 | 920 | 2 |
| comp66737c0 | 793 | 2 |
| comp66745c0 | 1145 | 2 |
| comp66749c0 | 372 | 2 |
| comp66750c0 | 369 | 2 |
| comp66751c0 | 388 | 2 |
| comp66752c0 | 311 | 2 |
| comp66766c0 | 862 | 2 |
| comp66769c0 | 718 | 2 |
| comp66781c0 | 409 | 2 |
| comp66782c0 | 404 | 2 |
| comp66789c0 | 43 | 2 |
| comp66797c0 | 652 | 2 |
| comp66807c0 | 171 | 2 |
| comp66816c0 | 679 | 2 |
| comp66822c2 | 831 | 2 |
| comp66827c0 | 176 | 2 |
| comp66828c0 | 1312 | 2 |
| comp66835c0 | 404 | 2 |
| comp66838c0 | 139 | 2 |
| comp66842c0 | 861 | 2 |
| comp66853c0 | 1192 | 2 |
| comp66855c0 | 393 | 2 |
| comp66858c0 | 331 | 2 |
| comp66861c0 | 1374 | 2 |
| comp66872c1 | 1109 | 2 |
| comp66874c0 | 908 | 2 |
| comp66875c0 | 308 | 2 |
| comp66876c0 | 924 | 2 |
| comp66877c0 | 346 | 2 |
| comp66879c0 | 954 | 2 |
| comp66883c0 | 1194 | 2 |
| comp66898c0 | 1135 | 2 |
| comp66899c0 | 1412 | 2 |
| comp66901c0 | 437 | 2 |
| comp66910c0 | 66 | 2 |
| comp66912c0 | 1096 | 2 |
| comp66919c0 | 1257 | 2 |
| comp66925c0 | 319 | 2 |
| comp66939c0 | 875 | 2 |
| comp66941c0 | 1356 | 2 |
| comp66946c0 | 610 | 2 |
| comp66955c0 | 1265 | 2 |
| comp66960c0 | 673 | 2 |
| comp66963c0 | 1005 | 2 |
| comp66965c0 | 576 | 2 |
| comp66970c0 | 534 | 2 |
| comp66981c0 | 1214 | 2 |
| comp66983c0 | 1233 | 2 |
| comp66983c1 | 1085 | 2 |
| comp66987c0 | 864 | 2 |
| comp66988c0 | 672 | 2 |
| comp66991c0 | 423 | 2 |
| comp66993c1 | 237 | 2 |
| comp66994c0 | 336 | 2 |
| comp66996c0 | 4744 | 2 |
| comp67004c0 | 599 | 2 |
| comp67006c0 | 212 | 2 |
| comp67008c0 | 814 | 2 |
| comp67016c0 | 206 | 2 |
| comp67018c0 | 95 | 2 |
| comp67020c0 | 509 | 2 |
| comp67024c0 | 1375 | 2 |
| comp67028c0 | 1389 | 2 |
| comp67030c0 | 1030 | 2 |
| comp67039c0 | 304 | 2 |
| comp67041c0 | 579 | 2 |
| comp67045c0 | 233 | 2 |
| comp67046c0 | 30 | 2 |
| comp67058c0 | 453 | 2 |
| comp67067c0 | 1978 | 2 |
| comp67068c0 | 436 | 2 |
| comp67069c0 | 255 | 2 |
| comp67074c0 | 1254 | 2 |
| comp67077c0 | 1178 | 2 |
| comp67078c0 | 371 | 2 |
| comp67083c0 | 124 | 2 |
| comp67086c0 | 1019 | 2 |
| comp67089c0 | 1238 | 2 |
| comp67092c0 | 1248 | 2 |
| comp67094c0 | 864 | 2 |
| comp67100c0 | 44 | 2 |
| comp67104c6 | 505 | 2 |
| comp67106c0 | 1657 | 2 |
| comp67111c0 | 966 | 2 |
| comp67117c0 | 194 | 2 |
| comp67118c0 | 1189 | 2 |
| comp67120c0 | 1188 | 2 |
| comp67126c0 | 311 | 2 |
| comp67132c0 | 589 | 2 |
| comp67133c0 | 857 | 2 |
| comp67138c0 | 1106 | 2 |
| comp67139c0 | 670 | 2 |
| comp67144c0 | 2548 | 2 |
| comp67148c0 | 355 | 2 |
| comp67153c0 | 314 | 2 |
| comp67156c1 | 1278 | 2 |
| comp67158c0 | 1595 | 2 |
| comp67162c0 | 899 | 2 |
| comp67167c0 | 1161 | 2 |
| comp67170c0 | 622 | 2 |
| comp67171c0 | 950 | 2 |
| comp67186c0 | 1043 | 2 |
| comp67190c0 | 1486 | 2 |
| comp67191c0 | 1301 | 2 |
| comp67193c0 | 1027 | 2 |
| comp67193c1 | 1265 | 2 |
| comp67197c0 | 90 | 2 |
| comp67204c0 | 199 | 2 |
| comp67205c0 | 2324 | 2 |
| comp67209c0 | 977 | 2 |
| comp67211c0 | 559 | 2 |
| comp67221c0 | 947 | 2 |
| comp67229c0 | 904 | 2 |
| comp67232c0 | 1419 | 2 |
| comp67236c0 | 611 | 2 |
| comp67238c0 | 423 | 2 |
| comp67241c0 | 1426 | 2 |
| comp67245c0 | 185 | 2 |
| comp67252c0 | 711 | 2 |
| comp67253c0 | 1620 | 2 |
| comp67255c0 | 1144 | 2 |
| comp67259c0 | 427 | 2 |
| comp67263c0 | 421 | 2 |
| comp67266c0 | 1364 | 2 |
| comp67273c0 | 633 | 2 |
| comp67276c0 | 398 | 2 |
| comp67279c0 | 243 | 2 |
| comp67283c0 | 1314 | 2 |
| comp67288c0 | 1358 | 2 |
| comp67292c0 | 942 | 2 |
| comp67298c0 | 1254 | 2 |
| comp67303c0 | 397 | 2 |
| comp67305c0 | 1699 | 2 |
| comp67310c0 | 367 | 2 |
| comp67315c0 | 172 | 2 |
| comp67317c0 | 1615 | 2 |
| comp67318c0 | 508 | 2 |
| comp67325c0 | 907 | 2 |
| comp67328c0 | 190 | 2 |
| comp67341c0 | 980 | 2 |
| comp67344c0 | 1408 | 2 |
| comp67345c0 | 511 | 2 |
| comp67349c0 | 522 | 2 |
| comp67356c0 | 1213 | 2 |
| comp67366c0 | 2269 | 2 |
| comp67368c0 | 199 | 2 |
| comp67371c0 | 580 | 2 |
| comp67374c0 | 777 | 2 |
| comp67378c0 | 650 | 2 |
| comp67381c0 | 407 | 2 |
| comp67383c0 | 252 | 2 |
| comp67393c0 | 909 | 2 |
| comp67401c0 | 432 | 2 |
| comp67407c0 | 400 | 2 |
| comp67411c0 | 777 | 2 |
| comp67412c1 | 1056 | 2 |
| comp67414c0 | 1213 | 2 |
| comp67420c0 | 1436 | 2 |
| comp67421c0 | 940 | 2 |
| comp67428c2 | 372 | 2 |
| comp67429c0 | 1253 | 2 |
| comp67430c0 | 638 | 2 |
| comp67435c0 | 325 | 2 |
| comp67436c0 | 1139 | 2 |
| comp67456c0 | 1572 | 2 |
| comp67465c1 | 740 | 2 |
| comp67467c0 | 1706 | 2 |
| comp67468c0 | 790 | 2 |
| comp67471c0 | 673 | 2 |
| comp67472c0 | 106 | 2 |
| comp67475c6 | 783 | 2 |
| comp67487c0 | 1331 | 2 |
| comp67490c0 | 711 | 2 |
| comp67492c0 | 404 | 2 |
| comp67495c0 | 110 | 2 |
| comp67498c0 | 490 | 2 |
| comp67501c0 | 146 | 2 |
| comp67503c0 | 723 | 2 |
| comp67506c0 | 69 | 2 |
| comp67509c0 | 1128 | 2 |
| comp67509c2 | 766 | 2 |
| comp67512c0 | 647 | 2 |
| comp67516c0 | 524 | 2 |
| comp67517c0 | 1394 | 2 |
| comp67519c0 | 644 | 2 |
| comp67521c0 | 105 | 2 |
| comp67526c0 | 183 | 2 |
| comp67528c0 | 1201 | 2 |
| comp67529c0 | 937 | 2 |
| comp67530c0 | 962 | 2 |
| comp67532c0 | 999 | 2 |
| comp67533c0 | 231 | 2 |
| comp67538c0 | 683 | 2 |
| comp67540c1 | 512 | 2 |
| comp67549c0 | 1393 | 2 |
| comp67552c0 | 730 | 2 |
| comp67554c0 | 463 | 2 |
| comp67569c0 | 938 | 2 |
| comp67571c0 | 306 | 2 |
| comp67575c0 | 128 | 2 |
| comp67576c0 | 1112 | 2 |
| comp67582c0 | 684 | 2 |
| comp67585c0 | 450 | 2 |
| comp67586c0 | 520 | 2 |
| comp67590c0 | 326 | 2 |
| comp67600c0 | 2319 | 2 |
| comp67604c0 | 448 | 2 |
| comp67607c0 | 1030 | 2 |
| comp67609c0 | 685 | 2 |
| comp67612c0 | 394 | 2 |
| comp67613c1 | 1442 | 2 |
| comp67628c0 | 979 | 2 |
| comp67636c0 | 2413 | 2 |
| comp67636c1 | 538 | 2 |
| comp67637c0 | 1010 | 2 |
| comp67642c0 | 380 | 2 |
| comp67647c0 | 658 | 2 |
| comp67661c0 | 238 | 2 |
| comp67662c0 | 494 | 2 |
| comp67667c0 | 872 | 2 |
| comp67668c0 | 843 | 2 |
| comp67670c0 | 136 | 2 |
| comp67672c0 | 455 | 2 |
| comp67673c0 | 933 | 2 |
| comp67674c0 | 1030 | 2 |
| comp67685c0 | 1159 | 2 |
| comp67686c0 | 458 | 2 |
| comp67688c0 | 1641 | 2 |
| comp67698c0 | 201 | 2 |
| comp67699c0 | 1994 | 2 |
| comp67699c1 | 311 | 2 |
| comp67704c0 | 194 | 2 |
| comp67707c0 | 115 | 2 |
| comp67708c0 | 525 | 2 |
| comp67708c1 | 1272 | 2 |
| comp67710c0 | 357 | 2 |
| comp67711c0 | 1771 | 2 |
| comp67713c0 | 639 | 2 |
| comp67715c0 | 370 | 2 |
| comp67723c0 | 842 | 2 |
| comp67724c0 | 494 | 2 |
| comp67725c0 | 1173 | 2 |
| comp67731c0 | 1503 | 2 |
| comp67741c0 | 3084 | 2 |
| comp67751c0 | 112 | 2 |
| comp67757c0 | 1880 | 2 |
| comp67761c0 | 2453 | 2 |
| comp67762c0 | 993 | 2 |
| comp67766c0 | 3081 | 2 |
| comp6776c0 | 70 | 2 |
| comp67770c0 | 142 | 2 |
| comp67773c0 | 232 | 2 |
| comp67781c0 | 624 | 2 |
| comp67782c0 | 195 | 2 |
| comp67793c0 | 1675 | 2 |
| comp67794c0 | 2644 | 2 |
| comp67799c0 | 1654 | 2 |
| comp67804c0 | 598 | 2 |
| comp67818c0 | 586 | 2 |
| comp67823c0 | 1272 | 2 |
| comp67824c0 | 502 | 2 |
| comp67829c0 | 1762 | 2 |
| comp67835c0 | 275 | 2 |
| comp67837c0 | 703 | 2 |
| comp67845c0 | 456 | 2 |
| comp67847c0 | 1080 | 2 |
| comp67847c1 | 362 | 2 |
| comp67848c0 | 1434 | 2 |
| comp67851c0 | 815 | 2 |
| comp67852c0 | 1317 | 2 |
| comp67853c0 | 1298 | 2 |
| comp67855c0 | 56 | 2 |
| comp67857c0 | 66 | 2 |
| comp67868c0 | 1254 | 2 |
| comp67870c0 | 2003 | 2 |
| comp67871c0 | 179 | 2 |
| comp67876c1 | 1182 | 2 |
| comp67879c1 | 71 | 2 |
| comp67880c0 | 1703 | 2 |
| comp67882c0 | 657 | 2 |
| comp67884c0 | 532 | 2 |
| comp67898c0 | 966 | 2 |
| comp67899c0 | 691 | 2 |
| comp67904c0 | 507 | 2 |
| comp67909c0 | 728 | 2 |
| comp67912c0 | 1487 | 2 |
| comp67912c1 | 1156 | 2 |
| comp67917c0 | 275 | 2 |
| comp67919c0 | 506 | 2 |
| comp67920c0 | 149 | 2 |
| comp67924c0 | 1223 | 2 |
| comp67927c0 | 576 | 2 |
| comp67933c0 | 579 | 2 |
| comp67935c0 | 504 | 2 |
| comp67937c0 | 636 | 2 |
| comp67939c0 | 542 | 2 |
| comp67940c0 | 826 | 2 |
| comp67947c0 | 173 | 2 |
| comp67952c0 | 783 | 2 |
| comp67955c0 | 428 | 2 |
| comp67960c0 | 867 | 2 |
| comp67960c1 | 2872 | 2 |
| comp67963c0 | 386 | 2 |
| comp67966c0 | 513 | 2 |
| comp67967c0 | 1125 | 2 |
| comp67968c0 | 655 | 2 |
| comp67970c0 | 379 | 2 |
| comp67978c0 | 1170 | 2 |
| comp67980c0 | 1134 | 2 |
| comp67984c0 | 787 | 2 |
| comp67989c0 | 815 | 2 |
| comp67993c0 | 1104 | 2 |
| comp67994c0 | 116 | 2 |
| comp67998c0 | 1574 | 2 |
| comp67999c0 | 1470 | 2 |
| comp68002c0 | 1891 | 2 |
| comp68004c0 | 125 | 2 |
| comp68005c0 | 428 | 2 |
| comp68016c0 | 380 | 2 |
| comp68018c0 | 792 | 2 |
| comp68019c0 | 1763 | 2 |
| comp68024c1 | 1200 | 2 |
| comp68030c0 | 1427 | 2 |
| comp68036c0 | 781 | 2 |
| comp68037c0 | 311 | 2 |
| comp68038c0 | 2316 | 2 |
| comp68040c0 | 1385 | 2 |
| comp68043c0 | 1190 | 2 |
| comp68050c0 | 404 | 2 |
| comp68051c0 | 1140 | 2 |
| comp68058c0 | 745 | 2 |
| comp68064c2 | 1178 | 2 |
| comp68066c0 | 958 | 2 |
| comp68067c0 | 1111 | 2 |
| comp68074c0 | 41 | 2 |
| comp680792c0 | 711 | 2 |
| comp68080c0 | 1034 | 2 |
| comp68085c0 | 595 | 2 |
| comp68096c0 | 1129 | 2 |
| comp68106c1 | 549 | 2 |
| comp68109c0 | 527 | 2 |
| comp68112c0 | 279 | 2 |
| comp68114c0 | 917 | 2 |
| comp68121c0 | 25 | 2 |
| comp68125c0 | 371 | 2 |
| comp68129c0 | 1205 | 2 |
| comp68132c0 | 1081 | 2 |
| comp68133c4 | 2975 | 2 |
| comp68137c0 | 764 | 2 |
| comp68152c0 | 78 | 2 |
| comp68153c0 | 55 | 2 |
| comp68154c0 | 602 | 2 |
| comp68158c0 | 529 | 2 |
| comp68162c0 | 444 | 2 |
| comp68170c1 | 797 | 2 |
| comp68171c0 | 188 | 2 |
| comp68172c0 | 1087 | 2 |
| comp68174c0 | 689 | 2 |
| comp68180c0 | 917 | 2 |
| comp68181c0 | 1110 | 2 |
| comp68185c0 | 754 | 2 |
| comp68189c0 | 390 | 2 |
| comp68191c0 | 284 | 2 |
| comp68193c0 | 354 | 2 |
| comp68199c0 | 883 | 2 |
| comp68200c0 | 586 | 2 |
| comp68201c0 | 1697 | 2 |
| comp68203c0 | 376 | 2 |
| comp68205c0 | 419 | 2 |
| comp68205c1 | 459 | 2 |
| comp68217c0 | 64 | 2 |
| comp68231c0 | 491 | 2 |
| comp68240c0 | 823 | 2 |
| comp68243c0 | 316 | 2 |
| comp68248c0 | 415 | 2 |
| comp68250c0 | 920 | 2 |
| comp68253c0 | 491 | 2 |
| comp68255c2 | 973 | 2 |
| comp68256c0 | 666 | 2 |
| comp68259c0 | 1619 | 2 |
| comp68260c0 | 455 | 2 |
| comp68263c0 | 1771 | 2 |
| comp68266c0 | 960 | 2 |
| comp68269c0 | 124 | 2 |
| comp68284c0 | 1468 | 2 |
| comp68293c0 | 270 | 2 |
| comp68299c0 | 625 | 2 |
| comp68306c0 | 1817 | 2 |
| comp68307c0 | 930 | 2 |
| comp68310c0 | 2122 | 2 |
| comp68313c0 | 1460 | 2 |
| comp68320c0 | 850 | 2 |
| comp68324c0 | 1400 | 2 |
| comp68325c0 | 1453 | 2 |
| comp68328c0 | 182 | 2 |
| comp68333c0 | 1524 | 2 |
| comp68335c1 | 1506 | 2 |
| comp68339c0 | 411 | 2 |
| comp68341c0 | 525 | 2 |
| comp68343c0 | 896 | 2 |
| comp68346c0 | 650 | 2 |
| comp68355c0 | 101 | 2 |
| comp68360c0 | 985 | 2 |
| comp68361c0 | 754 | 2 |
| comp68364c0 | 2542 | 2 |
| comp68372c0 | 353 | 2 |
| comp68373c0 | 571 | 2 |
| comp68376c0 | 3471 | 2 |
| comp68390c0 | 1178 | 2 |
| comp68405c0 | 1153 | 2 |
| comp68407c0 | 908 | 2 |
| comp68413c0 | 1254 | 2 |
| comp68418c0 | 91 | 2 |
| comp68421c0 | 1760 | 2 |
| comp68428c1 | 230 | 2 |
| comp68433c0 | 1035 | 2 |
| comp68434c0 | 801 | 2 |
| comp68436c0 | 625 | 2 |
| comp68437c0 | 589 | 2 |
| comp68440c0 | 672 | 2 |
| comp68441c0 | 310 | 2 |
| comp68442c0 | 682 | 2 |
| comp68443c0 | 1275 | 2 |
| comp68444c0 | 351 | 2 |
| comp68451c0 | 338 | 2 |
| comp68452c0 | 817 | 2 |
| comp68453c0 | 1218 | 2 |
| comp68456c0 | 267 | 2 |
| comp68458c0 | 923 | 2 |
| comp68467c0 | 1103 | 2 |
| comp68469c0 | 490 | 2 |
| comp68477c0 | 255 | 2 |
| comp68482c1 | 95 | 2 |
| comp68485c0 | 1081 | 2 |
| comp68487c0 | 370 | 2 |
| comp68492c0 | 1131 | 2 |
| comp68503c0 | 1013 | 2 |
| comp68505c0 | 566 | 2 |
| comp68507c0 | 254 | 2 |
| comp68510c0 | 251 | 2 |
| comp68514c0 | 435 | 2 |
| comp68517c0 | 575 | 2 |
| comp68519c0 | 553 | 2 |
| comp68529c0 | 605 | 2 |
| comp68532c0 | 617 | 2 |
| comp68539c0 | 582 | 2 |
| comp68540c0 | 284 | 2 |
| comp68545c0 | 3004 | 2 |
| comp68548c0 | 780 | 2 |
| comp68555c0 | 1237 | 2 |
| comp68561c0 | 341 | 2 |
| comp68566c0 | 514 | 2 |
| comp68572c0 | 843 | 2 |
| comp68575c0 | 1503 | 2 |
| comp68578c0 | 517 | 2 |
| comp68581c0 | 664 | 2 |
| comp68588c0 | 1317 | 2 |
| comp68591c0 | 284 | 2 |
| comp68593c0 | 155 | 2 |
| comp68595c0 | 1608 | 2 |
| comp68602c0 | 597 | 2 |
| comp68609c0 | 621 | 2 |
| comp68619c0 | 1356 | 2 |
| comp68625c0 | 612 | 2 |
| comp68626c0 | 524 | 2 |
| comp68634c0 | 1007 | 2 |
| comp68639c0 | 1100 | 2 |
| comp68642c0 | 2570 | 2 |
| comp68644c0 | 596 | 2 |
| comp68647c1 | 1064 | 2 |
| comp68648c0 | 270 | 2 |
| comp68650c0 | 284 | 2 |
| comp68655c0 | 170 | 2 |
| comp68664c1 | 278 | 2 |
| comp68670c1 | 400 | 2 |
| comp68674c0 | 530 | 2 |
| comp68690c0 | 3853 | 2 |
| comp68694c0 | 143 | 2 |
| comp68698c0 | 712 | 2 |
| comp68710c0 | 777 | 2 |
| comp68713c0 | 827 | 2 |
| comp68715c0 | 1256 | 2 |
| comp68715c1 | 275 | 2 |
| comp68719c0 | 68 | 2 |
| comp68722c0 | 562 | 2 |
| comp68723c2 | 364 | 2 |
| comp68727c0 | 1354 | 2 |
| comp68733c0 | 1222 | 2 |
| comp68734c0 | 984 | 2 |
| comp68736c0 | 406 | 2 |
| comp68742c0 | 897 | 2 |
| comp68745c0 | 1035 | 2 |
| comp68753c2 | 984 | 2 |
| comp68754c0 | 50 | 2 |
| comp68760c0 | 1035 | 2 |
| comp68766c0 | 870 | 2 |
| comp68769c0 | 1258 | 2 |
| comp68777c0 | 706 | 2 |
| comp68781c0 | 1450 | 2 |
| comp68784c0 | 166 | 2 |
| comp68787c0 | 243 | 2 |
| comp68792c0 | 543 | 2 |
| comp68795c0 | 391 | 2 |
| comp68796c0 | 371 | 2 |
| comp68805c0 | 362 | 2 |
| comp68809c0 | 256 | 2 |
| comp68811c0 | 1215 | 2 |
| comp68821c0 | 988 | 2 |
| comp68822c0 | 275 | 2 |
| comp68823c0 | 1067 | 2 |
| comp68831c0 | 988 | 2 |
| comp68834c0 | 714 | 2 |
| comp68845c0 | 2034 | 2 |
| comp68849c0 | 1361 | 2 |
| comp68854c0 | 2206 | 2 |
| comp68869c0 | 184 | 2 |
| comp68872c0 | 282 | 2 |
| comp68875c0 | 619 | 2 |
| comp68877c0 | 1075 | 2 |
| comp68888c0 | 1198 | 2 |
| comp68890c0 | 831 | 2 |
| comp68894c0 | 849 | 2 |
| comp68896c0 | 1096 | 2 |
| comp68902c0 | 832 | 2 |
| comp68909c1 | 122 | 2 |
| comp68912c0 | 293 | 2 |
| comp68917c1 | 311 | 2 |
| comp68918c0 | 115 | 2 |
| comp68921c0 | 453 | 2 |
| comp68926c0 | 1170 | 2 |
| comp68927c0 | 1333 | 2 |
| comp68929c0 | 813 | 2 |
| comp68930c0 | 620 | 2 |
| comp68936c0 | 890 | 2 |
| comp68947c0 | 180 | 2 |
| comp68954c0 | 1321 | 2 |
| comp68956c0 | 734 | 2 |
| comp68957c0 | 180 | 2 |
| comp68958c0 | 592 | 2 |
| comp68959c0 | 409 | 2 |
| comp68960c0 | 1385 | 2 |
| comp68963c0 | 1868 | 2 |
| comp68969c0 | 916 | 2 |
| comp68971c0 | 553 | 2 |
| comp68973c0 | 280 | 2 |
| comp68977c0 | 166 | 2 |
| comp68985c0 | 649 | 2 |
| comp68988c0 | 1097 | 2 |
| comp68991c0 | 1083 | 2 |
| comp68992c0 | 814 | 2 |
| comp68995c0 | 481 | 2 |
| comp68996c0 | 214 | 2 |
| comp68999c0 | 777 | 2 |
| comp69001c0 | 1171 | 2 |
| comp69002c0 | 564 | 2 |
| comp69005c0 | 692 | 2 |
| comp69009c0 | 389 | 2 |
| comp69016c0 | 1000 | 2 |
| comp69018c0 | 773 | 2 |
| comp69019c0 | 81 | 2 |
| comp69024c2 | 292 | 2 |
| comp69026c0 | 471 | 2 |
| comp69030c0 | 370 | 2 |
| comp69033c0 | 405 | 2 |
| comp69049c0 | 1234 | 2 |
| comp69052c0 | 414 | 2 |
| comp69053c0 | 741 | 2 |
| comp69056c0 | 672 | 2 |
| comp69059c0 | 1443 | 2 |
| comp69061c0 | 1383 | 2 |
| comp69064c0 | 1302 | 2 |
| comp69065c0 | 584 | 2 |
| comp69072c0 | 972 | 2 |
| comp69073c0 | 1280 | 2 |
| comp69081c0 | 531 | 2 |
| comp69087c0 | 1150 | 2 |
| comp69092c0 | 1790 | 2 |
| comp69093c0 | 811 | 2 |
| comp69101c0 | 378 | 2 |
| comp69104c0 | 1130 | 2 |
| comp69105c0 | 1053 | 2 |
| comp69109c0 | 1399 | 2 |
| comp69119c0 | 457 | 2 |
| comp69121c0 | 239 | 2 |
| comp69130c0 | 1249 | 2 |
| comp69133c0 | 504 | 2 |
| comp69134c0 | 950 | 2 |
| comp69135c0 | 471 | 2 |
| comp69138c0 | 123 | 2 |
| comp69143c0 | 37 | 2 |
| comp69147c0 | 641 | 2 |
| comp69154c0 | 286 | 2 |
| comp69159c0 | 891 | 2 |
| comp69161c0 | 611 | 2 |
| comp69162c1 | 1492 | 2 |
| comp69184c0 | 617 | 2 |
| comp69186c0 | 448 | 2 |
| comp69188c0 | 541 | 2 |
| comp69200c0 | 647 | 2 |
| comp69201c0 | 487 | 2 |
| comp69204c0 | 159372 | 2 |
| comp69206c0 | 1245 | 2 |
| comp69214c0 | 136 | 2 |
| comp69217c0 | 355 | 2 |
| comp69221c0 | 219 | 2 |
| comp69224c1 | 666 | 2 |
| comp69226c0 | 677 | 2 |
| comp69229c0 | 2124 | 2 |
| comp69231c0 | 688 | 2 |
| comp69236c0 | 528 | 2 |
| comp69239c7 | 1856 | 2 |
| comp69240c0 | 441 | 2 |
| comp69245c0 | 506 | 2 |
| comp69246c2 | 1587 | 2 |
| comp69249c0 | 58 | 2 |
| comp69251c0 | 652 | 2 |
| comp69260c0 | 456 | 2 |
| comp69264c0 | 2181 | 2 |
| comp69265c0 | 1015 | 2 |
| comp69268c0 | 535 | 2 |
| comp69272c0 | 1114 | 2 |
| comp69276c0 | 124 | 2 |
| comp69280c0 | 1204 | 2 |
| comp69281c0 | 915 | 2 |
| comp69287c0 | 704 | 2 |
| comp69290c0 | 317 | 2 |
| comp69295c0 | 629 | 2 |
| comp69300c0 | 1527 | 2 |
| comp69302c0 | 1686 | 2 |
| comp69313c0 | 891 | 2 |
| comp69327c0 | 1046 | 2 |
| comp69328c1 | 372 | 2 |
| comp69330c0 | 809 | 2 |
| comp69333c0 | 663 | 2 |
| comp69345c0 | 218 | 2 |
| comp69348c0 | 835 | 2 |
| comp69349c0 | 754 | 2 |
| comp69351c2 | 1449 | 2 |
| comp69354c0 | 218 | 2 |
| comp69358c0 | 321 | 2 |
| comp69361c0 | 133 | 2 |
| comp69363c0 | 199 | 2 |
| comp69364c0 | 472 | 2 |
| comp69369c0 | 201 | 2 |
| comp69380c0 | 623 | 2 |
| comp69383c0 | 518 | 2 |
| comp69384c0 | 937 | 2 |
| comp69385c0 | 234 | 2 |
| comp69386c0 | 226 | 2 |
| comp69389c0 | 554 | 2 |
| comp69403c0 | 600 | 2 |
| comp69410c0 | 1189 | 2 |
| comp69411c0 | 723 | 2 |
| comp69418c0 | 3257 | 2 |
| comp69421c0 | 635 | 2 |
| comp69431c0 | 470 | 2 |
| comp69440c0 | 932 | 2 |
| comp69449c0 | 1132 | 2 |
| comp69453c0 | 152 | 2 |
| comp69459c0 | 1731 | 2 |
| comp69461c0 | 401 | 2 |
| comp69463c0 | 419 | 2 |
| comp69468c1 | 387 | 2 |
| comp69479c0 | 965 | 2 |
| comp69481c0 | 699 | 2 |
| comp69486c1 | 1281 | 2 |
| comp69486c6 | 952 | 2 |
| comp69488c0 | 971 | 2 |
| comp69489c0 | 287 | 2 |
| comp69491c0 | 468 | 2 |
| comp69492c0 | 1127 | 2 |
| comp69497c0 | 1743 | 2 |
| comp69498c0 | 924 | 2 |
| comp69500c0 | 339 | 2 |
| comp69504c0 | 1307 | 2 |
| comp69505c0 | 621 | 2 |
| comp69511c0 | 917 | 2 |
| comp69517c0 | 170 | 2 |
| comp69517c1 | 1139 | 2 |
| comp69521c0 | 369 | 2 |
| comp69528c0 | 2498 | 2 |
| comp69531c0 | 318 | 2 |
| comp69532c0 | 1070 | 2 |
| comp69533c0 | 500 | 2 |
| comp69540c0 | 511 | 2 |
| comp69546c1 | 326 | 2 |
| comp69553c0 | 1475 | 2 |
| comp69566c0 | 453 | 2 |
| comp69569c0 | 212 | 2 |
| comp69573c0 | 1439 | 2 |
| comp69582c0 | 565 | 2 |
| comp69585c0 | 581 | 2 |
| comp69589c1 | 246 | 2 |
| comp69596c0 | 921 | 2 |
| comp69606c0 | 938 | 2 |
| comp69612c0 | 604 | 2 |
| comp69614c0 | 437 | 2 |
| comp69617c0 | 961 | 2 |
| comp69621c0 | 54 | 2 |
| comp69623c0 | 2146 | 2 |
| comp69625c0 | 833 | 2 |
| comp69626c0 | 1159 | 2 |
| comp69630c0 | 168 | 2 |
| comp69651c0 | 807 | 2 |
| comp69653c0 | 340 | 2 |
| comp69657c0 | 73 | 2 |
| comp69659c0 | 823 | 2 |
| comp69661c0 | 896 | 2 |
| comp69662c0 | 254 | 2 |
| comp69664c0 | 479 | 2 |
| comp69667c0 | 2468 | 2 |
| comp69668c0 | 610 | 2 |
| comp69677c0 | 899 | 2 |
| comp69682c0 | 731 | 2 |
| comp69683c0 | 255 | 2 |
| comp69684c0 | 496 | 2 |
| comp69685c1 | 809 | 2 |
| comp69687c0 | 1000 | 2 |
| comp69689c0 | 112 | 2 |
| comp69691c0 | 422 | 2 |
| comp69692c0 | 1967 | 2 |
| comp69694c1 | 689 | 2 |
| comp69696c0 | 685 | 2 |
| comp69700c0 | 445 | 2 |
| comp69707c0 | 33 | 2 |
| comp69719c0 | 954 | 2 |
| comp69721c0 | 147 | 2 |
| comp69724c0 | 1123 | 2 |
| comp69743c0 | 208 | 2 |
| comp69745c0 | 2383 | 2 |
| comp69752c0 | 883 | 2 |
| comp69759c0 | 475 | 2 |
| comp69761c0 | 108 | 2 |
| comp69769c0 | 321 | 2 |
| comp69770c0 | 418 | 2 |
| comp69773c0 | 487 | 2 |
| comp69774c0 | 68 | 2 |
| comp69782c0 | 642 | 2 |
| comp69787c0 | 81 | 2 |
| comp69788c0 | 1101 | 2 |
| comp69793c2 | 715 | 2 |
| comp69804c0 | 371 | 2 |
| comp69806c0 | 1275 | 2 |
| comp69807c0 | 554 | 2 |
| comp69808c0 | 361 | 2 |
| comp69822c0 | 1199 | 2 |
| comp69832c0 | 492 | 2 |
| comp69835c0 | 335 | 2 |
| comp69836c0 | 1195 | 2 |
| comp69839c0 | 492 | 2 |
| comp69841c0 | 222 | 2 |
| comp69842c0 | 34 | 2 |
| comp69850c0 | 1005 | 2 |
| comp69852c0 | 365 | 2 |
| comp69853c0 | 148 | 2 |
| comp69862c0 | 652 | 2 |
| comp69863c0 | 1140 | 2 |
| comp69865c0 | 1194 | 2 |
| comp69868c0 | 1429 | 2 |
| comp69884c0 | 600 | 2 |
| comp69904c0 | 357 | 2 |
| comp69905c1 | 1055 | 2 |
| comp69911c0 | 1004 | 2 |
| comp69913c0 | 287 | 2 |
| comp69919c0 | 854 | 2 |
| comp69930c0 | 664 | 2 |
| comp69932c0 | 569 | 2 |
| comp69939c0 | 122 | 2 |
| comp69941c0 | 973 | 2 |
| comp69943c0 | 1095 | 2 |
| comp69947c0 | 270 | 2 |
| comp69949c0 | 847 | 2 |
| comp69951c0 | 1469 | 2 |
| comp69969c0 | 762 | 2 |
| comp69975c0 | 254 | 2 |
| comp69978c0 | 1860 | 2 |
| comp69983c0 | 3501 | 2 |
| comp69999c0 | 948 | 2 |
| comp70000c0 | 1011 | 2 |
| comp70004c0 | 591 | 2 |
| comp70005c0 | 329 | 2 |
| comp70016c0 | 729 | 2 |
| comp70022c0 | 1095 | 2 |
| comp70025c0 | 561 | 2 |
| comp70026c0 | 458 | 2 |
| comp70031c0 | 409 | 2 |
| comp70032c0 | 619 | 2 |
| comp70034c0 | 701 | 2 |
| comp70045c0 | 1125 | 2 |
| comp70052c0 | 208 | 2 |
| comp70053c0 | 802 | 2 |
| comp70061c0 | 575 | 2 |
| comp70073c0 | 829 | 2 |
| comp70082c0 | 1132 | 2 |
| comp70083c0 | 928 | 2 |
| comp70086c0 | 1440 | 2 |
| comp70088c0 | 976 | 2 |
| comp70109c0 | 652 | 2 |
| comp70111c0 | 662 | 2 |
| comp70112c0 | 1392 | 2 |
| comp70113c0 | 931 | 2 |
| comp70116c0 | 455 | 2 |
| comp70117c0 | 491 | 2 |
| comp70118c0 | 1109 | 2 |
| comp70129c0 | 716 | 2 |
| comp70132c0 | 1112 | 2 |
| comp70136c0 | 215 | 2 |
| comp70137c0 | 872 | 2 |
| comp70141c0 | 67 | 2 |
| comp70147c0 | 1345 | 2 |
| comp70149c0 | 267 | 2 |
| comp70150c1 | 1853 | 2 |
| comp70150c2 | 1028 | 2 |
| comp70154c0 | 382 | 2 |
| comp70157c0 | 635 | 2 |
| comp70164c0 | 15 | 2 |
| comp70173c0 | 571 | 2 |
| comp70175c0 | 2070 | 2 |
| comp70177c0 | 997 | 2 |
| comp70181c0 | 158 | 2 |
| comp70182c0 | 1135 | 2 |
| comp70185c0 | 180 | 2 |
| comp70186c0 | 683 | 2 |
| comp70188c0 | 796 | 2 |
| comp70190c0 | 308 | 2 |
| comp70191c0 | 399 | 2 |
| comp70195c0 | 37 | 2 |
| comp70196c0 | 462 | 2 |
| comp70199c0 | 416 | 2 |
| comp70204c0 | 486 | 2 |
| comp70211c0 | 953 | 2 |
| comp70214c0 | 936 | 2 |
| comp70215c0 | 1093 | 2 |
| comp70221c0 | 325 | 2 |
| comp70223c0 | 1223 | 2 |
| comp70227c0 | 530 | 2 |
| comp70228c0 | 421 | 2 |
| comp70229c0 | 754 | 2 |
| comp70234c1 | 1192 | 2 |
| comp70246c0 | 312 | 2 |
| comp70247c0 | 1454 | 2 |
| comp70258c0 | 503 | 2 |
| comp70263c0 | 394 | 2 |
| comp70265c0 | 873 | 2 |
| comp70272c0 | 819 | 2 |
| comp70273c0 | 232 | 2 |
| comp70274c0 | 738 | 2 |
| comp70278c0 | 750 | 2 |
| comp70281c0 | 71 | 2 |
| comp70293c0 | 183 | 2 |
| comp70300c0 | 1272 | 2 |
| comp70301c0 | 1442 | 2 |
| comp70302c0 | 962 | 2 |
| comp70303c0 | 1051 | 2 |
| comp70307c0 | 603 | 2 |
| comp70310c0 | 370 | 2 |
| comp70315c0 | 1420 | 2 |
| comp70325c1 | 185 | 2 |
| comp70331c0 | 637 | 2 |
| comp70335c0 | 697 | 2 |
| comp70336c0 | 206 | 2 |
| comp70339c0 | 1027 | 2 |
| comp70343c1 | 569 | 2 |
| comp70345c0 | 311 | 2 |
| comp70349c0 | 159 | 2 |
| comp70354c0 | 382 | 2 |
| comp70357c0 | 526 | 2 |
| comp70360c0 | 695 | 2 |
| comp70366c0 | 1266 | 2 |
| comp70368c0 | 976 | 2 |
| comp70371c0 | 605 | 2 |
| comp70372c0 | 686 | 2 |
| comp70381c0 | 954 | 2 |
| comp70388c0 | 494 | 2 |
| comp70392c0 | 1232 | 2 |
| comp70399c0 | 764 | 2 |
| comp70402c0 | 1462 | 2 |
| comp70404c0 | 402 | 2 |
| comp70405c0 | 49 | 2 |
| comp70411c0 | 1567 | 2 |
| comp70413c0 | 937 | 2 |
| comp70421c0 | 528 | 2 |
| comp70422c0 | 499 | 2 |
| comp70430c0 | 783 | 2 |
| comp70434c0 | 370 | 2 |
| comp70440c0 | 323 | 2 |
| comp70442c0 | 4010 | 2 |
| comp70444c0 | 845 | 2 |
| comp70450c1 | 557 | 2 |
| comp70462c0 | 48 | 2 |
| comp70467c0 | 1996 | 2 |
| comp70468c0 | 165 | 2 |
| comp70471c0 | 526 | 2 |
| comp70478c0 | 612 | 2 |
| comp70481c0 | 266 | 2 |
| comp70490c0 | 169 | 2 |
| comp70492c0 | 669 | 2 |
| comp70495c1 | 36 | 2 |
| comp70503c0 | 889 | 2 |
| comp70514c0 | 164 | 2 |
| comp70517c0 | 541 | 2 |
| comp70522c0 | 1474 | 2 |
| comp70523c1 | 286 | 2 |
| comp70525c0 | 1959 | 2 |
| comp70538c0 | 305 | 2 |
| comp70541c0 | 320 | 2 |
| comp70545c0 | 763 | 2 |
| comp70553c0 | 1214 | 2 |
| comp70556c0 | 1531 | 2 |
| comp70560c0 | 433 | 2 |
| comp70561c0 | 175 | 2 |
| comp70565c0 | 1212 | 2 |
| comp70573c0 | 1323 | 2 |
| comp70576c0 | 3907 | 2 |
| comp70578c0 | 1347 | 2 |
| comp70580c0 | 496 | 2 |
| comp70582c0 | 781 | 2 |
| comp70583c0 | 442 | 2 |
| comp70586c1 | 722 | 2 |
| comp70592c0 | 524 | 2 |
| comp70593c0 | 106 | 2 |
| comp70594c0 | 196 | 2 |
| comp70594c1 | 1524 | 2 |
| comp70597c0 | 636 | 2 |
| comp70600c0 | 331 | 2 |
| comp70601c0 | 690 | 2 |
| comp70607c0 | 1176 | 2 |
| comp70608c0 | 864 | 2 |
| comp70611c0 | 562 | 2 |
| comp70619c0 | 475 | 2 |
| comp70624c0 | 1171 | 2 |
| comp70627c0 | 197 | 2 |
| comp70637c0 | 123 | 2 |
| comp70641c0 | 704 | 2 |
| comp70643c0 | 295 | 2 |
| comp706455c0 | 18 | 2 |
| comp70645c0 | 173 | 2 |
| comp70646c0 | 2537 | 2 |
| comp70652c1 | 1575 | 2 |
| comp70653c0 | 1149 | 2 |
| comp70657c0 | 605 | 2 |
| comp70658c0 | 1163 | 2 |
| comp70659c0 | 592 | 2 |
| comp70659c1 | 298 | 2 |
| comp70660c0 | 189 | 2 |
| comp70660c1 | 1214 | 2 |
| comp70661c0 | 639 | 2 |
| comp70670c0 | 576 | 2 |
| comp70680c1 | 820 | 2 |
| comp70685c0 | 404 | 2 |
| comp70703c0 | 954 | 2 |
| comp70707c0 | 741 | 2 |
| comp70710c0 | 919 | 2 |
| comp70716c0 | 346 | 2 |
| comp70717c0 | 561 | 2 |
| comp70722c0 | 248 | 2 |
| comp70729c0 | 974 | 2 |
| comp70730c0 | 742 | 2 |
| comp70731c0 | 651 | 2 |
| comp70740c0 | 775 | 2 |
| comp70742c0 | 149434 | 2 |
| comp70743c0 | 2138 | 2 |
| comp70745c0 | 224 | 2 |
| comp70751c0 | 763 | 2 |
| comp70753c2 | 667 | 2 |
| comp70756c0 | 1290 | 2 |
| comp70760c0 | 384 | 2 |
| comp70762c0 | 202 | 2 |
| comp70769c0 | 173 | 2 |
| comp70775c0 | 327 | 2 |
| comp70777c0 | 628 | 2 |
| comp70800c0 | 573 | 2 |
| comp70806c0 | 139 | 2 |
| comp70810c0 | 229 | 2 |
| comp70811c0 | 661 | 2 |
| comp70820c0 | 676 | 2 |
| comp70822c0 | 1102 | 2 |
| comp70827c0 | 256 | 2 |
| comp70831c0 | 752 | 2 |
| comp70835c0 | 976 | 2 |
| comp70836c0 | 151 | 2 |
| comp70854c0 | 2526 | 2 |
| comp70856c0 | 215 | 2 |
| comp70862c0 | 305 | 2 |
| comp70863c0 | 387 | 2 |
| comp70869c0 | 791 | 2 |
| comp70878c0 | 740 | 2 |
| comp70879c0 | 644 | 2 |
| comp70884c0 | 925 | 2 |
| comp70886c0 | 1029 | 2 |
| comp70891c0 | 606 | 2 |
| comp70906c2 | 785 | 2 |
| comp70918c0 | 1725 | 2 |
| comp70924c0 | 653 | 2 |
| comp70925c2 | 199 | 2 |
| comp70931c0 | 795 | 2 |
| comp70932c0 | 52 | 2 |
| comp70936c1 | 14 | 2 |
| comp70937c0 | 1247 | 2 |
| comp70938c0 | 951 | 2 |
| comp70943c0 | 217 | 2 |
| comp70944c0 | 1200 | 2 |
| comp70947c0 | 872 | 2 |
| comp70957c0 | 1469 | 2 |
| comp70965c0 | 868 | 2 |
| comp70973c0 | 684 | 2 |
| comp70981c0 | 508 | 2 |
| comp70982c0 | 1275 | 2 |
| comp70991c0 | 50 | 2 |
| comp70992c0 | 1770 | 2 |
| comp70993c0 | 969 | 2 |
| comp70995c2 | 320 | 2 |
| comp70996c0 | 833 | 2 |
| comp70998c0 | 840 | 2 |
| comp71003c0 | 1947 | 2 |
| comp71008c0 | 1551 | 2 |
| comp71011c0 | 1374 | 2 |
| comp71014c0 | 2402 | 2 |
| comp71028c0 | 1927 | 2 |
| comp71031c0 | 440 | 2 |
| comp71036c0 | 606 | 2 |
| comp71041c0 | 765 | 2 |
| comp71043c1 | 754 | 2 |
| comp71049c0 | 300 | 2 |
| comp71051c0 | 691 | 2 |
| comp71059c0 | 1005 | 2 |
| comp71075c0 | 1145 | 2 |
| comp71084c0 | 98 | 2 |
| comp71088c1 | 812 | 2 |
| comp71100c0 | 562 | 2 |
| comp71103c0 | 757 | 2 |
| comp71104c0 | 1062 | 2 |
| comp71105c1 | 431 | 2 |
| comp71106c0 | 642 | 2 |
| comp71107c0 | 930 | 2 |
| comp71112c0 | 890 | 2 |
| comp71114c0 | 1212 | 2 |
| comp71118c0 | 1058 | 2 |
| comp71122c0 | 656 | 2 |
| comp71126c0 | 501 | 2 |
| comp71130c0 | 697 | 2 |
| comp71140c1 | 816 | 2 |
| comp71143c0 | 369 | 2 |
| comp71145c0 | 1037 | 2 |
| comp71151c0 | 456 | 2 |
| comp71156c0 | 231 | 2 |
| comp71163c0 | 115 | 2 |
| comp71164c0 | 509 | 2 |
| comp71165c0 | 474 | 2 |
| comp71176c0 | 150 | 2 |
| comp71178c0 | 1565 | 2 |
| comp71179c0 | 299 | 2 |
| comp71185c0 | 1035 | 2 |
| comp71187c0 | 1593 | 2 |
| comp71193c0 | 1908 | 2 |
| comp71194c0 | 558 | 2 |
| comp71198c0 | 328 | 2 |
| comp71201c0 | 1378 | 2 |
| comp71207c0 | 1311 | 2 |
| comp71207c1 | 812 | 2 |
| comp71214c0 | 1195 | 2 |
| comp71215c0 | 124 | 2 |
| comp71217c0 | 459 | 2 |
| comp71223c0 | 874 | 2 |
| comp71230c0 | 393 | 2 |
| comp71231c0 | 935 | 2 |
| comp71232c0 | 1016 | 2 |
| comp71235c1 | 883 | 2 |
| comp71242c0 | 82 | 2 |
| comp71244c0 | 906 | 2 |
| comp71272c0 | 337 | 2 |
| comp71277c0 | 856 | 2 |
| comp71278c0 | 1648 | 2 |
| comp71290c1 | 471 | 2 |
| comp71292c0 | 309 | 2 |
| comp71297c0 | 779 | 2 |
| comp71305c1 | 323 | 2 |
| comp71315c0 | 1627 | 2 |
| comp71316c0 | 442 | 2 |
| comp71320c1 | 1366 | 2 |
| comp71325c1 | 347 | 2 |
| comp71330c0 | 15 | 2 |
| comp71332c0 | 843 | 2 |
| comp71333c0 | 1570 | 2 |
| comp71334c0 | 1449 | 2 |
| comp71344c0 | 688 | 2 |
| comp713509c0 | 606 | 2 |
| comp71351c0 | 560 | 2 |
| comp71367c0 | 11 | 2 |
| comp71379c0 | 699 | 2 |
| comp71381c0 | 902 | 2 |
| comp71383c0 | 1236 | 2 |
| comp71386c0 | 760 | 2 |
| comp71391c0 | 464 | 2 |
| comp71392c0 | 496 | 2 |
| comp71395c0 | 84 | 2 |
| comp71397c0 | 381 | 2 |
| comp71398c1 | 1052 | 2 |
| comp71409c0 | 634 | 2 |
| comp71411c0 | 908 | 2 |
| comp71419c0 | 971 | 2 |
| comp71436c0 | 200 | 2 |
| comp71437c0 | 123 | 2 |
| comp71460c0 | 1203 | 2 |
| comp71461c3 | 507 | 2 |
| comp71465c0 | 444 | 2 |
| comp71468c0 | 589 | 2 |
| comp71469c0 | 1383 | 2 |
| comp71475c0 | 260 | 2 |
| comp71485c0 | 505 | 2 |
| comp71492c0 | 259 | 2 |
| comp71495c0 | 1064 | 2 |
| comp71499c0 | 196 | 2 |
| comp71502c0 | 1248 | 2 |
| comp71511c0 | 1208 | 2 |
| comp71511c1 | 783 | 2 |
| comp71512c2 | 66 | 2 |
| comp71515c0 | 634 | 2 |
| comp71517c0 | 1397 | 2 |
| comp71521c0 | 661 | 2 |
| comp71529c0 | 209 | 2 |
| comp71532c0 | 1627 | 2 |
| comp71536c0 | 50 | 2 |
| comp71537c0 | 2188 | 2 |
| comp71554c0 | 1312 | 2 |
| comp71558c0 | 949 | 2 |
| comp71560c0 | 1639 | 2 |
| comp71567c0 | 848 | 2 |
| comp71572c0 | 1181 | 2 |
| comp71574c0 | 916 | 2 |
| comp71581c0 | 702 | 2 |
| comp71587c0 | 1237 | 2 |
| comp71589c0 | 536 | 2 |
| comp71594c0 | 629 | 2 |
| comp71607c0 | 590 | 2 |
| comp71609c0 | 703 | 2 |
| comp71610c0 | 1534 | 2 |
| comp71616c0 | 720 | 2 |
| comp71626c0 | 1253 | 2 |
| comp71628c0 | 644 | 2 |
| comp71630c0 | 1064 | 2 |
| comp71632c0 | 668 | 2 |
| comp71645c0 | 71 | 2 |
| comp71647c0 | 707 | 2 |
| comp71649c0 | 1250 | 2 |
| comp71660c1 | 1838 | 2 |
| comp71666c0 | 742 | 2 |
| comp71669c0 | 1284 | 2 |
| comp71670c0 | 252 | 2 |
| comp71674c0 | 647 | 2 |
| comp71678c0 | 72 | 2 |
| comp71679c0 | 839 | 2 |
| comp71685c1 | 630 | 2 |
| comp71688c0 | 97 | 2 |
| comp71696c0 | 558 | 2 |
| comp71712c0 | 351 | 2 |
| comp71720c0 | 1595 | 2 |
| comp71721c0 | 570 | 2 |
| comp71722c0 | 207 | 2 |
| comp71728c0 | 704 | 2 |
| comp71729c0 | 1305 | 2 |
| comp71736c0 | 459 | 2 |
| comp71762c1 | 449 | 2 |
| comp71764c1 | 589 | 2 |
| comp71768c0 | 977 | 2 |
| comp71776c0 | 1262 | 2 |
| comp71780c0 | 1911 | 2 |
| comp71789c0 | 682 | 2 |
| comp71803c0 | 663 | 2 |
| comp71806c0 | 1168 | 2 |
| comp71814c0 | 947 | 2 |
| comp71824c1 | 102 | 2 |
| comp71836c0 | 811 | 2 |
| comp71838c0 | 488 | 2 |
| comp71844c0 | 314 | 2 |
| comp71854c0 | 969 | 2 |
| comp71862c1 | 665 | 2 |
| comp71868c0 | 143 | 2 |
| comp71875c3 | 217 | 2 |
| comp71879c0 | 131 | 2 |
| comp71882c0 | 650 | 2 |
| comp71884c1 | 1377 | 2 |
| comp71887c1 | 81 | 2 |
| comp71903c0 | 316 | 2 |
| comp71905c0 | 155 | 2 |
| comp71907c0 | 573 | 2 |
| comp71911c1 | 409 | 2 |
| comp71916c0 | 378 | 2 |
| comp71928c0 | 520 | 2 |
| comp71930c0 | 789 | 2 |
| comp71946c0 | 269 | 2 |
| comp71950c0 | 249 | 2 |
| comp71951c0 | 620 | 2 |
| comp71960c0 | 1225 | 2 |
| comp71976c0 | 324 | 2 |
| comp71984c0 | 706 | 2 |
| comp71991c0 | 402 | 2 |
| comp72001c0 | 370 | 2 |
| comp72003c0 | 541 | 2 |
| comp72004c0 | 612 | 2 |
| comp72010c0 | 857 | 2 |
| comp72012c0 | 1374 | 2 |
| comp72016c0 | 991 | 2 |
| comp72019c0 | 981 | 2 |
| comp72024c0 | 232 | 2 |
| comp72030c0 | 140 | 2 |
| comp72034c0 | 509 | 2 |
| comp72036c0 | 1026 | 2 |
| comp72038c0 | 871 | 2 |
| comp72040c0 | 1106 | 2 |
| comp72044c0 | 529 | 2 |
| comp72065c3 | 926 | 2 |
| comp72069c0 | 4798 | 2 |
| comp72070c0 | 36 | 2 |
| comp72087c0 | 826 | 2 |
| comp72093c0 | 2275 | 2 |
| comp72098c0 | 348 | 2 |
| comp72100c0 | 1152 | 2 |
| comp72108c0 | 1084 | 2 |
| comp72125c0 | 212 | 2 |
| comp72130c0 | 289 | 2 |
| comp72131c0 | 1011 | 2 |
| comp72132c0 | 1144 | 2 |
| comp72133c0 | 1180 | 2 |
| comp72134c0 | 1912 | 2 |
| comp72142c0 | 147 | 2 |
| comp72159c0 | 1566 | 2 |
| comp72163c2 | 547 | 2 |
| comp72165c0 | 622 | 2 |
| comp72167c0 | 1210 | 2 |
| comp72172c0 | 636 | 2 |
| comp72176c0 | 280 | 2 |
| comp72186c0 | 276 | 2 |
| comp72189c0 | 165 | 2 |
| comp72190c0 | 689 | 2 |
| comp72203c0 | 1026 | 2 |
| comp72206c0 | 1910 | 2 |
| comp72215c0 | 1346 | 2 |
| comp72219c0 | 537 | 2 |
| comp72222c0 | 875 | 2 |
| comp72227c0 | 108 | 2 |
| comp72239c0 | 996 | 2 |
| comp72244c0 | 1710 | 2 |
| comp72249c1 | 121 | 2 |
| comp72252c0 | 131 | 2 |
| comp72255c0 | 960 | 2 |
| comp72259c0 | 344 | 2 |
| comp72278c0 | 1005 | 2 |
| comp72279c0 | 726 | 2 |
| comp72280c0 | 1256 | 2 |
| comp72289c1 | 1190 | 2 |
| comp72289c2 | 1568 | 2 |
| comp72293c0 | 1211 | 2 |
| comp72296c0 | 382 | 2 |
| comp72303c1 | 112 | 2 |
| comp72305c0 | 219 | 2 |
| comp72315c0 | 1421 | 2 |
| comp72317c0 | 2635 | 2 |
| comp72331c0 | 1187 | 2 |
| comp72337c0 | 1062 | 2 |
| comp72343c0 | 1064 | 2 |
| comp72362c0 | 432 | 2 |
| comp72390c0 | 390 | 2 |
| comp72393c0 | 1342 | 2 |
| comp72397c0 | 501 | 2 |
| comp72406c0 | 1070 | 2 |
| comp72413c0 | 796 | 2 |
| comp72414c0 | 291 | 2 |
| comp72426c0 | 66 | 2 |
| comp72427c0 | 638 | 2 |
| comp72439c0 | 215 | 2 |
| comp72440c0 | 1465 | 2 |
| comp72442c0 | 147 | 2 |
| comp72444c0 | 1501 | 2 |
| comp72449c0 | 1331 | 2 |
| comp72451c0 | 586 | 2 |
| comp72463c0 | 440 | 2 |
| comp72464c1 | 229 | 2 |
| comp72467c0 | 475 | 2 |
| comp72473c0 | 1243 | 2 |
| comp72475c0 | 705 | 2 |
| comp72488c0 | 222 | 2 |
| comp72491c0 | 914 | 2 |
| comp72517c0 | 755 | 2 |
| comp72526c0 | 218 | 2 |
| comp72527c0 | 1900 | 2 |
| comp72530c0 | 529 | 2 |
| comp72533c0 | 798 | 2 |
| comp72540c2 | 913 | 2 |
| comp72548c0 | 108 | 2 |
| comp72551c0 | 922 | 2 |
| comp72553c0 | 1281 | 2 |
| comp72556c0 | 490 | 2 |
| comp72569c0 | 655 | 2 |
| comp72572c1 | 467 | 2 |
| comp72582c0 | 2541 | 2 |
| comp72593c1 | 782 | 2 |
| comp72617c0 | 2640 | 2 |
| comp72627c0 | 61 | 2 |
| comp72636c0 | 2565 | 2 |
| comp72639c0 | 467 | 2 |
| comp72654c0 | 2029 | 2 |
| comp72658c0 | 2319 | 2 |
| comp72673c2 | 433 | 2 |
| comp72681c0 | 633 | 2 |
| comp72703c0 | 1103 | 2 |
| comp72704c0 | 593 | 2 |
| comp72710c0 | 822 | 2 |
| comp72713c0 | 526 | 2 |
| comp72717c0 | 69 | 2 |
| comp72717c1 | 19 | 2 |
| comp72735c0 | 1116 | 2 |
| comp72790c0 | 2310 | 2 |
| comp72805c0 | 297 | 2 |
| comp72810c0 | 269 | 2 |
| comp72882c0 | 220 | 2 |
| comp72885c0 | 419 | 2 |
| comp72888c0 | 165 | 2 |
| comp72898c0 | 783 | 2 |
| comp72918c0 | 256 | 2 |
| comp72965c0 | 515 | 2 |
| comp72988c0 | 444 | 2 |
| comp72994c0 | 1114 | 2 |
| comp73230c0 | 849 | 2 |
| comp73303c0 | 2172 | 2 |
| comp73503c0 | 672 | 2 |
| comp73565c0 | 1169 | 2 |
| comp73649c0 | 123 | 2 |
| comp73683c0 | 424 | 2 |
| comp73720c0 | 568 | 2 |
| comp73787c0 | 1285 | 2 |
| comp73865c0 | 738 | 2 |
| comp73928c0 | 573 | 2 |
| comp73972c0 | 190 | 2 |
| comp74147c0 | 805 | 2 |
| comp74184c0 | 626 | 2 |
| comp74218c0 | 656 | 2 |
| comp74248c0 | 1301 | 2 |
| comp74296c0 | 1482 | 2 |
| comp74314c0 | 89 | 2 |
| comp74322c0 | 352 | 2 |
| comp74356c0 | 669 | 2 |
| comp74639c0 | 1053 | 2 |
| comp74686c0 | 1499 | 2 |
| comp74836c0 | 248 | 2 |
| comp74861c0 | 7601 | 2 |
| comp74888c0 | 355 | 2 |
| comp74950c0 | 5839 | 2 |
| comp74970c0 | 959 | 2 |
| comp75187c0 | 1716 | 2 |
| comp75479c0 | 581 | 2 |
| comp75483c0 | 638 | 2 |
| comp75524c0 | 537 | 2 |
| comp75608c0 | 754 | 2 |
| comp76144c0 | 379 | 2 |
| comp76494c0 | 1186 | 2 |
| comp76747c0 | 367 | 2 |
| comp767c0 | 1358 | 2 |
| comp768110c0 | 1537 | 2 |
| comp77117c0 | 778 | 2 |
| comp77821c0 | 1141 | 2 |
| comp78292c0 | 907 | 2 |
| comp78314c0 | 369 | 2 |
| comp78339c0 | 1657 | 2 |
| comp78498c0 | 905 | 2 |
| comp78813c0 | 715 | 2 |
| comp79618c0 | 108 | 2 |
| comp79626c0 | 618 | 2 |
| comp80174c0 | 1177 | 2 |
| comp80302c0 | 597 | 2 |
| comp805959c0 | 161 | 2 |
| comp82165c0 | 398 | 2 |
| comp82818c0 | 634 | 2 |
| comp8313c0 | 1240 | 2 |
| comp83992c0 | 631 | 2 |
| comp8546c0 | 473 | 2 |
| comp858275c0 | 996 | 2 |
| comp860016c0 | 136 | 2 |
| comp86458c0 | 1275 | 2 |
| comp875293c0 | 1061 | 2 |
| comp8785c0 | 858 | 2 |
| comp88456c0 | 653 | 2 |
| comp88961c0 | 1163 | 2 |
| comp890994c0 | 202 | 2 |
| comp93703c0 | 2909 | 2 |
| comp96009c0 | 108 | 2 |
| comp972888c0 | 138 | 2 |
| comp974691c0 | 1373 | 2 |
| comp98140c0 | 424 | 2 |
| comp982588c0 | 82 | 2 |
| comp991233c0 | 1107 | 2 |
| comp992498c0 | 59 | 2 |
| comp99554c0 | 1135 | 2 |
| comp35301c0 | 555 | 1 |
| comp43045c0 | 453 | 1 |
| comp43851c0 | 391 | 1 |
| comp462443c0 | 476 | 1 |
| comp51449c0 | 637 | 1 |
| comp53330c1 | 275 | 1 |
| comp55373c0 | 475 | 1 |
| comp56141c0 | 45 | 1 |
| comp58255c0 | 1016 | 1 |
| comp59910c0 | 1323 | 1 |
| comp61553c0 | 717 | 1 |
| comp62175c0 | 1453 | 1 |
| comp63301c0 | 41 | 1 |
| comp63328c0 | 245 | 1 |
| comp63671c0 | 802 | 1 |
| comp64047c0 | 514 | 1 |
| comp65267c0 | 440 | 1 |
| comp65307c0 | 152 | 1 |
| comp65389c0 | 742 | 1 |
| comp65777c0 | 617 | 1 |
| comp65821c0 | 185 | 1 |
| comp65881c0 | 533 | 1 |
| comp65911c0 | 762 | 1 |
| comp65925c0 | 1284 | 1 |
| comp67214c0 | 684 | 1 |
| comp67347c0 | 2210 | 1 |
| comp67488c0 | 292 | 1 |
| comp68189c1 | 37 | 1 |
| comp68351c0 | 567 | 1 |
| comp68460c0 | 1037 | 1 |
| comp68475c0 | 824 | 1 |
| comp68774c0 | 862 | 1 |
| comp69124c1 | 1907 | 1 |
| comp69925c0 | 214 | 1 |
| comp70019c0 | 1514 | 1 |
| comp70130c0 | 738 | 1 |
| comp70288c0 | 243 | 1 |
| comp70464c0 | 737 | 1 |
| comp71363c0 | 182 | 1 |
| comp71717c0 | 51 | 1 |
| comp72023c0 | 532 | 1 |
| comp72072c0 | 280 | 1 |
| comp72262c0 | 130 | 1 |
| comp73227c0 | 761 | 1 |
| comp1174c0 | 12 | 0 |
| comp11780c0 | 466 | 0 |
| comp20184c0 | 18 | 0 |
| comp2022c0 | 2 | 0 |
| comp2555c0 | 20 | 0 |
| comp35485c0 | 459 | 0 |
| comp371746c0 | 38 | 0 |
| comp45470c0 | 1 | 0 |
| comp47231c0 | 1603 | 0 |
| comp50788c0 | 3 | 0 |
| comp51205c0 | 5 | 0 |
| comp51568c0 | 25 | 0 |
| comp52585c0 | 0 | 0 |
| comp53108c0 | 0 | 0 |
| comp53940c0 | 0 | 0 |
| comp54000c0 | 59 | 0 |
| comp55523c0 | 834 | 0 |
| comp56666c0 | 634 | 0 |
| comp57758c0 | 66 | 0 |
| comp58239c1 | 551 | 0 |
| comp58354c0 | 12 | 0 |
| comp61589c0 | 7 | 0 |
| comp63533c0 | 628 | 0 |
| comp64830c0 | 2 | 0 |
| comp64846c0 | 297 | 0 |
| comp706259c0 | 18 | 0 |
| comp715113c0 | 12 | 0 |
| comp71816c1 | 75 | 0 |
| comp72340c0 | 534 | 0 |
| comp72649c0 | 4 | 0 |
| comp74823c0 | 255 | 0 |
| comp760864c0 | 0 | 0 |
| comp779809c0 | 127 | 0 |
